# Tuning Anion Composition and Mobility to Balance Ionic Conductivity and Cation Selectivity in Solid Polymer Electrolytes

**DOI:** 10.1021/acs.macromol.5c03173

**Published:** 2026-04-14

**Authors:** Mengying Yang, Thomas H. Epps

**Affiliations:** † Department of Materials Science and Engineering, 5972 University of Delaware, Newark, Delaware 19716, United States; ‡ Department of Chemical and Biomolecular Engineering, University of Delaware, Newark, Delaware 19716, United States; § Center for Research in Soft matter and Polymers (CRiSP), University of Delaware, Newark, Delaware 19716, United States

## Abstract

Solid polymer electrolytes (SPEs) offer a promising route toward safe and high-performance electrochemical energy storage, yet a fundamental challenge in SPEs involves improving ionic conductivity while maintaining selective cation transport. The hurdle exists because ion transport is typically coupled closely to polymer segmental dynamics. Herein, a glassy single-ion-conducting polymer, poly­[lithium sulfonyl­(trifluoromethane sulfonyl)­imide methacrylate] (PLiMTFSI), in which the anions were tethered to the polymer, was blended with a flexible polymer, poly­(*oligo*-oxyethylene methyl ether methacrylate) (POEM), and a series of small-molecule lithium salts, in which the anions were untethered [lithium bis­(trifluoromethanesulfonyl)­imide (LiTFSI), lithium bis­(fluorosulfonyl)­imide (LiFSI), lithium trifluoromethanesulfonate (LiTf), or lithium perchlorate (LiClO_4_)]. The impact of salt anion volume and tethered-to-untethered anion ratio on the ion conduction behavior and thermal properties of blend electrolytes was investigated. In some cases, conductivity could be enhanced through this ternary blend approach. For example, a POEM-based polymer blend containing a bulky salt anion (TFSI^–^) and an equimolar mixture of PLiMTFSI and LiTFSI exhibited a Li^+^ conductivity (4.8 × 10^–4^ S/cm) an order of magnitude higher than that of a comparable POEM/LiTFSI system (6.3 × 10^–5^ S/cm) at 100 °C. This enhancement was attributed to a more than 9-fold increase in lithium transference number (0.66 in the ternary blend vs 0.07 in POEM/LiTFSI). Overall, this study highlights the potential for tuning anion composition and mobility to achieve relatively high ionic conductivities and maintain selective cation transport in SPEs, offering a pathway to enable batteries that tolerate elevated temperatures.

## Graphical abstract

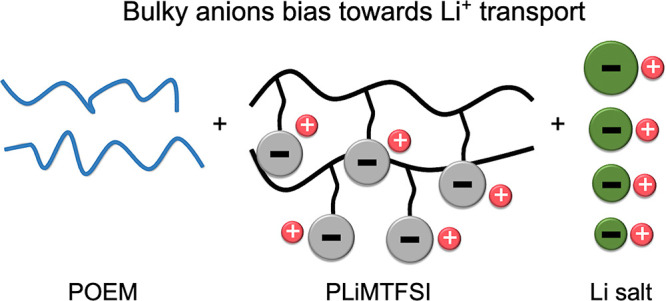

## Introduction

The increasing demand for high-performance energy storage has intensified the search for next-generation batteries with higher energy and power densities, and enhanced safety.^1–4^ The electrolyte is a critical component in a battery as it controls the transport of ions and impacts long-term battery stability.^5^ Commercial liquid electrolytes, in which lithium salts are dissolved in flammable organic solvents, have overall conductivities (σ_overall_s) of ∼10^–3^ to 10^–2^ S/cm at −10 to 80 °C),^6,7^ but their flammability poses a safety concern.^8,9^ Moreover, these liquid electrolytes suffer from relatively low lithium transference numbers (*t*_Li+_s, representing the fraction of current carried by Li^+^),^10^ e.g., 0.3–0.4, due to the unconstrained mobility of both cations and anions.^11^ The low *t*_Li+_s can result in salt concentration gradients, which eventually lead to salt precipitation on one electrode and depletion on the other, significantly increasing the internal resistance of the battery.^6,11^ These performance limitations can be largely mitigated in SPEs with a higher *t*_Li+_ (∼0.7 to 1.0).^6^ Yet, enhanced *t*_Li+_s present a challenge because, in conventional SPEs, Li^+^ binds more strongly to the polymer than the anion does, leading to slower Li^+^ diffusion and inherently limiting *t*_Li+_s.

One widely studied SPE is poly­(ethylene oxide) doped with LiTFSI (PEO/LiTFSI).^12–15^ This system has reasonably high σ_overall_s (∼10^–3^ S/cm at ≥ 70 °C).^12,16^ In this type of SPE, ion conduction primarily depends on the segmental motion of the amorphous polymer regions to create dynamic transport pathways.^17^ As a result, ions move when polymer segments rearrange to form solvation environments that enable diffusion.^18^ Despite having comparable σ_overall_s to those of commercial liquid electrolytes at elevated temperatures (∼10^–3^ S/cm at ≥ 70 °C),^12,16^ the tighter coordination between Li^+^ and PEO vs between the anion and PEO results in extremely low *t*_Li+_s (0.1–0.2 vs. 0.3–0.4 for common commercial liquid electrolytes).^10,19^ To increase *t*_Li+_s in PEO-based systems, the alteration of the Li^+^ solvation environment^20,21^ or the incorporation of nanofillers has shown promise. For instance, the addition of a non-ion-conducting Al_2_O_3_ ceramic filler^22^ or ball-milled ion-conducting Li_6.4_La_3_Zr_1.4_Ta_0.6_O_12_ filler^23^ into PEO increased *t*_Li+_s to ∼0.5 with less than an order of magnitude reduction in σ_overall_s;^22^ yet, particle agglomeration and poor interfacial contact with the electrodes are challenges in these systems,^2^ impacting their suitability for long-term battery operations.

To overcome the drawbacks of composite electrolytes, one strategy is to covalently tether anions to the polymers, resulting in *t*_Li+_s of ∼1. Known as single-ion-conducting (SIC) polymer electrolytes, these materials offer enhanced energy density, longer cycle life, and improved safety.^24^ Despite the above-mentioned advantages, strong electrostatic interactions between Li^+^ and the tethered anion significantly reduce the number of free charge carriers.^25^ Additionally, SIC SPEs typically have higher glass transition temperatures (*T*_g_s, > 60 °C),^26,27^ which greatly restrict segmental dynamics in comparison to PEO-based electrolytes (*T*_g_ = ∼−60 °C). These factors contribute to the poor conductivities in pure SIC SPEs, typically around 10^–6^ S/cm or lower at 100 °C.^26–28^ To enhance σ_overall_s while maintaining high *t*_Li+_s in SIC polymers, various approaches have been developed. For example, a SIC SPE with an alternating-like polymer sequence of lithium 2-(trifluoromethyl)-N-[(trifluoromethyl)­sulfonyl]-2-propenamide and PEO-based segments had relatively high *t*_Li+_s (∼0.9) while preserving σ_overall_s of ∼10^–4^ S/cm at 90 °C.^25^ Highly delocalized anions,^29^ tunable polymer topology,^30^ and tailored polymer architecture^31–34^ also have shown promise in enhancing σ_overall_s in SIC SPEs. Despite encouraging results in specific systems, ion transport remained closely coupled to slow polymer segmental motion, limiting the extent to which both σ_overall_s and *t*_Li+_s could be simultaneously increased.

The decoupling of ion transport from polymer segmental dynamics is one alternative approach to increase σ_overall_s and maintain selective cation transport. For instance, in polymeric zwitterionic liquids (PZILs), manipulation of IL chemistry created a large size disparity between Li^+^ and the constituent PZIL ions, which in turn generated sufficient free volume for decoupled Li^+^ transport (*t*_Li+_s of ∼0.67) with reasonably high σ_overall_s (∼10^–4^ S/cm at 100 °C).^2^ As another example, a polystyrene-based or polymethacrylate-based SIC polymer was blended with a PEO; σ_overall_s around 10^–4^ to 10^–3^ S/cm at 100 °C with a *t*_Li+_s of ∼0.9 to 1.0 were achieved, with ion transport occurring through percolating aggregate networks.^27,35^ This higher and selective ion transport was attributed to the percolating free volume generated by the SIC polymer chain packing frustration.^35^

Herein, a polymethacrylate-based SIC SPE with a delocalized anion^35^ was blended with a POEM and various small-molecule lithium salts. The ion conduction behavior and thermal properties of the blends were investigated as a function of anion size and molar ratio of SIC polymer to small-molecule salt. The anion composition and mobility were adjusted to induce a transition in the Li^+^ transport mechanism from a ‘coupled-to-polymer segmental motion’ to a ‘decoupled, hopping-like’ mechanism. As a result, a polymer blend containing a bulky salt anion (TFSI^–^) and an equimolar mixture of PLiMTFSI and LiTFSI-referred to as the mixed-salt blend, in which a portion of the anions were tethered and the rest remained untethered, demonstrated Li^+^ conductivities (σ_Li+_s) an order of magnitude higher than those of POEM/LiTFSI, due to a more than 9-fold increase in *t*_Li+_. Additionally, *t*_Li+_s remained largely unchanged between 60 and 100 °C, indicating that temperature had a limited impact on cation selectivity within this range. Both σ_overall_s and σ_Li+_s exhibited a nonmonotonic trend as the tethered-to-untethered anion ratio varied. Moreover, increasing anion volumes led to simultaneous enhancements in σ_overall_s, *t*_Li+_s, and σ_Li+_s, highlighting the effectiveness of leveraging bulky, delocalized anions to achieve decoupled ion transport and improve overall ion conduction properties.

## Experimental Section

### Chemicals

*Oligo*-oxyethylene methyl ether methacrylate (OEM, > 99%, stabilized, average molar mass = 500 g/mol, Sigma-Aldrich, USA) was purified by passage through a basic aluminum oxide column. Lithium bis­(trifluoromethanesulfonyl)­imide (LiTFSI, anhydrous, 99.99% trace metals basis), lithium bis­(fluorosulfonyl)­imide (LiFSI, ultra dry, 99.9% trace metals basis, battery grade), lithium trifluoromethanesulfonate (LiTf, 99.995% trace metals basis), and lithium perchlorate (LiClO_4_, anhydrous, ≥ 99.9% trace metals basis), all from Sigma-Aldrich, USA, were dried under dynamic vacuum at 150 °C for 48 h to remove moisture and then stored in an argon-filled glovebox. Lithium sulfonyl­(trifluoromethane sulfonyl)­imide methacrylate (LiMTFSI, 99%, stabilized, Specific Polymers, France), copper bromide [CuBr_2_, 98%, Acros Organics, USA], tris­(2-pyridylmethyl)­amine (TPMA, >98%, Tokyo Chemical Industry, Japan), ethyl 2-bromoisobutyrate (EBib, 98%, Sigma-Aldrich, USA), tin­(II) 2-ethylhexanoate [Sn­(Oct)_2_, 92.5–100.0%, Sigma-Aldrich, USA], anisole (>99%, Fisher Scientific, USA), dimethylformamide (DMF, Fisher Scientific, USA), diethyl ether (Fisher Scientific, USA), petroleum ether (Fisher Scientific, USA), acetone (Optima, Fisher Scientific, USA), acetone-*d*_6_ (with 0.03 v/v % tetramethylsilane, 99.9 at % D, Thermo Scientific, USA), chloroform-*d* (with 0.02–0.04 v/v % tetramethylsilane, 99.8+ at % D, Thermo Scientific, USA), and lithium bromide (LiBr, ≥99%, Sigma-Aldrich, USA) were used as received.

### Synthesis of Poly­(lithium Sulfonyl­(trifluoromethane Sulfonyl)­imide Methacrylate) (PLiMTFSI) and Poly­(*oligo*-oxyethylene Methyl Ether Methacrylate) (POEM)

PLiMTFSI and POEM homopolymers were synthesized using Activators ReGenerated by Electron Transfer Atom Transfer Radical Polymerization (ARGET ATRP), as described in the literature.^35^ For the PLiMTFSI polymerization, the reaction proceeded for 17 h, reaching a desired conversion of ∼60%. The reaction conversion was tracked via Proton Nuclear Magnetic Resonance (^1^H NMR, Bruker AV600III) spectroscopy using acetone-*d*_6_ as the solvent. The polymer was purified as described in the literature.^35^ For the POEM polymerization, the reaction proceeded for 3 h, at which point, a ∼60% conversion was reached, as determined by NMR spectroscopy in chloroform-*d*. POEM was purified following the protocols described in the literature.^35^ All polymers were stored in an argon-filled glovebox at −4 °C prior to use.

### Blend Electrolyte Film Fabrication

POEM, PLiMTFSI, and Li salt (LiTFSI, LiFSI, LiTf, and LiClO_4_) stock solutions were prepared in an argon-filled glovebox by dissolving each material separately in anhydrous DMF at ∼10 wt % and then stirring for 24 h. Next, the appropriate POEM and LiTFSI stock solutions (for untethered-salt POEM/LiTFSI blend sample, in which all anions in the salt were untethered), POEM and PLiMTFSI stock solutions (for tethered-salt POEM/PLiMTFSI blend samples, in which all anions in the salt were tethered), or POEM, PLiMTFSI, and Li salt stock solutions (for mixed-salt POEM/PLiMTFSI/Li salt blend samples) were mixed at varying gravimetric ratios and stirred for an additional 48 h. The overall Li^+^ concentration was kept consistent in all samples at [EO]:[Li^+^] of 10:1, which represents the molar ratio of ethylene oxide monomer segments in POEM side chains to Li^+^. In the mixed-salt POEM/PLiMTFSI/Li salt samples, the molar ratios of [EO]:[LiMTFSI]:[Li salt] were varied at 10:0.05:0.95, 10:0.15:0.85, 10:0.25:0.75, 10:0.40:0.60, 10:0.50:0.50, 10:0.60:0.40, and 10:0.80:0.20, respectively.

Next, the blend electrolyte solutions were recovered from the glovebox and drop-cast onto Teflon O-ring molds to form films with a thickness (*L*) of 0.05 cm and an area (*A*) of 0.32 cm^2^. To ensure uniform thickness and prevent bubble formation, the majority of the DMF was slowly evaporated under an inert atmosphere. Once the films had formed, they were sealed in drying chambers and dried under dynamic vacuum at 25 °C until the Schlenk line baseline pressure was reached, which typically took ∼20 h. To remove any residual solvent and moisture, the temperature was gradually increased to 150 °C in 10 °C increments. After reaching the Schlenk line baseline pressure (∼6 h), the films were dried at 150 °C under dynamic vacuum for 48 h. The dried films were stored in an argon-filled glovebox at −4 °C prior to characterization.

### Size Exclusion Chromatography (SEC)

SEC was performed using a Tosoh HLC-8420 EcoSEC Elite Gel Permeation Chromatography instrument with 0.1 vol % LiBr in DMF as the eluent at 25 °C (0.8 mL/min). The system included two PL aquagel–OH Mixed-H (8 μm, 50 × 7.5 mm) columns. A calibration curve was constructed using narrow-dispersity poly­(methyl methacrylate) standards (885–2,210,000 g/mol, Agilent Technologies, USA). Samples were prepared by dissolving ∼5 mg polymer in ∼5 mL of mobile phase, followed by 24 h equilibration at 25 °C to ensure complete dissolution. Before measurement, all samples were filtered through 0.1-μm polytetrafluoroethylene (PTFE) filters.

### Thermogravimetric Analysis (TGA)

Blend electrolyte samples (2–3 mg) were placed in 100-μL platinum pans and heated under continuous nitrogen flow at a flow rate of 50 mL/min. Measurements were performed using a TA Instruments (Discovery TGA5500 instrument). The samples were heated at a rate of 20 °C/min to 115 °C, annealed at 115 °C for 1 h to remove any moisture absorbed during sample loading, then cooled to 50 °C at a rate of 20 °C/min, held at 50 °C for 1 min, and heated at 15 °C/min to 600 °C.

### Differential Scanning Calorimetry (DSC)

Blend electrolyte samples (∼6 mg) were hermetically sealed in 40-μL aluminum pans in an argon-filled glovebox. Measurements were performed on a TA Instruments Discovery DSC instrument equipped with an RCS90 cooling accessory. Baseline and cell constant calibrations were conducted using sapphire disks and an indium standard, respectively. Three heating/cooling cycles were performed at a 10 °C/min ramp rate under a nitrogen environment from −80 to 150 °C (for untethered-salt POEM/LiTFSI blend) or −80 to 190 °C (for tethered-salt POEM/PLiMTFSI blend and mixed-salt POEM/PLiMTFSI/Li salt blends). The reported *T*_g_ values were determined from the midpoints of the inflections in the second heating traces.

### Small-Angle X-ray Scattering (SAXS) and Wide-Angle X-ray Scattering (WAXS)

SAXS and WAXS measurements were performed on a Xenocs Xeuss 2.0 instrument with a sealed-tube X-ray source (Cu Kα, λ = 1.54 Å) operating at 2.0 kW. A Dectris Pilatus 300k 2-D detector with a 2000 mm (SAXS) and 72 mm (WAXS) sample-to-detector distance was used. All samples were prepared in an argon-filled glovebox to prevent moisture absorption by sealing the material between two Kapton films within a rubber o-ring in a homemade sample holder. SAXS and WAXS profiles were acquired under dynamic vacuum at 80, 120, and 150 °C with a 3 h preanneal at each temperature using a Linkam HFSX350-CAP stage. All 2-D scattering data were azimuthally integrated, resulting in plots of scattered intensity versus scattering vector, *q* (for SAXS profiles), and scattering angle, 2θ (for WAXS profiles).

### Alternating Current (AC) Impedance Spectroscopy

Ionic conductivities were measured using a Princeton Applied Research PARSTAT 2273 frequency response analyzer with a custom-built test cell mounted on a Linkam HFS91 CAP stage, under vacuum in a standalone chamber. Each test cell comprised two stainless-steel electrodes and two aluminum foil electrodes with a Teflon o-ring spacer in the middle. The blend electrolyte film was placed between the aluminum electrodes, and the cell was assembled in an argon-filled glovebox. Prior to the measurement, all samples were annealed at 60 °C for 3 h in the sealed test cell to ensure good contact with the aluminum electrodes. Then, the samples were cooled to 25 °C and held at that temperature for 1 h. The ionic conductivities were measured during heating from 30 to 150 °C in 10 °C increments. At each temperature, two impedance measurements were taken after annealing for 5 and 8 min, respectively. The reported ionic conductivities at each temperature represent the average of these two measurements, with differences typically within 1%, indicating stable and reproducible results. Measurements were conducted over an AC frequency range of 0.1 Hz to 1.0 MHz with a 10-mV voltage amplitude, ensuring operation within the linear response regime for simplified analysis. The bulk resistance (*R*_bulk_) of the blend electrolyte was determined as the touchdown point in the Nyquist plot.^36–38^ The ionic conductivity (σ) then was calculated using eq 1,

$$\sigma =\frac{L}{R_{\mathrm{bulk}}\times A}$$ 1

### Potentiostatic Polarization

Lithium transference numbers (*t*_Li+_s) were measured at 60 and 100 °C using potentiostatic polarization and AC impedance spectroscopy under vacuum on the PARSTAT 2273. The measurements were conducted in lithium–lithium symmetric cells mounted on a Linkam HFS91 CAP stage. Each cell consisted of two stainless-steel electrodes and two lithium foil electrodes, with a Teflon o-ring spacer in the middle. The blend electrolyte film was sandwiched between the lithium electrodes, and the cell was assembled in an argon-filled glovebox. For each temperature, three replicate lithium–lithium symmetric cells were prepared. Prior to any electrochemical measurement, the cell was equilibrated at 60 or 100 °C for 3 h. An initial AC impedance spectroscopy measurement then was conducted prior to the potentiostatic polarization, with an AC frequency range of 0.1 Hz–1.0 MHz and a voltage amplitude of 10 mV, and the initial impedance was recorded as a function of frequency. After this measurement, the cell was polarized with a potential of 10 mV (a small polarization voltage was chosen to avoid any significant electrochemical reactions that might affect the results). The initial current, *I*_0_, was calculated by Ohm’s law using the applied potential and the total resistance.^39–44^ The current was closely monitored during the polarization process, and the potential was applied for 8 h, at which point a steady-state current was reached. A second AC impedance spectroscopy measurement then was performed to extract the steady-state impedance. *t*_Li+_ was determined using the Bruce-Vincent method (eq 2),^40^

$$t_{{\mathrm{Li}}^{+}}=\frac{I_{\mathrm{SS}}(\Delta V-I_{0}R_{0})}{I_{0}(\Delta V-I_{\mathrm{SS}}R_{\mathrm{SS}})}$$ 2

in which *I*_SS_ and *I*_0_ are the steady-state and initial current, respectively, Δ*V* is the polarization voltage, and *R*_SS_ and *R*_0_ are the steady-state and initial interfacial impedance, respectively. The interfacial impedance was taken as the difference between the values of the minima at the bounds of the low-frequency semicircle in a Nyquist plot.^38,44^ The reported *t*_Li+_ value was taken as the average of the data obtained from three lithium–lithium cells. The lithium foil (0.02 mm thickness, 99.9% lithium content, MTI Corporation, USA) was used as received. Lithium foil electrodes with a diameter of 0.95 cm were prepared using a hollow punch with a 0.95 cm cutting head (Mayhew Steel Products, Inc., USA) in an argon-filled glovebox.

## Results and Discussion

### Ion Transport Behavior can be Readily Modulated by the Anion van der Waals Volume

To investigate the impact of salt anion van der Waals volume (*V*_vdW_, which is defined as the volume occupied by a molecule that is impenetrable to other molecules)^45^ and the molar ratio of untethered-to-tethered anion on the ion conduction behavior, POEM and PLiMTFSI with a number-average molecular weight of 5.9 and 105.7 kg/mol, respectively, were synthesized via ARGET ATRP.^35^ POEM, PLiMTFSI, and four different lithium salts (LiTFSI, LiFSI, LiTf, and LiClO_4_; see Scheme 1 for polymer synthesis overview and chemical structures) were blended at various molar ratios of [EO]:[LiMTFSI]:[Li salt] (10:0.05:0.95, 10:0.15:0.85, 10:0.25:0.75, 10:0.40:0.60, 10:0.50:0.50, 10:0.60:0.40, and 10:0.80:0.20) while keeping the overall Li^+^ concentration constant at [EO]:[Li^+^] = 10:1 for all the blends. Herein, a high-molecular-weight, semi-flexible PLiMTFSI (105.7 kg/mol) was selected to suppress anion motion and increase free volume arising from chain-packing frustration. An increase in the molecular weight of a semiflexible polymer slows chain dynamics and promotes a more packing-frustrated system, in which chains struggle to adopt a closely packed structure, facilitating ion hopping.^46^ The molecular weight of POEM was low (5.9 kg/mol, i.e., oligomeric) to maximize POEM segmental motion and conductivity of the homopolymer.^47^ PEO/LiTFSI served as a reference system, in which ionic conductivity decreases with increasing molecular weight at low values and then plateaus beyond a critical molecular weight of ∼5 kg/mol.^47^ Although the critical molecular weights of POEM and PEO may differ due to differences in chemical structure, PEO provides a useful benchmark for guiding molecular weight selection of the POEM. A commercially available small-molecule lithium salt was incorporated because its range of anion sizes offers a practical and versatile means to tune the degree of ion dissociation. This strategy avoids chemical modification of PLiMTFSI, which may be less scalable and more challenging than blending. Additionally, conducting polymers such as PEO, POEM, and their derivatives doped with small-molecule salts have been well established in the literature.^18,20,48–51^ In contrast, the blending of a SIC polymer and a small-molecule lithium salt is relatively less explored in comparison to the former systems, generally due to limited segmental motion and poor ion dissociation, resulting in relatively low ionic conductivities (i.e., ∼10^–6^ S/cm at 90 °C).^52^ The combination of a semi-flexible SIC polymer, an ion-conducting polymer, and a small-molecule lithium salt with systematically varied anion volumes and chemistries has not been previously reported within a single, systematic framework to the authors’ knowledge. The use of the small-molecule salt enables facile tuning of ion dissociation strength, while the resulting platform allows a comprehensive investigation of how system composition influences ionic conduction and thermal properties and modulates the trade-off between high ionic conductivity and lithium-ion transference number.

**Scheme 1:**
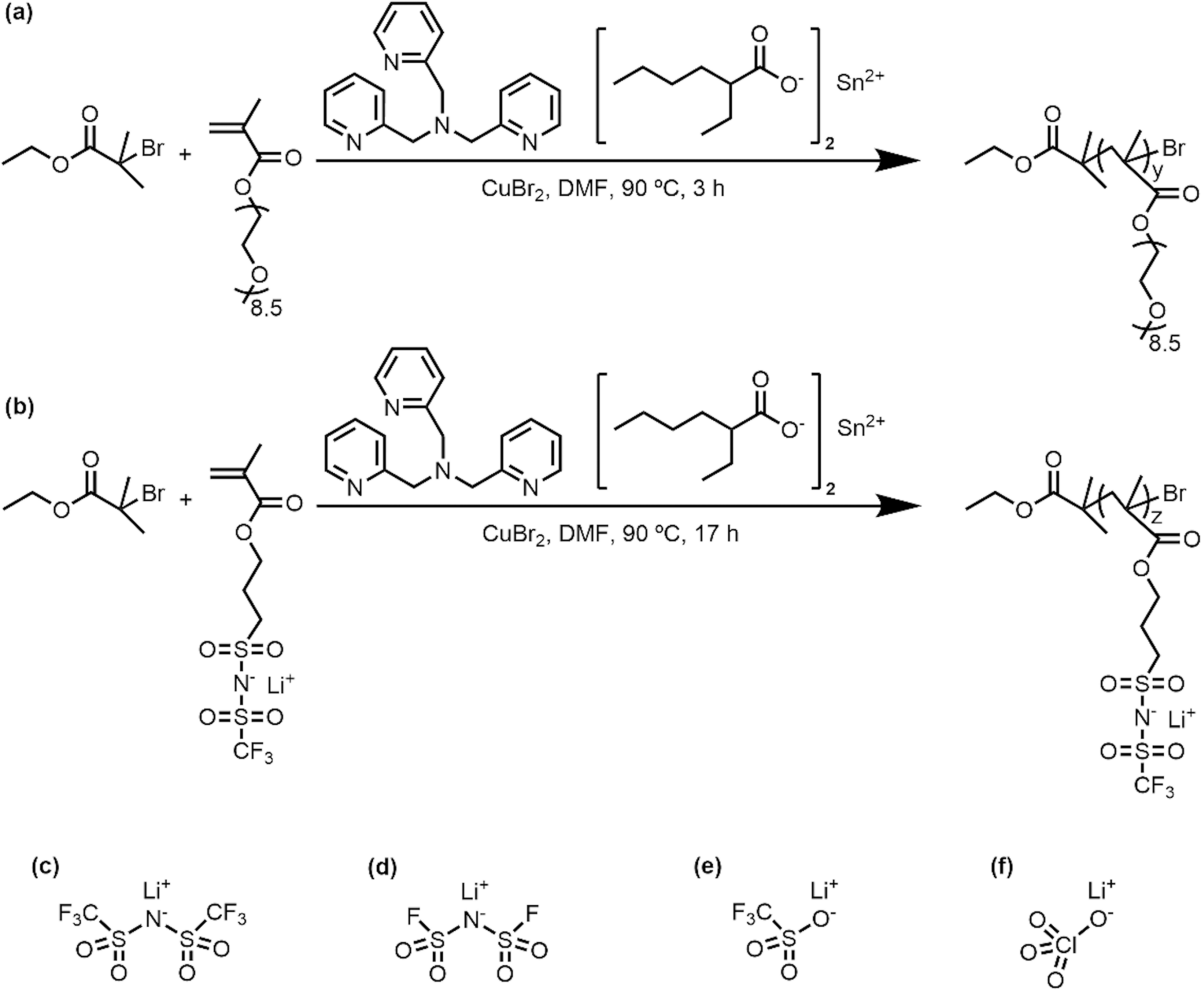
Synthesis Schemes for (a) POEM and (b) PLiMTFSI^a^

a Chemical structures of (c) LiTFSI, (d) LiFSI, (e) LiTf, and (f) LiClO_4_.

As determined from DSC, all blends exhibited a single *T*_g_ (*T*_g,blend_, see Figures S5–S9), suggesting that the two polymer components were miscible.^53–57^ Miscibility also was supported by the featureless SAXS profiles^58^ in the tethered-salt and untethered-salt blends (see Figures S15–S43). Ion aggregates of PLiMTFSI existed in all the samples (see Figures S14–S43), which is consistent with the literature reports.^59,60^ The thermal stability of the mixed-salt blends was investigated by TGA (see Figures S10–S13), and all samples exhibited 1% weight loss temperatures (*T*_d1%_s) above 230 °C. TGA was conducted to identify a safe temperature range for DSC and transport experiments, ensuring that potential thermal degradation would not interfere with the evaluation of salt dissolution and conduction. For LiFSI, which has a melting point of 145 °C (well below *T*_d1%_),^61^ the absence of a melting peak in the DSC traces indicated complete dissolution of LiFSI in the polymers (see Figure S7). In contrast, the melting points of LiTFSI (234 °C),^62^ LiTf (>300 °C),^63^ and LiClO_4_ (236 °C)^64^ exceeded *T*_d1%_ values of the corresponding blend samples. Thus, WAXS was employed to detect the presence of salt crystallinity. The absence of crystalline peaks for LiTFSI and LiTf in the associated mixed-salt blends suggested that both salts were well solvated by the polymers (see Figures S16–S22 and Figures S30–S36). In the case of mixed-salt blends containing LiClO_4_, complete solvation was only noted at [EO]:[LiMTFSI]:[LiClO_4_] molar ratios of 10:0.05:0.95, 10:0.15:0.85, 10:0.25:0.75, and 10:0.40:0.60 (see Figures S37–S40). At [EO]:[LiMTFSI]:[LiClO_4_] molar ratios of 10:0.50:0.50, 10:0.60:0.40, and 10:0.80:0.20, the sharp peak at 47° indicated the presence of unsolvated LiClO_4_ (see Figures S41–S43).^65^ Additionally, ion aggregates of PLiMTFSI existed in all the samples (see Figures S14–S43), which is consistent with literature reports.^59,60^

The temperature-dependent σ_overall_s were determined by AC impedance spectroscopy; see Figure 1a for the mixed-salt ternary blends at a molar ratio of [EO]:[LiMTFSI]:[Li salt] of 10:0.50:0.50. σ_overall_s as a function of temperature for other molar ratios of [EO]:[LiMTFSI]:[Li salt] (10:0.05:0.95, 10:0.15:0.85, 10:0.25:0.75, 10:0.60:0.40, and 10:0.80:0.20) are located in Figures S44–S48. The data were fit to Vogel–Tammann–Fulcher (VTF) or Arrhenius relationships using the following delineations. Conductivity results that were well described (*R* ^2^ ≥ 0.99) by the VTF equation (eq 3)^66^ are shown as dashed lines,

$$\sigma =\sigma_{0}\exp\left[\frac{-E_{a}}{R\left(T-T_{0}\right)}\right]$$ 3

in which σ_0_ is the ionic conductivity at infinitely high temperature and is proportional to the number of charge carriers,^36^ *E*_a_ is the pseudo activation energy associated with polymer segmental dynamics, *R* is the ideal gas constant, *T* is the absolute temperature in Kelvin, and *T*_0_ is the ideal *T*_g_ at which the free volume or configurational entropy becomes zero.^66^ Herein, *T*_0_ was chosen to be 50 K below *T*_g,blend_, which is common for polyether-containing systems.^67–71^ VTF-type behavior suggests that ion transport is closely coupled to polymer segmental relaxation.^60,66,72^ Conductivity results that were well described (*R* ^2^ ≥ 0.99) by the Arrhenius equation (eq 4)^73^ are shown as solid lines,

$$\sigma =\sigma_{0}\exp\left(\frac{-E_{a}}{RT}\right)$$ 4

in which *E*_a_ is the pseudo activation energy associated with ion hopping.^73^ Arrhenius-type behavior may imply that ion transport is a thermally activated process as ions hop from one available site to another.^60,73^ This type of ion transport is generally considered decoupled from polymer segmental relaxation.^46,72–77^ Decoupled ion transport also can be identified through a modified Walden plot analysis, in which molar conductivity is plotted against the inverse of segmental relaxation time,^46^ which will be explored in future studies. In the present work, the combination of temperature-dependent conductivity trends and the changes in activation behavior provides meaningful insight into the evolving ion transport characteristics of these systems.

**Figure 1:**
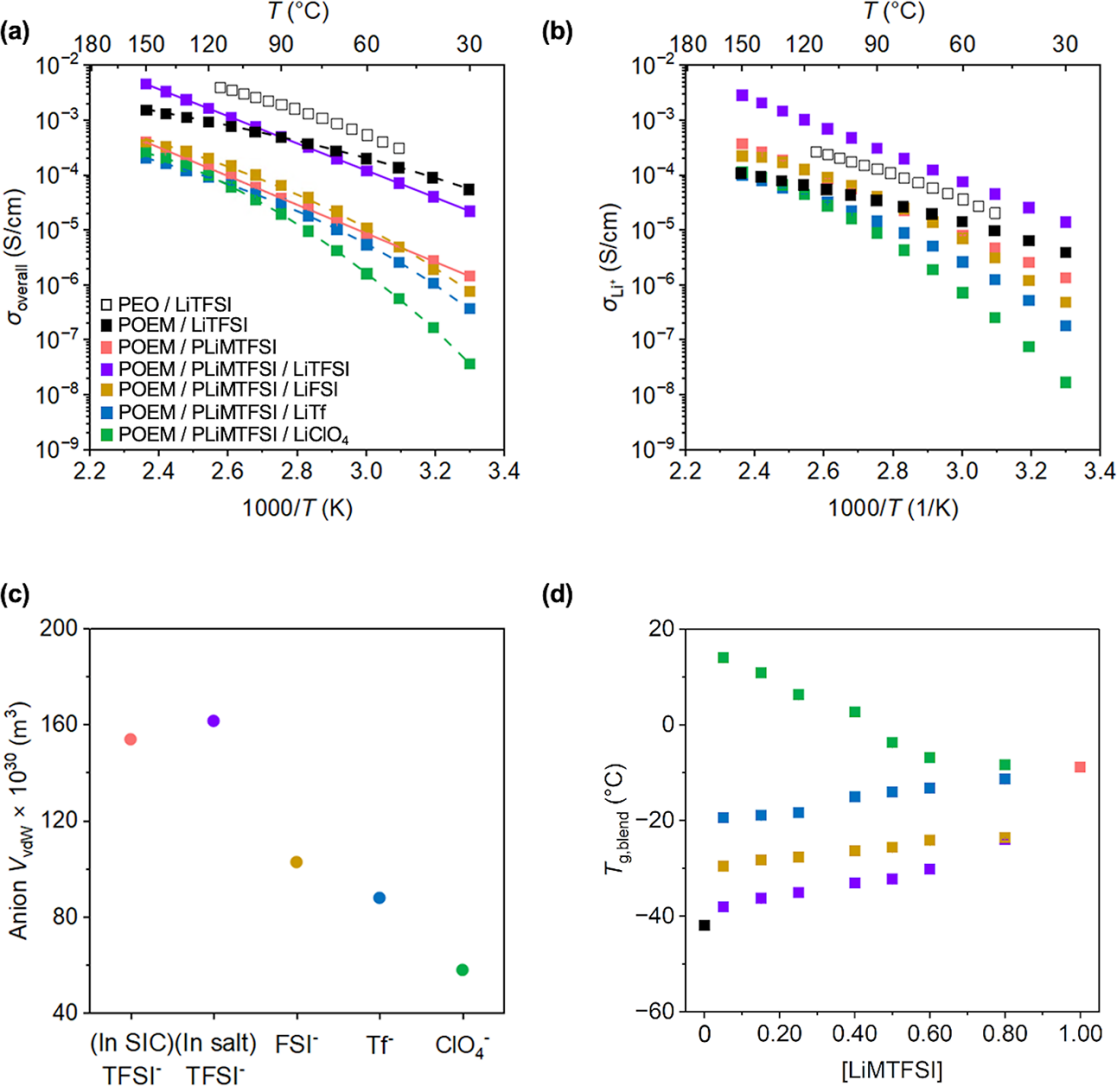
(a) σ_overall_ vs 1000/*T* and (b) σ_Li+_ vs 1000/*T* of POEM/LiTFSI, POEM/PLiMTFSI, and POEM/PLiMTFSI/Li salt blends. POEM/PLiMTFSI σ_overall_ data were obtained from ref 35, PEO/LiTFSI σ_overall_ data were obtained from ref 78, the PEO/LiTFSI *t*_Li+_ value obtained from ref 19 was used for POEM/LiTFSI due to the similar Li^+^ coordination environment and transport behavior. (c) Anion *V*_vdW_ at 25 °C vs anion chemistry. (d) *T*_g,blend_ vs. [LiMTFSI]. VTF-like fits to σ_overall_s are shown by dashed lines; Arrhenius-like fits to σ_overall_s are shown by solid lines. All the mixed-salt blends have a composition of [EO]:[LiMTFSI]:[Li salt] of 10:0.50:0.50. The error bars for σ_overall_s in (a) and σ_Li+_s in (b), all of which are smaller than the data points, represent the standard deviations from the 5 min and 8 min impedance measurements for σ_overall_s and three measurements for *t*_Li+_s. All the error bars for *T*_g,blend_s in (d) are smaller than the data points and represent the standard deviations from three measurements.

At [EO]:[LiMTFSI]:[Li salt] of 10:0.50:0.50, the POEM/PLiMTFSI/LiTFSI blend exhibited an order-of-magnitude higher σ_overall_s (Figure 1a) and σ_Li^+^_s (Figure 1b) in comparison to the tethered-salt POEM/PLiMTFSI.^60^ σ_Li^+^_s were obtained by multiplying σ_overall_s by the *t*Li+s. Notably, the POEM/PLiMTFSI/LiTFSI blend showed higher σ_Li^+^_s in comparison to the most widely studied SPE system – POEM/LiTFSI (Figure 1b). The POEM/PLiMTFSI/LiTFSI maintained the same Arrhenius-like transport behavior. This enhancement likely arose from two factors: easier Li^+^ dissociation in LiTFSI and faster POEM segmental dynamics. First, in LiTFSI, TFSI^–^ contains two trifluoromethyl groups, whereas PLiMTFSI has only one (see Scheme 1). This structural difference increased TFSI^–^ *V*_vdW_ (calculated based on atomic and bond contributions,^79^ see Figure 1c) and weakened its binding affinity to Li^+^,^80^ enhancing Li^+^ dissociation in LiTFSI vs PLiMTFSI. The Li^+^–anion bonding strength for all salts was estimated using Coulomb’s law (see Table S3a), which indicated that larger anion volumes led to weaker Li^+^–anion interactions, making Li^+^ easier to dissociate from the anions. Second, the bulkier TFSI^–^ in LiTFSI led to weaker binding with the −O–CH_2_– group in POEM, in comparison to the smaller, polymer-tethered TFSI^–^ in PLiMTFSI, as predicted by Coulomb’s law (see Table S3b). The interaction between the anion and the −O–CH_2_– group in POEM possibly originates from an anion–dipole interaction arising from the higher electronegativity of oxygen relative to carbon. This electronegativity difference causes electron density to shift toward the oxygen atom, resulting in a negative dipole on oxygen (which coordinates Li^+^)^81^ and a corresponding positive dipole on the carbon atom (which interacts with the anion).^82^ The thermodynamically favored binding motifs (i.e., single-chain or multi-chain coordination) and the specific −O–CH_2_– group(s) in the POEM side chain that dominate the coordination could be explored through future simulation efforts. The electrostatic binding force varied by only ∼2%, yet there was an order-of-magnitude change in σ_overall_s. These results suggested that the electrostatic force difference between the −O–CH_2_– group in POEM and TFSI^–^ might not be the only factor governing the conductivity. It is worth noting that the local solvation environment also influences transport efficiency by creating a mixed Li^+^ coordination environment from both PEO and salt anion in a ternary blend system consisting of a conducting polymer (PEO), a mobile lithium salt, and an ionic liquid;^83,84^ therefore, similar mixed coordination environments may also exist in the present system due to its compositional similarity. The elucidation of the ion-polymer coordination structure can be conducted through simulation studies, such as *via* molecular dynamics,^21,56^ which are the subject of future efforts. Additionally, the anion structural symmetry and electrolyte composition also have been shown to impact Li^+^ solvation structure in salt-doped poly­(ionic liquid) electrolytes^85^ and other systems.^86,87^ Among all the mixed-salt blends, the LiTFSI-based one exhibited the highest σ_overall_s, followed by LiFSI- and LiTf-based blends, while the LiClO_4_-based blend showed the lowest σ_overall_s. This trend suggested that anion dissociation likely played a dominant role in ion transport, with more readily dissociable anions leading to higher σ_overall_s.^88,89^

To probe the impact of composition and mobility (i.e., salt anion size and molar ratio of [EO]:[LiMTFSI]:[Li salt]) on electrolytes’ thermal properties, *T*_g,blend_s of untethered-salt, tethered-salt, and four types of mixed-salt blend electrolytes were examined using DSC (see Figures S8–S13 and Table S2). As shown in Figure 1d, *T*_g,blend_ increased with decreasing anion *V*_vdW_ for the mixed-salt blends, likely due to stronger interaction forces between anions and the polymers (see Table 1), which restricted chain flexibility.^90^ As the LiMTFSI concentration was gradually increased from 0 to 1, all the blends demonstrated higher *T*_g,blend_s, except for those containing LiClO_4_. This contrasting behavior was likely due to the incomplete solvation of LiClO_4_ within the polymer matrix at elevated LiMTFSI loadings. With more LiMTFSI added, the amount of LiClO_4_ was correspondingly reduced, leading to fewer LiMTFSI-POEM-ClO_4_^–^ crosslinks, thereby lowering *T*_g,blend_s.

**Table 1:** Summary of the Fitting Parameters in the VTF Equation

blend electrolyte	σ_0_ (S/cm)	*E*_a_ (K)
POEM/LiTFSI	0.06	955.0
POEM/PLiMTFSI/LiTFSI	0.05	1216.7
POEM/PLiMTFSI/LiFSI	0.14	1301.6
POEM/PLiMTFSI/LiTf	0.03	1041.0
POEM/PLiMTFSI/LiClO_4_	0.13	1278.3

### Blends with Bulkier Anion can Balance Ionic Conductivity and Cation Transport

To demonstrate the impact of composition on ion transport, σ_overall_ at 60 °C was examined as a function of LiMTFSI concentration ([LiMTFSI]) for untethered-salt POEM/LiTFSI, tethered-salt POEM/PLiMTFSI, and mixed-salt POEM/PLiMTFSI/Li salt blends (Figure 2a, see Figure 3 for results at 100 °C). σ_overall_s of the mixed-salt blends exhibited a nonmonotonic trend with LiMTFSI concentration. For mixed-salt blends containing LiTFSI, σ_overall_s initially enhanced with increasing LiMTFSI content, and reached a maximum at [EO]:[LiMTFSI]:[LiTFSI] of 10:0.50:0.50. At this composition, ion transport transitioned from VTF-like to Arrhenius-like behavior. More specifically, at lower [LiMTFSI] (i.e., < 0.50), all mixed-salt systems, regardless of the salt chemistry, displayed VTF-like transport mechanism (see Figures S44–S46). In contrast, at higher [LiMTFSI] (i.e., ≥0.50), Arrhenius behavior was seen in the temperature-dependent σ_overall_s (see Figures 1a, S47, and S48). This transition was likely due to the presence of a higher fraction of PLiMTFSI, which provided more free volume to facilitate ion-hopping motion. A further increase in LiMTFSI concentration led to a decrease in σ_overall_s, likely because the system now comprised more PLiMTFSI, which interacted more strongly with POEM than LiTFSI would (see Table 1). As a result, the segmental relaxation of POEM may be slowed, reducing the rate of solvation site renewal for Li^+^ hopping. For mixed-salt blends consisting of LiFSI and LiTf, both of which displayed VTF-like transport behavior across all tethered-to-untethered salt ratios, the σ_overall_ values were comparable and dropped almost monotonically with increasing LiMTFSI content. This conductivity trend mirrored the *T*_g,blend_ trend (see Figure 4a) in both systems, suggesting a strong correlation between segmental dynamics and ion transport, which is consistent with the VTF-like transport behavior. Additionally, all LiClO_4_-based, mixed-salt blends exhibited VTF-like transport behavior and had the lowest σ_overall_s across almost all tethered-to-untethered salt ratios. The relatively poor conductivities possibly arose from a combination of limited ion dissociation in LiClO_4_ and potentially increasingly torturous transport pathways, with their relative contributions depending on the blend composition. Specifically, in blends with [EO]:[LiMTFSI]:[LiClO_4_] molar ratios of 10:0.05:0.95, 10:0.15:0.85, 10:0.25:0.75, and 10:0.40:0.60, LiClO_4_ appeared to be fully solvated, as indicated by the absence of crystalline LiClO_4_ peaks in the WAXS data (see Figures S37–S40). Despite this complete solvation, these blends exhibited the lowest conductivities among the mixed-salt systems, suggesting that limited ion dissociation of LiClO_4_ (due to its smaller anion size) played a dominant role. In contrast, at higher LiClO_4_ loadings ([EO]:[LiMTFSI]:[LiClO_4_] of 10:0.50:0.50, 10:0.60:0.40, and 10:0.80:0.20), incomplete LiClO_4_ solvation by the polymers was noted, as evidenced by the sharp peak at 47° in the WAXS data (see Figures S41–S43).^65^ This incomplete solvation may lead to more tortuous ion transport pathways, further suppressing conductivity. A detailed computational analysis of transport pathway evolution with composition may provide additional insights.

**Figure 2:**
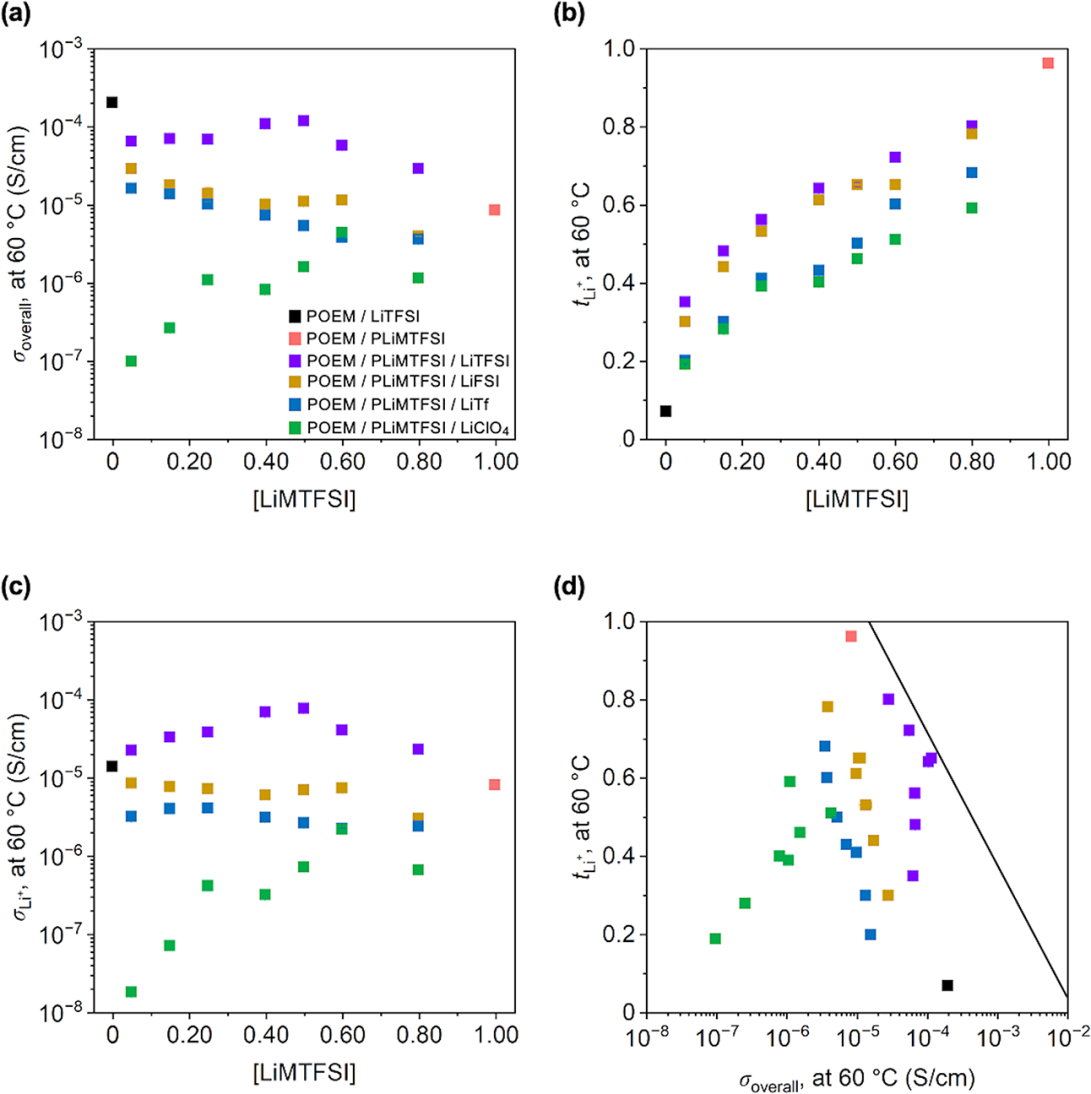
(a) σ_overall_ (at 60 °C) vs [LiMTFSI], (b) *t*_Li+_ vs [LiMTFSI], (c) σ_Li+_ vs [LiMTFSI], and (d) *t*_Li+_ vs σ_overall_ of POEM/LiTFSI, POEM/PLiMTFSI and POEM/PLiMTFSI/Li salt blends. POEM/PLiMTFSI σ_overall_ data were obtained from ref 35, the PEO/LiTFSI *t*_Li+_ value obtained from ref 19 was used for POEM/LiTFSI due to the similar Li^+^ coordination environment and transport behavior. The straight line in (d) is an empirically established upper bound for polymer electrolytes from ref 34. The error bars for σ_overall_s in (a) and (d) and *t*_Li+_s in (b) and (d), all of which are smaller than the data points, represent the standard deviations from the 5 min and 8 min impedance measurements for σ_overall_s and three measurements for *t*_Li+_s.

**Figure 3:**
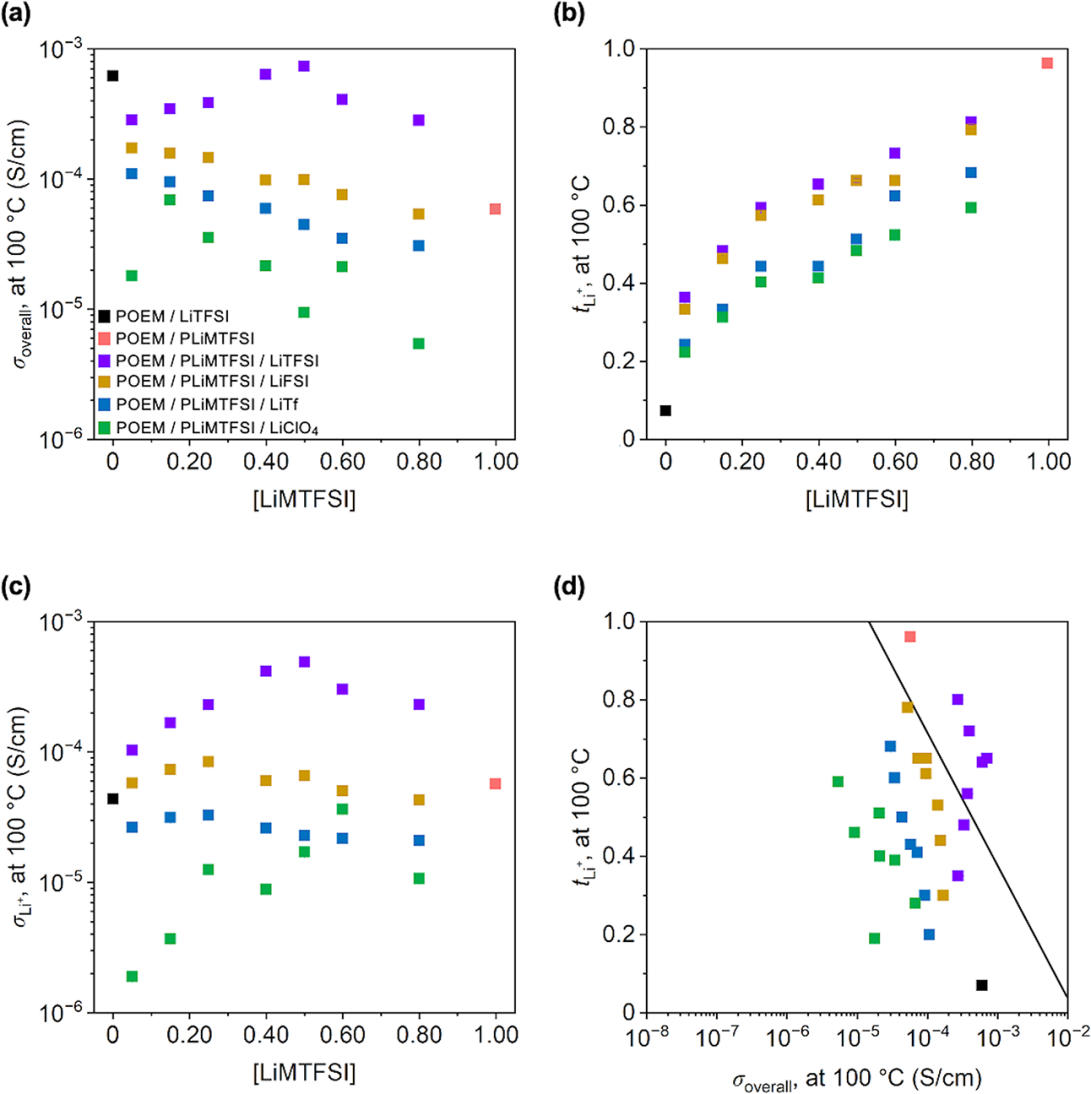
(a) σ_overall_ (at 100 °C) vs [LiMTFSI], (b) *t*_Li+_ vs [LiMTFSI], (c) σ_Li+_ vs [LiMTFSI], and (d) *t*_Li+_ vs σ_overall_ of POEM/LiTFSI, POEM/PLiMTFSI, and POEM/PLiMTFSI/Li salt blends. POEM/PLiMTFSI σ_overall_ data were obtained from ref 35, the PEO/LiTFSI *t*_Li+_ value obtained from ref 19 was used for POEM/LiTFSI due to the similar Li^+^ coordination environment and transport behavior. The straight line in (d) is an empirically established upper bound for polymer electrolytes from ref 34. The error bars for σ_overall_s in (a) and (d) and *t*_Li+_s in (b) and (d), all of which are smaller than the data points, represent the standard deviations from the 5 min and 8 min impedance measurements for σ_overall_s and three measurements for *t*_Li+_s.

**Figure 4:**
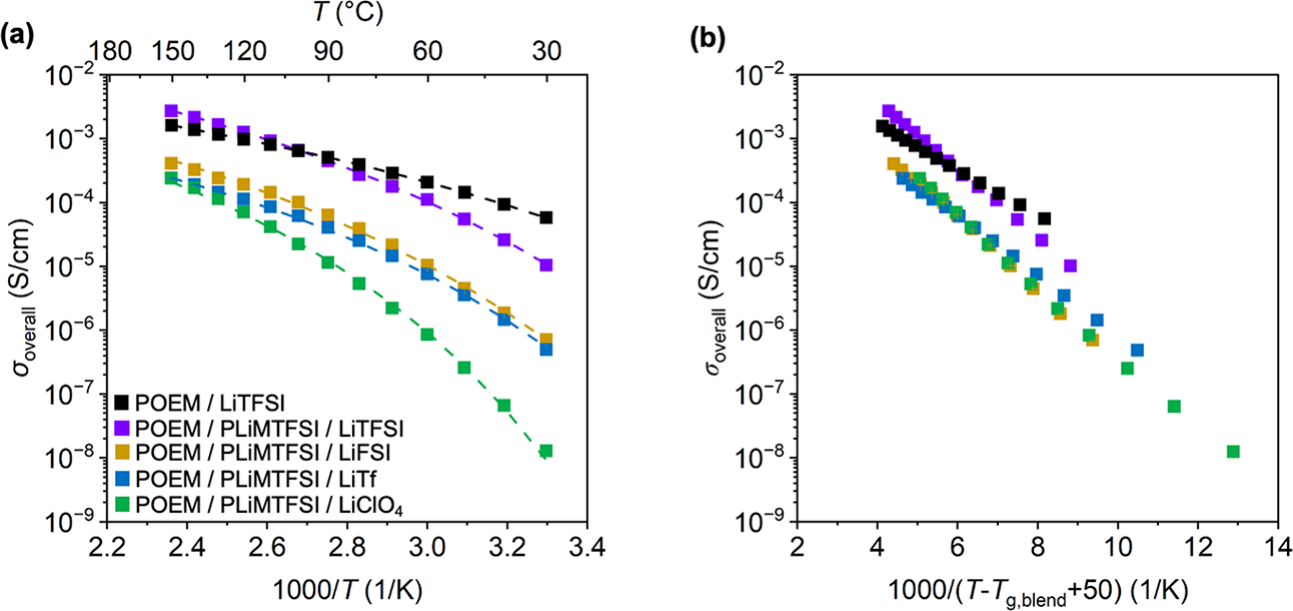
(a) σ_overall_ vs 1000/*T* and (b) σ_overall_ vs 1000/(*T – T*_g,blend_ + 50) of POEM/LiTFSI blend and POEM/PLiMTFSI/Li salt blends. All the mixed-salt blends have a composition of [EO]:[LiMTFSI]:[Li salt] of 10:0.40:0.60. All the error bars for σ_overall_s in (a) and (b) are smaller than the data points and represent the standard deviations from the 5 and 8 min impedance measurements.

The impact of salt anion size on the ion mobility is illustrated in Figure 2b by examining *t*_Li+_ as a function of LiMTFSI concentration. Tethered-salt POEM/PLiMTFSI exhibited a *t*_Li+_ slightly below 1. In comparison to the untethered-salt POEM/LiTFSI system, the mixed-salt blends showed at least a 2-fold enhancement in *t*_Li+_s. All mixed-salt blends demonstrated a monotonic increase in *t*_Li+_ with rising LiMTFSI concentration, as more anions became tethered. Additionally, higher *t*_Li+_s were associated with larger salt anion *V*_vdW_s – a trend consistent with the literature.^43^

To better understand how system composition influences effective conductivity, σ_Li+_ (Figure 2c) at 60 °C was plotted against LiMTFSI concentration.^91^ σ_Li+_ values were obtained by multiplying σ_overall_ by the appropriate *t*_Li+_. The mixed-salt blends displayed a nonmonotonic trend of σ_Li+_ vs LiMTFSI concentration, and σ_Li+_s increased with the anion *V*_vdW_. In comparison to both the untethered-salt POEM/LiTFSI and tethered-salt POEM/PLiMTFSI, LiTFSI-based, mixed-salt blends featured an at least 2-fold enhancement in σ_Li+_s across all concentrations. The intrinsic trade-off between σ_overall_ and *t*_Li+_ can be visualized through an analogy to a Robeson plot (Figure 2d), commonly used to evaluate gas separation membranes.^2,39,92–96^ An empirical upper bound – independent of polymer structure or chemistry – has been established for polymer electrolytes.^2,39^ Among the mixed-salt systems, the LiTFSI-based blends are located closest to this upper limit, with those exhibiting Arrhenius-like transport behavior closer than those showing VTF-like behavior. In contrast, LiFSI-, LiTf-, and LiClO_4_-based blends, all with VTF-like transport, lie progressively farther from the upper bound.

Similar composition-dependent trends were found for σ_overall_s (Figure 3a), *t*_Li+_s (Figure 3b), and σ_Li+_s (Figure 3c) at 100 °C, except for the Robeson-inspired plot (i.e., σ_overall_s vs *t*_Li+_s, see Figure 3d). The *t*_Li+_ values determined at 60 °C were in good agreement with those measured at 100 °C (see Figure 2b, Figure 3b, and Tables S4–S32). The impedance and current responses for *t*_Li+_ measurements conducted at 100 °C are located in Figures S70–S90, S112–S132, S154–S174, S196–S216, and S220–S222. Additionally, as shown in Figure 3d, in the Robeson-inspired plot, LiTFSI-based, mixed-salt systems exceeded the empirically established upper bound at [EO]:[LiMTFSI]:[LiTFSI] molar ratios of 10:0.25:0.75, 10:0.40:0.60, 10:0.50:0.50, 10:0.60:0.40, and 10:0.80:0.20, with blends displaying Arrhenius-like transport behavior (i.e., ≥0.50) being located further above the upper limit. These results demonstrated that both employing a bulky anion (i.e., TFSI^–^) and decoupling ion transport from the slow polymer segmental dynamics (i.e., Arrhenius-like transport mechanism) were key factors to enhance ion conduction.

To examine the role of *T*_g,blend_ in ion transport, the σ_overall_s of the four types of mixed-salt blends were compared with the untethered-salt POEM/LiTFSI (Figure 4b,c). All systems had a molar ratio of [EO]:[LiMTFSI]:[Li salt] of 10:0.40:0.60 and demonstrated VTF-like transport behavior. This molar ratio was chosen due to all the systems displaying VTF-like transport, in which *T*_g_ was the governing factor.^12^ The fitting parameters from the VTF equation (eq 3) are summarized in Table 1. POEM/LiTFSI and the POEM/PLiMTFSI/LiTFSI blends had similar charger carrier concentrations (σ_0_s) but differed in activation energies associated with transport (*E*_a_s), while the POEM/PLiMTFSI/LiFSI and POEM/PLiMTFSI/LiClO_4_ systems exhibited increased σ_0_s but with higher *E*_a_s and lower σ_overall_s. This relationship among σ_0_, *E*_a_, and anion *V*_vdW_ indicated that the anion size, which directly influenced the strength of Li^+^-anion interactions,^97^ was not the sole parameter governing ion transport properties. Additionally, σ_overall_ was plotted as a function of 1000/(*T – T*_g,blend_ + 50), as shown in Figure 4c. Although the slopes of the mixed-salt blends were similar, their intercepts varied (i.e., the data do not collapse into a single curve when altering the salt chemistry), suggesting that *T*_g,blend_ alone did not account for the σ_overall_ changes.^91,98^ These results suggested that ion transport in POEM-based electrolytes was governed cooperatively by multiple factors, including anion size, polymer segmental dynamics, and potential variations in the local solvation environment. Bulkier anions dissociate more easily and simultaneously promote faster polymer segmental dynamics by exerting weaker electrostatic interactions with the polymer matrix, as suggested by Figure 1d. Faster segmental motion facilitates the rearrangement of polymer chains to form favorable ion coordination environments, enabling ions to hop more efficiently between coordination sites, thereby leading to higher ionic conductivities. Taken together, these insights provided a clear design framework for optimizing polymer electrolytes, highlighting the importance of tuning both anion properties and polymer dynamics to achieve enhanced ion transport.

## Conclusions

This work demonstrates that leveraging anion composition and mobility in SPEs can modulate ionic conductivity and selective cation transport. The anion composition and mobility were adjusted to induce a transition in the Li^+^ transport mechanism from coupled-to-polymer segmental motion (VTF-like) to a decoupled, hopping-like mechanism (Arrhenius-like). Specifically, a polymer blend containing a bulky salt anion (TFSI^–^) and an equimolar mixture of PLiMTFSI and LiTFSI demonstrated Li^+^ conductivities an order of magnitude higher than those of POEM/LiTFSI −4.8 × 10^–4^ S/cm in the ternary blend vs 6.3 × 10^–5^ S/cm in POEM/LiTFSI at 100 °C. This enhancement was attributed to a more than 9-fold increase in *t*_Li+_ (0.66 in the ternary blend vs 0.07 in POEM/LiTFSI at 100 °C). Moreover, increasing anion volumes led to simultaneous enhancements in σ_overall_s, *t*_Li+_s, and σ_Li+_s, highlighting the effectiveness of leveraging bulky, delocalized anions to decouple ion transport and improve overall cation conductivities.

## Supplementary Material



## Notes

Neat PLiMTFSI and neat POEM characterization via ^1^H NMR spectroscopy and SEC. TGA, DSC, SAXS, and WAXS characterization for neat polymers and/or blends; ionic conductivity vs 1000/*T*, impedance response before and after polarization, and current response before and after polarization for salt-containing blends (PDF)
