# Supplementary material for: Tuning Anion Composition and Mobility to Balance Ionic Conductivity and Cation Selectivity in Solid Polymer Electrolytes
Source: Macromolecules. 2026 Apr 14;59(8):5005–16. doi: 10.1021/acs.macromol.5c03173 (PMC13131046; doi:10.1021/acs.macromol.5c03173)
Supplement: Supplementary file 1 [file ma5c03173_si_001.pdf]

Supporting Information for:

Tuning Anion Composition and Mobility to Balance Ionic Conductivity and Cation Selectivity in Solid Polymer Electrolytes

Mengying Yang,[a] Thomas H. Epps, III[a,b,c,*]

[a]Department of Materials Science & Engineering, University of Delaware, Newark, DE 19716, United States

[b]Department of Chemical & Biomolecular Engineering, University of Delaware, Newark, DE 19716, United States

[c]Center for Research in Soft matter & Polymers (CRiSP), University of Delaware, Newark, DE 19716, United States

*Email: thepps@udel.edu

Contents

1. Proton Nuclear Magnetic Resonance (^{1}H NMR) Spectroscopy 3
2. Size Exclusion Chromatography (SEC) 5
3. Differential Scanning Calorimetry (DSC)...6
4. Thermogravimetric Analysis (TGA) 13
5. Small-Angle X-ray Scattering (SAXS) and Wide-Angle X-ray Scattering (WAXS) 17

6. Alternating Current (AC) Impedance Spectroscopy 47

7. Electrostatic Force (F_e) 52

8. Potentiostatic Polarization 54

9. References 191

1. Proton Nuclear Magnetic Resonance (^{1}H NMR) spectroscopy

1.1 ^{1}H NMR spectrum of poly(*oligo*-oxyethylene methyl ether methacrylate) (POEM)

^{1}H NMR spectroscopy experiments were run on a Bruker AV600III instrument using chloroform-d [0.03% v/v tetramethylsilane (TMS)] as the solvent. ^{1}H NMR (chloroform-d, 600 MHz, δ) for POEM: 4.08 (2H, t), 3.65 (32H, t), 3.38 (3H, t).

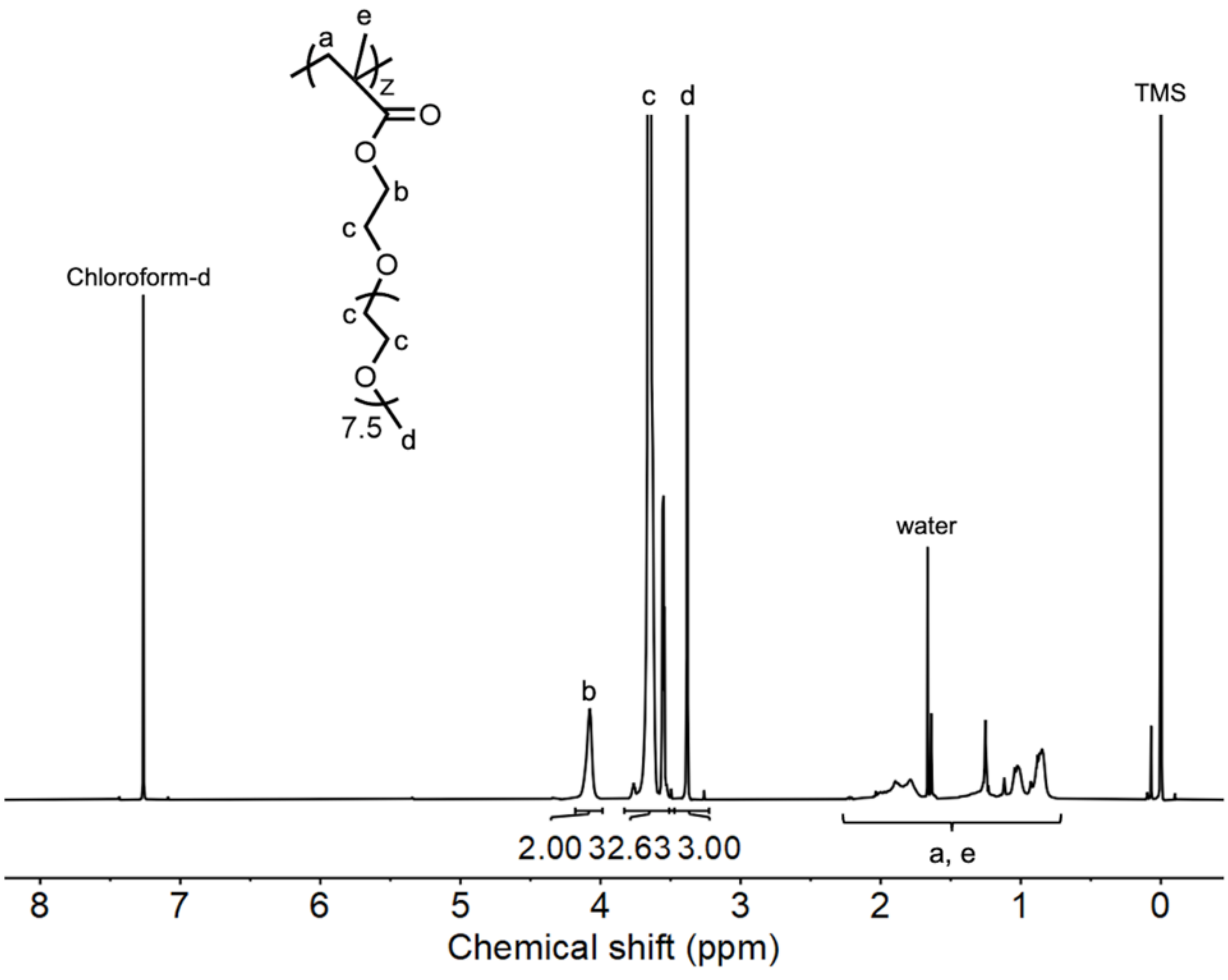

Figure S1. ^{1}H NMR spectrum of POEM.

1.2 ^{1}H NMR spectrum of poly(lithium sulfonyl(trifluoromethane sulfonyl)imide methacrylate) (PLiMTFSI)

^{1}H NMR spectroscopy experiments were run on a Bruker AV600III instrument using acetone-d_6 (0.03% v/v TMS) as the solvent. ^{1}H NMR (acetone-d_6, 600 MHz, δ) for PLiMTFSI: 4.18 (2H, t), 3.27 (2H, qt), 2.22 (2H, t).

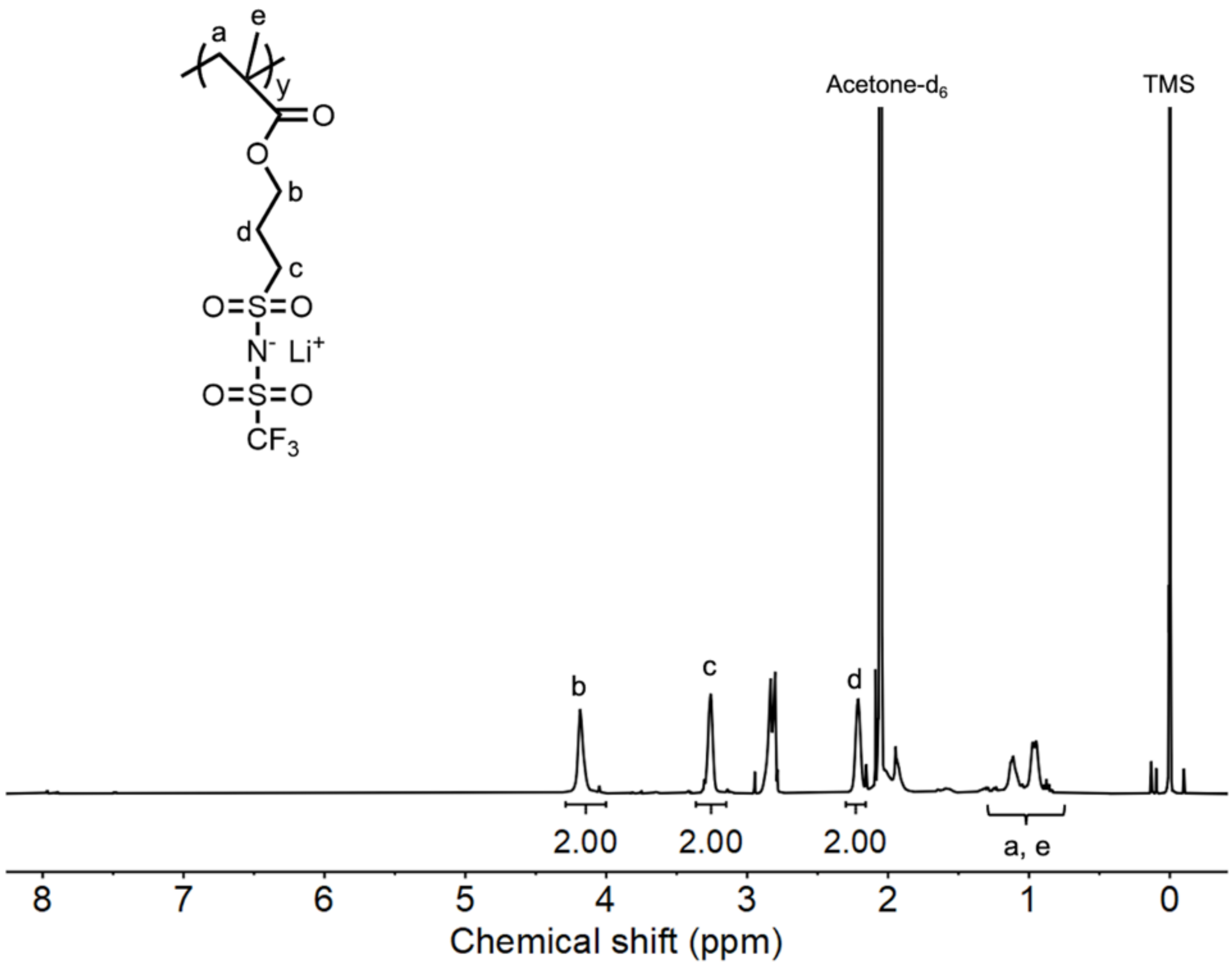

Figure S2. ^{1}H NMR spectrum of PLiMTFSI.

2. Size Exclusion Chromatography (SEC)

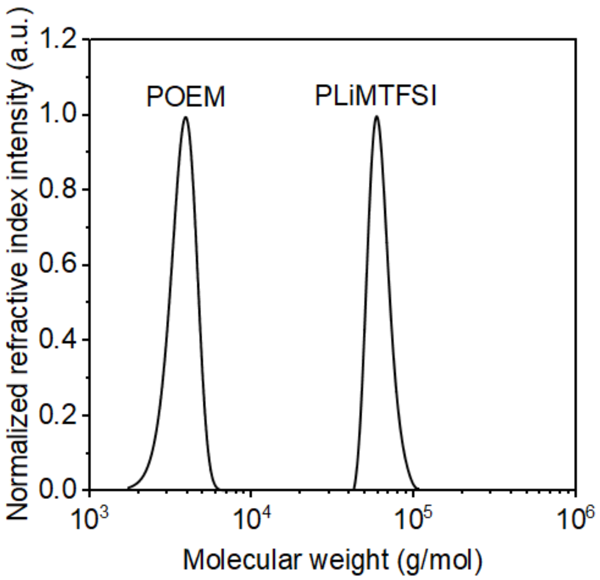

Figure S3. SEC traces for POEM and PLiMTFSI homopolymers.

Table S1. SEC characterization data for POEM and PLiMTFSI homopolymers.

Polymer	M_n[a] (kg/mol)	M_w[b] (kg/mol)	$Đ$[c]
POEM	5.9	7.5	1.3
PLiMTFSI	105.7	141.6	1.3

[a]Number-average molecular weight as determined by SEC. [b]Weight-average molecular weight as determined by SEC. [c]Dispersity as determined by SEC.

3. Differential Scanning Calorimetry (DSC)

The overall Li^+ concentration was kept constant in all samples at [EO]:[Li^+] of 10:1, which represents the molar ratio of ethylene oxide monomer segments in POEM side chains to Li^+. In the mixed-salt POEM / PLiMTFSI / Li salt samples, the molar ratios of [EO]:[LiMTFSI]:[Li salt] were either 10:0.05:0.95, 10:0.15:0.85, 10:0.25:0.75, 10:0.40:0.60, 10:0.50:0.50, 10:0.60:0.40, or 10:0.80:0.20. Li salt was chosen as lithium bis(trifluoromethanesulfonyl)imide (LiTFSI), lithium bis(fluorosulfonyl)imide (LiFSI), lithium trifluoromethanesulfonate (LiTf), or lithium perchlorate ($LiClO_4$).

3.1 POEM / LiTFSI blend

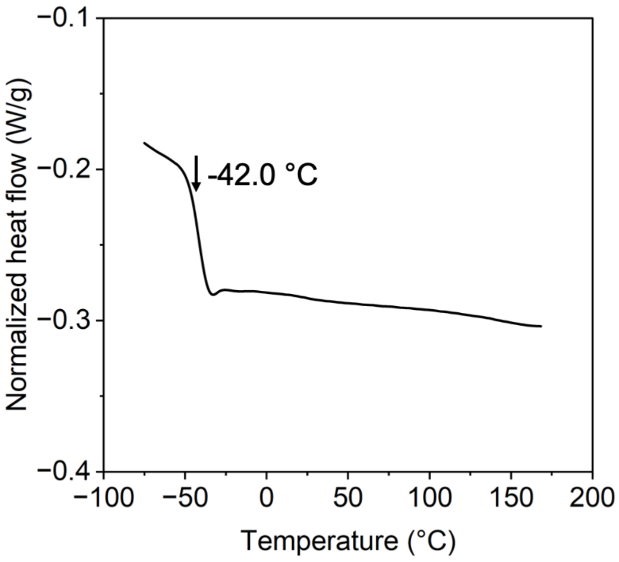

Figure S4. DSC trace [second heating, exotherm up, 10 °C/min ramp rate, nitrogen (N_2)] for POEM / LiTFSI blend, normalized by the total sample mass.

3.2 POEM / PLiMTFSI blend

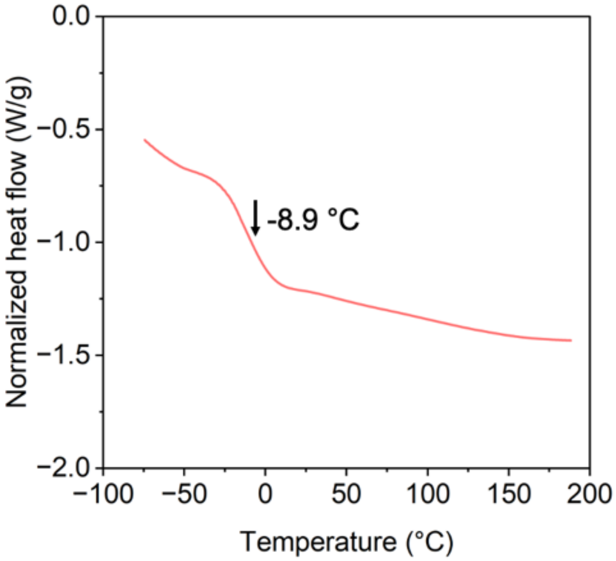

Figure S5. DSC trace (second heating, exotherm up, 10 °C/min ramp rate, N_2) for POEM / PLiMTFSI blend, normalized by the total sample mass.

3.3 POEM / PLiMTFSI / LiTFSI blends

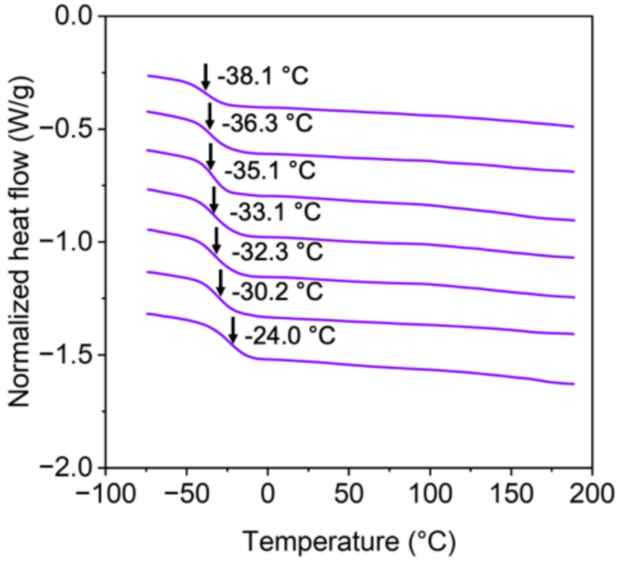

Figure S6. DSC traces (second heating, exotherm up, 10 °C/min ramp rate, N_2) for POEM / PLiMTFSI / LiTFSI blends [top to bottom: [EO]:[LiMTFSI]:[LiTFSI] = 10:0.05:0.95, 10:0.15:0.85, 10:0.25:0.75, 10:0.40:0.60, 10:0.50:0.50, 10:0.60:0.40, 10:0.80:0.20 (molar ratio)], normalized by the total sample mass. Curves are shifted vertically for clarity.

3.4 POEM / PLiMTFSI / LiFSI blends

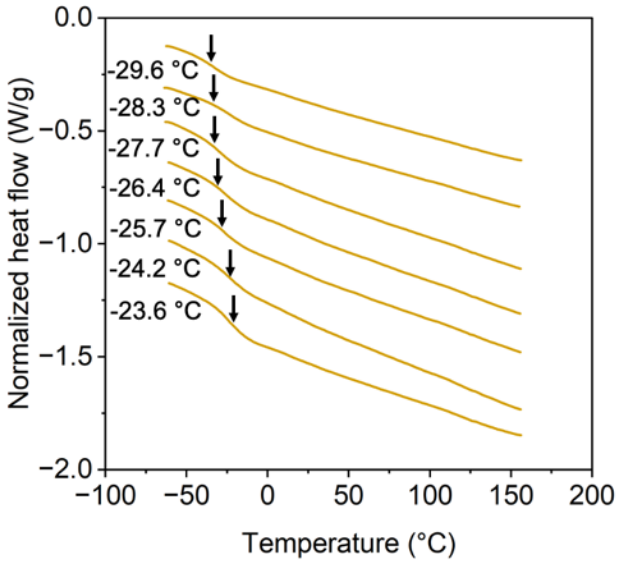

Figure S7. DSC traces (second heating, exotherm up, 10 °C/min ramp rate, N_2) for POEM / PLiMTFSI / LiFSI blends [top to bottom: [EO]:[LiMTFSI]:[LiFSI] = 10:0.05:0.95, 10:0.15:0.85, 10:0.25:0.75, 10:0.40:0.60, 10:0.50:0.50, 10:0.60:0.40, 10:0.80:0.20 (molar ratio)], normalized by the total sample mass. Curves are shifted vertically for clarity.

3.5 POEM / PLiMTFSI / LiTf blends

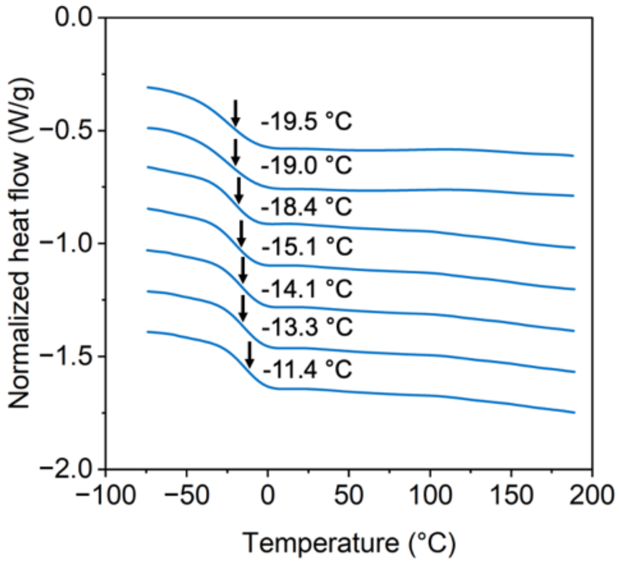

Figure S8. DSC traces (second heating, exotherm up, 10 °C/min ramp rate, N_2) for POEM / PLiMTFSI / LiTf blends [top to bottom: [EO]:[LiMTFSI]:[LiTf] = 10:0.05:0.95, 10:0.15:0.85, 10:0.25:0.75, 10:0.40:0.60, 10:0.50:0.50, 10:0.60:0.40, 10:0.80:0.20 (molar ratio)], normalized by the total sample mass. Curves are shifted vertically for clarity.

3.6 POEM / PLiMTFSI / $LiClO_4$ blends

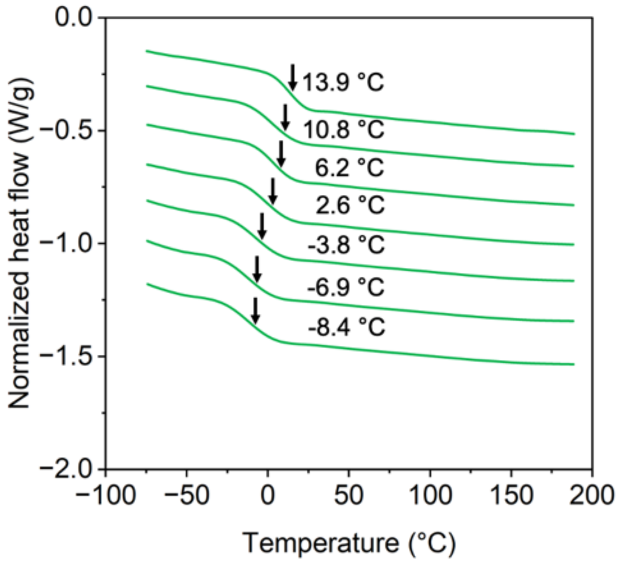

Figure S9. DSC traces (second heating, exotherm up, 10 °C/min ramp rate, N_2) for POEM / PLiMTFSI / $LiClO_4$ blends [top to bottom: [EO]:[LiMTFSI]:[$LiClO_4$] = 10:0.05:0.95, 10:0.15:0.85, 10:0.25:0.75, 10:0.40:0.60, 10:0.50:0.50, 10:0.60:0.40, 10:0.80:0.20 (molar ratio)], normalized by the total sample mass. Curves are shifted vertically for clarity.

Table S2. T_g of POEM / LiTFSI, POEM / PLiMTFSI, and POEM / PLiMTFSI / Li salt blends.

Sample	[EO]:[LiMTFSI]:[Li salt] (molar ratio)	[EO]:[Li^+] (molar ratio)	T_g^{ab} (°C)
POEM / LiTFSI blend	N/A	10:1	-42.0 ± 0.1
POEM / PLiMTFSI blend	N/A		-8.9 ± 0.5
POEM / PLiMTFSI / LiTFSI blends	10:0.05:0.95		-38.1 ± 0.1
	10:0.15:0.85		-36.3 ± 0.2
	10:0.25:0.75		-35.1 ± 0.1
	10:0.40:0.60		-33.1 ± 0.1
	10:0.50:0.50		-32.3 ± 0.1
	10:0.60:0.40		-30.2 ± 0.1
	10:0.80:0.20		-24.0 ± 0.5
POEM / PLiMTFSI / LiFSI blends	10:0.05:0.95		-29.6 ± 0.1
	10:0.15:0.85		-28.3 ± 0.2
	10:0.25:0.75		-27.7 ± 0.4
	10:0.40:0.60		-26.4 ± 0.3
	10:0.50:0.50		-25.7 ± 0.2
	10:0.60:0.40		-24.2 ± 0.4
	10:0.80:0.20		-23.6 ± 0.3
POEM / PLiMTFSI / LiTf blends	10:0.05:0.95		-19.5 ± 0.1
	10:0.15:0.85		-19.0 ± 0.4
	10:0.25:0.75		-18.4 ± 0.2
	10:0.40:0.60		-15.1 ± 0.3
	10:0.50:0.50		-14.1 ± 0.1
	10:0.60:0.40		-13.3 ± 0.1
	10:0.80:0.20		-11.4 ± 0.2
POEM / PLiMTFSI / $LiClO_4$ blends	10:0.05:0.95		13.9 ± 0.3
	10:0.15:0.85		10.8 ± 0.4
	10:0.25:0.75		6.2 ± 0.5
	10:0.40:0.60		2.6 ± 0.5
	10:0.50:0.50		-3.8 ± 0.3
	10:0.60:0.40		-6.9 ± 0.1
	10:0.80:0.20		-8.4 ± 0.2

[a]The reported values are the average of three repeats with errors representing the standard deviation. [b]Determined from the second heating trace.

4. Thermogravimetric Analysis (TGA)

4.1 POEM / PLiMTFSI / LiTFSI blend

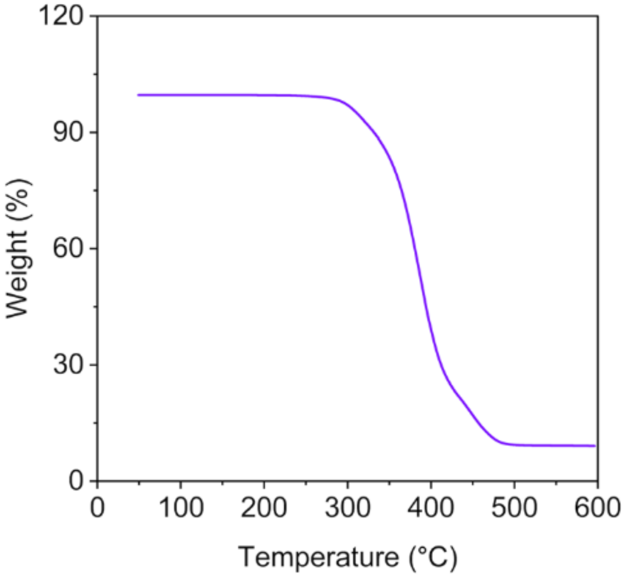

Figure S10. TGA trace for POEM / PLiMTFSI / LiTFSI blend; [EO]:[LiMTFSI]:[LiTFSI] = 10:0.50:0.50 (molar ratio).

4.2 POEM / PLiMTFSI / LiFSI blend

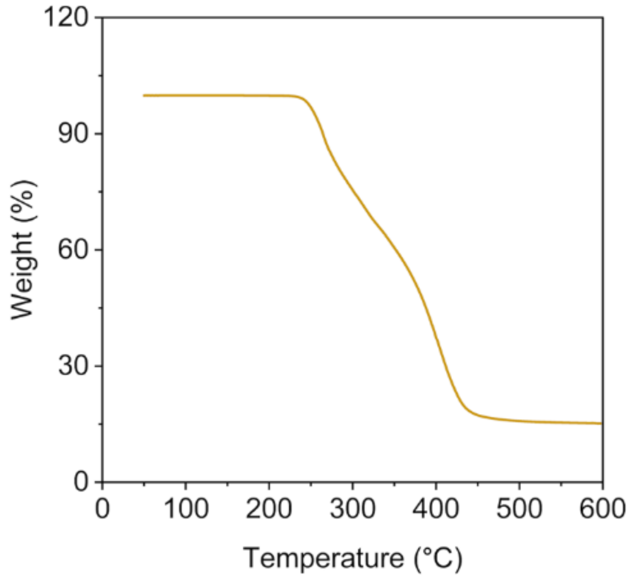

Figure S11. TGA trace for POEM / PLiMTFSI / LiFSI blend; [EO]:[LiMTFSI]:[LiFSI] = 10:0.50:0.50 (molar ratio).

4.3 POEM / PLiMTFSI / LiTf blend

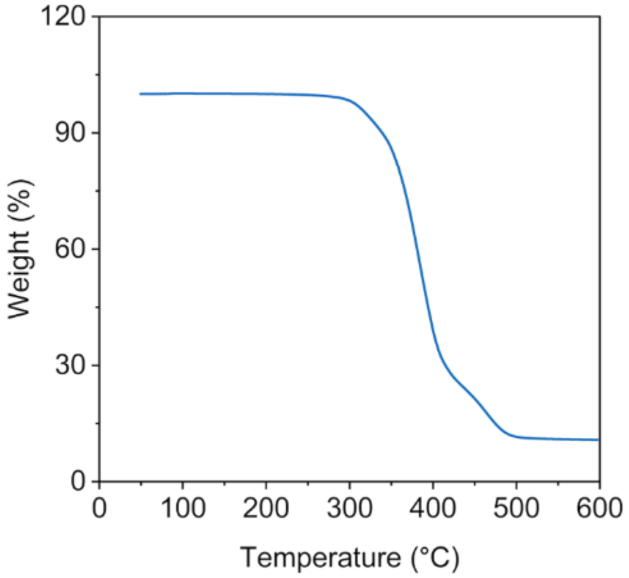

Figure S12. TGA trace for POEM / PLiMTFSI / LiTf blend; [EO]:[LiMTFSI]:[LiTf] = 10:0.50:0.50 (molar ratio).

4.4 POEM / PLiMTFSI / $LiClO_4$ blend

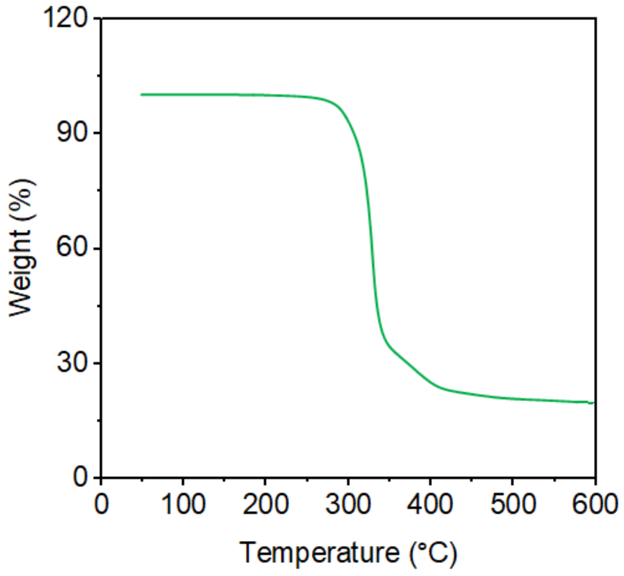

Figure S13. TGA trace for POEM / PLiMTFSI / $LiClO_4$ blend; [EO]:[LiMTFSI]:[$LiClO_4$] = 10:0.50:0.50 (molar ratio).

5. Small-Angle X-ray Scattering (SAXS) and Wide-Angle X-ray Scattering (WAXS)

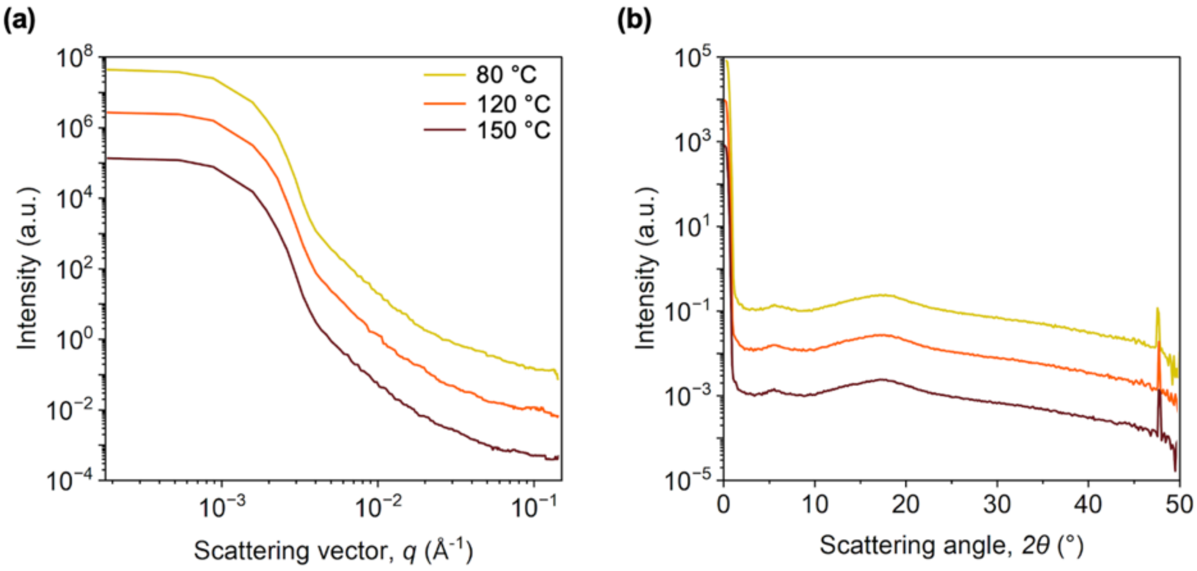

Figure S14. (a) SAXS and (b) WAXS patterns for PLiMTFSI collected upon heating. All patterns are plotted after subtracting the scattering contribution from the Kapton film in the sample chamber. Curves are shifted vertically for clarity.

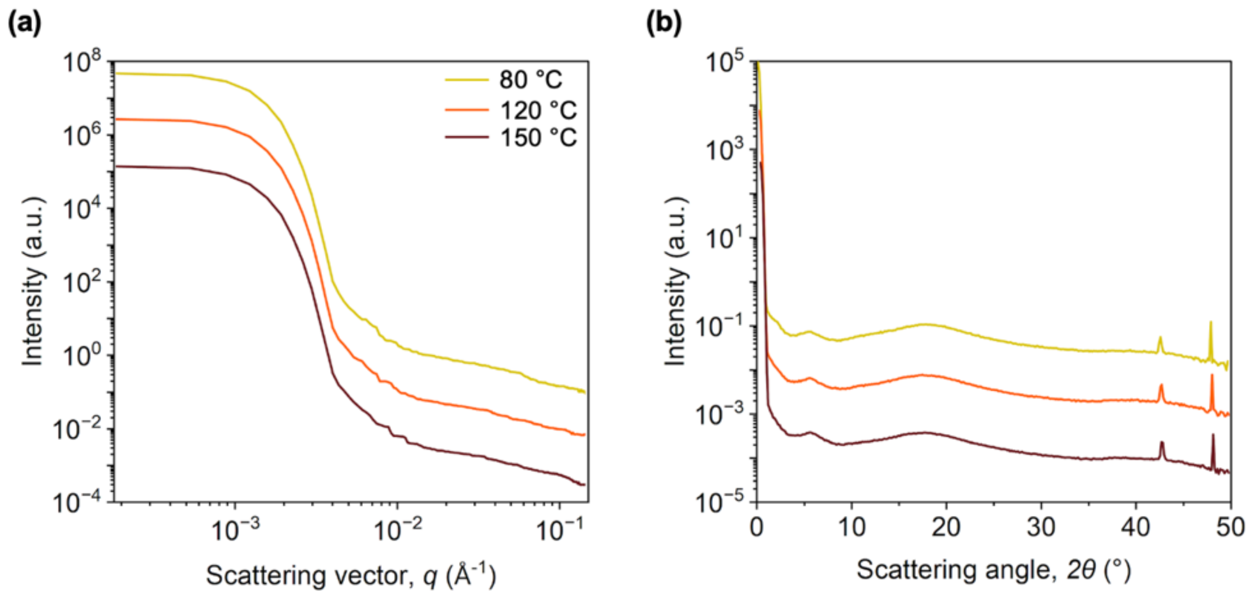

Figure S15. (a) SAXS and (b) WAXS patterns for POEM / PLiMTFSI blend collected upon heating. All patterns are plotted after subtracting the scattering contribution from the Kapton film in the sample chamber. Curves are shifted vertically for clarity.

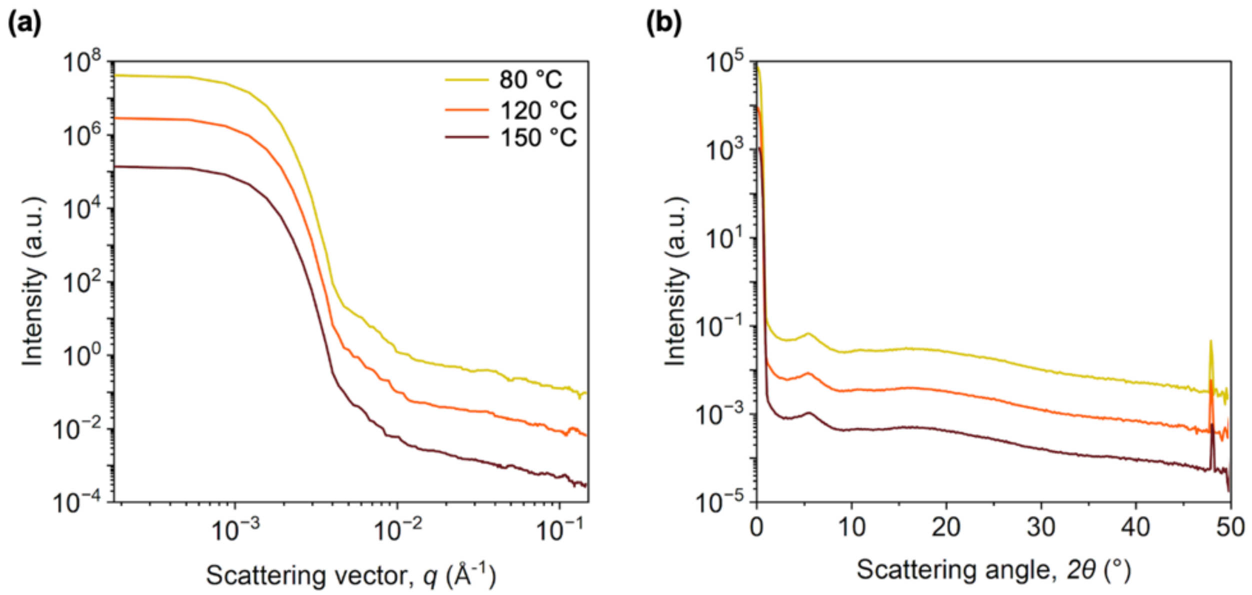

Figure S16. (a) SAXS and (b) WAXS patterns for POEM / PLiMTFSI / LiTFSI blend collected upon heating. All patterns are plotted after subtracting the scattering contribution from the Kapton film in the sample chamber; [EO]:[LiMTFSI]:[LiTFSI] = 10:0.05:0.95 (molar ratio). Curves are shifted vertically for clarity.

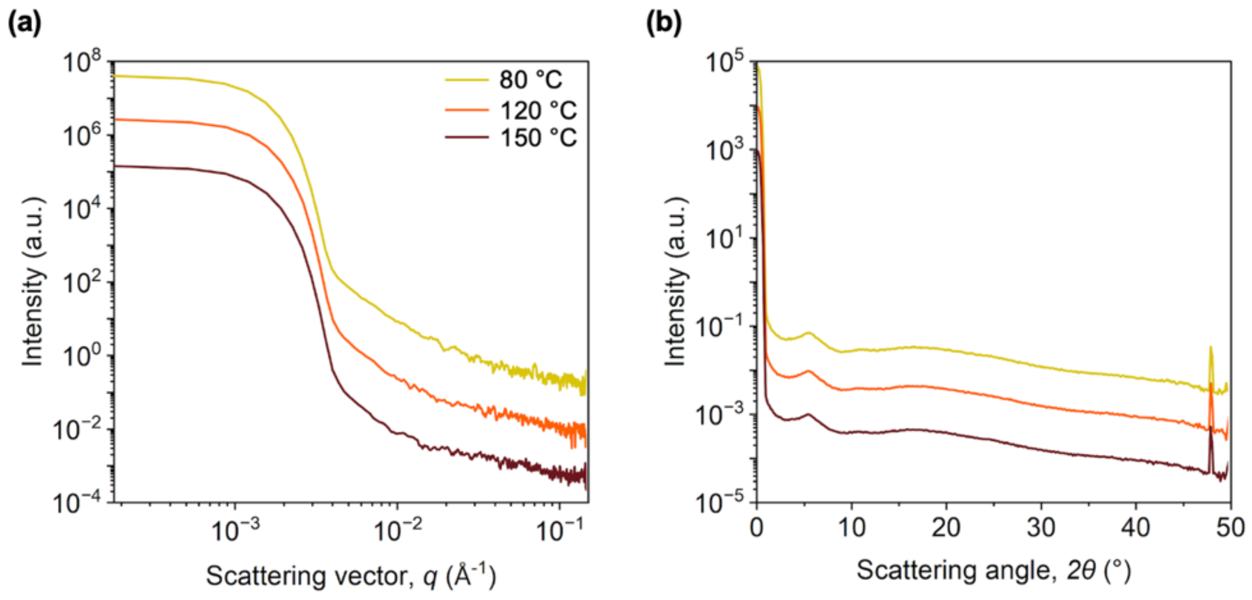

Figure S17. (a) SAXS and (b) WAXS patterns for POEM / PLiMTFSI / LiTFSI blend collected upon heating. All patterns are plotted after subtracting the scattering contribution from the Kapton film in the sample chamber; [EO]:[LiMTFSI]:[LiTFSI] = 10:0.15:0.85 (molar ratio). Curves are shifted vertically for clarity.

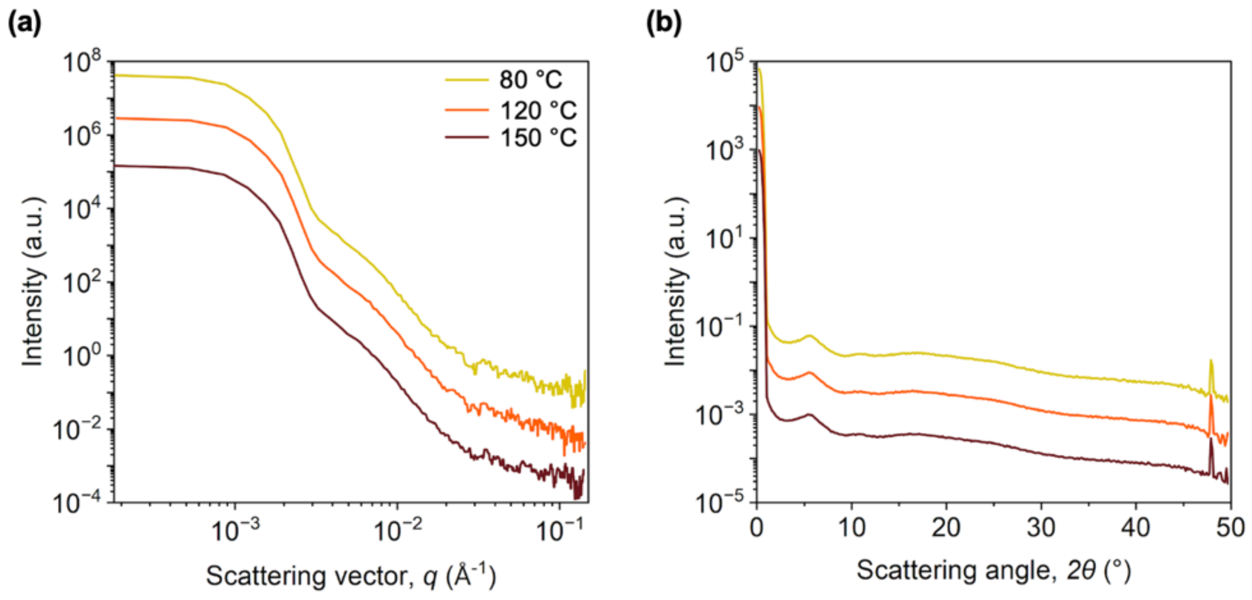

Figure S18. (a) SAXS and (b) WAXS patterns for POEM / PLiMTFSI / LiTFSI blend collected upon heating. All patterns are plotted after subtracting the scattering contribution from the Kapton film in the sample chamber; [EO]:[LiMTFSI]:[LiTFSI] = 10:0.25:0.75 (molar ratio). Curves are shifted vertically for clarity.

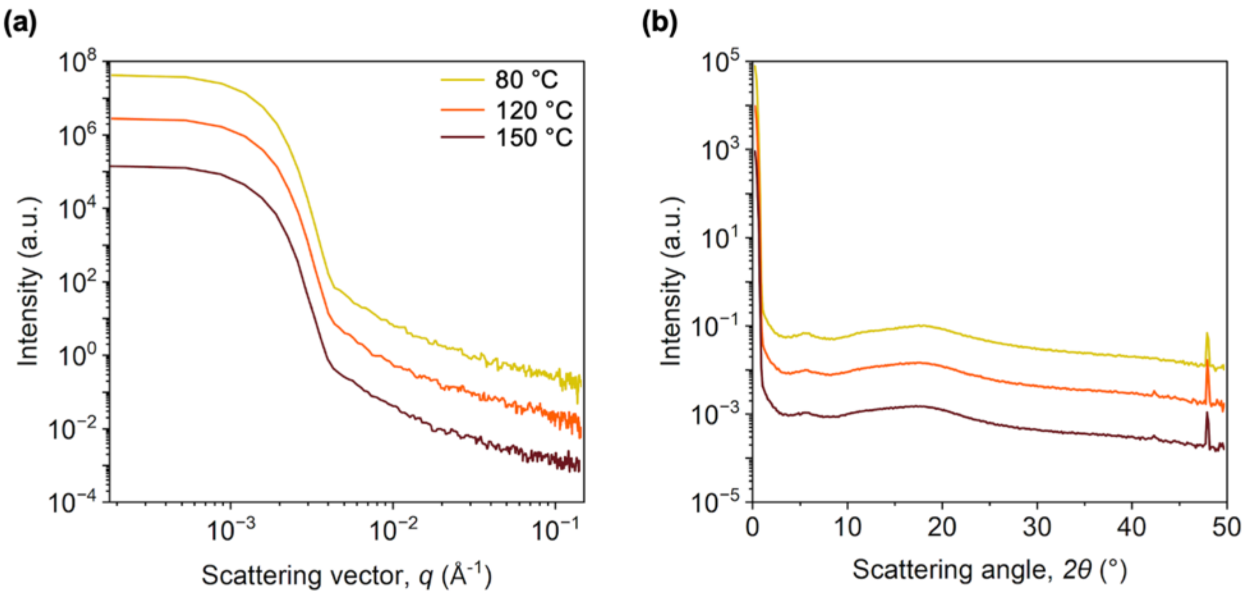

Figure S19. (a) SAXS and (b) WAXS patterns for POEM / PLiMTFSI / LiTFSI blend collected upon heating. All patterns are plotted after subtracting the scattering contribution from the Kapton film in the sample chamber; [EO]:[LiMTFSI]:[LiTFSI] = 10:0.40:0.60 (molar ratio). Curves are shifted vertically for clarity.

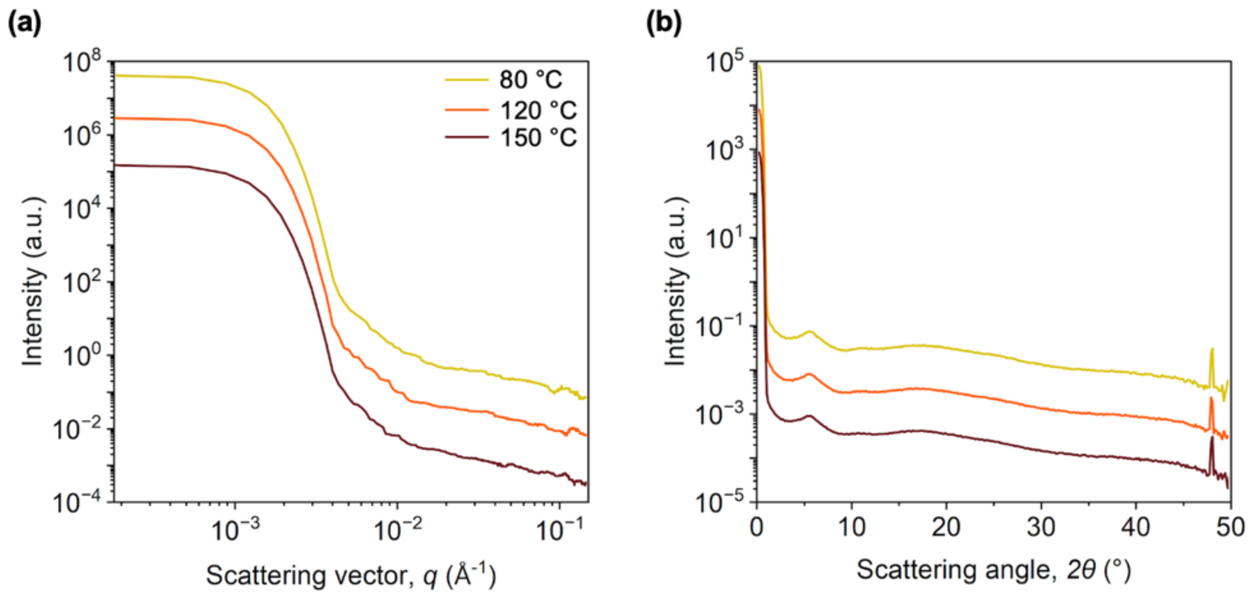

Figure S20. (a) SAXS and (b) WAXS patterns for POEM / PLiMTFSI / LiTFSI blend collected upon heating. All patterns are plotted after subtracting the scattering contribution from the Kapton film in the sample chamber; [EO]:[LiMTFSI]:[LiTFSI] = 10:0.50:0.50 (molar ratio). Curves are shifted vertically for clarity.

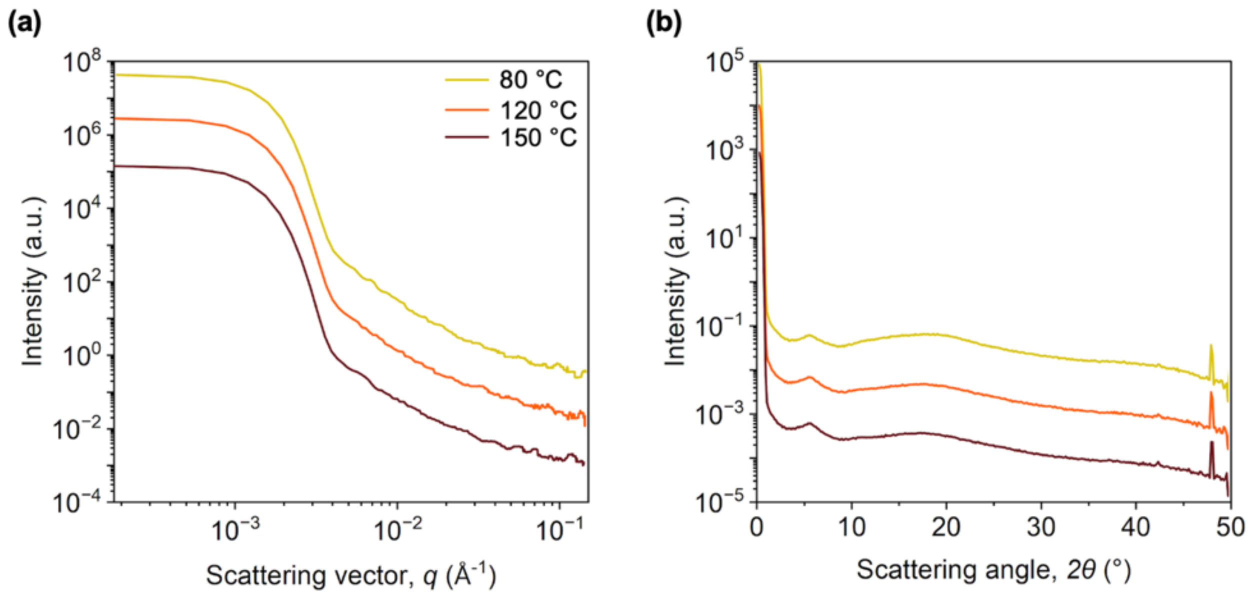

Figure S21. (a) SAXS and (b) WAXS patterns for POEM / PLiMTFSI / LiTFSI blend collected upon heating. All patterns are plotted after subtracting the scattering contribution from the Kapton film in the sample chamber; [EO]:[LiMTFSI]:[LiTFSI] = 10:0.60:0.40 (molar ratio). Curves are shifted vertically for clarity.

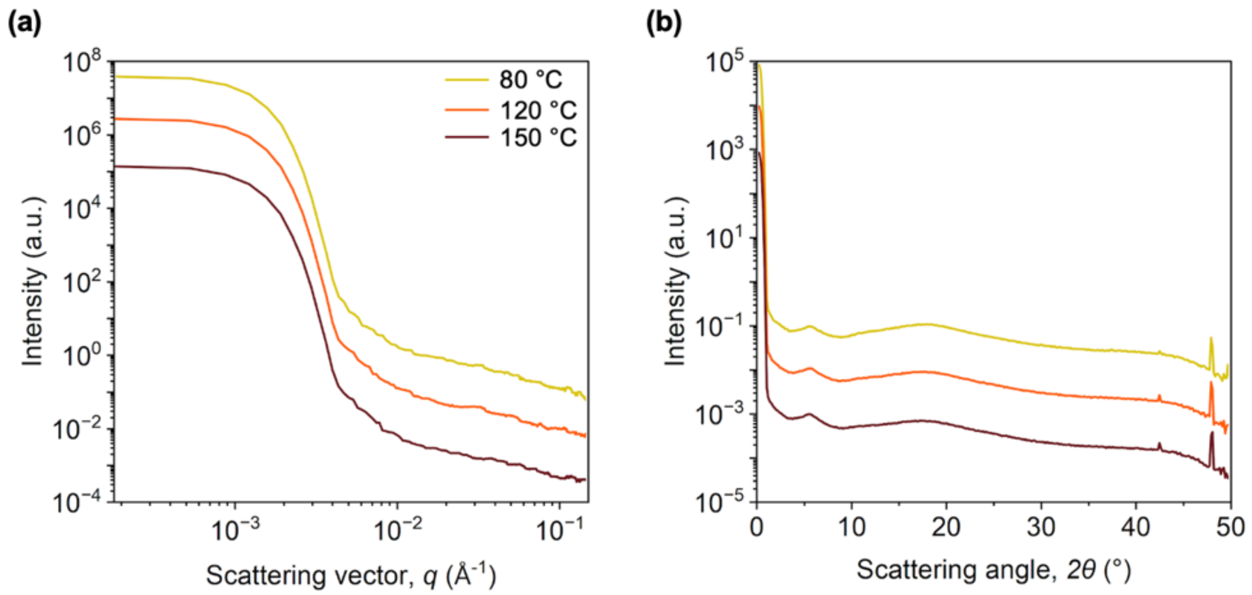

Figure S22. (a) SAXS and (b) WAXS patterns for POEM / PLiMTFSI / LiTFSI blend collected upon heating. All patterns are plotted after subtracting the scattering contribution from the Kapton film in the sample chamber; [EO]:[LiMTFSI]:[LiTFSI] = 10:0.80:0.20 (molar ratio). Curves are shifted vertically for clarity.

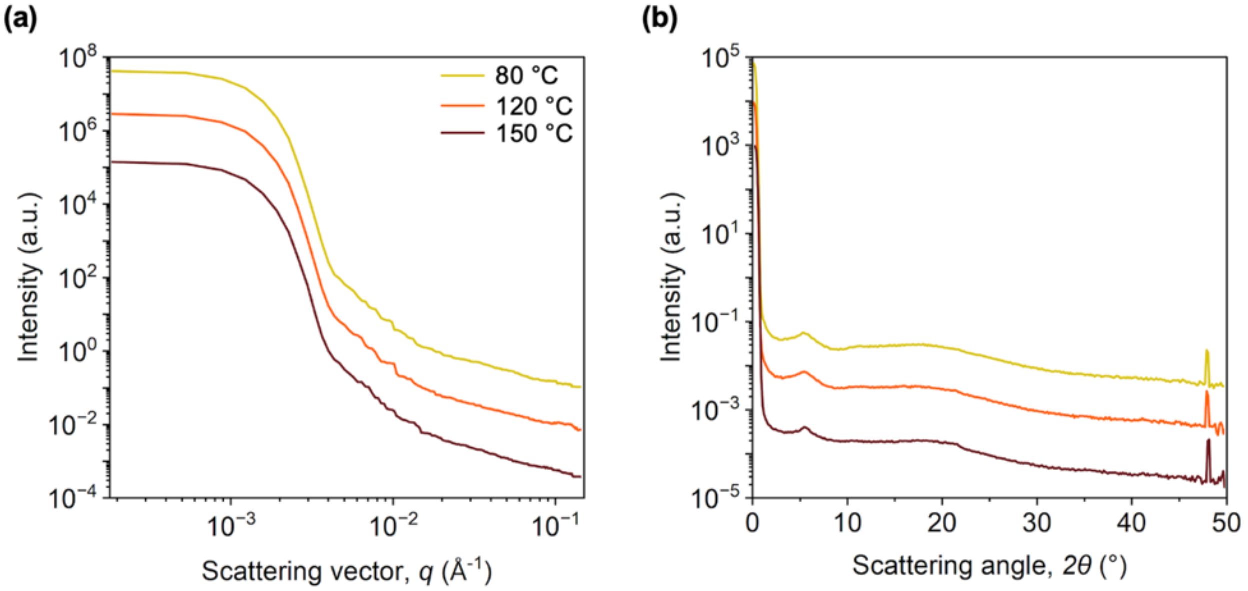

Figure S23. (a) SAXS and (b) WAXS patterns for POEM / PLiMTFSI / LiFSI blend collected upon heating. All patterns are plotted after subtracting the scattering contribution from the Kapton film in the sample chamber; [EO]:[LiMTFSI]:[LiFSI] = 10:0.05:0.95 (molar ratio). Curves are shifted vertically for clarity.

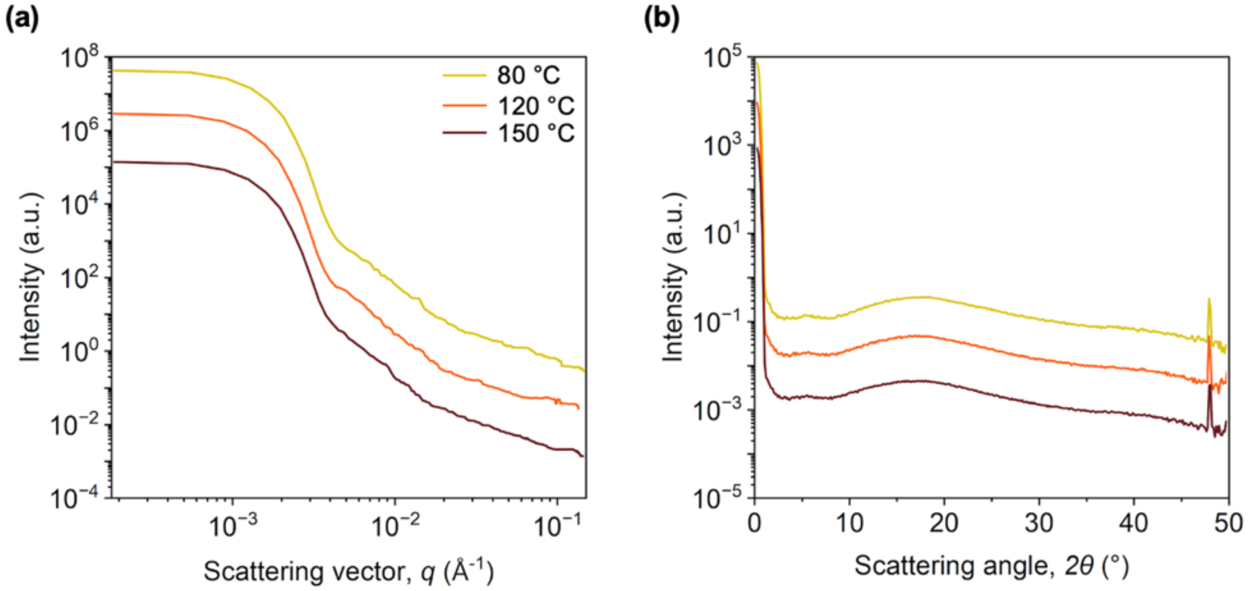

Figure S24. (a) SAXS and (b) WAXS patterns for POEM / PLiMTFSI / LiFSI blend collected upon heating. All patterns are plotted after subtracting the scattering contribution from the Kapton film in the sample chamber; [EO]:[LiMTFSI]:[LiFSI] = 10:0.15:0.85 (molar ratio). Curves are shifted vertically for clarity.

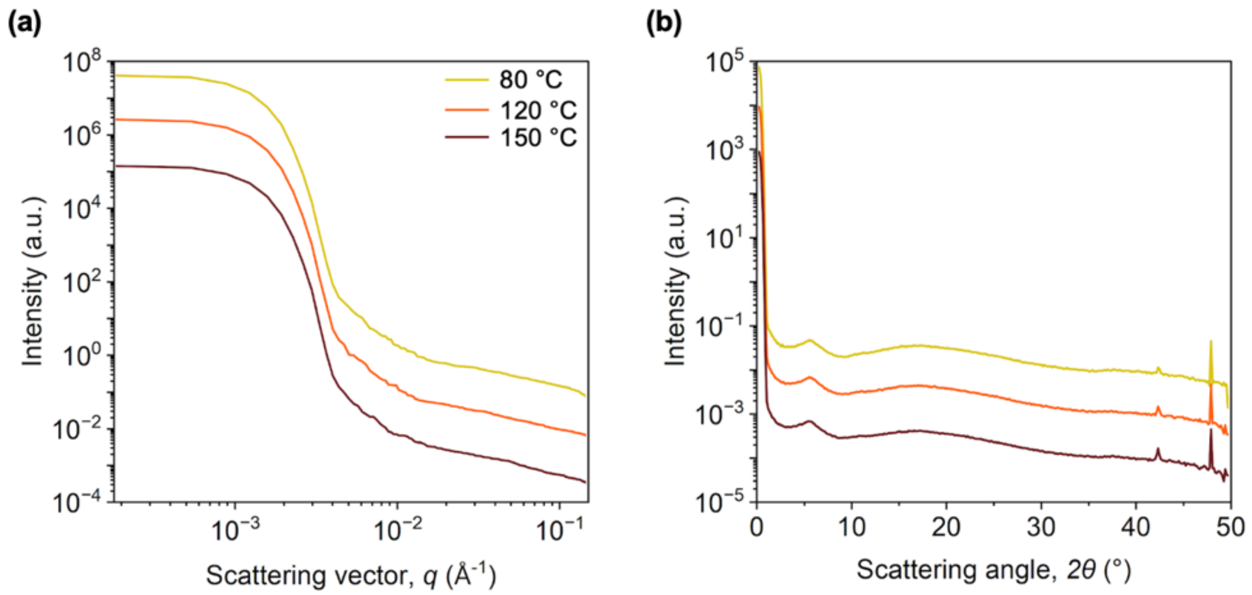

Figure S25. (a) SAXS and (b) WAXS patterns for POEM / PLiMTFSI / LiFSI blend collected upon heating. All patterns are plotted after subtracting the scattering contribution from the Kapton film in the sample chamber; [EO]:[LiMTFSI]:[LiFSI] = 10:0.25:0.75 (molar ratio). Curves are shifted vertically for clarity.

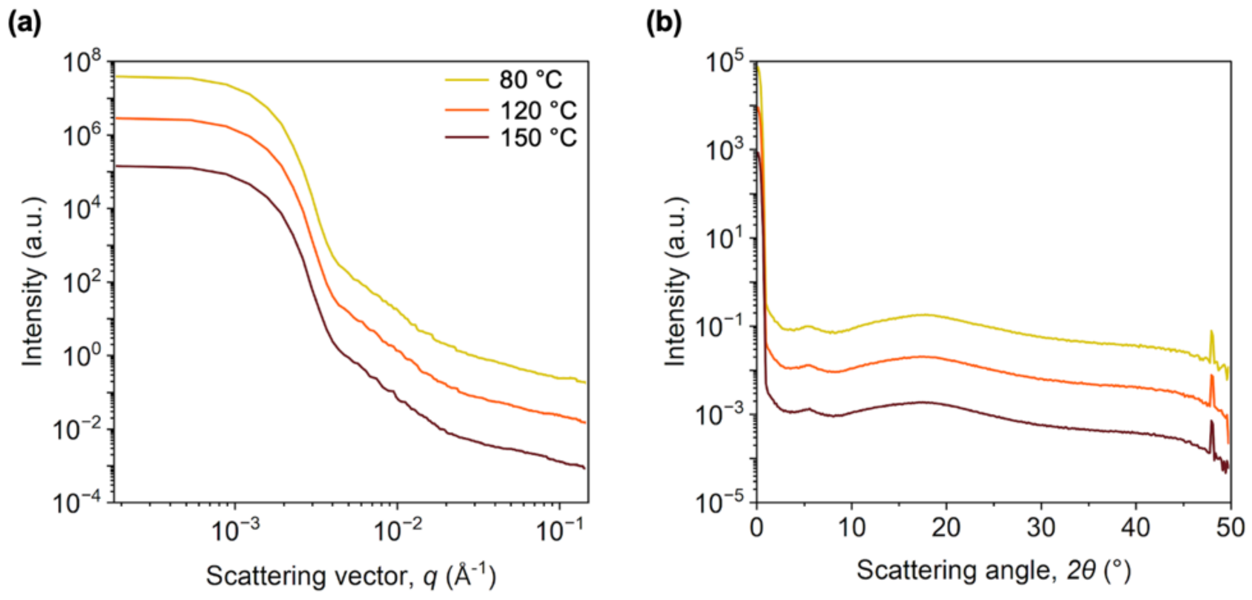

Figure S26. (a) SAXS and (b) WAXS patterns for POEM / PLiMTFSI / LiFSI blend collected upon heating. All patterns are plotted after subtracting the scattering contribution from the Kapton film in the sample chamber; [EO]:[LiMTFSI]:[LiFSI] = 10:0.40:0.60 (molar ratio). Curves are shifted vertically for clarity.

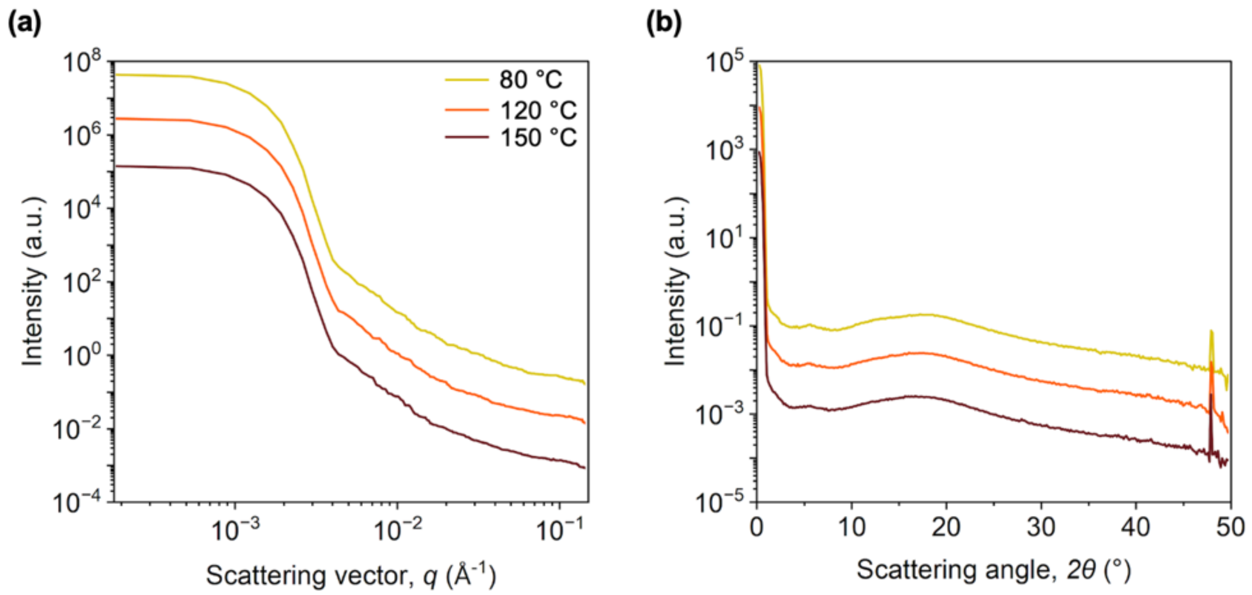

Figure S27. (a) SAXS and (b) WAXS patterns for POEM / PLiMTFSI / LiFSI blend collected upon heating. All patterns are plotted after subtracting the scattering contribution from the Kapton film in the sample chamber; [EO]:[LiMTFSI]:[LiFSI] = 10:0.50:0.50 (molar ratio). Curves are shifted vertically for clarity.

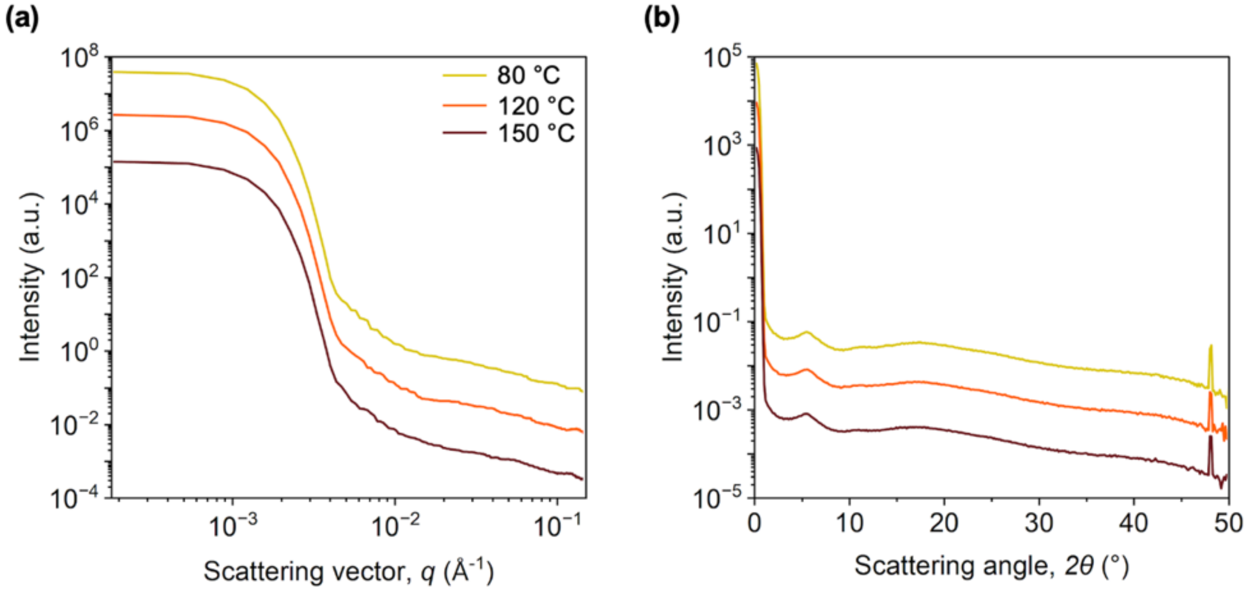

Figure S28. (a) SAXS and (b) WAXS patterns for POEM / PLiMTFSI / LiFSI blend collected upon heating. All patterns are plotted after subtracting the scattering contribution from the Kapton film in the sample chamber; [EO]:[LiMTFSI]:[LiFSI] = 10:0.60:0.40 (molar ratio). Curves are shifted vertically for clarity.

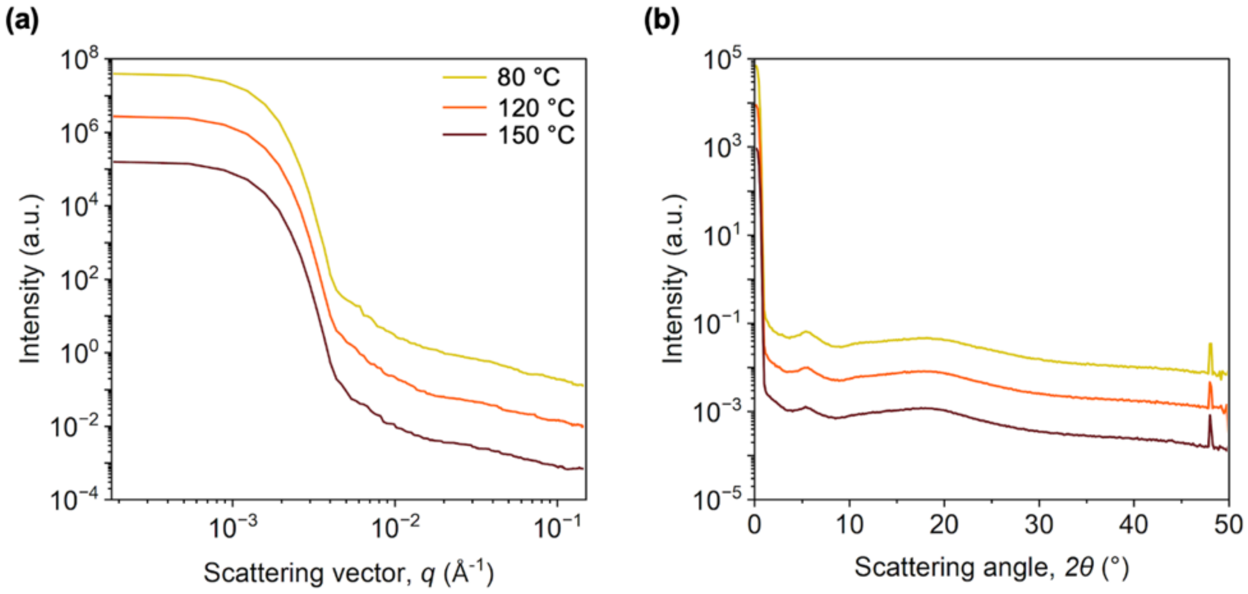

Figure S29. (a) SAXS and (b) WAXS patterns for POEM / PLiMTFSI / LiFSI blend collected upon heating. All patterns are plotted after subtracting the scattering contribution from the Kapton film in the sample chamber; [EO]:[LiMTFSI]:[LiFSI] = 10:0.80:0.20 (molar ratio). Curves are shifted vertically for clarity.

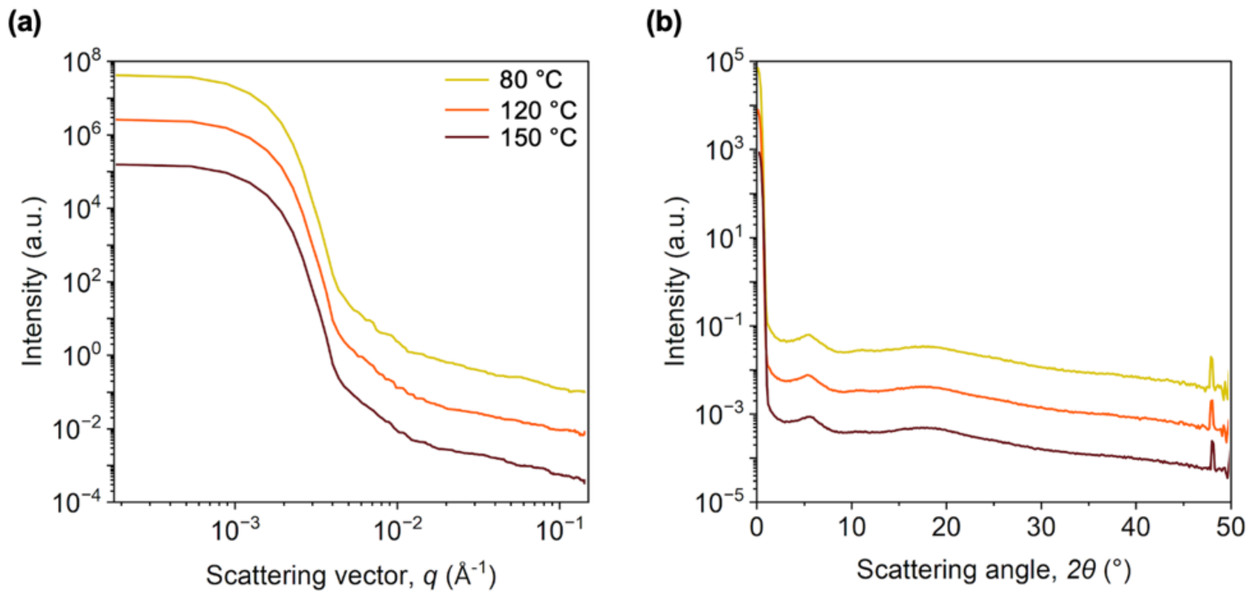

Figure S30. (a) SAXS and (b) WAXS patterns for POEM / PLiMTFSI / LiTf blend collected upon heating. All patterns are plotted after subtracting the scattering contribution from the Kapton film in the sample chamber; [EO]:[LiMTFSI]:[LiTf] = 10:0.05:0.95 (molar ratio). Curves are shifted vertically for clarity.

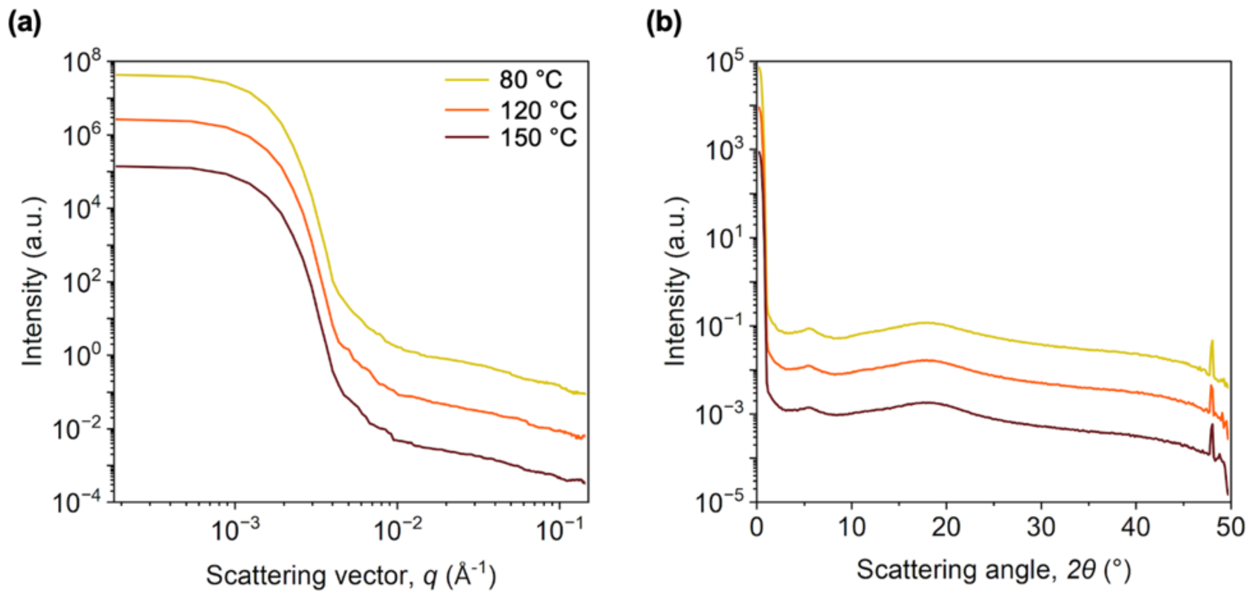

Figure S31. (a) SAXS and (b) WAXS patterns for POEM / PLiMTFSI / LiTf blend collected upon heating. All patterns are plotted after subtracting the scattering contribution from the Kapton film in the sample chamber; [EO]:[LiMTFSI]:[LiTf] = 10:0.15:0.85 (molar ratio). Curves are shifted vertically for clarity.

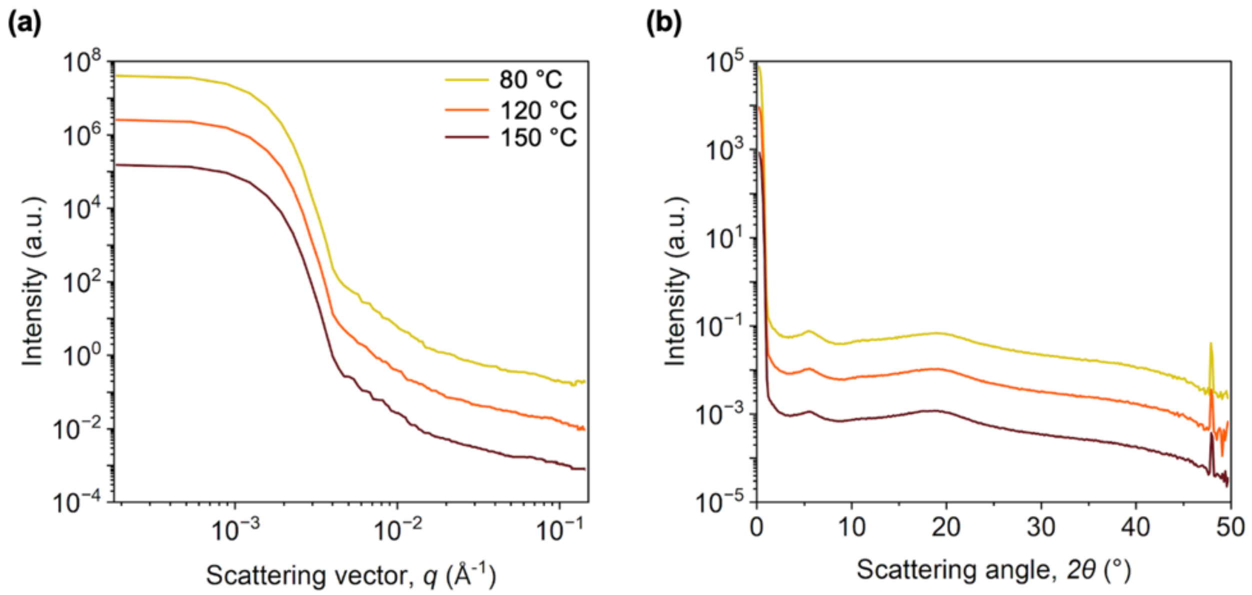

Figure S32. (a) SAXS and (b) WAXS patterns for POEM / PLiMTFSI / LiTf blend collected upon heating. All patterns are plotted after subtracting the scattering contribution from the Kapton film in the sample chamber; [EO]:[LiMTFSI]:[LiTf] = 10:0.25:0.75 (molar ratio). Curves are shifted vertically for clarity.

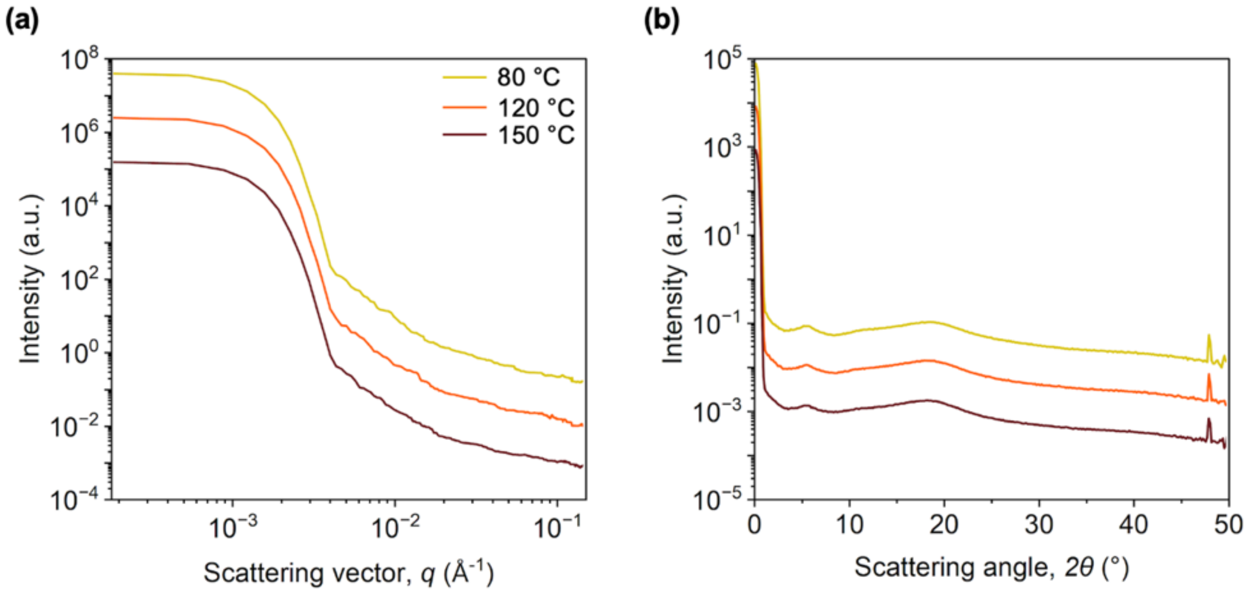

Figure S33. (a) SAXS and (b) WAXS patterns for POEM / PLiMTFSI / LiTf blend collected upon heating. All patterns are plotted after subtracting the scattering contribution from the Kapton film in the sample chamber; [EO]:[LiMTFSI]:[LiTf] = 10:0.40:0.60 (molar ratio). Curves are shifted vertically for clarity.

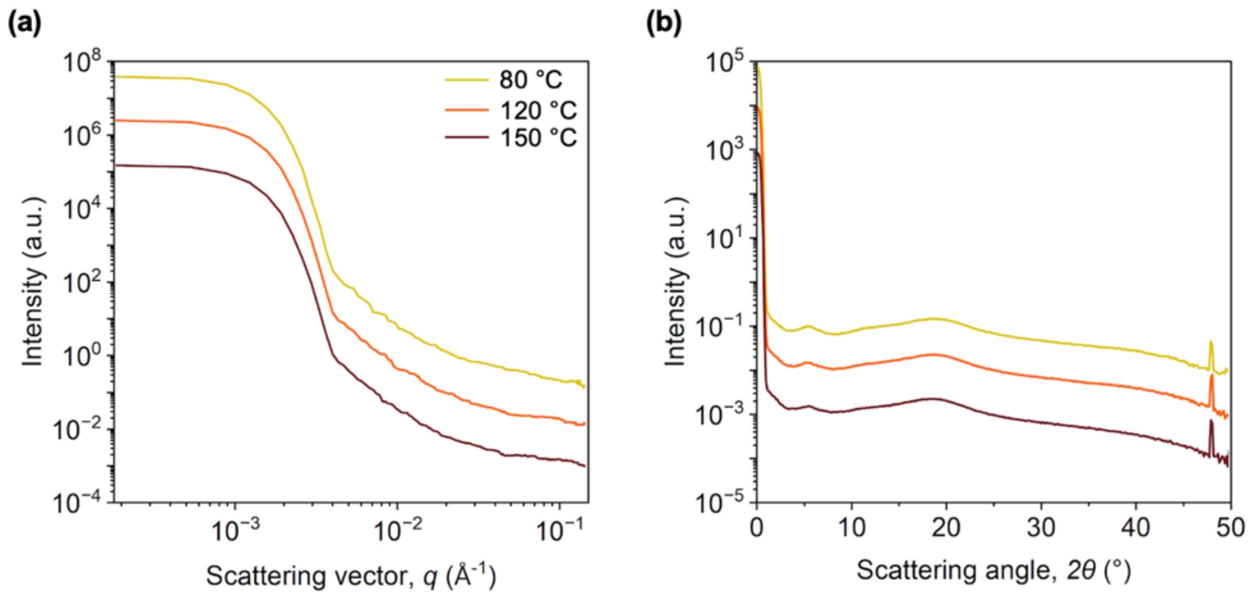

Figure S34. (a) SAXS and (b) WAXS patterns for POEM / PLiMTFSI / LiTf blend collected upon heating. All patterns are plotted after subtracting the scattering contribution from the Kapton film in the sample chamber; [EO]:[LiMTFSI]:[LiTf] = 10:0.50:0.50 (molar ratio). Curves are shifted vertically for clarity.

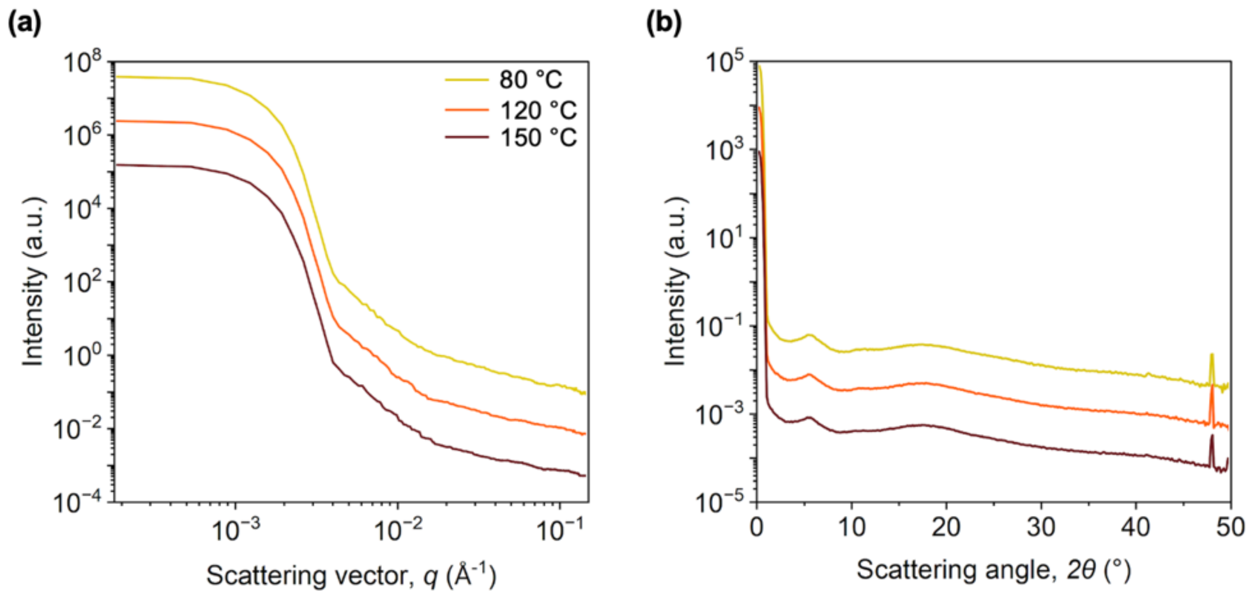

Figure S35. (a) SAXS and (b) WAXS patterns for POEM / PLiMTFSI / LiTf blend collected upon heating. All patterns are plotted after subtracting the scattering contribution from the Kapton film in the sample chamber; [EO]:[LiMTFSI]:[LiTf] = 10:0.60:0.40 (molar ratio). Curves are shifted vertically for clarity.

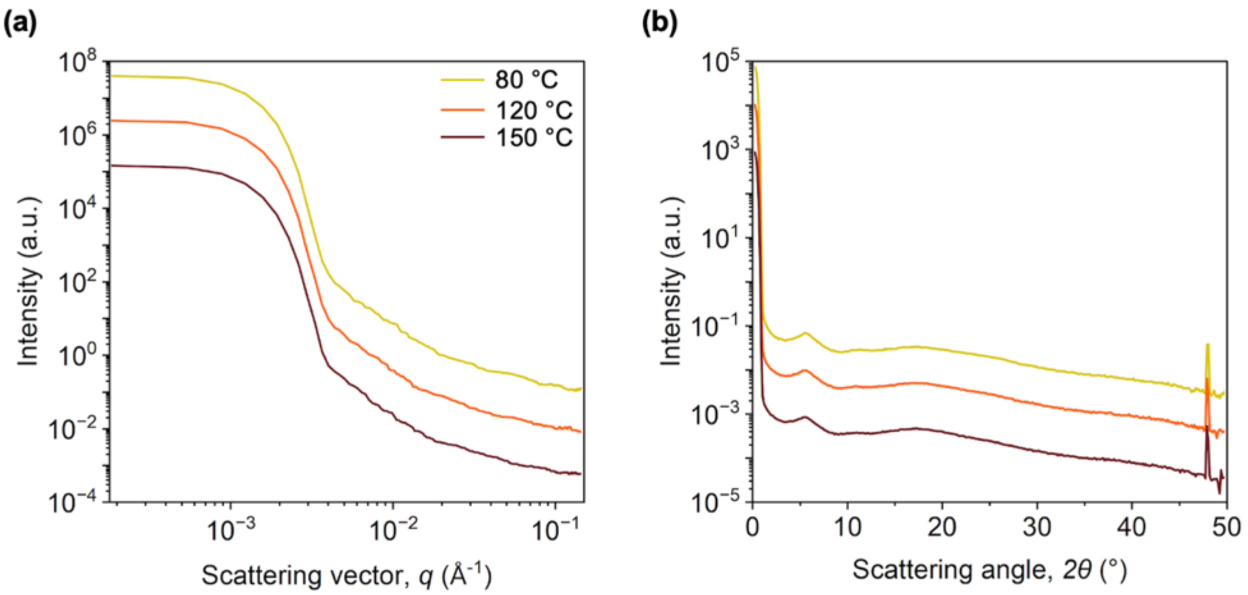

Figure S36. (a) SAXS and (b) WAXS patterns for POEM / PLiMTFSI / LiTf blend collected upon heating. All patterns are plotted after subtracting the scattering contribution from the Kapton film in the sample chamber; [EO]:[LiMTFSI]:[LiTf] = 10:0.80:0.20 (molar ratio). Curves are shifted vertically for clarity.

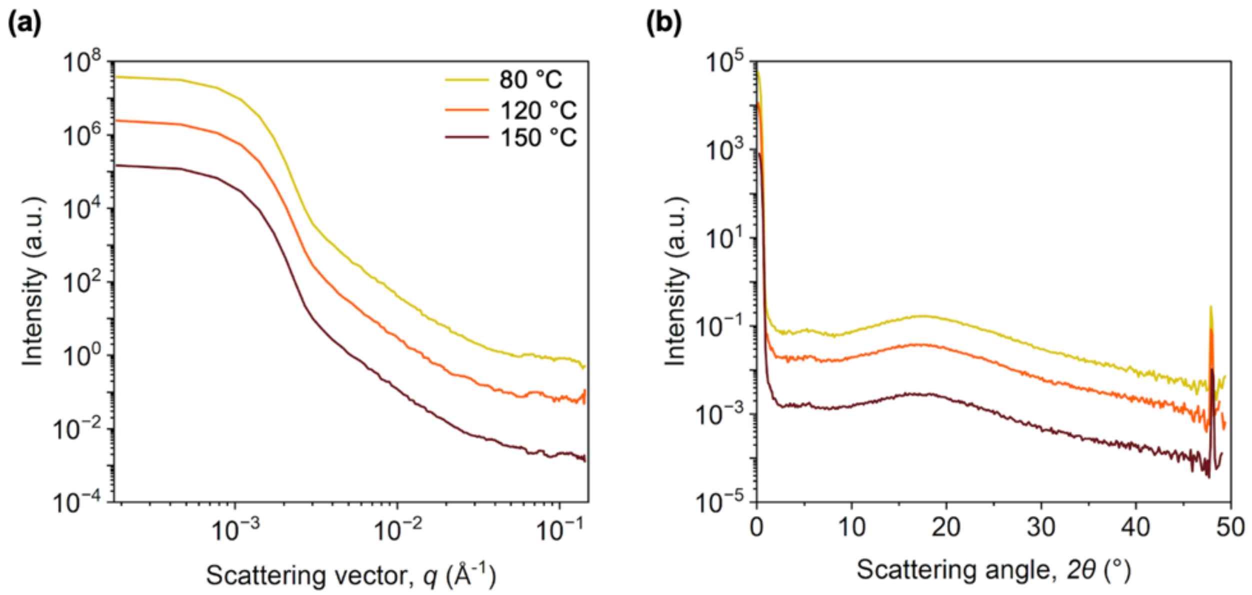

Figure S37. (a) SAXS and (b) WAXS patterns for POEM / PLiMTFSI / $LiClO_4$ blend collected upon heating. All patterns are plotted after subtracting the scattering contribution from the Kapton film in the sample chamber; [EO]:[LiMTFSI]:[$LiClO_4$] = 10:0.05:0.95 (molar ratio). Curves are shifted vertically for clarity.

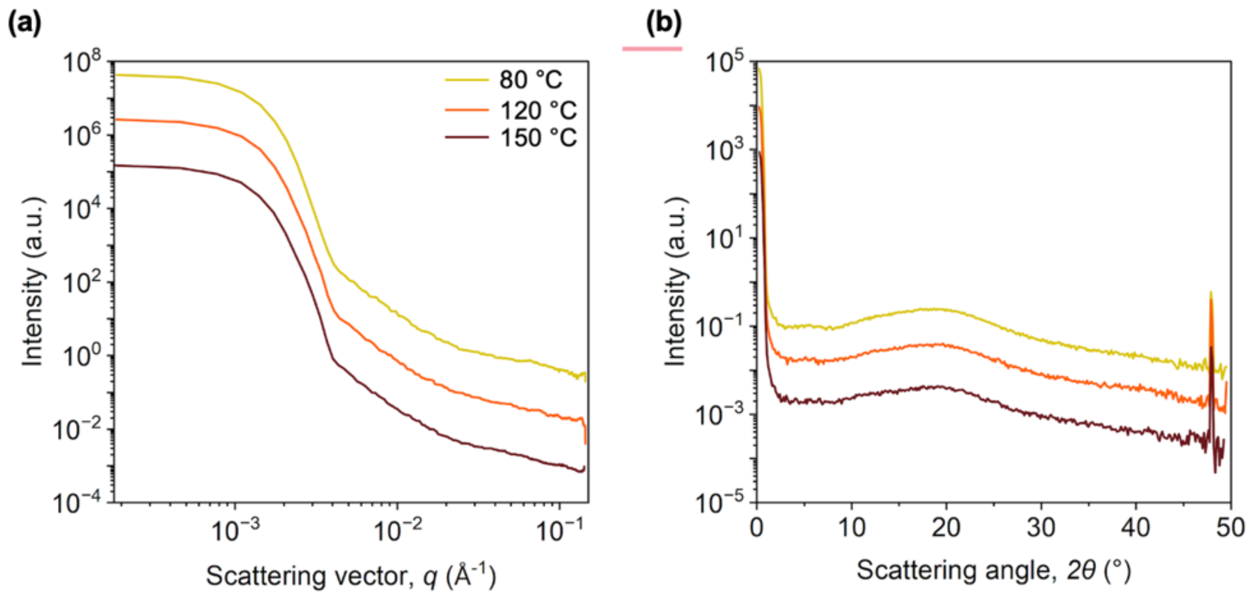

Figure S38. (a) SAXS and (b) WAXS patterns for POEM / PLiMTFSI / $LiClO_4$ blend collected upon heating. All patterns are plotted after subtracting the scattering contribution from the Kapton film in the sample chamber; [EO]:[LiMTFSI]:[$LiClO_4$] = 10:0.15:0.85 (molar ratio). Curves are shifted vertically for clarity.

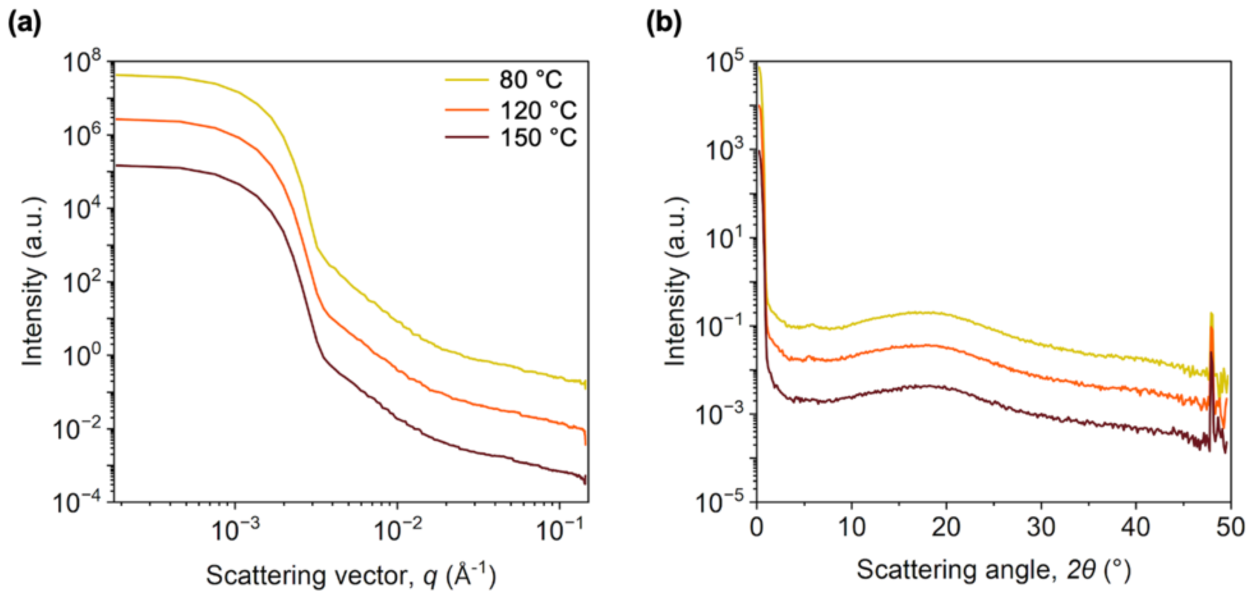

Figure S39. (a) SAXS and (b) WAXS patterns for POEM / PLiMTFSI / $LiClO_4$ blend collected upon heating. All patterns are plotted after subtracting the scattering contribution from the Kapton film in the sample chamber; [EO]:[LiMTFSI]:[$LiClO_4$] = 10:0.25:0.75 (molar ratio). Curves are shifted vertically for clarity.

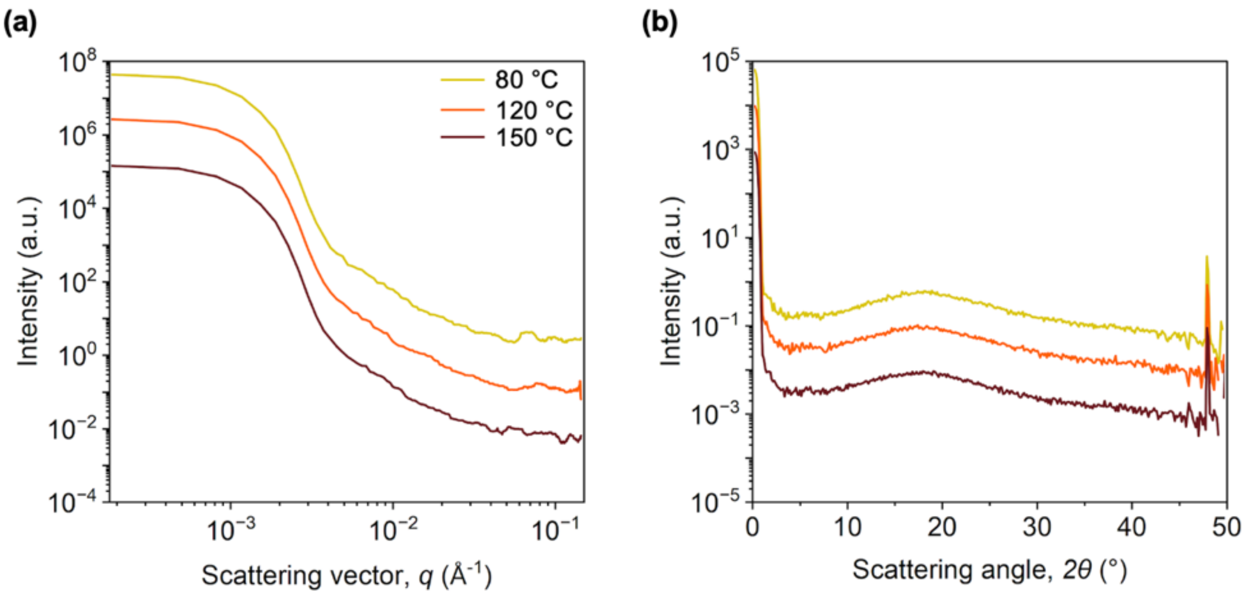

Figure S40. (a) SAXS and (b) WAXS patterns for POEM / PLiMTFSI / $LiClO_4$ blend collected upon heating. All patterns are plotted after subtracting the scattering contribution from the Kapton film in the sample chamber; [EO]:[LiMTFSI]:[$LiClO_4$] = 10:0.40:0.60 (molar ratio). Curves are shifted vertically for clarity.

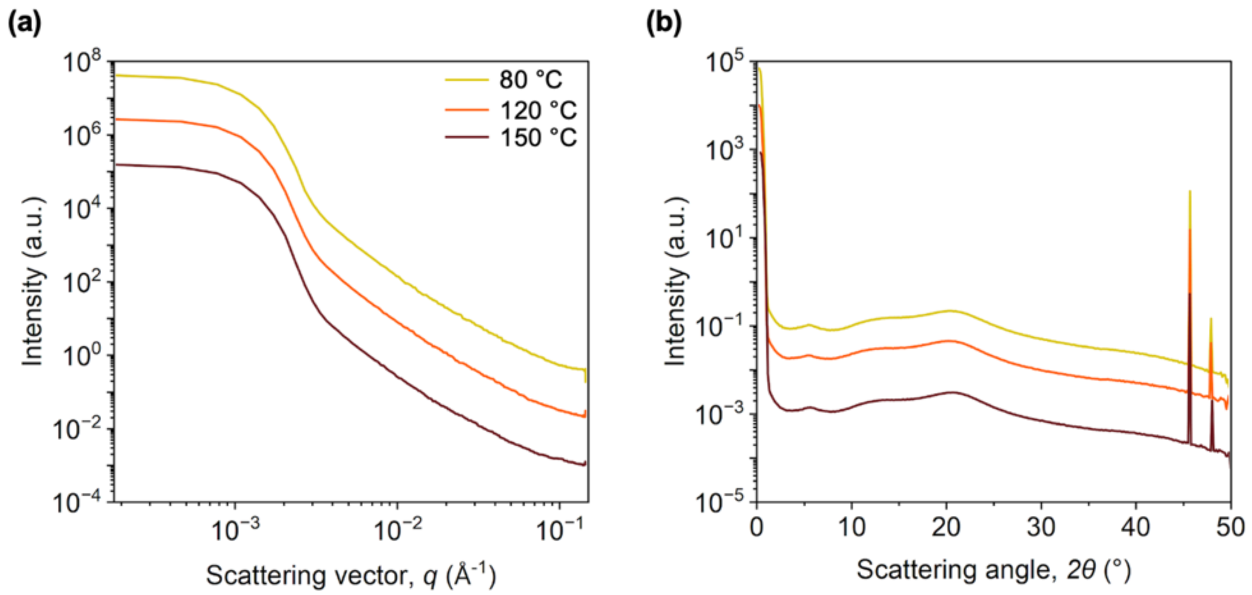

Figure S41. (a) SAXS and (b) WAXS patterns for POEM / PLiMTFSI / $LiClO_4$ blend collected upon heating. All patterns are plotted after subtracting the scattering contribution from the Kapton film in the sample chamber; [EO]:[LiMTFSI]:[$LiClO_4$] = 10:0.50:0.50 (molar ratio). Curves are shifted vertically for clarity.

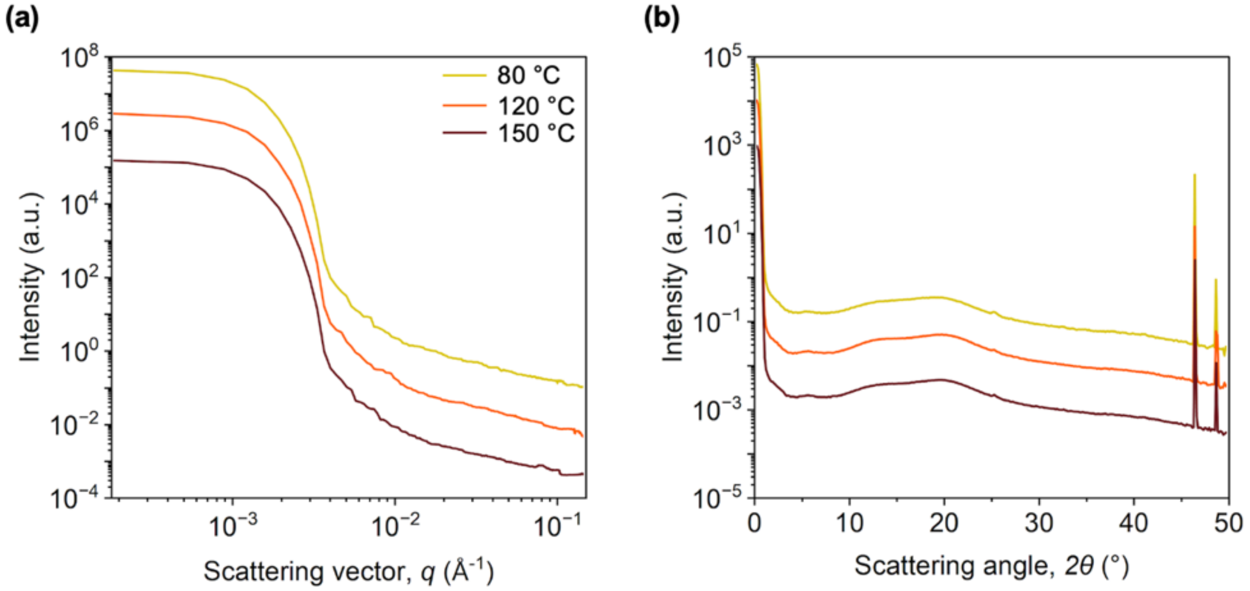

Figure S42. (a) SAXS and (b) WAXS patterns for POEM / PLiMTFSI / $LiClO_4$ blend collected upon heating. All patterns are plotted after subtracting the scattering contribution from the Kapton film in the sample chamber; [EO]:[LiMTFSI]:[$LiClO_4$] = 10:0.60:0.40 (molar ratio). Curves are shifted vertically for clarity.

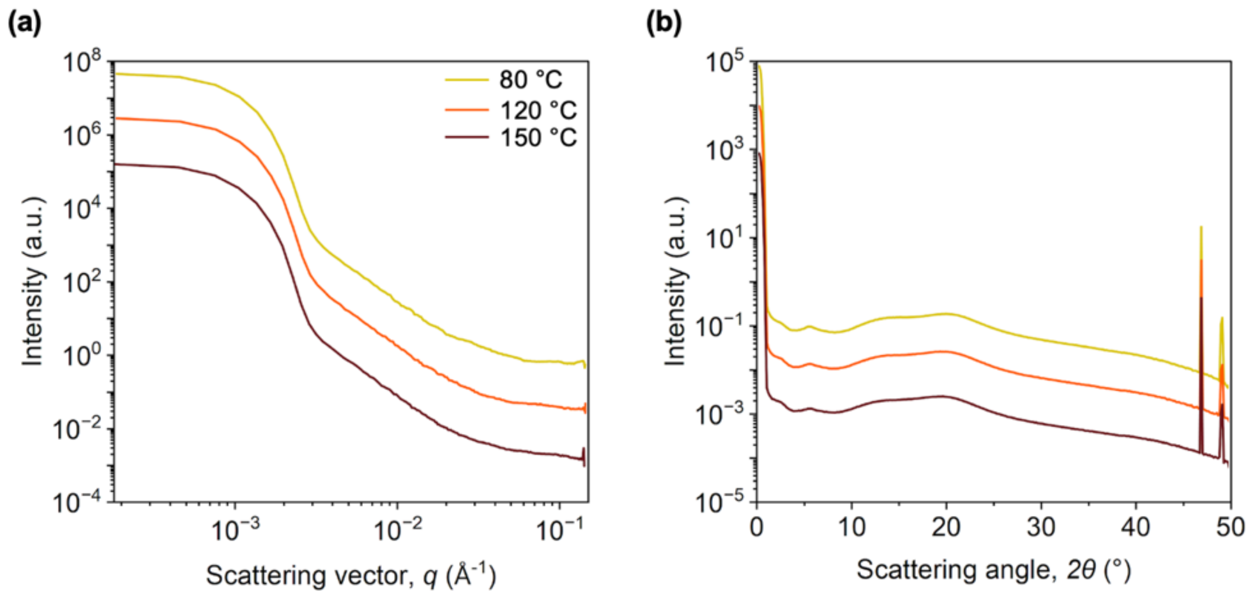

Figure S43. (a) SAXS and (b) WAXS patterns for POEM / PLiMTFSI / $LiClO_4$ blend collected upon heating. All patterns are plotted after subtracting the scattering contribution from the Kapton film in the sample chamber; [EO]:[LiMTFSI]:[$LiClO_4$] = 10:0.80:0.20 (molar ratio). Curves are shifted vertically for clarity.

6. Alternating Current (AC) Impedance Spectroscopy

6.1 POEM / PLiMTFSI / Li salt blends; [EO]:[LiMTFSI]:[Li salt] = 10:0.05:0.95 (molar ratio)

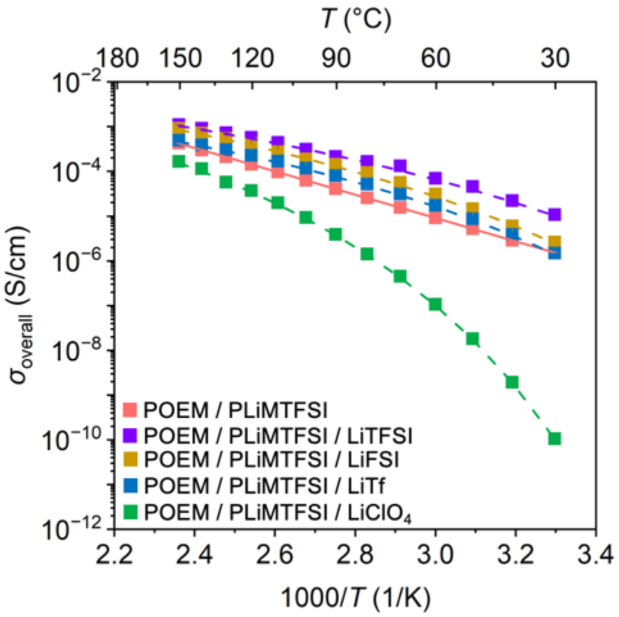

Figure S44. Overall conductivity ($\sigma_{overall}$) *vs.* 1000/*T*. Vogel-Tammann-Fulcher (VTF)-like fits to $\sigma_{overall}$s are shown by dashed lines; Arrhenius-like fits to $\sigma_{overall}$s are shown by solid lines. All the error bars are smaller than the data points and represent the standard deviations from the 5-min and 8-min impedance measurements.

6.2 POEM / PLiMTFSI / Li salt blends; [EO]:[LiMTFSI]:[Li salt] = 10:0.15:0.85 (molar ratio)

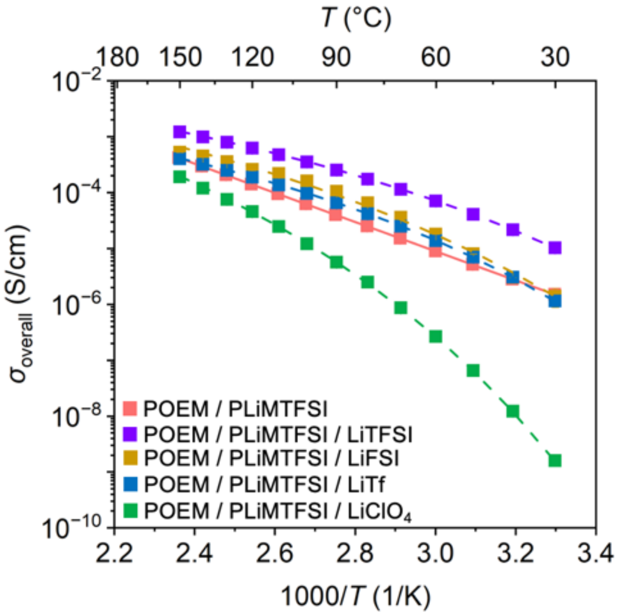

Figure S45. $\sigma_{overall}$ *vs.* 1000/*T*. VTF-like fits to $\sigma_{overall}$s are shown by dashed lines; Arrhenius-like fits to $\sigma_{overall}$s are shown by solid lines. All the error bars are smaller than the data points and represent the standard deviations from the 5-min and 8-min impedance measurements.

6.3 POEM / PLiMTFSI / Li salt blends; [EO]:[LiMTFSI]:[Li salt] = 10:0.25:0.75 (molar ratio)

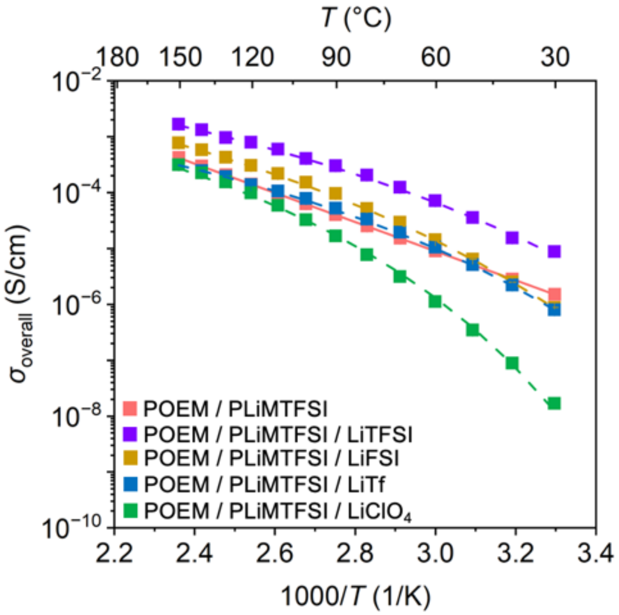

Figure S46. σ_{overall} *vs.* 1000/*T*. VTF-like fits to σ_{overall}s are shown by dashed lines; Arrhenius-like fits to σ_{overall}s are shown by solid lines. All the error bars are smaller than the data points and represent the standard deviations from the 5-min and 8-min impedance measurements.

6.4 POEM / PLiMTFSI / Li salt blends; [EO]:[LiMTFSI]:[Li salt] = 10:0.60:0.40 (molar ratio)

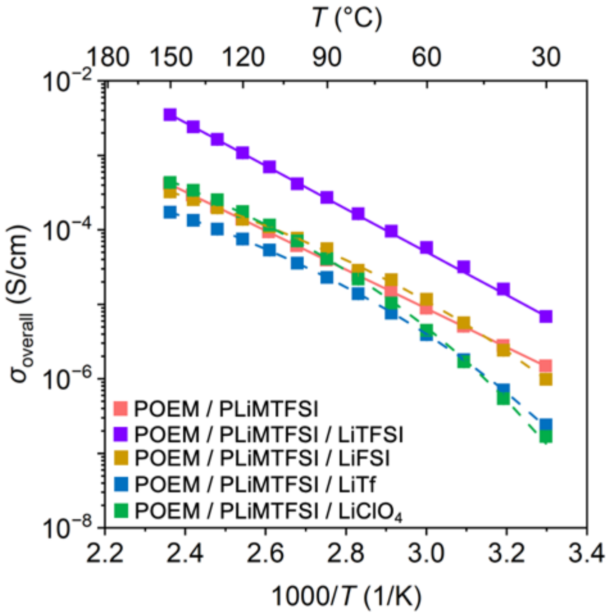

Figure S47. $\sigma_{overall}$ *vs.* 1000/*T*. VTF-like fits to $\sigma_{overall}$s are shown by dashed lines; Arrhenius-like fits to $\sigma_{overall}$s are shown by solid lines. All the error bars are smaller than the data points and represent the standard deviations from the 5-min and 8-min impedance measurements.

6.5 POEM / PLiMTFSI / Li salt blends; [EO]:[LiMTFSI]:[Li salt] = 10:0.80:0.20 (molar ratio)

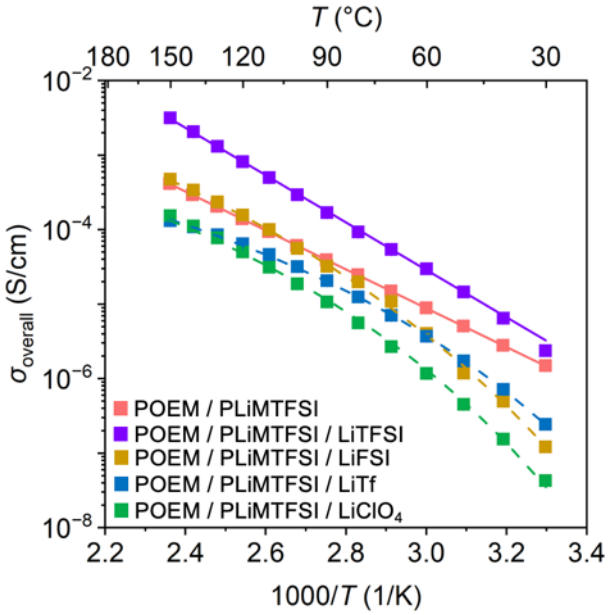

Figure S48. $\sigma_{overall}$ *vs.* 1000/*T*. VTF-like fits to $\sigma_{overall}$s are shown by dashed lines; Arrhenius-like fits to $\sigma_{overall}$s are shown by solid lines. All the error bars are smaller than the data points and represent the standard deviations from the 5-min and 8-min impedance measurements.

7. Electrostatic Force (F_e)

F_e between Li^+ and salt anions and between -O-CH_2- groups in POEM and salt anions was estimated from Coulomb's law [eq (1), see **Tables S3 (a) and S3 (b)**],[1]

$$F_e = \frac{qQ}{4\pi\varepsilon_0 r^2} \quad (1)$$

in which F_e is the electrostatic force between charged species, q and Q are individual charges of interacting species, ε_0 is the dielectric constant of vacuum, and r is the distance between charged species.[1]

Table S3 (a). Estimation of F_e between Li^+ and salt anions.

Anion	$^a q$ (C)	$^a Q$ (C)	$\frac{1}{4\pi\varepsilon_0}$ (N m^2/C^2)	$^b r_{Li+}$ (m)	$^c r_{anion}$ (m)	F_e (N)
$TFSI^-$ (in SIC polymer)					3.32×10^{-10}	9.12×10^{-9}
$TFSI^-$ (in Li salt)	1.60×10^{-19}	1.60×10^{-19}	8.99×10^{9}	7.60×10^{-11}	3.38×10^{-10}	8.96×10^{-8}
FSI^-					2.91×10^{-10}	1.04×10^{-8}
Tf^-					2.76×10^{-10}	1.10×10^{-8}
ClO_4^-					2.40×10^{-10}	1.26×10^{-8}

^{a}For the positively charged Li^+, q was set to be +1 e, whereas for the negatively charged anions, Q was set to be -1 e.[2] $^b r_{Li+}$ was the Li^+ radius obtained from reference 3. $^c r_{anion}$ was the anion radius determined from the van der Waals volume.

Table S3 (b). Estimation of F_e between -O-CH_2- groups in POEM and various salt anions.

Anion	$^a q$ (C)	$^a Q$ (C)	$\frac{1}{4\pi\varepsilon_0}$ (N m^2/C^2)	$^b r_{C\text{–}H}$ (m)	$^c r_{anion}$ (m)	F_e (N)
$TFSI^-$ (in SIC polymer)					3.32×10^{-10}	1.18×10^{-9}
$TFSI^-$ (in Li salt)	1.60×10^{-19}	1.60×10^{-19}	8.99×10^{9}	1.09×10^{-10}	3.38×10^{-10}	1.15×10^{-9}
FSI^-					2.91×10^{-10}	1.44×10^{-9}
Tf^-					2.76×10^{-10}	1.55×10^{-9}
ClO_4^-					2.40×10^{-10}	1.89×10^{-9}

^{a}For the positive dipole moment on the carbon atom in -O-CH_2- groups in POEM, q was set to be +1 e, whereas for the negatively charged anions, Q was set to be -1 e.[2] $^b r_{C\text{–}H}$ was the C-H bond length obtained from reference 4. $^c r_{anion}$ was the anion radius determined from the van der Waals volume.

8. Potentiostatic Polarization

Summary of the parameters for the Bruce-Vincent method for POEM / PLiMTFSI / LiTFSI blends.

Table S4 (a). Impedance and current values extracted from AC impedance spectroscopy and potentiostatic polarization measurements of three lithium–lithium symmetric cells for POEM / PLiMTFSI / LiTFSI blend; [EO]:[LiMTFSI]:[LiTFSI] = 10:0.05:0.95 (molar ratio) at 60 °C.

	${}^{b}R_1$ (Ohms)	${}^{b}R_2$ (Ohms)	${}^{c}R_1^*$ (Ohms)	${}^{c}R_2^*$ (Ohms)	${}^{d}R_0$ (Ohms)	${}^{e}R_{SS}$ (Ohms)	${}^{f}I_0$ (A)	I_{SS} (A)	t_{Li+}
[a]Cell A	2435	3970	2430	3900	1535	1470	2.52×10^{-6}	8.80×10^{-7}	0.35
[a]Cell B	2430	3900	2700	4150	1470	1450	2.56×10^{-6}	8.98×10^{-7}	0.35
[a]Cell C	2430	3450	2400	3790	1020	1390	2.90×10^{-6}	1.01×10^{-6}	0.35

[a]Cells A, B, and C were made with lithium non-blocking electrodes. [b]R_1 and R_2 are the impedances of the minima at the bounds of the low-frequency semicircle in a Nyquist plot before cell polarization. [c]R_1* and R_2* are the corresponding impedances after polarization. [d]Initial interfacial impedance was defined as $R_0 = R_2 - R_1$. [e]Steady-state interfacial impedance was defined as $R_{SS} = R_2^* - R_1^*$. [f]Initial current, I_0, was calculated by Ohm's law. This footnote applies to all tables in this section.

Table S4 (b). Impedance and current values extracted from AC impedance spectroscopy and potentiostatic polarization measurements of three lithium–lithium symmetric cells for POEM / PLiMTFSI / LiTFSI blend; [EO]:[LiMTFSI]:[LiTFSI] = 10:0.05:0.95 (molar ratio) at 100 °C.

	${}^{b}R_1$ (Ohms)	${}^{b}R_2$ (Ohms)	${}^{c}R_1^*$ (Ohms)	${}^{c}R_2^*$ (Ohms)	${}^{d}R_0$ (Ohms)	${}^{e}R_{SS}$ (Ohms)	${}^{f}I_0$ (A)	I_{SS} (A)	t_{Li+}
[a]Cell A	550	720	570	760	170	190	1.39×10^{-5}	5.06×10^{-6}	0.36
[a]Cell B	550	660	570	690	110	120	1.52×10^{-5}	5.47×10^{-6}	0.36
[a]Cell C	540	650	585	680	110	95	1.54×10^{-5}	5.61×10^{-6}	0.36

Table S5 (a). Impedance and current values extracted from AC impedance spectroscopy and potentiostatic polarization measurements of three lithium–lithium symmetric cells for POEM / PLiMTFSI / LiTFSI blend; [EO]:[LiMTFSI]:[LiTFSI] = 10:0.15:0.85 (molar ratio) at 60 °C.

	$^{b}R_1$ (Ohms)	$^{b}R_2$ (Ohms)	$^{c}R_1^*$ (Ohms)	$^{c}R_2^*$ (Ohms)	$^{d}R_0$ (Ohms)	$^{e}R_{SS}$ (Ohms)	$^{f}I_0$ (A)	I_{SS} (A)	t_{Li+}
^{a}Cell A	1450	2200	1630	2370	750	740	4.55×10^{-6}	2.18×10^{-6}	0.48
^{a}Cell B	1500	2580	1630	2720	1080	1090	3.88×10^{-6}	1.87×10^{-6}	0.48
^{a}Cell C	1450	2215	1610	2295	765	685	4.51×10^{-6}	2.17×10^{-6}	0.48

Table S5 (b). Impedance and current values extracted from AC impedance spectroscopy and potentiostatic polarization measurements of three lithium–lithium symmetric cells for POEM / PLiMTFSI / LiTFSI blend; [EO]:[LiMTFSI]:[LiTFSI] = 10:0.15:0.85 (molar ratio) at 100 °C.

	$^{b}R_1$ (Ohms)	$^{b}R_2$ (Ohms)	$^{c}R_1^*$ (Ohms)	$^{c}R_2^*$ (Ohms)	$^{d}R_0$ (Ohms)	$^{e}R_{SS}$ (Ohms)	$^{f}I_0$ (A)	I_{SS} (A)	t_{Li+}
^{a}Cell A	450	780	500	810	330	310	1.28×10^{-5}	6.16×10^{-6}	0.48
^{a}Cell B	450	690	490	720	240	230	1.45×10^{-5}	6.96×10^{-6}	0.48
^{a}Cell C	450	780	520	825	330	305	1.28×10^{-5}	6.16×10^{-6}	0.48

Table S6 (a). Impedance and current values extracted from AC impedance spectroscopy and potentiostatic polarization measurements of three lithium–lithium symmetric cells for POEM / PLiMTFSI / LiTFSI blend; [EO]:[LiMTFSI]:[LiTFSI] = 10:0.25:0.75 (molar ratio) at 60 °C.

	$^{b}R_1$ (Ohms)	$^{b}R_2$ (Ohms)	$^{c}R_1^*$ (Ohms)	$^{c}R_2^*$ (Ohms)	$^{d}R_0$ (Ohms)	$^{e}R_{SS}$ (Ohms)	$^{f}I_0$ (A)	I_{SS} (A)	t_{Li+}
^{a}Cell A	2350	2500	2590	2840	150	250	4.00×10^{-6}	2.24×10^{-6}	0.56
^{a}Cell B	2325	2450	2540	2825	125	285	4.08×10^{-6}	2.25×10^{-6}	0.55
^{a}Cell C	2365	2500	2520	2750	135	230	4.00×10^{-6}	2.24×10^{-6}	0.56

Table S6 (b). Impedance and current values extracted from AC impedance spectroscopy and potentiostatic polarization measurements of three lithium–lithium symmetric cells for POEM / PLiMTFSI / LiTFSI blend; [EO]:[LiMTFSI]:[LiTFSI] = 10:0.25:0.75 (molar ratio) at 100 °C.

	$^{b}R_1$ (Ohms)	$^{b}R_2$ (Ohms)	$^{c}R_1^*$ (Ohms)	$^{c}R_2^*$ (Ohms)	$^{d}R_0$ (Ohms)	$^{e}R_{SS}$ (Ohms)	$^{f}I_0$ (A)	I_{SS} (A)	t_{Li+}
[a]Cell A	400	445	445	485	45	40	2.25×10^{-5}	1.33×10^{-5}	0.59
[a]Cell B	400	430	440	480	30	40	2.33×10^{-5}	1.37×10^{-5}	0.59
[a]Cell C	410	435	460	490	25	30	2.30×10^{-5}	1.36×10^{-5}	0.59

Table S7 (a). Impedance and current values extracted from AC impedance spectroscopy and potentiostatic polarization measurements of three lithium–lithium symmetric cells for POEM / PLiMTFSI / LiTFSI blend; [EO]:[LiMTFSI]:[LiTFSI] = 10:0.40:0.60 (molar ratio) at 60 °C.

	$^{b}R_1$ (Ohms)	$^{b}R_2$ (Ohms)	$^{c}R_1^*$ (Ohms)	$^{c}R_2^*$ (Ohms)	$^{d}R_0$ (Ohms)	$^{e}R_{SS}$ (Ohms)	$^{f}I_0$ (A)	I_{SS} (A)	t_{Li+}
[a]Cell A	1500	1610	1550	1700	110	150	6.21×10^{-6}	3.98×10^{-6}	0.64
[a]Cell B	1460	1565	1600	1700	105	100	6.39×10^{-6}	4.09×10^{-6}	0.64
[a]Cell C	1470	1560	1550	1660	90	110	6.41×10^{-6}	4.17×10^{-6}	0.65

Table S7 (b). Impedance and current values extracted from AC impedance spectroscopy and potentiostatic polarization measurements of three lithium–lithium symmetric cells for POEM / PLiMTFSI / LiTFSI blend; [EO]:[LiMTFSI]:[LiTFSI] = 10:0.40:0.60 (molar ratio) at 100 °C.

	$^{b}R_1$ (Ohms)	$^{b}R_2$ (Ohms)	$^{c}R_1^*$ (Ohms)	$^{c}R_2^*$ (Ohms)	$^{d}R_0$ (Ohms)	$^{e}R_{SS}$ (Ohms)	$^{f}I_0$ (A)	I_{SS} (A)	t_{Li+}
[a]Cell A	250	270	270	290	20	20	3.70×10^{-5}	2.45×10^{-5}	0.66
[a]Cell B	240	265	250	270	25	20	3.77×10^{-5}	2.45×10^{-5}	0.65
[a]Cell C	255	280	265	285	25	20	3.57×10^{-5}	2.32×10^{-5}	0.65

Table S8 (a). Impedance and current values extracted from AC impedance spectroscopy and potentiostatic polarization measurements of three lithium–lithium symmetric cells for POEM / PLiMTFSI / LiTFSI blend; [EO]:[LiMTFSI]:[LiTFSI] = 10:0.50:0.50 (molar ratio) at 60 °C.

	$^{b}R_1$ (Ohms)	$^{b}R_2$ (Ohms)	$^{c}R_1^*$ (Ohms)	$^{c}R_2^*$ (Ohms)	$^{d}R_0$ (Ohms)	$^{e}R_{SS}$ (Ohms)	$^{f}I_0$ (A)	I_{SS} (A)	t_{Li+}
^{a}Cell A	1300	1390	1380	1460	90	80	7.22×10^{-6}	4.69×10^{-6}	0.65
^{a}Cell B	1300	1370	1310	1420	70	110	7.30×10^{-6}	4.75×10^{-6}	0.65
^{a}Cell C	1350	1500	1420	1560	150	140	6.72×10^{-6}	4.37×10^{-6}	0.65

Table S8 (b). Impedance and current values extracted from AC impedance spectroscopy and potentiostatic polarization measurements of three lithium–lithium symmetric cells for POEM / PLiMTFSI / LiTFSI blend; [EO]:[LiMTFSI]:[LiTFSI] = 10:0.50:0.50 (molar ratio) at 100 °C.

	$^{b}R_1$ (Ohms)	$^{b}R_2$ (Ohms)	$^{c}R_1^*$ (Ohms)	$^{c}R_2^*$ (Ohms)	$^{d}R_0$ (Ohms)	$^{e}R_{SS}$ (Ohms)	$^{f}I_0$ (A)	I_{SS} (A)	t_{Li+}
^{a}Cell A	220	230	220	240	10	20	4.35×10^{-5}	2.87×10^{-5}	0.66
^{a}Cell B	220	240	220	250	20	30	4.17×10^{-5}	2.71×10^{-5}	0.65
^{a}Cell C	210	240	210	240	30	30	4.26×10^{-5}	2.81×10^{-5}	0.66

Table S9 (a). Impedance and current values extracted from AC impedance spectroscopy and potentiostatic polarization measurements of three lithium–lithium symmetric cells for POEM / PLiMTFSI / LiTFSI blend; [EO]:[LiMTFSI]:[LiTFSI] = 10:0.60:0.40 (molar ratio) at 60 °C.

	$^{b}R_1$ (Ohms)	$^{b}R_2$ (Ohms)	$^{c}R_1^*$ (Ohms)	$^{c}R_2^*$ (Ohms)	$^{d}R_0$ (Ohms)	$^{e}R_{SS}$ (Ohms)	$^{f}I_0$ (A)	I_{SS} (A)	t_{Li+}
^{a}Cell A	2770	3100	2900	3210	330	310	3.23×10^{-6}	2.32×10^{-6}	0.72
^{a}Cell B	2780	3070	2800	3150	290	350	3.26×10^{-6}	2.35×10^{-6}	0.72
^{a}Cell C	2770	3060	2820	3140	290	320	3.27×10^{-6}	2.32×10^{-6}	0.71

Table S9 (b). Impedance and current values extracted from AC impedance spectroscopy and potentiostatic polarization measurements of three lithium–lithium symmetric cells for POEM / PLiMTFSI / LiTFSI blend; [EO]:[LiMTFSI]:[LiTFSI] = 10:0.60:0.40 (molar ratio) at 100 °C.

	$^{b}R_1$ (Ohms)	$^{b}R_2$ (Ohms)	$^{c}R_1^*$ (Ohms)	$^{c}R_2^*$ (Ohms)	$^{d}R_0$ (Ohms)	$^{e}R_{SS}$ (Ohms)	$^{f}I_0$ (A)	I_{SS} (A)	t_{Li+}
[a]Cell A	390	480	410	480	90	70	2.08×10^{-5}	1.52×10^{-5}	0.72
[a]Cell B	390	480	410	500	90	90	2.08×10^{-5}	1.52×10^{-5}	0.73
[a]Cell C	390	480	415	490	90	75	2.08×10^{-5}	1.52×10^{-5}	0.73

Table S10 (a). Impedance and current values extracted from AC impedance spectroscopy and potentiostatic polarization measurements of three lithium–lithium symmetric cells for POEM / PLiMTFSI / LiTFSI blend; [EO]:[LiMTFSI]:[LiTFSI] = 10:0.80:0.20 (molar ratio) at 60 °C.

	$^{b}R_1$ (Ohms)	$^{b}R_2$ (Ohms)	$^{c}R_1^*$ (Ohms)	$^{c}R_2^*$ (Ohms)	$^{d}R_0$ (Ohms)	$^{e}R_{SS}$ (Ohms)	$^{f}I_0$ (A)	I_{SS} (A)	t_{Li+}
[a]Cell A	5450	6090	5630	6200	640	570	1.64×10^{-6}	1.33×10^{-6}	0.81
[a]Cell B	5430	5880	5630	6080	450	450	1.70×10^{-6}	1.36×10^{-6}	0.80
[a]Cell C	5440	6330	5660	6480	890	820	1.58×10^{-6}	1.27×10^{-6}	0.80

Table S10 (b). Impedance and current values extracted from AC impedance spectroscopy and potentiostatic polarization measurements of three lithium–lithium symmetric cells for POEM / PLiMTFSI / LiTFSI blend; [EO]:[LiMTFSI]:[LiTFSI] = 10:0.80:0.20 (molar ratio) at 100 °C.

	$^{b}R_1$ (Ohms)	$^{b}R_2$ (Ohms)	$^{c}R_1^*$ (Ohms)	$^{c}R_2^*$ (Ohms)	$^{d}R_0$ (Ohms)	$^{e}R_{SS}$ (Ohms)	$^{f}I_0$ (A)	I_{SS} (A)	t_{Li+}
[a]Cell A	550	620	580	640	70	60	1.61×10^{-5}	1.31×10^{-5}	0.81
[a]Cell B	550	630	570	650	80	80	1.59×10^{-5}	1.29×10^{-5}	0.81
[a]Cell C	550	640	580	660	90	80	1.56×10^{-5}	1.27×10^{-5}	0.81

8.1 Measurements conducted at 60 °C for POEM / PLiMTFSI / LiTFSI blends

8.1.1 POEM / PLiMTFSI / LiTFSI blend; [EO]:[LiMTFSI]:[LiTFSI] = 10:0.05:0.95 (molar ratio)

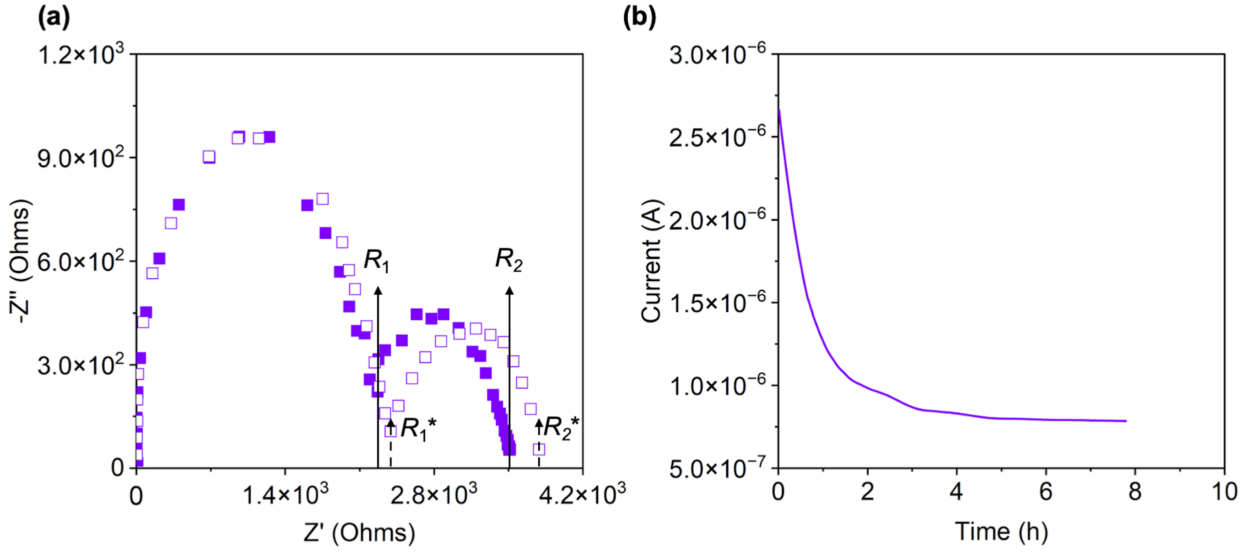

Figure S49. (a) Imaginary impedance (Z″) as a function of the real impedance (Z′) for lithium–lithium symmetric cell A before (filled squares) and after polarization (hollow squares). (b) Current *vs.* time during cell polarization. R_1 and R_2, which are the impedances of the minima at the bounds of the low-frequency semicircle in a Nyquist plot before cell polarization, R_1* and R_2*, which are the corresponding impedances after polarization, are shown in (a).

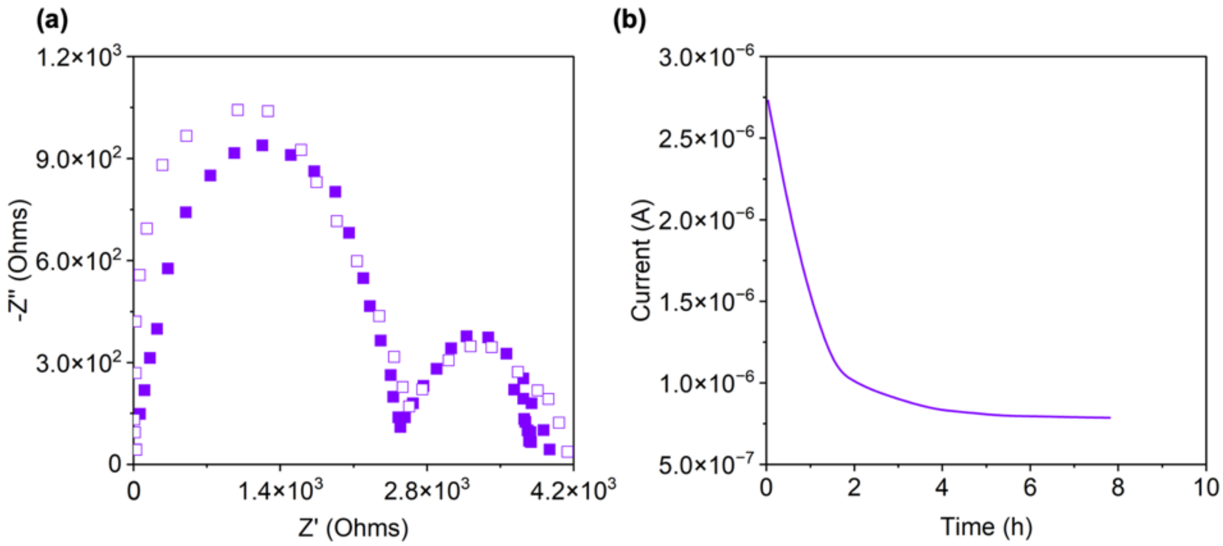

Figure S50. (a) Z'' as a function of Z' for lithium–lithium symmetric cell B before (filled squares) and after polarization (hollow squares). (b) Current *vs.* time during cell polarization.

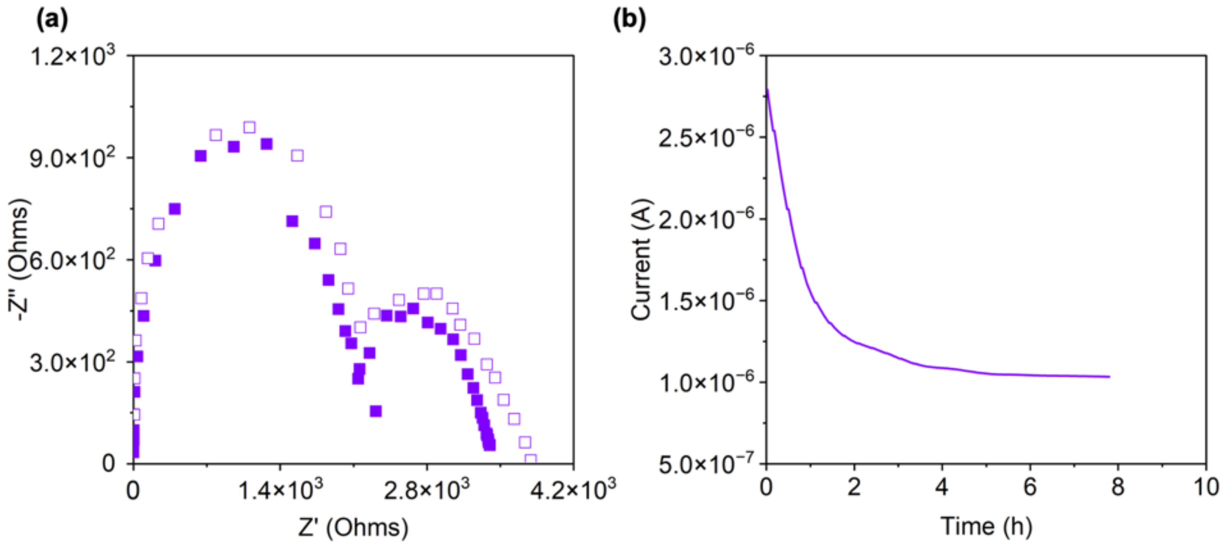

Figure S51. (a) Z'' as a function of Z' for lithium–lithium symmetric cell C before (filled squares) and after polarization (hollow squares). (b) Current *vs.* time during cell polarization.

8.1.2 POEM / PLiMTFSI / LiTFSI blend; [EO]:[LiMTFSI]:[LiTFSI] = 10:0.15:0.85 (molar ratio)

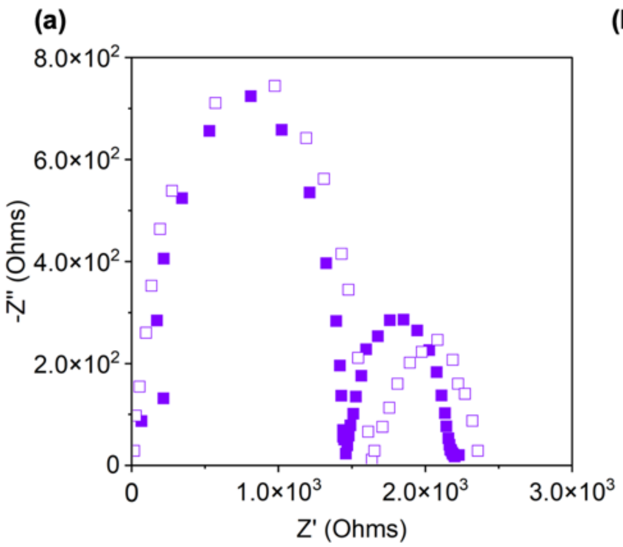

Figure S52. (a) Z'' as a function of Z' for lithium–lithium symmetric cell A before (filled squares) and after polarization (hollow squares). (b) Current *vs.* time during cell polarization.

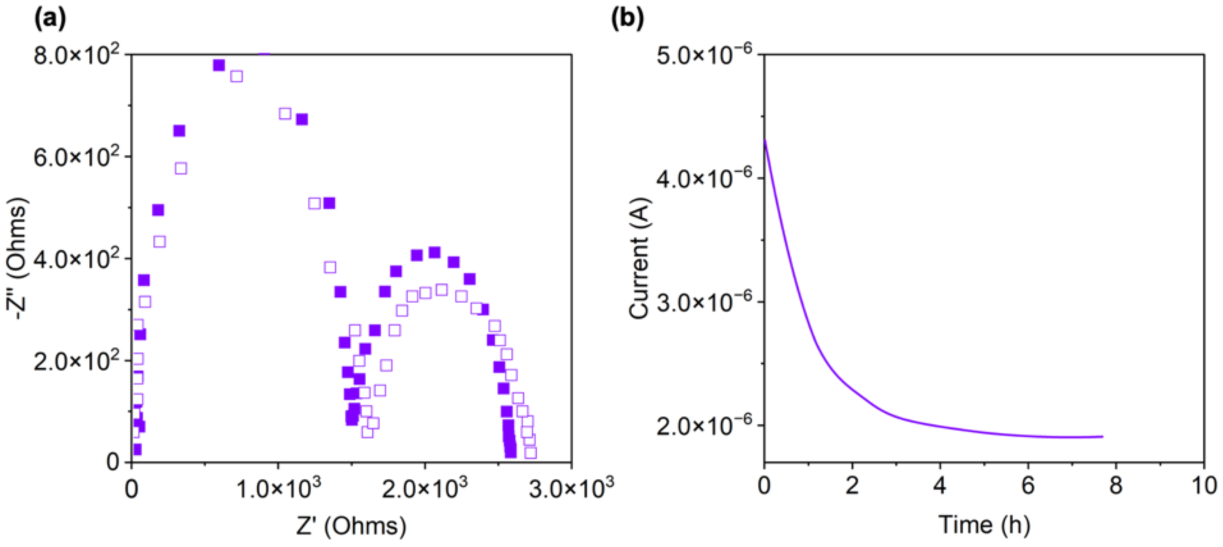

Figure S53. (a) Z'' as a function of Z' for lithium–lithium symmetric cell B before (filled squares) and after polarization (hollow squares). (b) Current *vs.* time during cell polarization.

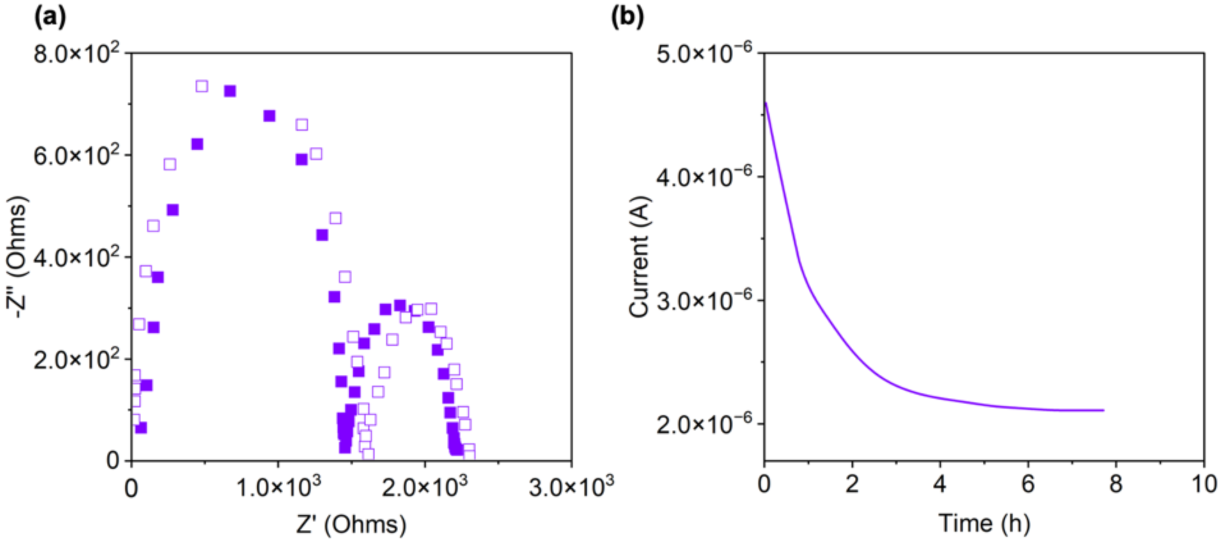

Figure S54. (a) Z'' as a function of Z' for lithium–lithium symmetric cell C before (filled squares) and after polarization (hollow squares). (b) Current *vs.* time during cell polarization.

8.1.3 POEM / PLiMTFSI / LiTFSI blend; [EO]:[LiMTFSI]:[LiTFSI] = 10:0.25:0.75 (molar ratio)

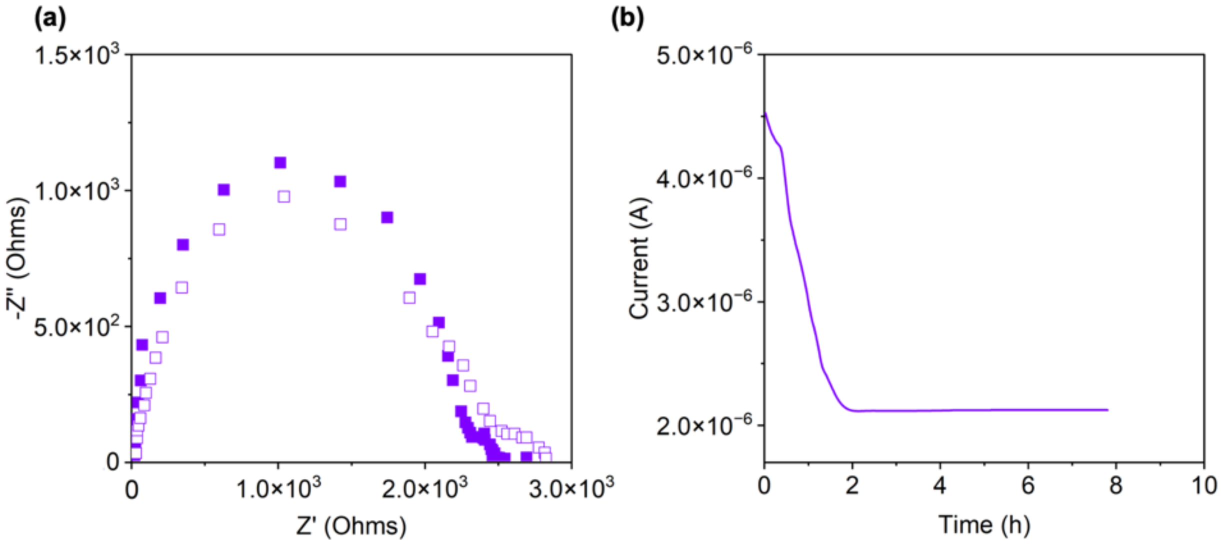

Figure S55. (a) Z'' as a function of Z' for lithium–lithium symmetric cell A before (filled squares) and after polarization (hollow squares). (b) Current *vs.* time during cell polarization.

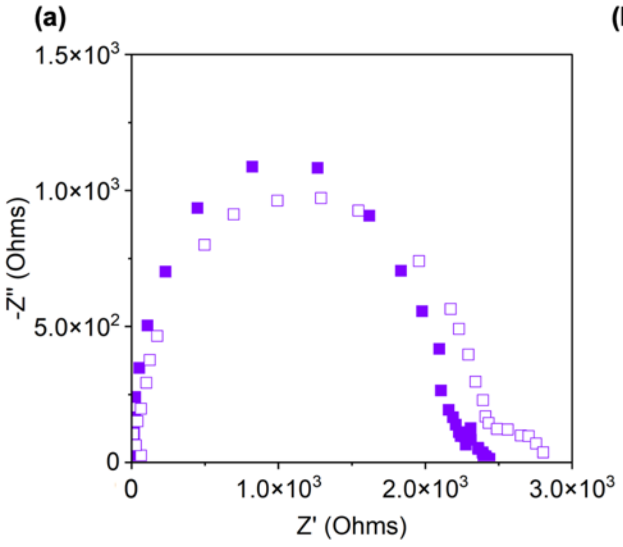

Figure S56. (a) Z'' as a function of Z' for lithium–lithium symmetric cell B before (filled squares) and after polarization (hollow squares). (b) Current *vs.* time during cell polarization.

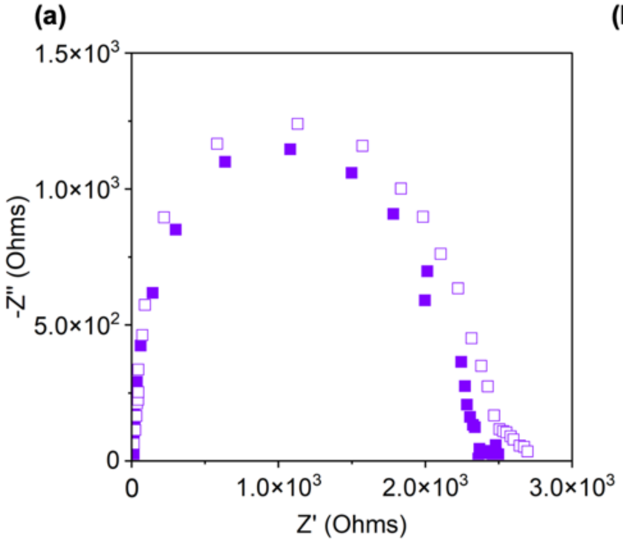

Figure S57. (a) Z'' as a function of Z' for lithium–lithium symmetric cell C before (filled squares) and after polarization (hollow squares). (b) Current *vs.* time during cell polarization.

8.1.4 POEM / PLiMTFSI / LiTFSI blend; [EO]:[LiMTFSI]:[LiTFSI] = 10:0.40:0.60 (molar ratio)

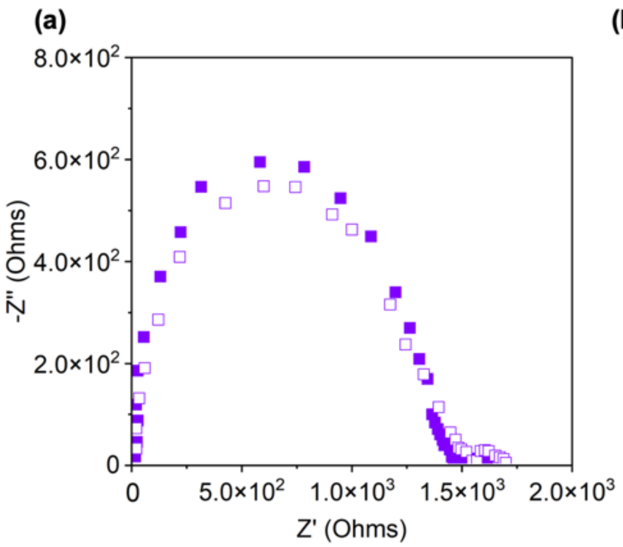

Figure S58. (a) Z'' as a function of Z' for lithium–lithium symmetric cell A before (filled squares) and after polarization (hollow squares). (b) Current *vs.* time during cell polarization.

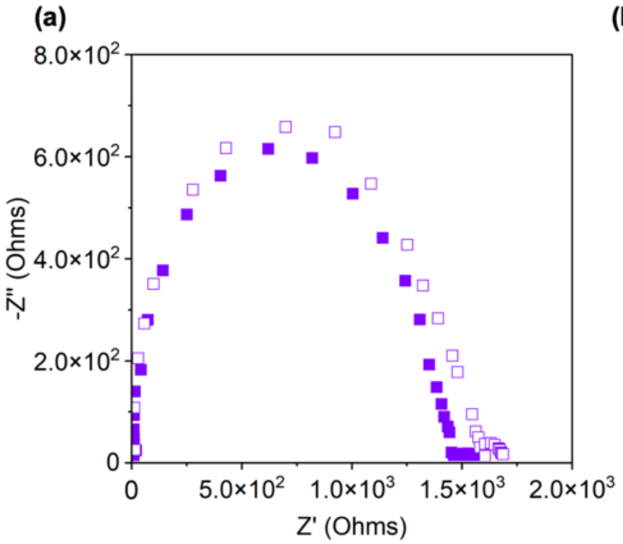

Figure S59. (a) Z'' as a function of Z' for lithium–lithium symmetric cell B before (filled squares) and after polarization (hollow squares). (b) Current *vs.* time during cell polarization.

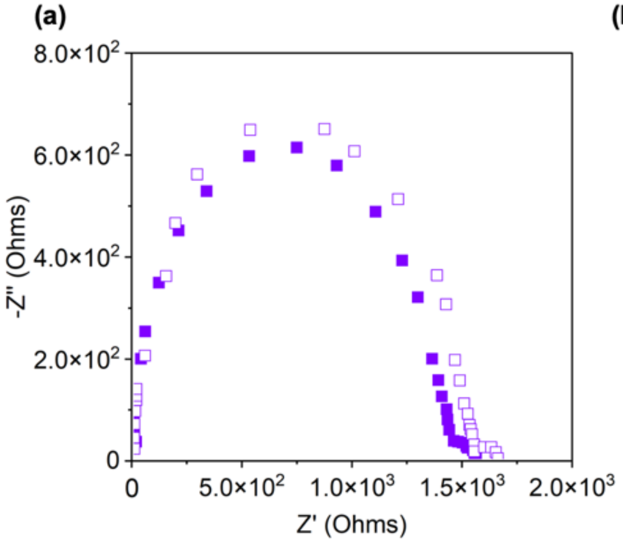

Figure S60. (a) Z'' as a function of Z' for lithium–lithium symmetric cell C before (filled squares) and after polarization (hollow squares). (b) Current *vs.* time during cell polarization.

8.1.5 POEM / PLiMTFSI / LiTFSI blend; [EO]:[LiMTFSI]:[LiTFSI] = 10:0.50:0.50 (molar ratio)

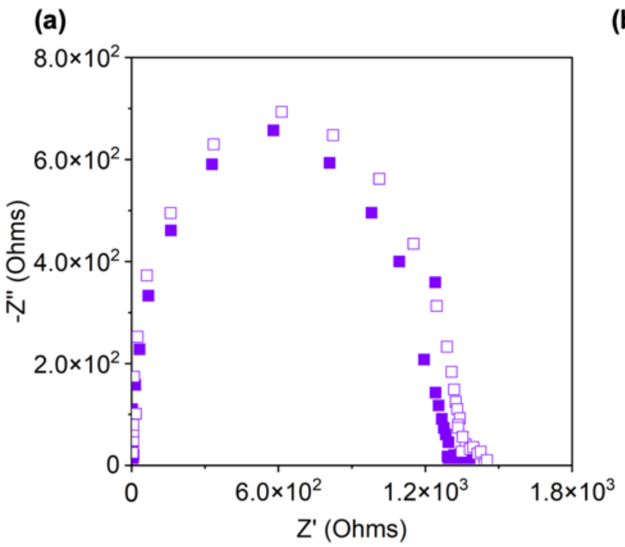

Figure S61. (a) Z'' as a function of Z' for lithium–lithium symmetric cell A before (filled squares) and after polarization (hollow squares). (b) Current *vs.* time during cell polarization.

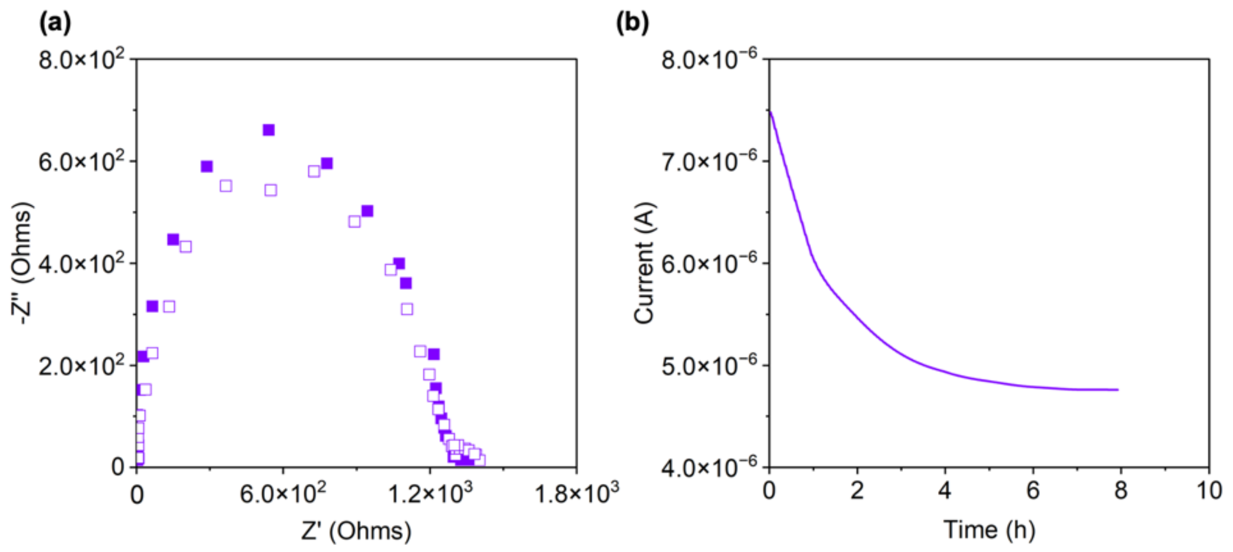

Figure S62. (a) Z'' as a function of Z' for lithium–lithium symmetric cell B before (filled squares) and after polarization (hollow squares). (b) Current *vs.* time during cell polarization.

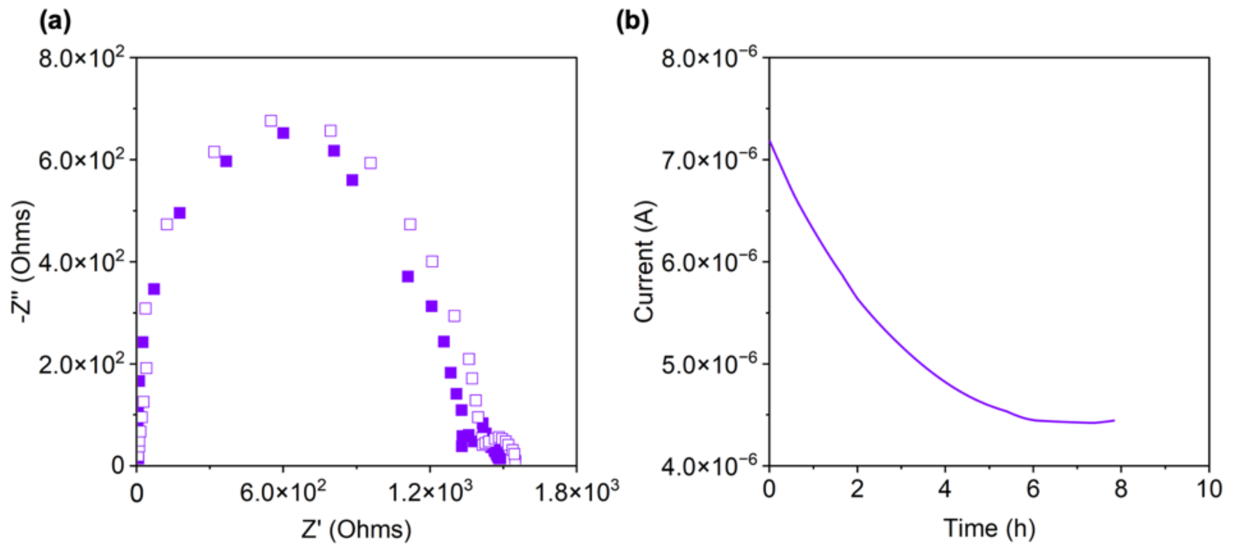

Figure S63. (a) Z'' as a function of Z' for lithium–lithium symmetric cell C before (filled squares) and after polarization (hollow squares). (b) Current *vs.* time during cell polarization.

8.1.6 POEM / PLiMTFSI / LiTFSI blend; [EO]:[LiMTFSI]:[LiTFSI] = 10:0.60:0.40 (molar ratio)

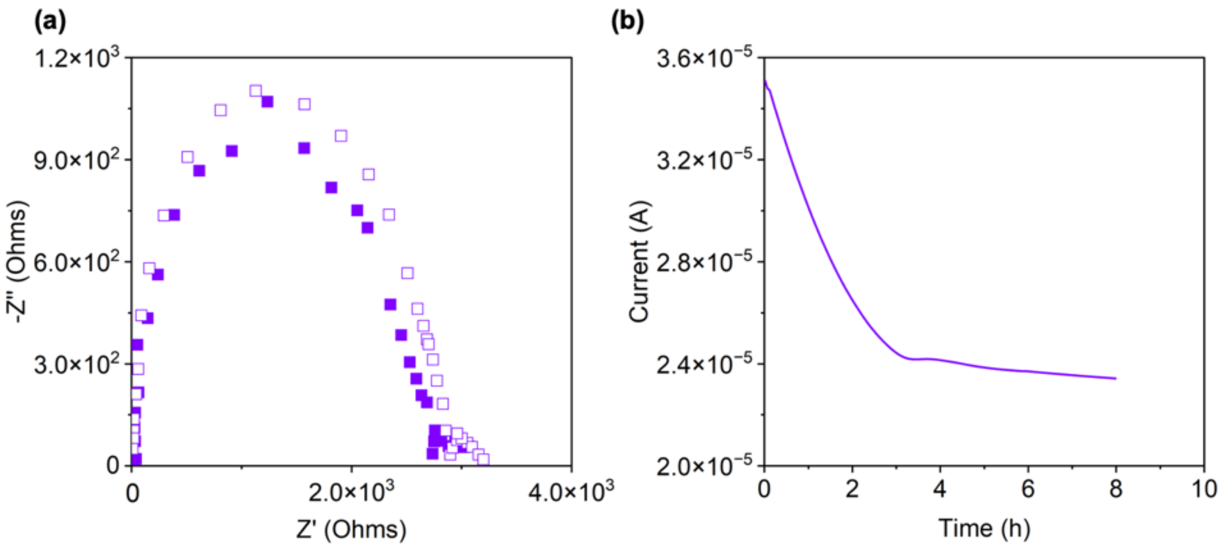

Figure S64. (a) Z'' as a function of Z' for lithium–lithium symmetric cell A before (filled squares) and after polarization (hollow squares). (b) Current *vs.* time during cell polarization.

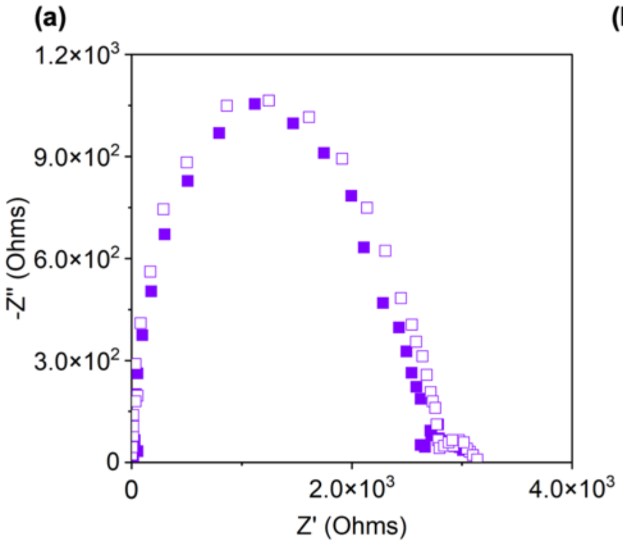

Figure S65. (a) Z'' as a function of Z' for lithium–lithium symmetric cell B before (filled squares) and after polarization (hollow squares). (b) Current *vs.* time during cell polarization.

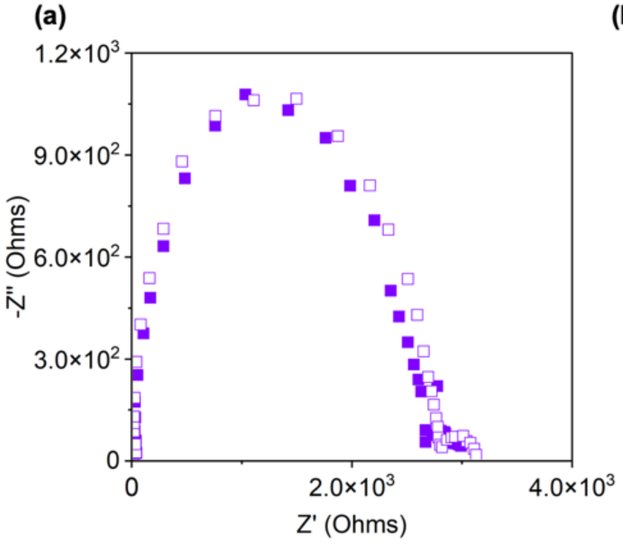

Figure S66. (a) Z'' as a function of Z' for lithium–lithium symmetric cell C before (filled squares) and after polarization (hollow squares). (b) Current *vs.* time during cell polarization.

8.1.7 POEM / PLiMTFSI / LiTFSI blend; [EO]:[LiMTFSI]:[LiTFSI] = 10:0.80:0.20 (molar ratio)

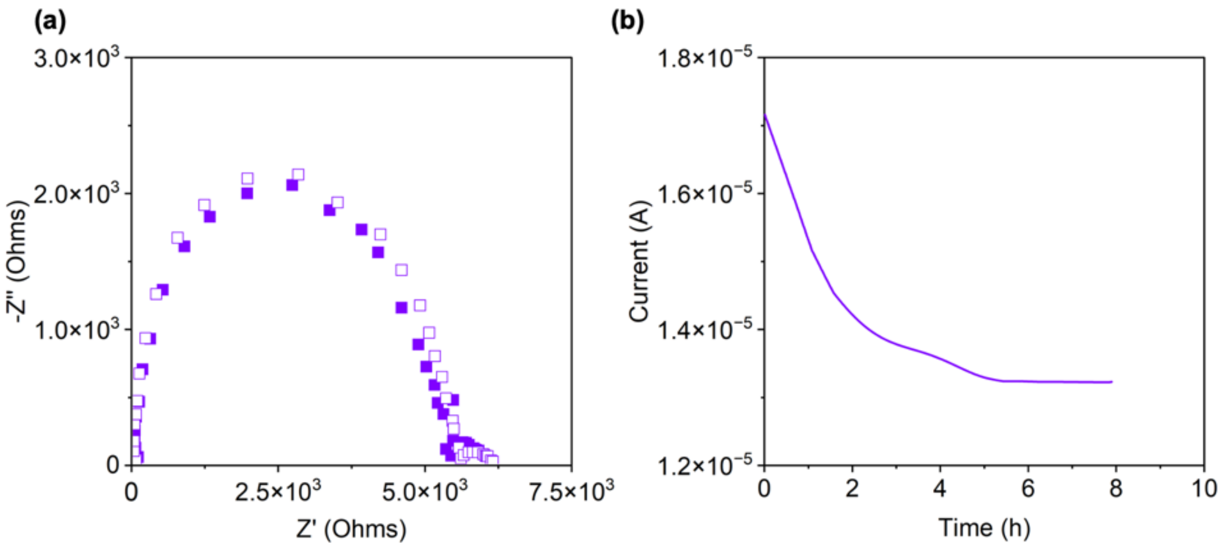

Figure S67. (a) Z'' as a function of Z' for lithium–lithium symmetric cell A before (filled squares) and after polarization (hollow squares). (b) Current *vs.* time during cell polarization.

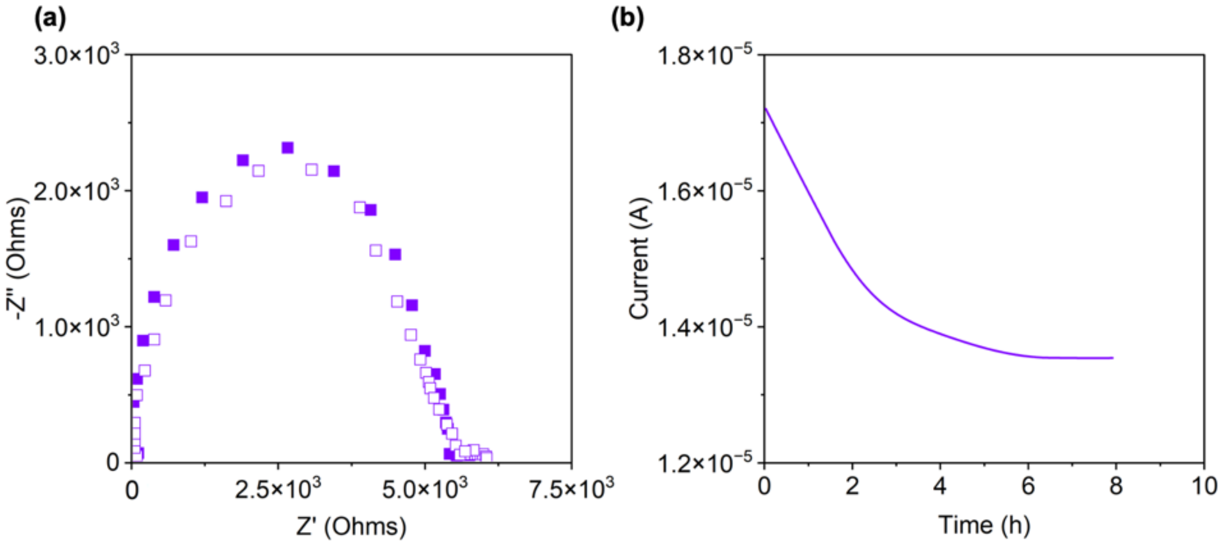

Figure S68. (a) Z'' as a function of Z' for lithium–lithium symmetric cell B before (filled squares) and after polarization (hollow squares). (b) Current *vs.* time during cell polarization.

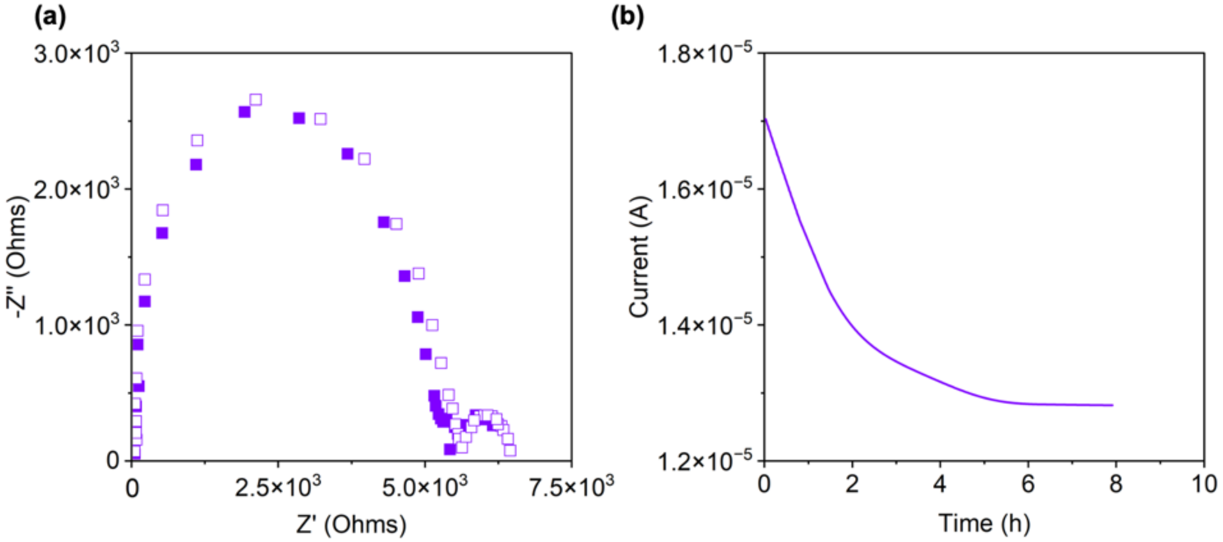

Figure S69. (a) Z'' as a function of Z' for lithium–lithium symmetric cell C before (filled squares) and after polarization (hollow squares). (b) Current *vs.* time during cell polarization.

8.1 Measurement conducted at 100 °C for POEM / PLiMTFSI / LiTFSI blends

8.2.1 POEM / PLiMTFSI / LiTFSI blend; [EO]:[LiMTFSI]:[LiTFSI] = 10:0.05:0.95 (molar ratio)

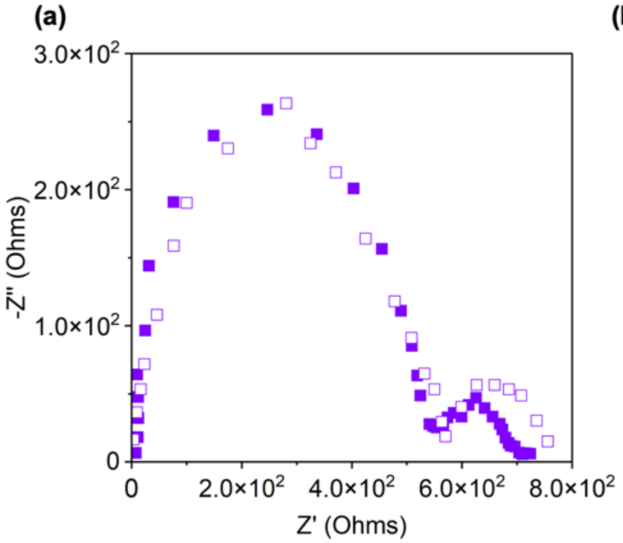

Figure S70. (a) Z″ as a function of Z′ for lithium–lithium symmetric cell A before (filled squares) and after polarization (hollow squares). (b) Current *vs.* time during cell polarization.

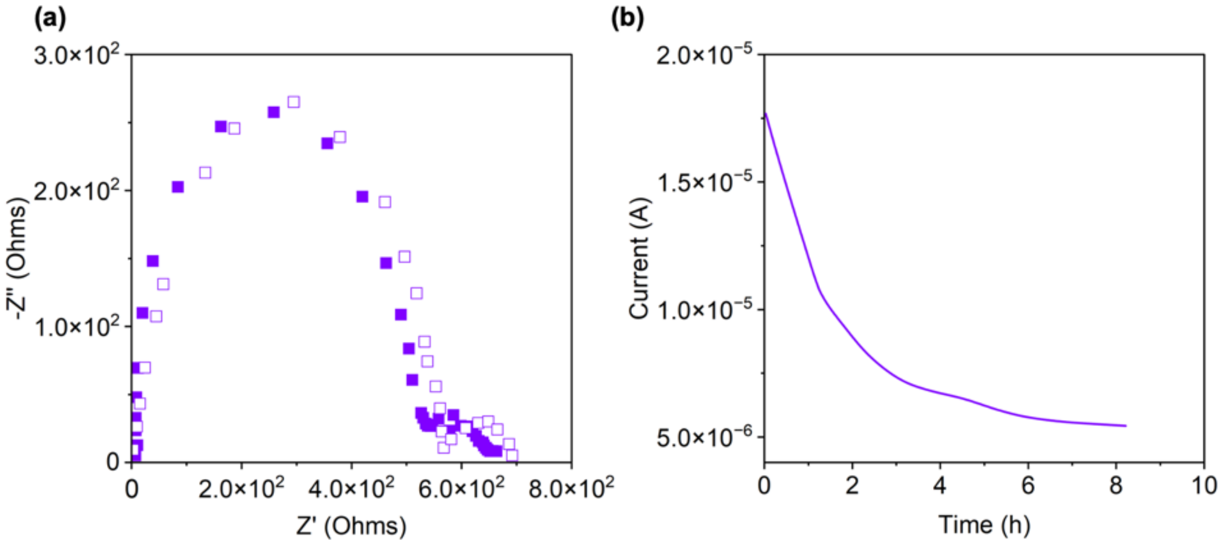

Figure S71. (a) Z'' as a function of Z' for lithium–lithium symmetric cell B before (filled squares) and after polarization (hollow squares). (b) Current *vs.* time during cell polarization.

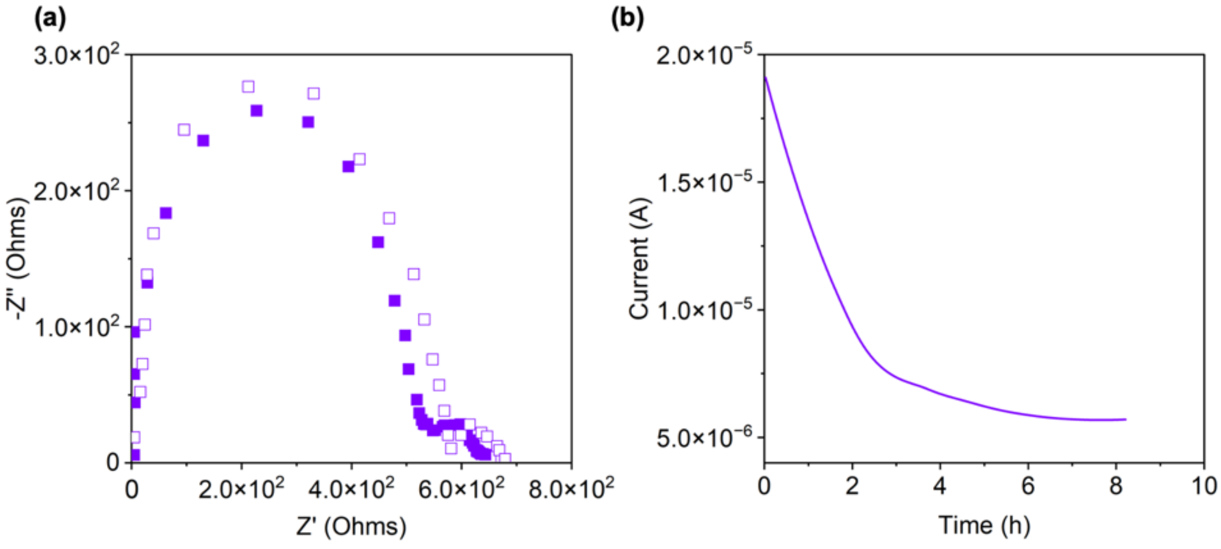

Figure S72. (a) Z'' as a function of Z' for lithium–lithium symmetric cell C before (filled squares) and after polarization (hollow squares). (b) Current *vs.* time during cell polarization.

8.2.2 POEM / PLiMTFSI / LiTFSI blend; [EO]:[LiMTFSI]:[LiTFSI] = 10:0.15:0.85 (molar ratio)

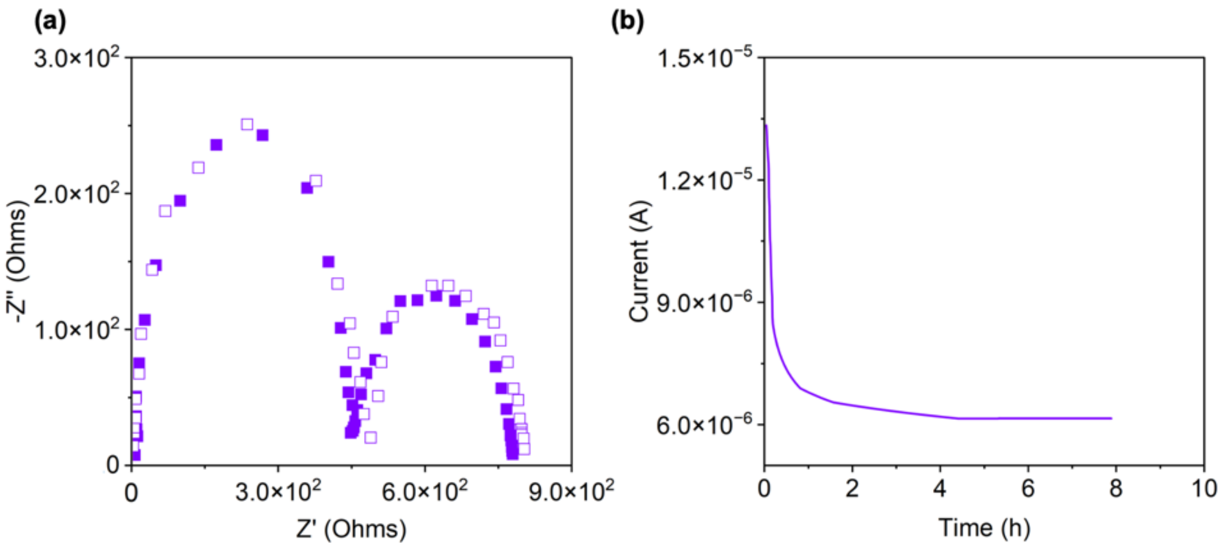

Figure S73. (a) Z'' as a function of Z' for lithium–lithium symmetric cell A before (filled squares) and after polarization (hollow squares). (b) Current *vs.* time during cell polarization.

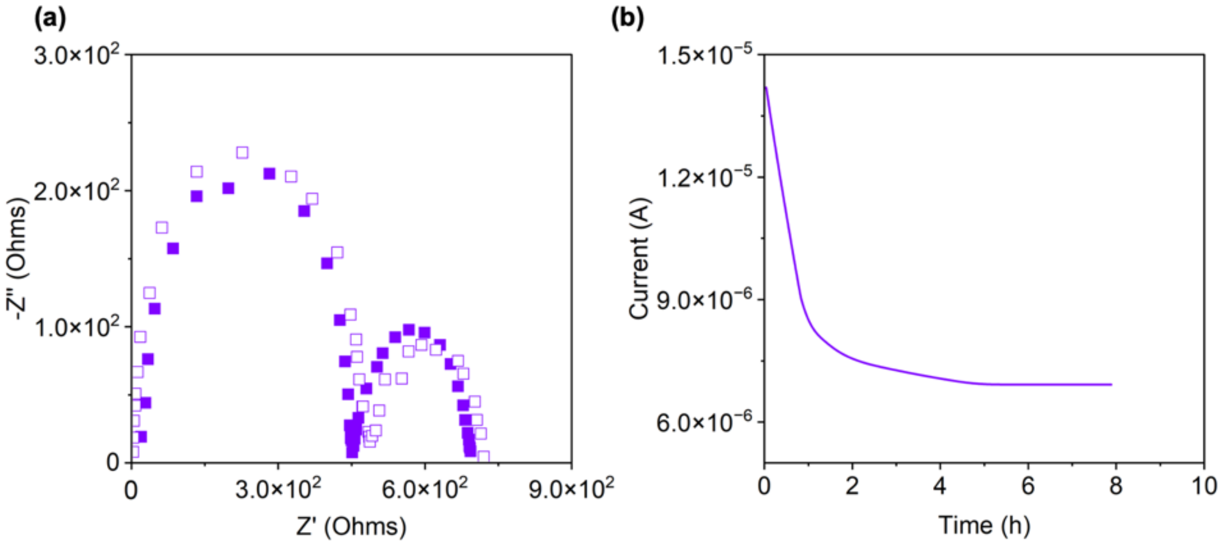

Figure S74. (a) Z'' as a function of Z' for lithium–lithium symmetric cell B before (filled squares) and after polarization (hollow squares). (b) Current *vs.* time during cell polarization.

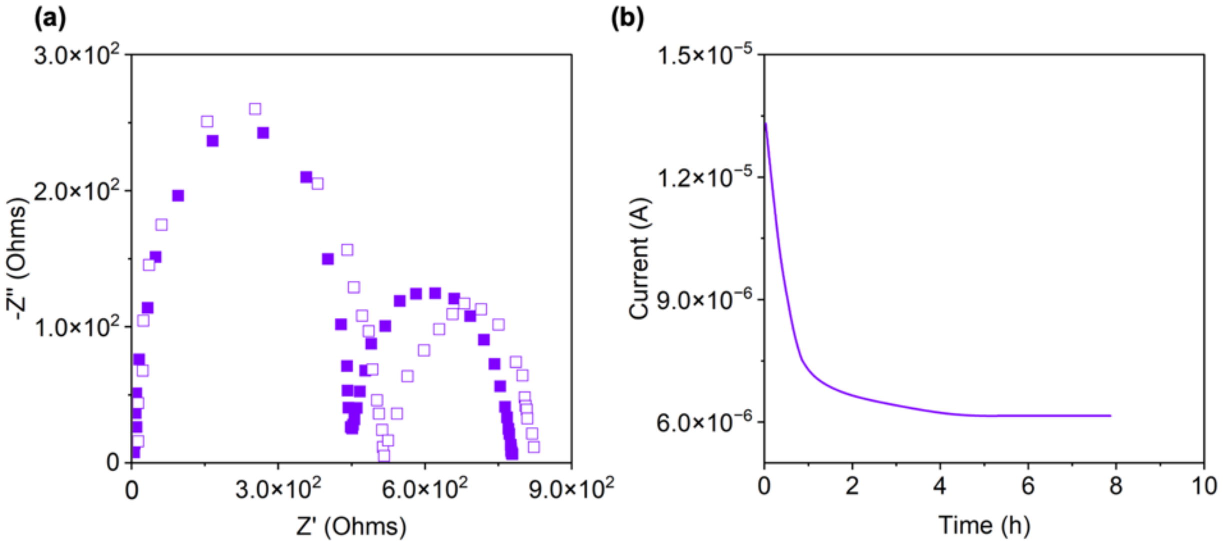

Figure S75. (a) Z'' as a function of Z' for lithium–lithium symmetric cell C before (filled squares) and after polarization (hollow squares). (b) Current *vs.* time during cell polarization.

8.2.3 POEM / PLiMTFSI / LiTFSI blend; [EO]:[LiMTFSI]:[LiTFSI] = 10:0.25:0.75 (molar ratio)

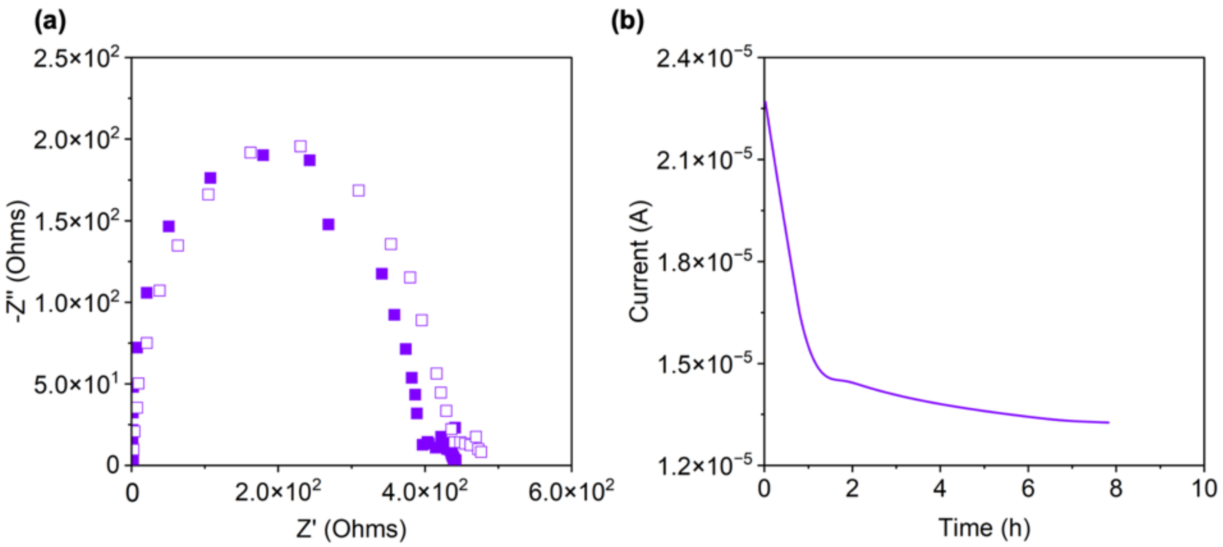

Figure S76. (a) Z'' as a function of Z' for lithium–lithium symmetric cell A before (filled squares) and after polarization (hollow squares). (b) Current *vs.* time during cell polarization.

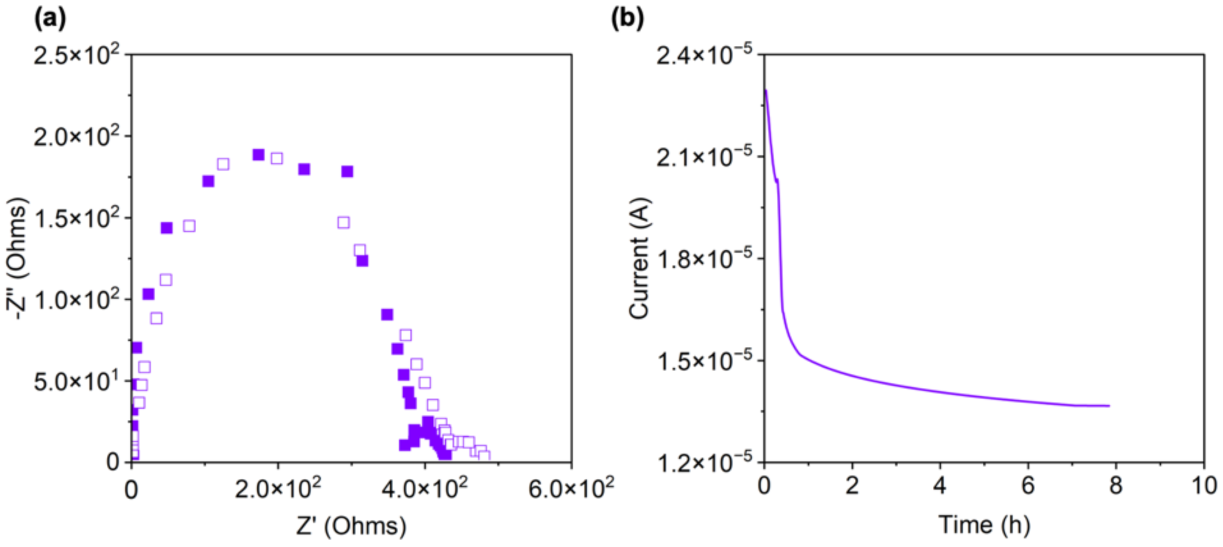

Figure S77. (a) Z'' as a function of Z' for lithium–lithium symmetric cell B before (filled squares) and after polarization (hollow squares). (b) Current *vs.* time during cell polarization.

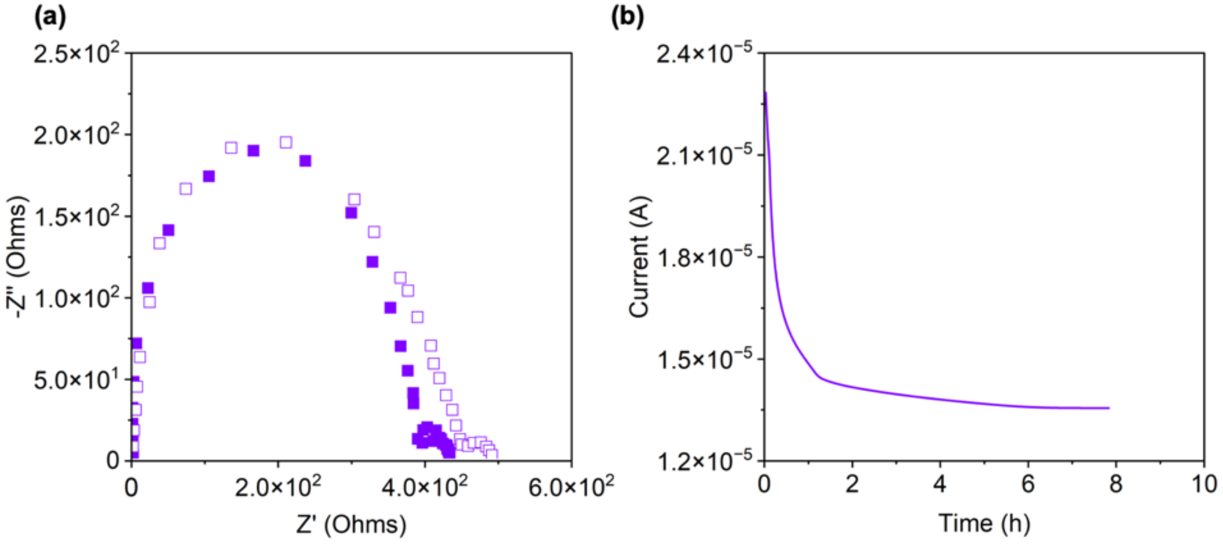

Figure S78. (a) Z'' as a function of Z' for lithium–lithium symmetric cell C before (filled squares) and after polarization (hollow squares). (b) Current *vs.* time during cell polarization.

8.2.4 POEM / PLiMTFSI / LiTFSI blend; [EO]:[LiMTFSI]:[LiTFSI] = 10:0.40:0.60 (molar ratio)

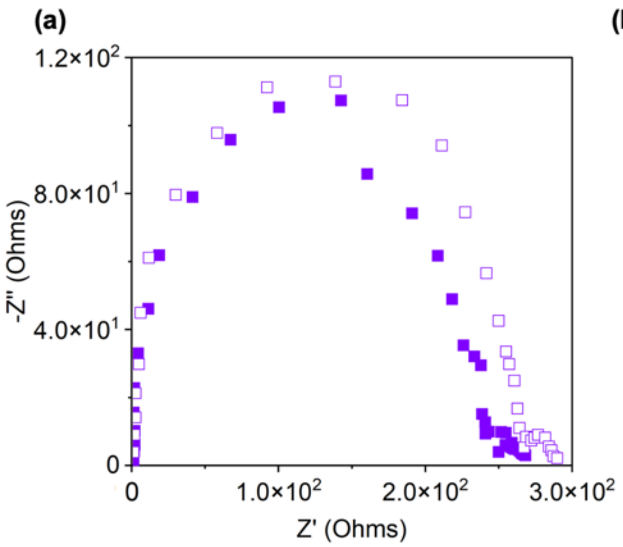

Figure S79. (a) Z'' as a function of Z' for lithium–lithium symmetric cell A before (filled squares) and after polarization (hollow squares). (b) Current *vs.* time during cell polarization.

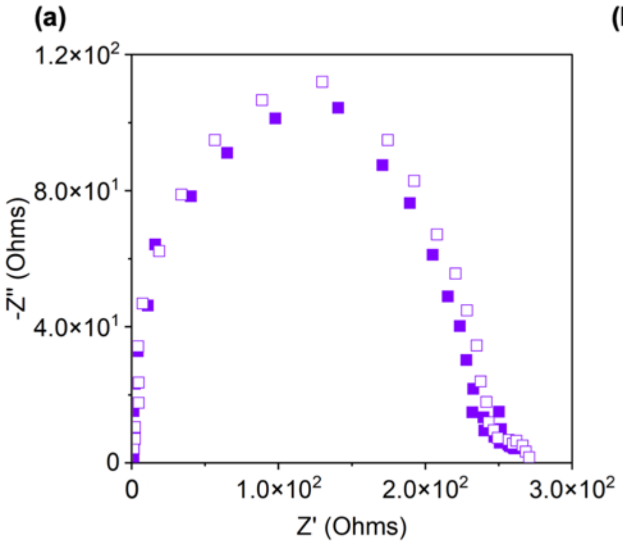

Figure S80. (a) Z'' as a function of Z' for lithium–lithium symmetric cell B before (filled squares) and after polarization (hollow squares). (b) Current *vs.* time during cell polarization.

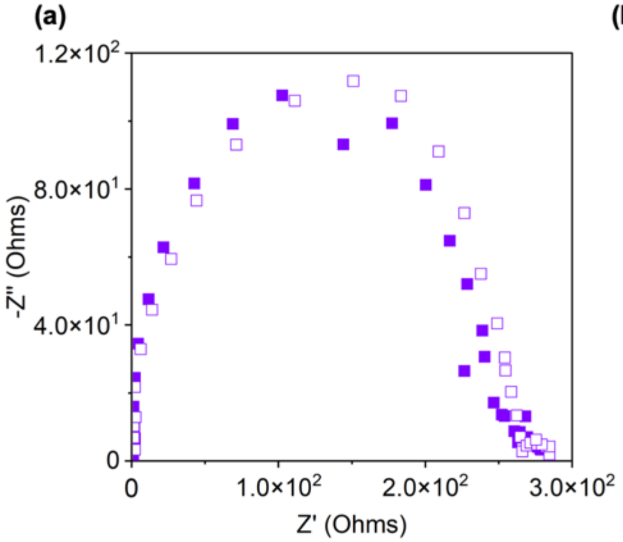

Figure S81. (a) Z'' as a function of Z' for lithium–lithium symmetric cell C before (filled squares) and after polarization (hollow squares). (b) Current *vs.* time during cell polarization.

8.2.5 POEM / PLiMTFSI / LiTFSI blend; [EO]:[LiMTFSI]:[LiTFSI] = 10:0.50:0.50 (molar ratio)

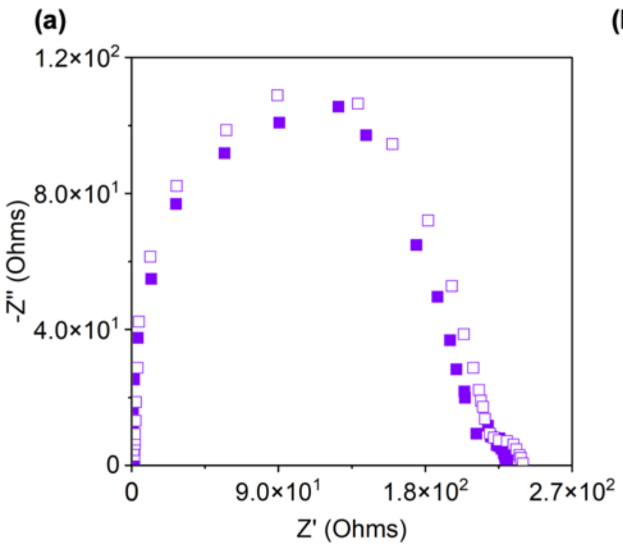

Figure S82. (a) Z'' as a function of Z' for lithium–lithium symmetric cell A before (filled squares) and after polarization (hollow squares). (b) Current *vs.* time during cell polarization.

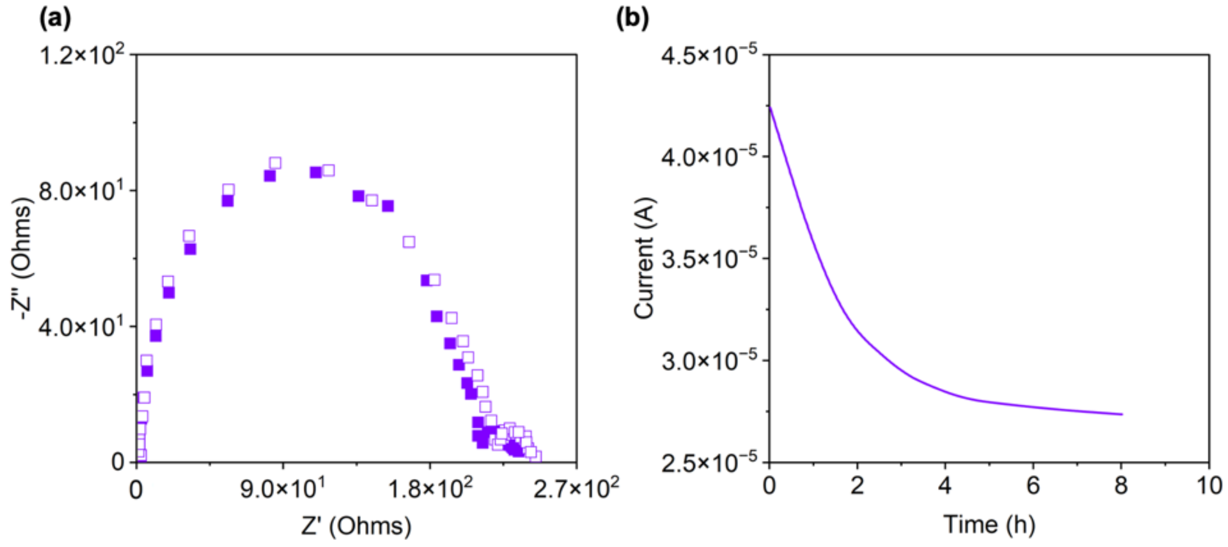

Figure S83. (a) Z'' as a function of Z' for lithium–lithium symmetric cell B before (filled squares) and after polarization (hollow squares). (b) Current *vs.* time during cell polarization.

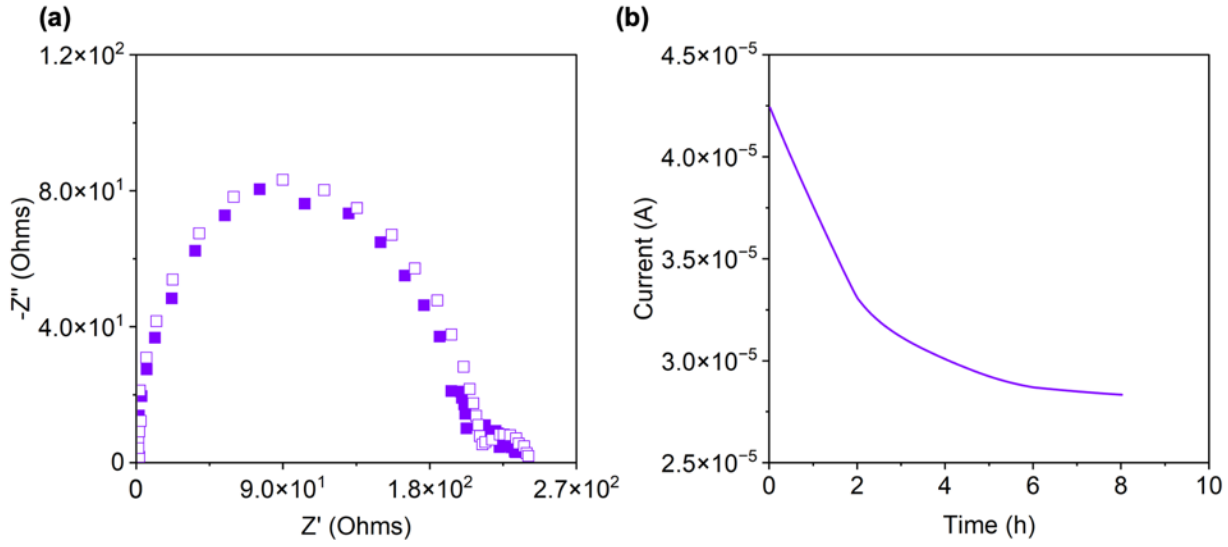

Figure S84. (a) Z'' as a function of Z' for lithium–lithium symmetric cell C before (filled squares) and after polarization (hollow squares). (b) Current *vs.* time during cell polarization.

8.2.6 POEM / PLiMTFSI / LiTFSI blend; [EO]:[LiMTFSI]:[LiTFSI] = 10:0.60:0.40 (molar ratio)

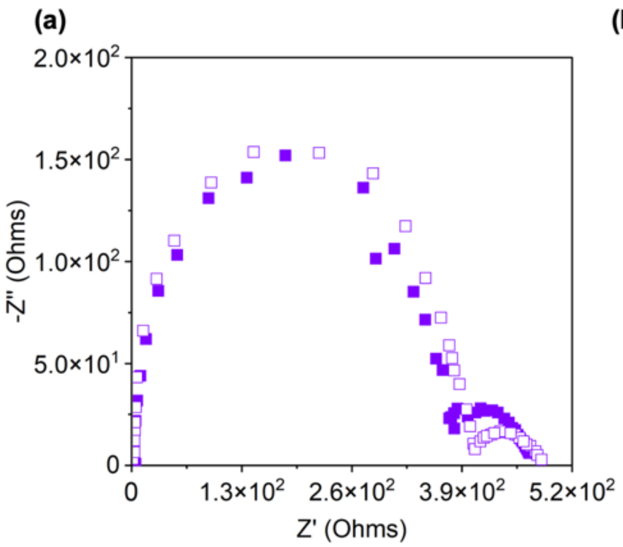

Figure S85. (a) Z'' as a function of Z' for lithium–lithium symmetric cell A before (filled squares) and after polarization (hollow squares). (b) Current *vs.* time during cell polarization.

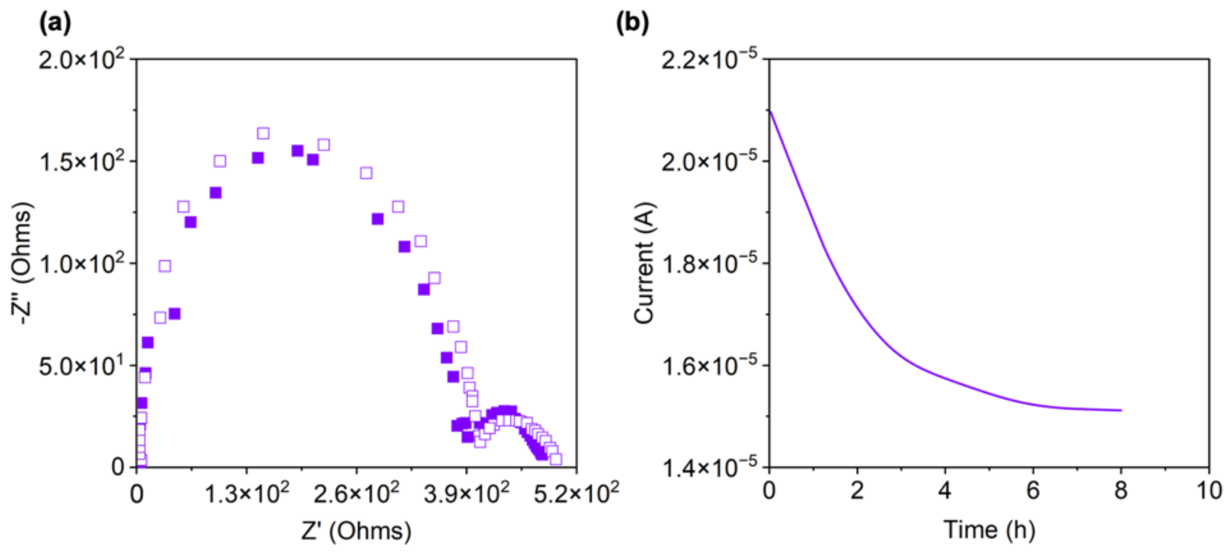

Figure S86. (a) Z'' as a function of Z' for lithium–lithium symmetric cell B before (filled squares) and after polarization (hollow squares). (b) Current *vs.* time during cell polarization.

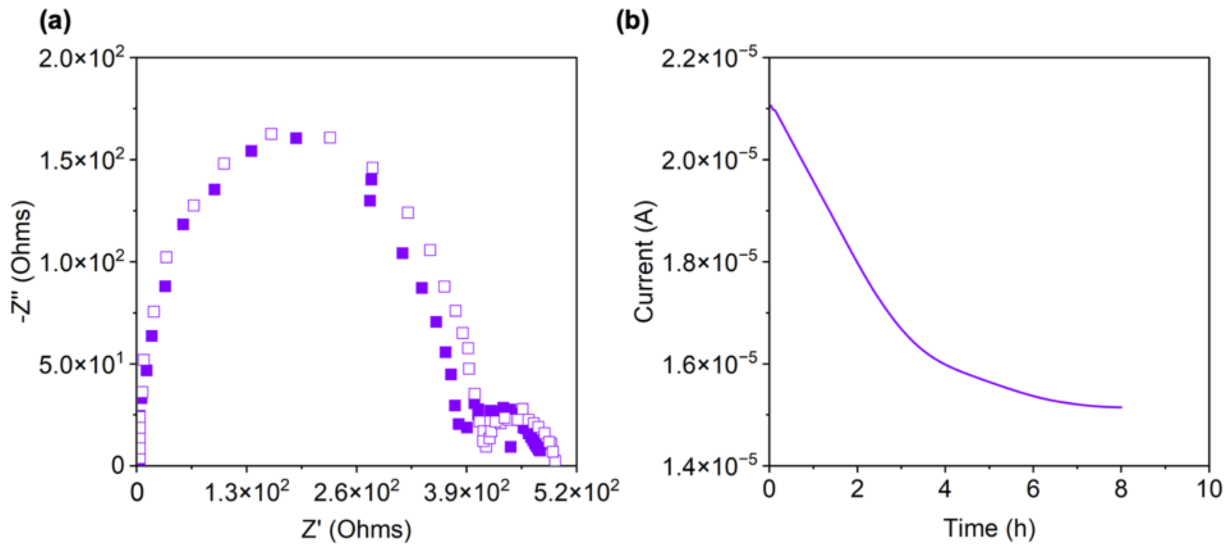

Figure S87. (a) Z'' as a function of Z' for lithium–lithium symmetric cell C before (filled squares) and after polarization (hollow squares). (b) Current *vs.* time during cell polarization.

8.2.7 POEM / PLiMTFSI / LiTFSI blend; [EO]:[LiMTFSI]:[LiTFSI] = 10:0.80:0.20 (molar ratio)

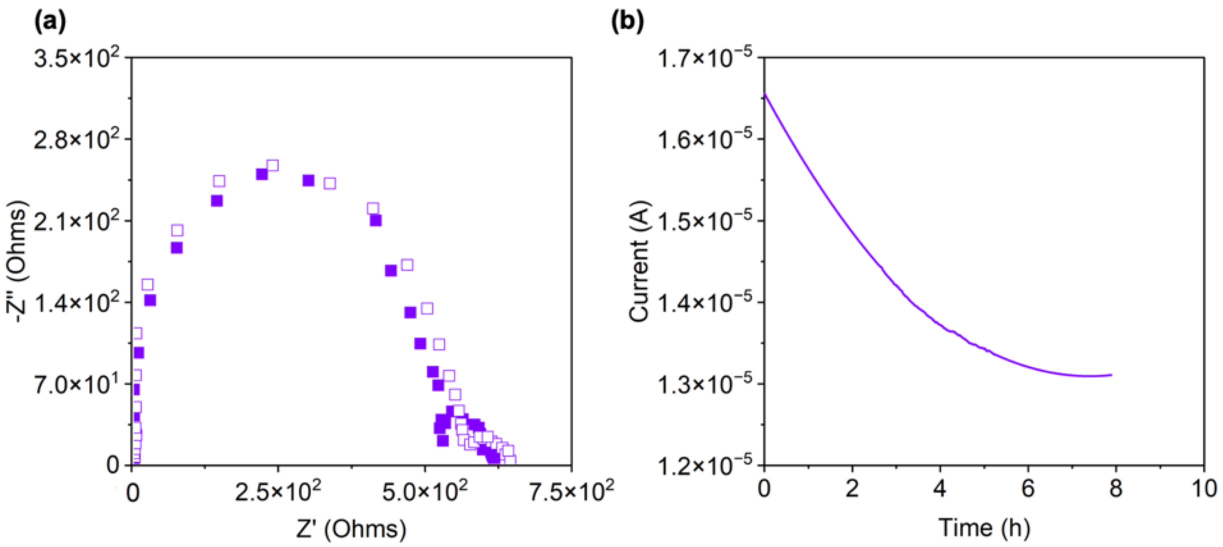

Figure S88. (a) Z'' as a function of Z' for lithium–lithium symmetric cell A before (filled squares) and after polarization (hollow squares). (b) Current *vs.* time during cell polarization.

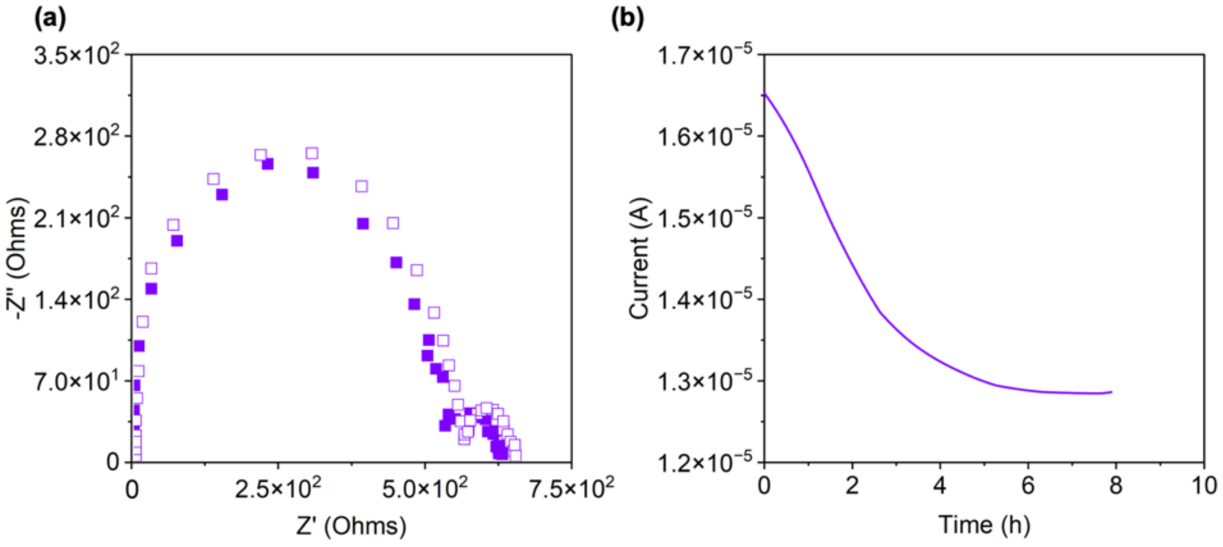

Figure S89. (a) Z'' as a function of Z' for lithium–lithium symmetric cell B before (filled squares) and after polarization (hollow squares). (b) Current *vs.* time during cell polarization.

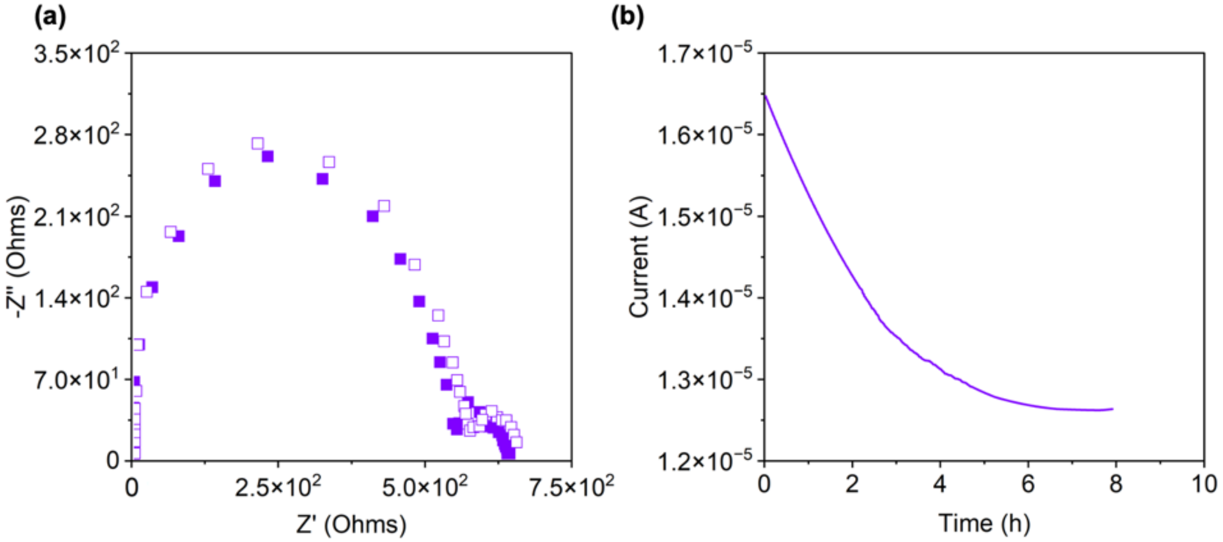

Figure S90. (a) Z'' as a function of Z' for lithium–lithium symmetric cell C before (filled squares) and after polarization (hollow squares). (b) Current *vs.* time during cell polarization.

Summary of the parameters for the Bruce-Vincent method for POEM / PLiMTFSI / LiFSI blends.

Table S11 (a). Impedance and current values extracted from AC impedance spectroscopy and potentiostatic polarization measurements of three lithium–lithium symmetric cells for POEM / PLiMTFSI / LiFSI blend; [EO]:[LiMTFSI]:[LiFSI] = 10:0.05:0.95 (molar ratio) at 60 °C.

	$^{b}R_1$ (Ohms)	$^{b}R_2$ (Ohms)	$^{c}R_1^*$ (Ohms)	$^{c}R_2^*$ (Ohms)	$^{d}R_0$ (Ohms)	$^{e}R_{SS}$ (Ohms)	$^{f}I_0$ (A)	I_{SS} (A)	t_{Li+}
^{a}Cell A	5440	8100	5450	8240	2660	2790	1.23×10^{-6}	3.70×10^{-7}	0.30
^{a}Cell B	5430	8160	5540	8430	2730	2890	1.23×10^{-6}	3.78×10^{-7}	0.31
^{a}Cell C	5580	8310	5610	8410	2730	2800	1.20×10^{-6}	3.61×10^{-7}	0.30

^{a}Cells A, B, and C were made with lithium non-blocking electrodes. $^{b}R_1$ and R_2 are the impedances of the minima at the bounds of the low-frequency semicircle in a Nyquist plot before cell polarization. $^{c}R_1$* and R_2* are the corresponding impedances after polarization. ^{d}Initial interfacial impedance was defined as $R_0 = R_2 - R_1$. ^{e}Steady-state interfacial impedance was defined as $R_{SS} = R_2^* - R_1^*$. ^{f}Initial current, I_0, was calculated by Ohm's law. This footnote applies to all tables in this section.

Table S11 (b). Impedance and current values extracted from AC impedance spectroscopy and potentiostatic polarization measurements of three lithium–lithium symmetric cells for POEM / PLiMTFSI / LiFSI blend; [EO]:[LiMTFSI]:[LiFSI] = 10:0.05:0.95 (molar ratio) at 100 °C.

	$^{b}R_1$ (Ohms)	$^{b}R_2$ (Ohms)	$^{c}R_1^*$ (Ohms)	$^{c}R_2^*$ (Ohms)	$^{d}R_0$ (Ohms)	$^{e}R_{SS}$ (Ohms)	$^{f}I_0$ (A)	I_{SS} (A)	t_{Li+}
^{a}Cell A	900	1150	930	1180	250	250	8.73×10^{-6}	2.88×10^{-6}	0.33
^{a}Cell B	910	1270	930	1290	360	360	7.87×10^{-6}	2.61×10^{-6}	0.33
^{a}Cell C	910	1320	960	1360	410	400	7.60×10^{-6}	2.51×10^{-6}	0.33

Table S12 (a). Impedance and current values extracted from AC impedance spectroscopy and potentiostatic polarization measurements of three lithium–lithium symmetric cells for POEM / PLiMTFSI / LiFSI blend; [EO]:[LiMTFSI]:[LiFSI] = 10:0.15:0.85 (molar ratio) at 60 °C.

	$^{b}R_1$ (Ohms)	$^{b}R_2$ (Ohms)	$^{c}R_1^*$ (Ohms)	$^{c}R_2^*$ (Ohms)	$^{d}R_0$ (Ohms)	$^{e}R_{SS}$ (Ohms)	$^{f}I_0$ (A)	I_{SS} (A)	t_{Li+}
^{a}Cell A	8800	1.21×10^{4}	8990	1.24×10^{4}	3280	3450	8.27×10^{-7}	3.70×10^{-7}	0.45
^{a}Cell B	8810	1.31×10^{4}	9020	1.34×10^{4}	4260	4380	7.65×10^{-7}	3.38×10^{-7}	0.44
^{a}Cell C	9060	1.14×10^{4}	9540	1.20×10^{4}	2340	2420	8.78×10^{-7}	3.86×10^{-7}	0.44

Table S12 (b). Impedance and current values extracted from AC impedance spectroscopy and potentiostatic polarization measurements of three lithium–lithium symmetric cells for POEM / PLiMTFSI / LiFSI blend; [EO]:[LiMTFSI]:[LiFSI] = 10:0.15:0.85 (molar ratio) at 100 °C.

	$^{b}R_1$ (Ohms)	$^{b}R_2$ (Ohms)	$^{c}R_1^*$ (Ohms)	$^{c}R_2^*$ (Ohms)	$^{d}R_0$ (Ohms)	$^{e}R_{SS}$ (Ohms)	$^{f}I_0$ (A)	I_{SS} (A)	t_{Li+}
^{a}Cell A	1000	1250	1020	1290	250	270	8.01×10^{-6}	3.69×10^{-6}	0.46
^{a}Cell B	1050	1340	1090	1400	290	310	7.46×10^{-6}	3.53×10^{-6}	0.47
^{a}Cell C	1020	1490	1060	1550	470	490	6.70×10^{-6}	3.13×10^{-6}	0.47

Table S13 (a). Impedance and current values extracted from AC impedance spectroscopy and potentiostatic polarization measurements of three lithium–lithium symmetric cells for POEM / PLiMTFSI / LiFSI blend; [EO]:[LiMTFSI]:[LiFSI] = 10:0.25:0.75 (molar ratio) at 60 °C.

	$^{b}R_1$ (Ohms)	$^{b}R_2$ (Ohms)	$^{c}R_1^*$ (Ohms)	$^{c}R_2^*$ (Ohms)	$^{d}R_0$ (Ohms)	$^{e}R_{SS}$ (Ohms)	$^{f}I_0$ (A)	I_{SS} (A)	t_{Li+}
^{a}Cell A	1.15×10^{4}	1.29×10^{4}	1.19×10^{4}	1.33×10^{4}	1400	1430	7.73×10^{-7}	4.11×10^{-7}	0.53
^{a}Cell B	1.15×10^{4}	1.28×10^{4}	1.18×10^{4}	1.33×10^{4}	1220	1470	7.84×10^{-7}	4.16×10^{-7}	0.53
^{a}Cell C	1.16×10^{4}	1.29×10^{4}	1.20×10^{4}	1.34×10^{4}	1260	1400	7.75×10^{-7}	4.11×10^{-7}	0.53

Table S13 (b). Impedance and current values extracted from AC impedance spectroscopy and potentiostatic polarization measurements of three lithium–lithium symmetric cells for POEM / PLiMTFSI / LiFSI blend; [EO]:[LiMTFSI]:[LiFSI] = 10:0.25:0.75 (molar ratio) at 100 °C.

	$^{b}R_1$ (Ohms)	$^{b}R_2$ (Ohms)	$^{c}R_1^{*}$ (Ohms)	$^{c}R_2^{*}$ (Ohms)	$^{d}R_0$ (Ohms)	$^{e}R_{SS}$ (Ohms)	$^{f}I_0$ (A)	I_{SS} (A)	t_{Li+}
^{a}Cell A	1080	1120	1100	1170	40	70	8.97×10^{-6}	5.11×10^{-6}	0.57
^{a}Cell B	1100	1200	1150	1250	100	100	8.37×10^{-6}	4.85×10^{-6}	0.58
^{a}Cell C	1130	1230	1160	1270	100	110	8.13×10^{-6}	4.63×10^{-6}	0.57

Table S14 (a). Impedance and current values extracted from AC impedance spectroscopy and potentiostatic polarization measurements of three lithium–lithium symmetric cells for POEM / PLiMTFSI / LiFSI blend; [EO]:[LiMTFSI]:[LiFSI] = 10:0.40:0.60 (molar ratio) at 60 °C.

	$^{b}R_1$ (Ohms)	$^{b}R_2$ (Ohms)	$^{c}R_1^{*}$ (Ohms)	$^{c}R_2^{*}$ (Ohms)	$^{d}R_0$ (Ohms)	$^{e}R_{SS}$ (Ohms)	$^{f}I_0$ (A)	I_{SS} (A)	t_{Li+}
^{a}Cell A	1.60×10^{4}	1.93×10^{4}	1.64×10^{4}	2.05×10^{4}	3310	4090	5.18×10^{-7}	3.18×10^{-7}	0.61
^{a}Cell B	1.61×10^{4}	1.95×10^{4}	1.69×10^{4}	2.05×10^{4}	3440	3620	5.12×10^{-7}	3.19×10^{-7}	0.61
^{a}Cell C	1.62×10^{4}	1.95×10^{4}	1.74×10^{4}	2.12×10^{4}	3370	3860	5.12×10^{-7}	3.21×10^{-7}	0.62

Table S14 (b). Impedance and current values extracted from AC impedance spectroscopy and potentiostatic polarization measurements of three lithium–lithium symmetric cells for POEM / PLiMTFSI / LiFSI blend; [EO]:[LiMTFSI]:[LiFSI] = 10:0.40:0.60 (molar ratio) at 100 °C.

	$^{b}R_1$ (Ohms)	$^{b}R_2$ (Ohms)	$^{c}R_1^{*}$ (Ohms)	$^{c}R_2^{*}$ (Ohms)	$^{d}R_0$ (Ohms)	$^{e}R_{SS}$ (Ohms)	$^{f}I_0$ (A)	I_{SS} (A)	t_{Li+}
^{a}Cell A	1580	1920	1650	2030	340	380	5.21×10^{-6}	3.18×10^{-6}	0.61
^{a}Cell B	1530	1860	1570	1920	330	350	5.38×10^{-6}	3.28×10^{-6}	0.61
^{a}Cell C	1560	1890	1620	1950	330	330	5.31×10^{-6}	3.24×10^{-6}	0.61

Table S15 (a). Impedance and current values extracted from AC impedance spectroscopy and potentiostatic polarization measurements of three lithium–lithium symmetric cells for POEM / PLiMTFSI / LiFSI blend; [EO]:[LiMTFSI]:[LiFSI] = 10:0.50:0.50 (molar ratio) at 60 °C.

	$^{b}R_{1}$ (Ohms)	$^{b}R_{2}$ (Ohms)	$^{c}R_{1}^{*}$ (Ohms)	$^{c}R_{2}^{*}$ (Ohms)	$^{d}R_{0}$ (Ohms)	$^{e}R_{SS}$ (Ohms)	$^{f}I_{0}$ (A)	I_{SS} (A)	t_{Li+}
^{a}Cell A	1.45×10^{4}	1.49×10^{4}	1.52×10^{4}	1.57×10^{4}	360	560	6.72×10^{-7}	4.37×10^{-7}	0.65
^{a}Cell B	1.46×10^{4}	1.51×10^{4}	1.53×10^{4}	1.58×10^{4}	490	470	6.63×10^{-7}	4.38×10^{-7}	0.66
^{a}Cell C	1.45×10^{4}	1.57×10^{4}	1.50×10^{4}	1.69×10^{4}	1240	1880	6.36×10^{-7}	4.13×10^{-7}	0.65

Table S15 (b). Impedance and current values extracted from AC impedance spectroscopy and potentiostatic polarization measurements of three lithium–lithium symmetric cells for POEM / PLiMTFSI / LiFSI blend; [EO]:[LiMTFSI]:[LiFSI] = 10:0.50:0.50 (molar ratio) at 100 °C.

	$^{b}R_{1}$ (Ohms)	$^{b}R_{2}$ (Ohms)	$^{c}R_{1}^{*}$ (Ohms)	$^{c}R_{2}^{*}$ (Ohms)	$^{d}R_{0}$ (Ohms)	$^{e}R_{SS}$ (Ohms)	$^{f}I_{0}$ (A)	I_{SS} (A)	t_{Li+}
^{a}Cell A	1570	1690	1550	1730	120	180	5.93×10^{-6}	3.91×10^{-6}	0.66
^{a}Cell B	1550	1680	1620	1760	130	140	5.95×10^{-6}	3.87×10^{-6}	0.65
^{a}Cell C	1550	1680	1590	1740	130	150	5.96×10^{-6}	3.94×10^{-6}	0.66

Table S16 (a). Impedance and current values extracted from AC impedance spectroscopy and potentiostatic polarization measurements of three lithium–lithium symmetric cells for POEM / PLiMTFSI / LiFSI blend; [EO]:[LiMTFSI]:[LiFSI] = 10:0.60:0.40 (molar ratio) at 60 °C.

	$^{b}R_{1}$ (Ohms)	$^{b}R_{2}$ (Ohms)	$^{c}R_{1}^{*}$ (Ohms)	$^{c}R_{2}^{*}$ (Ohms)	$^{d}R_{0}$ (Ohms)	$^{e}R_{SS}$ (Ohms)	$^{f}I_{0}$ (A)	I_{SS} (A)	t_{Li+}
^{a}Cell A	1.38×10^{4}	1.50×10^{4}	1.40×10^{4}	1.57×10^{4}	1190	1700	6.68×10^{-7}	4.37×10^{-7}	0.65
^{a}Cell B	1.32×10^{4}	1.53×10^{4}	1.33×10^{4}	1.61×10^{4}	2060	2750	6.55×10^{-7}	4.30×10^{-7}	0.66
^{a}Cell C	1.36×10^{4}	1.57×10^{4}	1.38×10^{4}	1.61×10^{4}	2110	2380	6.39×10^{-7}	4.15×10^{-7}	0.65

Table S16 (b). Impedance and current values extracted from AC impedance spectroscopy and potentiostatic polarization measurements of three lithium–lithium symmetric cells for POEM / PLiMTFSI / LiFSI blend; [EO]:[LiMTFSI]:[LiFSI] = 10:0.60:0.40 (molar ratio) at 100 °C.

	${}^{b}R_1$ (Ohms)	${}^{b}R_2$ (Ohms)	${}^{c}R_1^*$ (Ohms)	${}^{c}R_2^*$ (Ohms)	${}^{d}R_0$ (Ohms)	${}^{e}R_{SS}$ (Ohms)	${}^{f}I_0$ (A)	I_{SS} (A)	t_{Li+}
^{a}Cell A	1960	2280	2010	2350	320	340	4.38×10^{-6}	2.90×10^{-6}	0.66
^{a}Cell B	2100	2400	2120	2480	300	360	4.16×10^{-6}	2.70×10^{-6}	0.65
^{a}Cell C	2150	2400	2140	2500	250	360	4.17×10^{-6}	2.75×10^{-6}	0.66

Table S17 (a). Impedance and current values extracted from AC impedance spectroscopy and potentiostatic polarization measurements of three lithium–lithium symmetric cells for POEM / PLiMTFSI / LiFSI blend; [EO]:[LiMTFSI]:[LiFSI] = 10:0.80:0.20 (molar ratio) at 60 °C.

	${}^{b}R_1$ (Ohms)	${}^{b}R_2$ (Ohms)	${}^{c}R_1^*$ (Ohms)	${}^{c}R_2^*$ (Ohms)	${}^{d}R_0$ (Ohms)	${}^{e}R_{SS}$ (Ohms)	${}^{f}I_0$ (A)	I_{SS} (A)	t_{Li+}
^{a}Cell A	4.01×10^{4}	4.24×10^{4}	3.99×10^{4}	4.37×10^{4}	2290	3830	2.36×10^{-7}	1.84×10^{-7}	0.78
^{a}Cell B	4.03×10^{4}	4.33×10^{4}	4.12×10^{4}	4.58×10^{4}	3090	4580	2.31×10^{-7}	1.82×10^{-7}	0.79
^{a}Cell C	3.90×10^{4}	4.64×10^{4}	4.09×10^{4}	4.58×10^{4}	7400	4990	2.15×10^{-7}	1.68×10^{-7}	0.78

Table S17 (b). Impedance and current values extracted from AC impedance spectroscopy and potentiostatic polarization measurements of three lithium–lithium symmetric cells for POEM / PLiMTFSI / LiFSI blend; [EO]:[LiMTFSI]:[LiFSI] = 10:0.80:0.20 (molar ratio) at 100 °C.

	${}^{b}R_1$ (Ohms)	${}^{b}R_2$ (Ohms)	${}^{c}R_1^*$ (Ohms)	${}^{c}R_2^*$ (Ohms)	${}^{d}R_0$ (Ohms)	${}^{e}R_{SS}$ (Ohms)	${}^{f}I_0$ (A)	I_{SS} (A)	t_{Li+}
^{a}Cell A	2920	3400	3010	3420	480	410	2.94×10^{-6}	2.32×10^{-6}	0.79
^{a}Cell B	2910	3280	3040	3460	370	420	3.05×10^{-6}	2.41×10^{-6}	0.79
^{a}Cell C	2930	3460	3050	3650	530	600	2.89×10^{-6}	2.31×10^{-6}	0.80

8.3 Measurement conducted at 60 °C for POEM / PLiMTFSI / LiFSI blends

8.3.1 POEM / PLiMTFSI / LiFSI blend; [EO]:[LiMTFSI]:[LiFSI] = 10:0.05:0.95 (molar ratio)

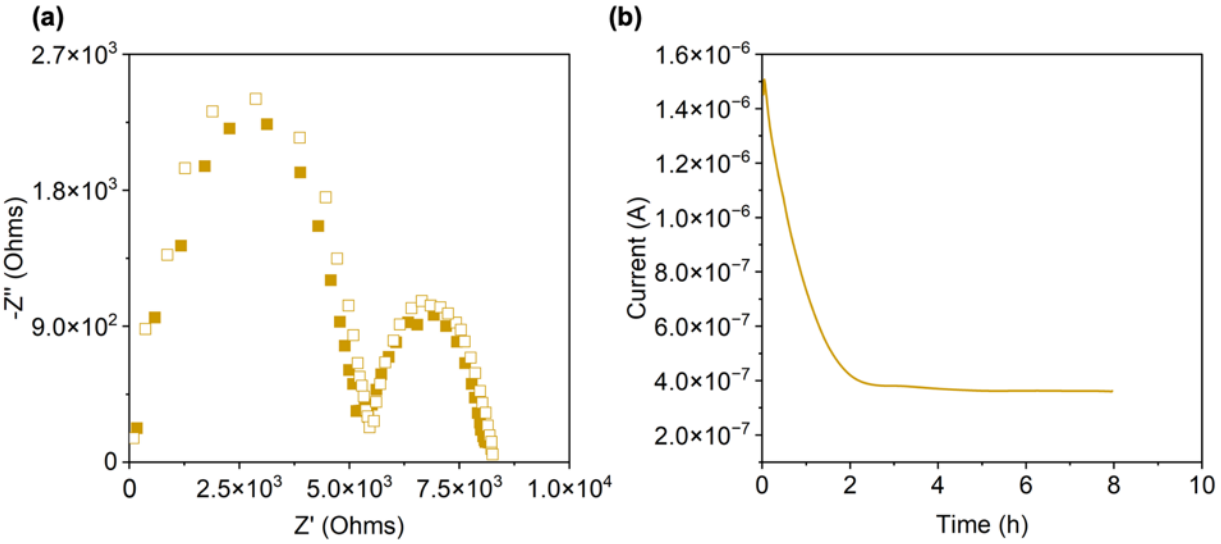

Figure S91. (a) Z'' as a function of Z' for lithium–lithium symmetric cell A before (filled squares) and after polarization (hollow squares). (b) Current *vs.* time during cell polarization.

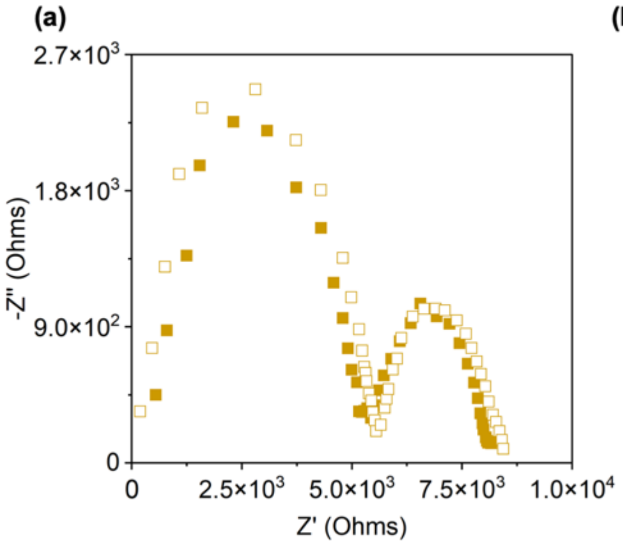

Figure S92. (a) Z'' as a function of Z' for lithium–lithium symmetric cell B before (filled squares) and after polarization (hollow squares). (b) Current *vs.* time during cell polarization.

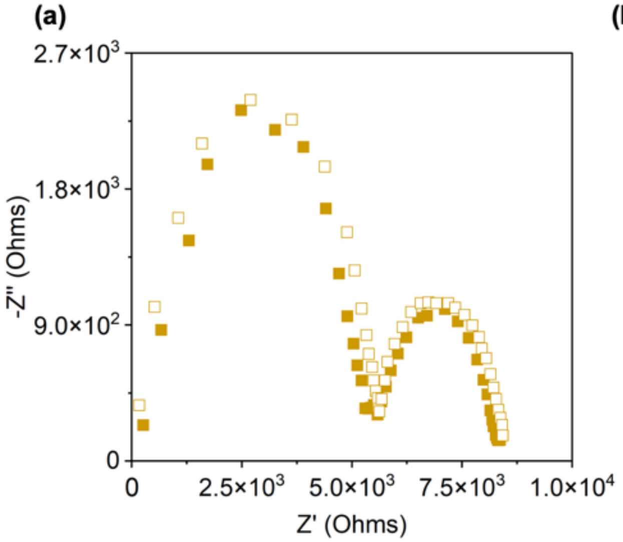

Figure S93. (a) Z'' as a function of Z' for lithium–lithium symmetric cell C before (filled squares) and after polarization (hollow squares). (b) Current *vs.* time during cell polarization.

8.3.2 POEM / PLiMTFSI / LiFSI blend; [EO]:[LiMTFSI]:[LiFSI] = 10:0.15:0.85 (molar ratio)

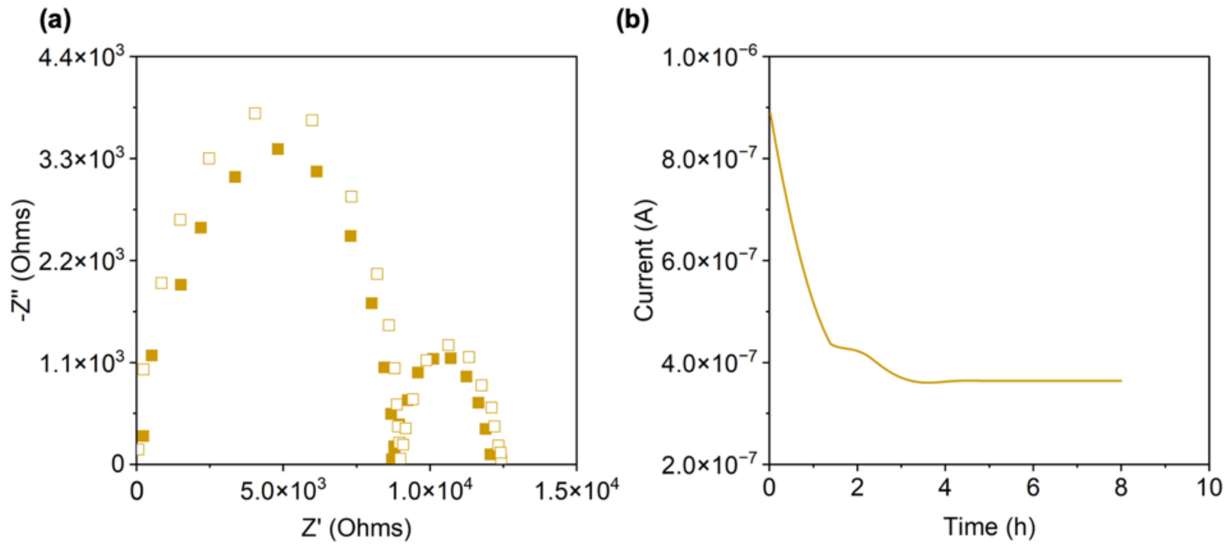

Figure S94. (a) Z'' as a function of Z' for lithium–lithium symmetric cell A before (filled squares) and after polarization (hollow squares). (b) Current *vs.* time during cell polarization.

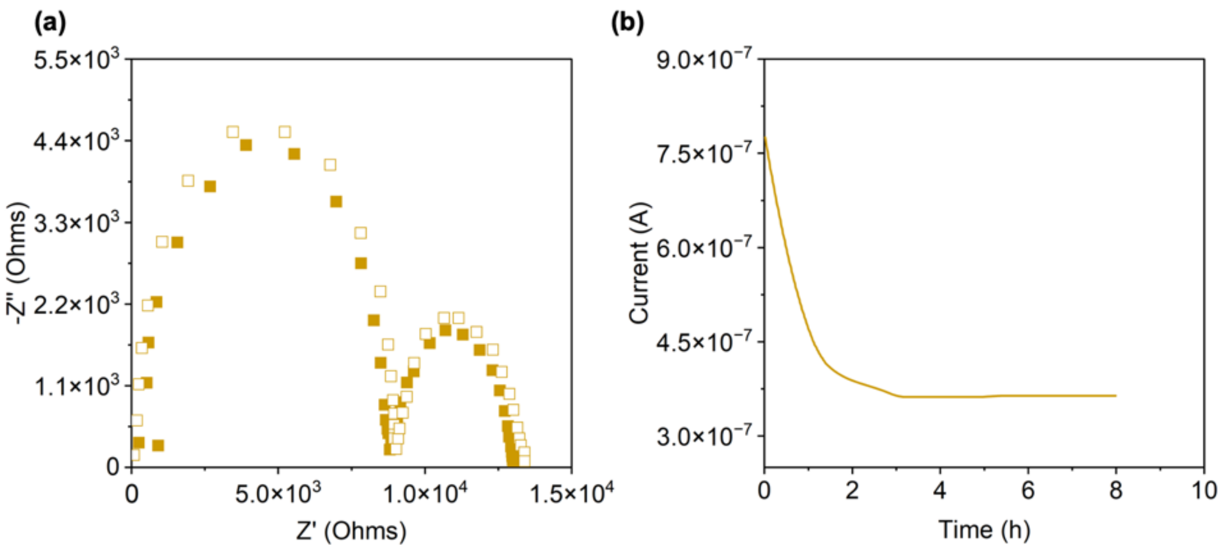

Figure S95. (a) Z'' as a function of Z' for lithium–lithium symmetric cell B before (filled squares) and after polarization (hollow squares). (b) Current *vs.* time during cell polarization.

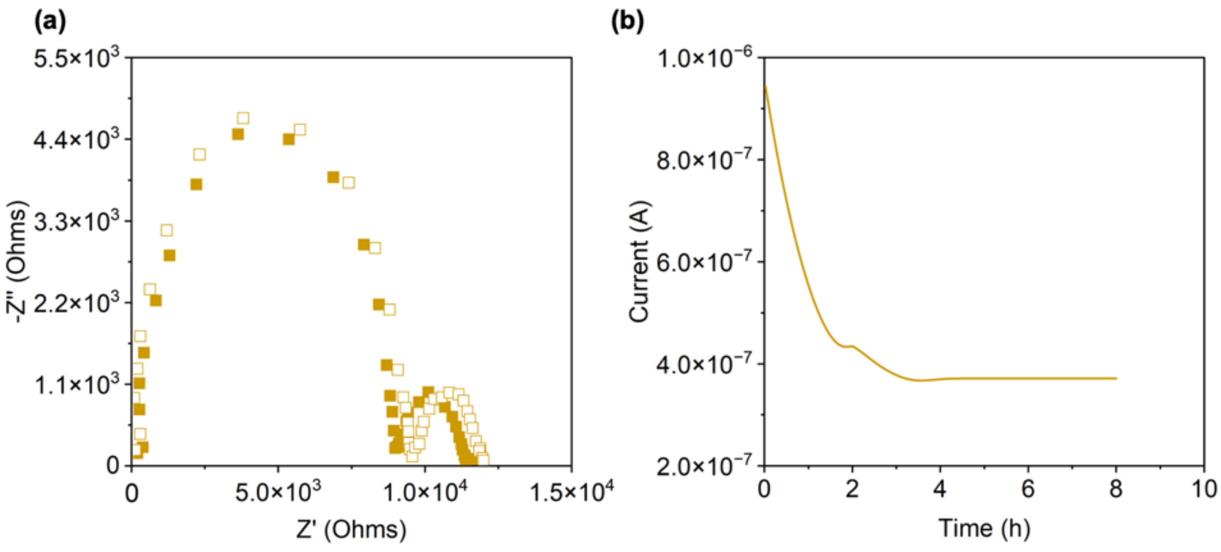

Figure S96. (a) Z'' as a function of Z' for lithium–lithium symmetric cell C before (filled squares) and after polarization (hollow squares). (b) Current *vs.* time during cell polarization.

8.3.3 POEM / PLiMTFSI / LiFSI blend; [EO]:[LiMTFSI]:[LiFSI] = 10:0.25:0.75 (molar ratio)

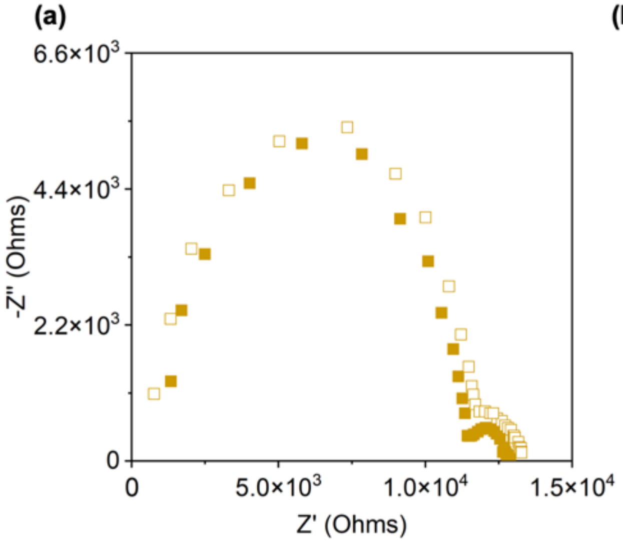

Figure S97. (a) Z'' as a function of Z' for lithium–lithium symmetric cell A before (filled squares) and after polarization (hollow squares). (b) Current *vs.* time during cell polarization.

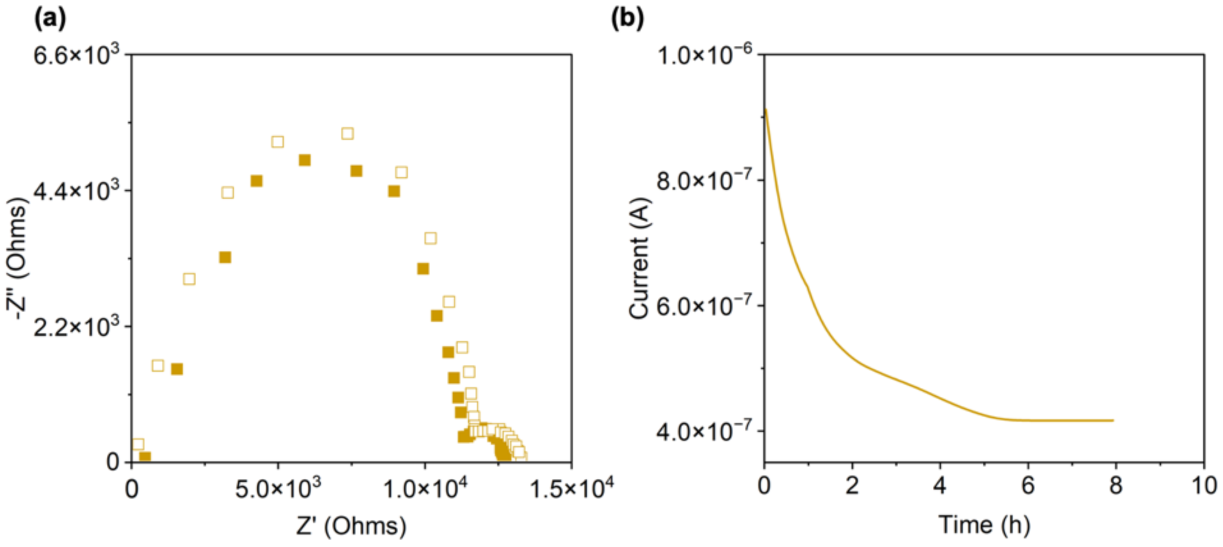

Figure S98. (a) Z'' as a function of Z' for lithium–lithium symmetric cell B before (filled squares) and after polarization (hollow squares). (b) Current *vs.* time during cell polarization.

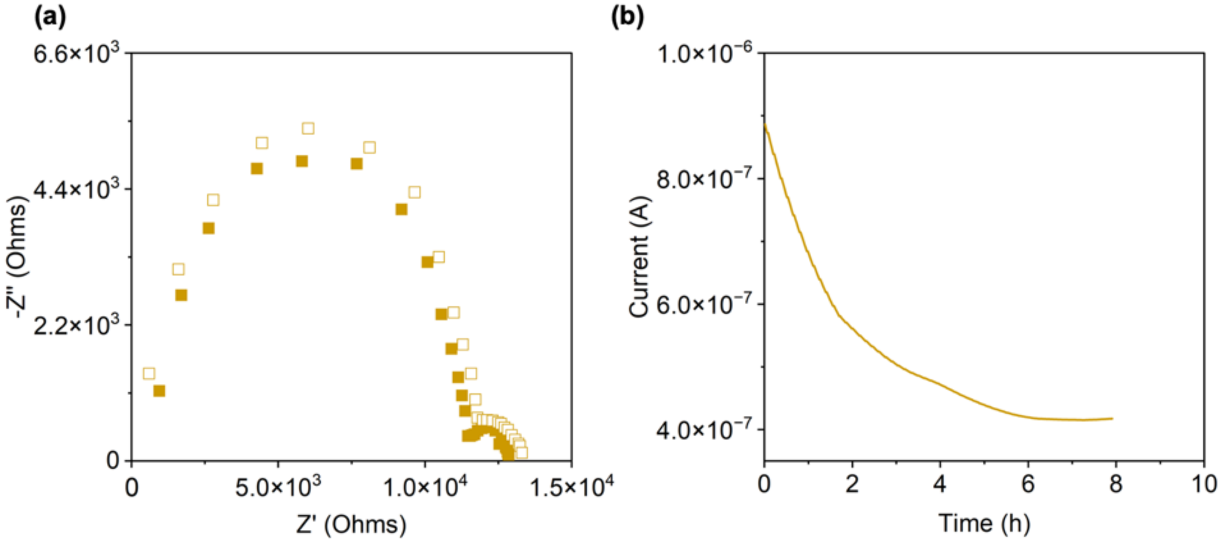

Figure S99. (a) Z'' as a function of Z' for lithium–lithium symmetric cell C before (filled squares) and after polarization (hollow squares). (b) Current *vs.* time during cell polarization.

8.3.4 POEM / PLiMTFSI / LiFSI blend; [EO]:[LiMTFSI]:[LiFSI] = 10:0.40:0.60 (molar ratio)

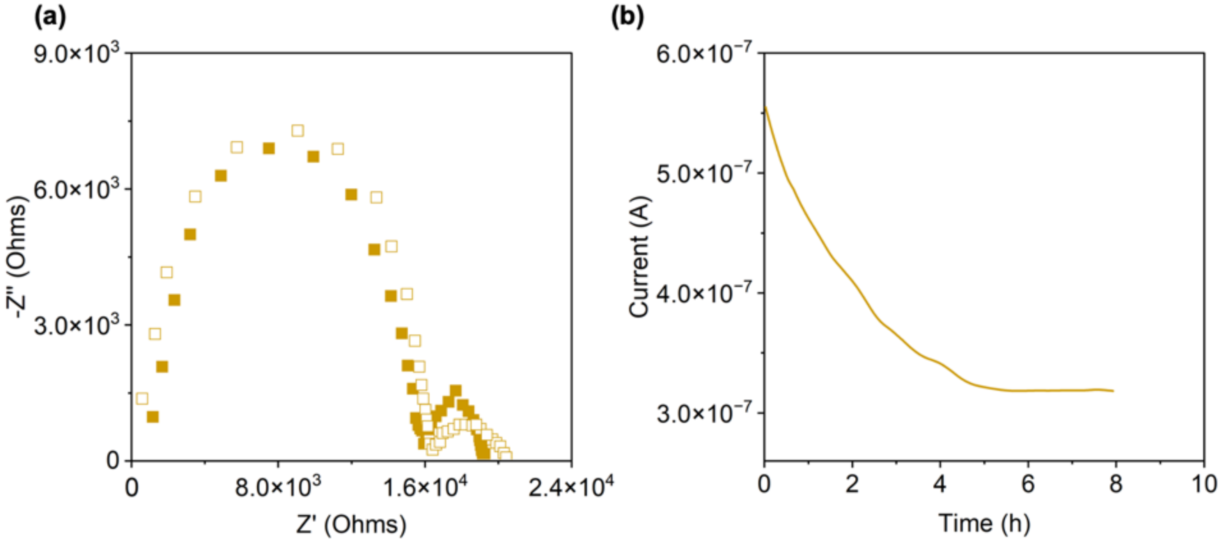

Figure S100. (a) Z″ as a function of Z′ for lithium–lithium symmetric cell A before (filled squares) and after polarization (hollow squares). (b) Current *vs.* time during cell polarization.

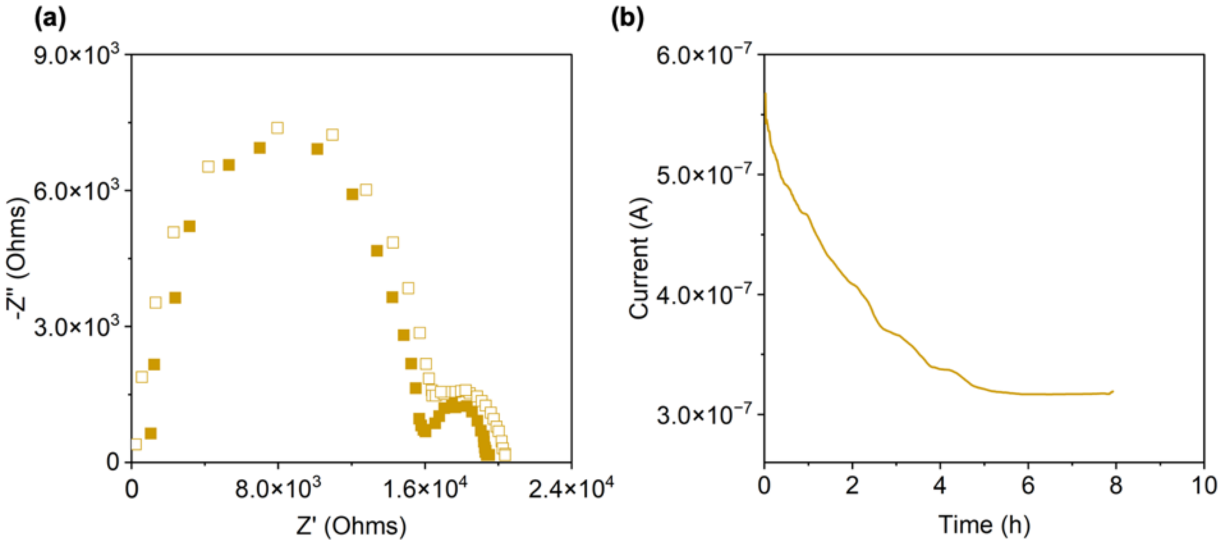

Figure S101. (a) Z'' as a function of Z' for lithium–lithium symmetric cell B before (filled squares) and after polarization (hollow squares). (b) Current *vs.* time during cell polarization.

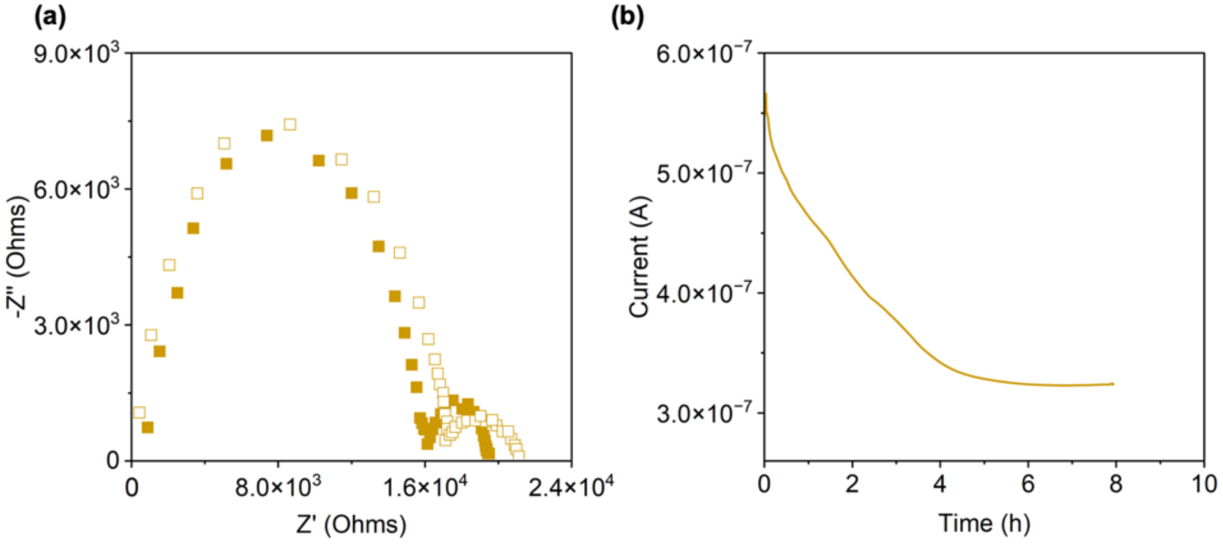

Figure S102. (a) Z'' as a function of Z' for lithium–lithium symmetric cell C before (filled squares) and after polarization (hollow squares). (b) Current *vs.* time during cell polarization.

8.3.5 POEM / PLiMTFSI / LiFSI blend; [EO]:[LiMTFSI]:[LiFSI] = 10:0.50:0.50 (molar ratio)

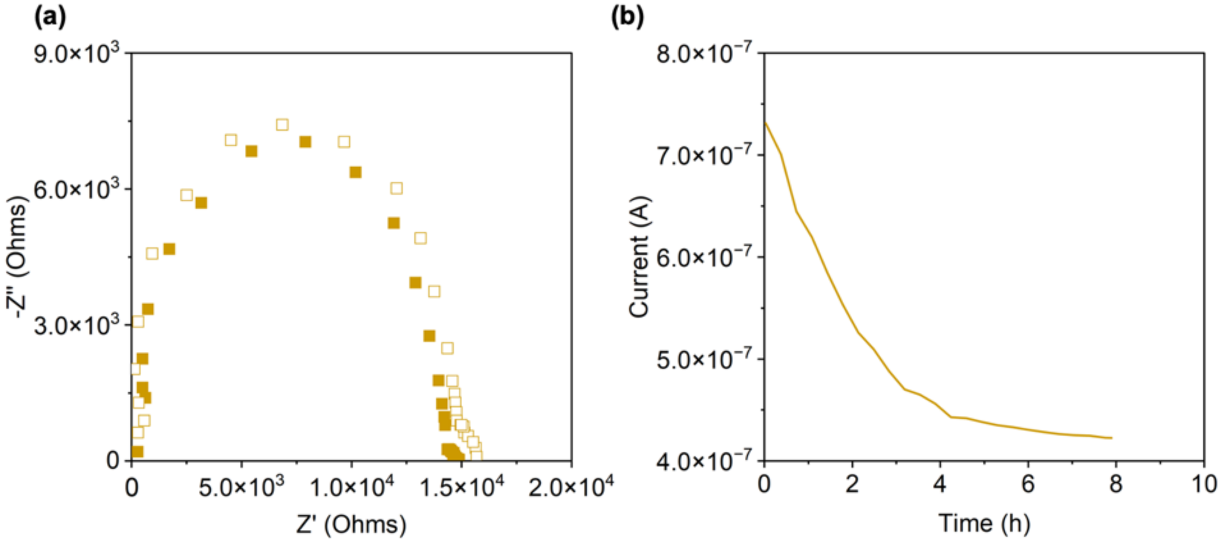

Figure S103. (a) Z'' as a function of Z' for lithium–lithium symmetric cell A before (filled squares) and after polarization (hollow squares). (b) Current *vs.* time during cell polarization.

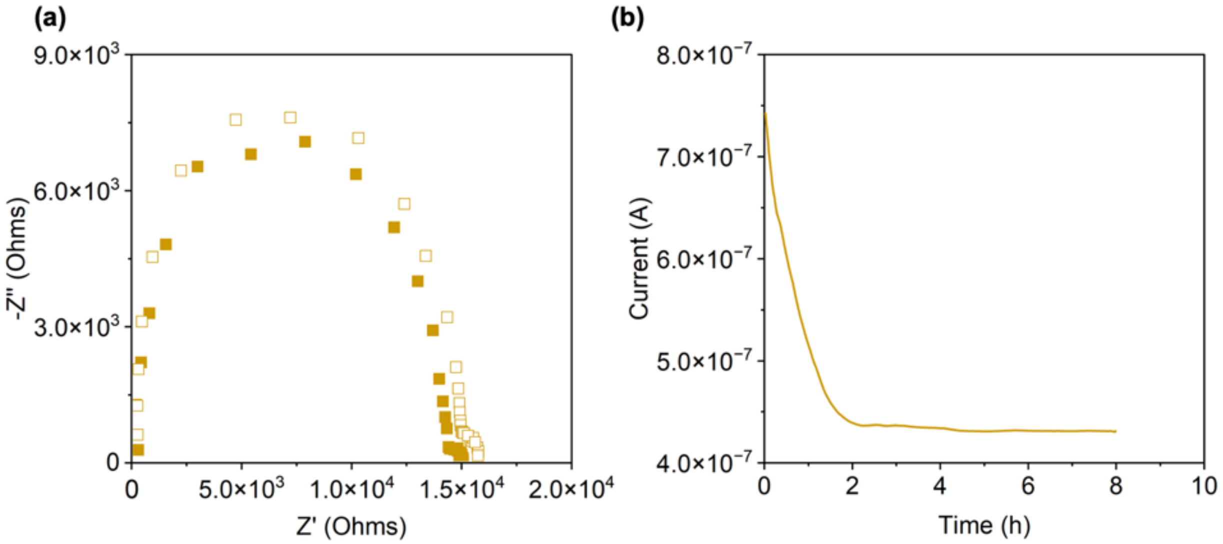

Figure S104. (a) Z'' as a function of Z' for lithium–lithium symmetric cell B before (filled squares) and after polarization (hollow squares). (b) Current *vs.* time during cell polarization.

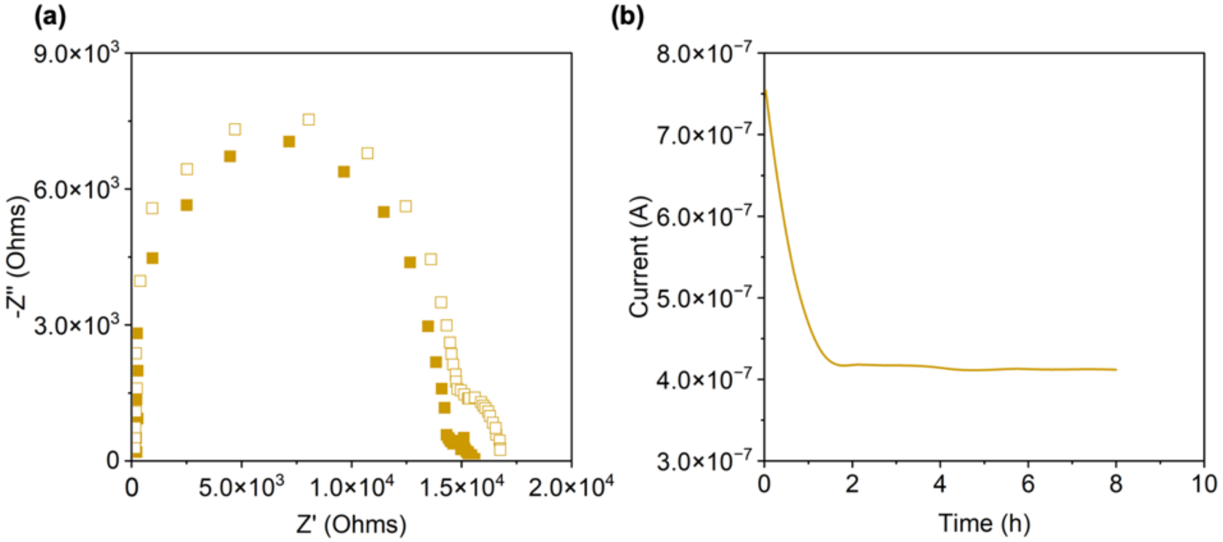

Figure S105. (a) Z'' as a function of Z' for lithium–lithium symmetric cell C before (filled squares) and after polarization (hollow squares). (b) Current *vs.* time during cell polarization.

8.3.6 POEM / PLiMTFSI / LiFSI blend; [EO]:[LiMTFSI]:[LiFSI] = 10:0.60:0.40 (molar ratio)

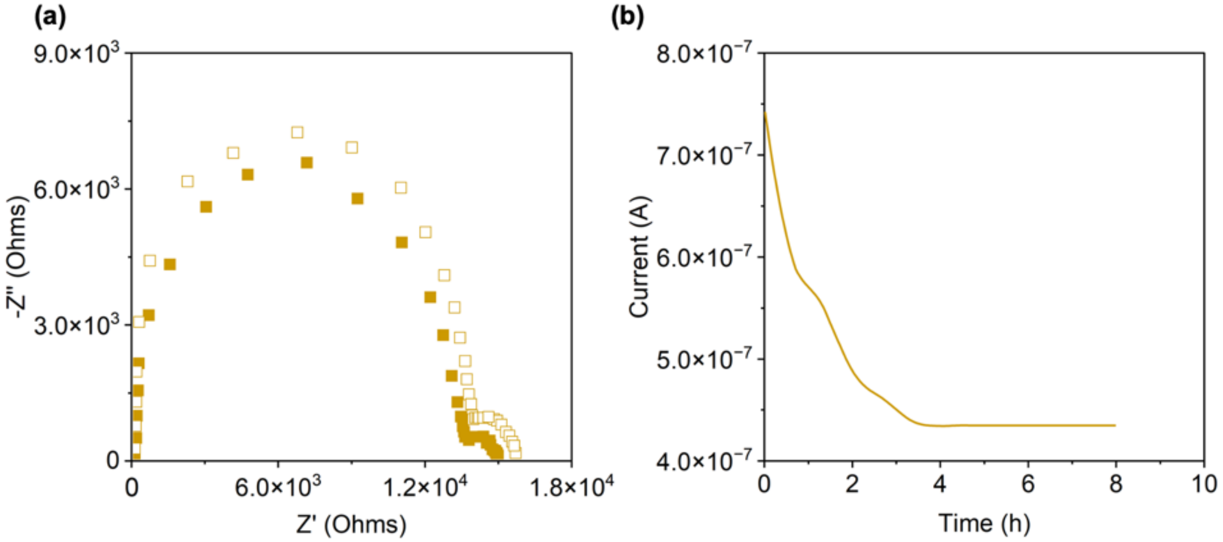

Figure S106. (a) Z″ as a function of Z′ for lithium–lithium symmetric cell A before (filled squares) and after polarization (hollow squares). (b) Current *vs.* time during cell polarization.

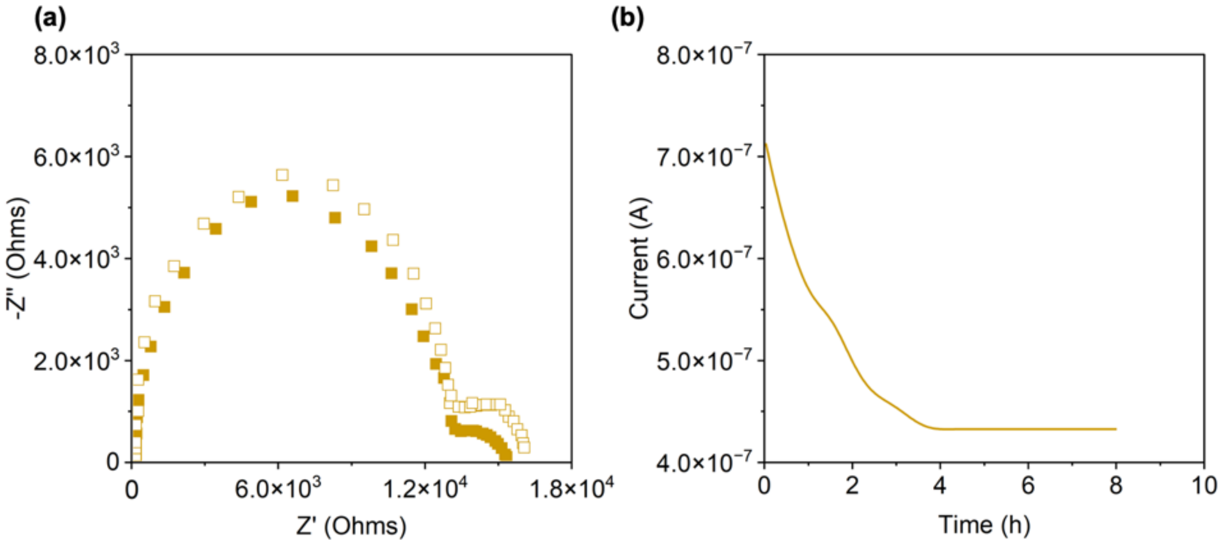

Figure S107. (a) Z'' as a function of Z' for lithium–lithium symmetric cell B before (filled squares) and after polarization (hollow squares). (b) Current *vs.* time during cell polarization.

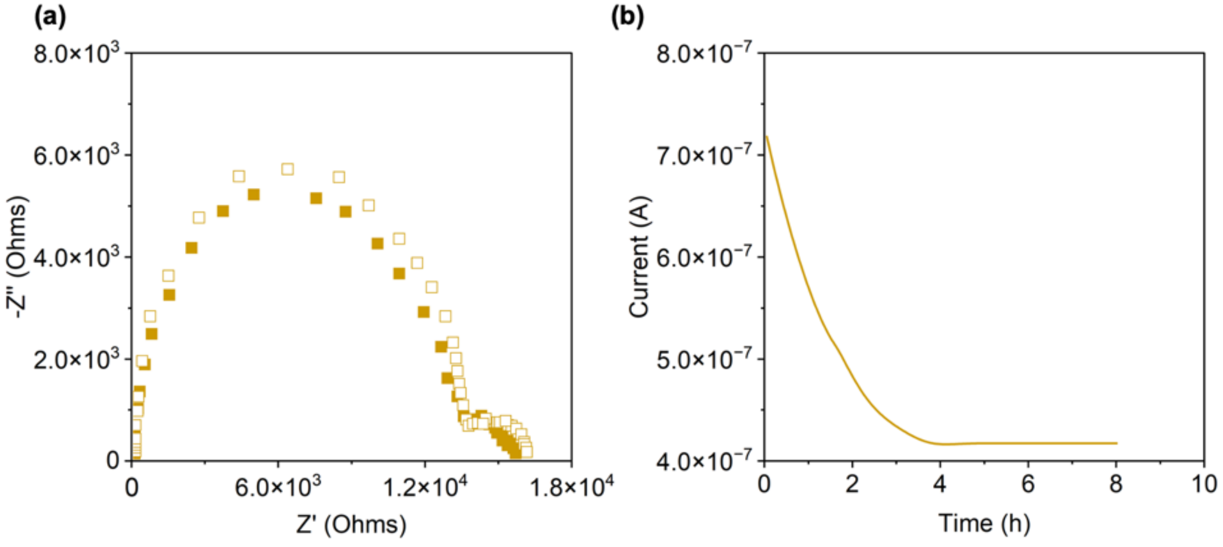

Figure S108. (a) Z'' as a function of Z' for lithium–lithium symmetric cell C before (filled squares) and after polarization (hollow squares). (b) Current *vs.* time during cell polarization.

8.3.7 POEM / PLiMTFSI / LiFSI blend; [EO]:[LiMTFSI]:[LiFSI] = 10:0.80:0.20 (molar ratio)

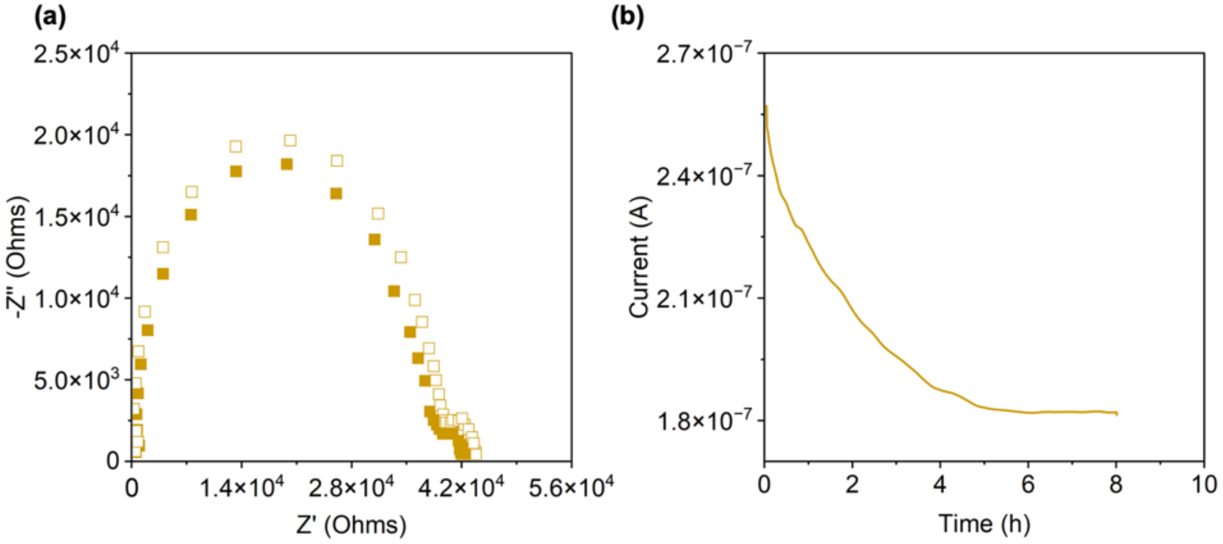

Figure S109. (a) Z″ as a function of Z′ for lithium–lithium symmetric cell A before (filled squares) and after polarization (hollow squares). (b) Current *vs.* time during cell polarization.

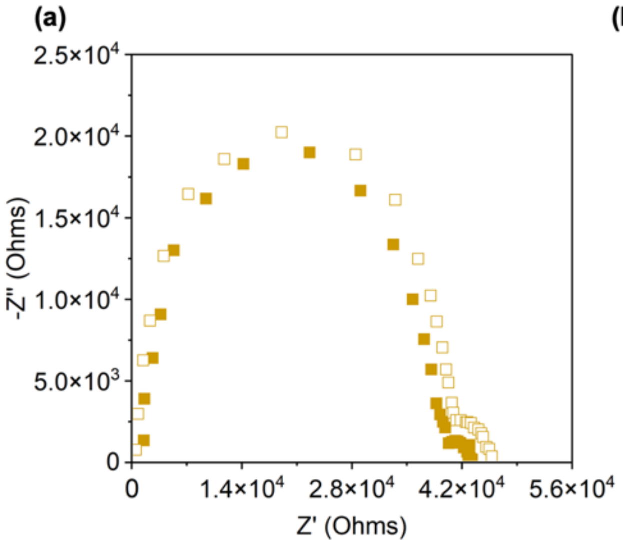

Figure S110. (a) Z'' as a function of Z' for lithium–lithium symmetric cell B before (filled squares) and after polarization (hollow squares). (b) Current *vs.* time during cell polarization.

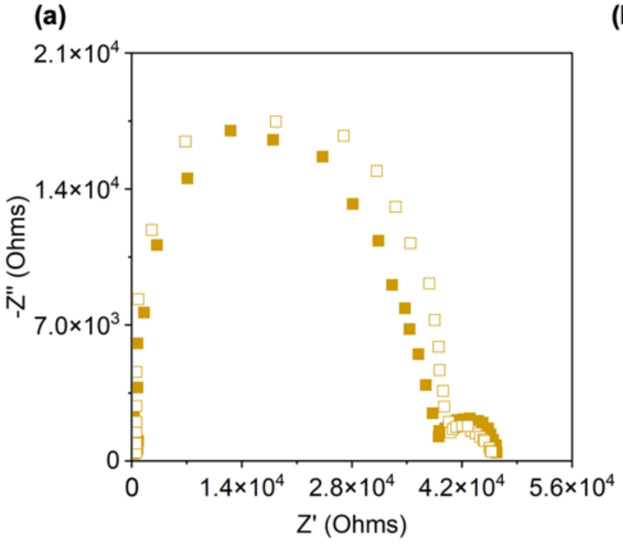

Figure S111. (a) Z'' as a function of Z' for lithium–lithium symmetric cell C before (filled squares) and after polarization (hollow squares). (b) Current *vs.* time during cell polarization.

8.4 Measurement conducted at 100 °C for POEM / PLiMTFSI / LiFSI blends

8.4.1 POEM / PLiMTFSI / LiFSI blend; [EO]:[LiMTFSI]:[LiFSI] = 10:0.05:0.95 (molar ratio)

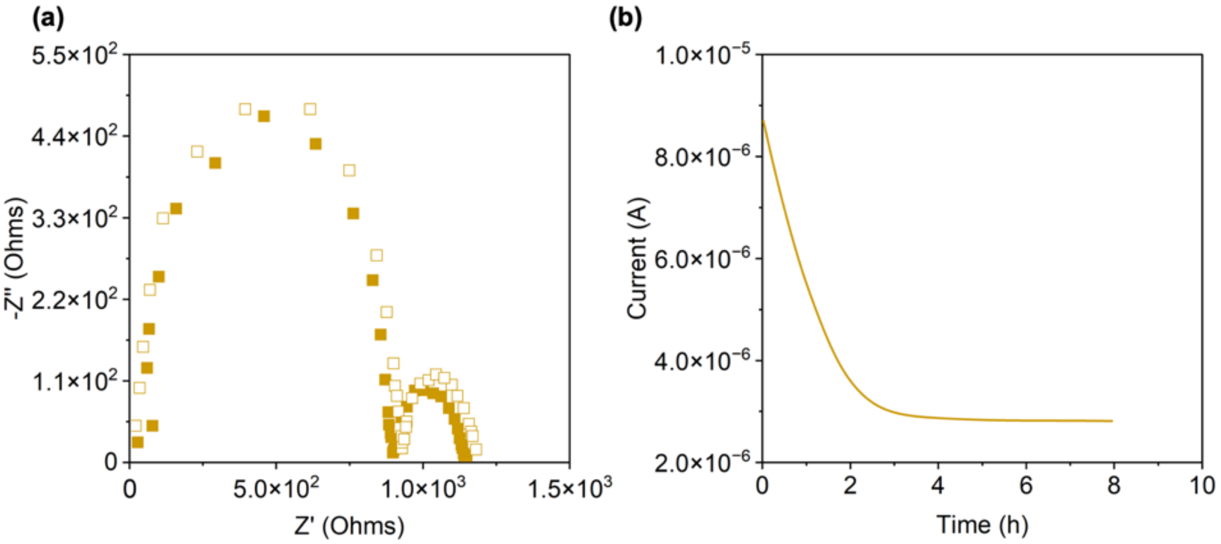

Figure S112. (a) Z'' as a function of Z' for lithium–lithium symmetric cell A before (filled squares) and after polarization (hollow squares). (b) Current *vs.* time during cell polarization.

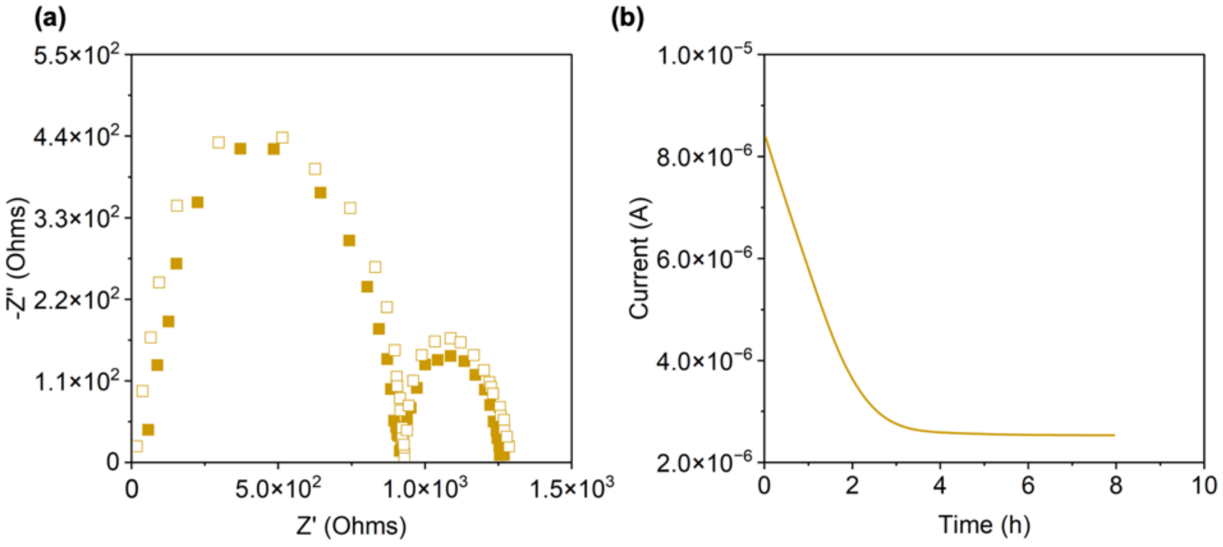

Figure S113. (a) Z'' as a function of Z' for lithium–lithium symmetric cell B before (filled squares) and after polarization (hollow squares). (b) Current *vs.* time during cell polarization.

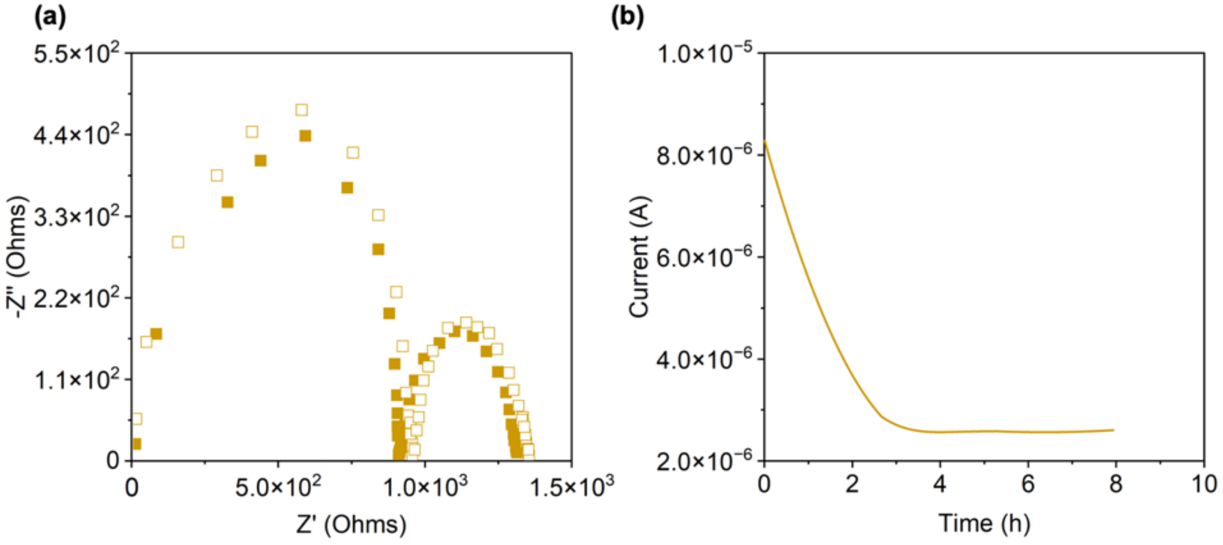

Figure S114. (a) Z'' as a function of Z' for lithium–lithium symmetric cell C before (filled squares) and after polarization (hollow squares). (b) Current *vs.* time during cell polarization.

8.4.2 POEM / PLiMTFSI / LiFSI blend; [EO]:[LiMTFSI]:[LiFSI] = 10:0.15:0.85 (molar ratio)

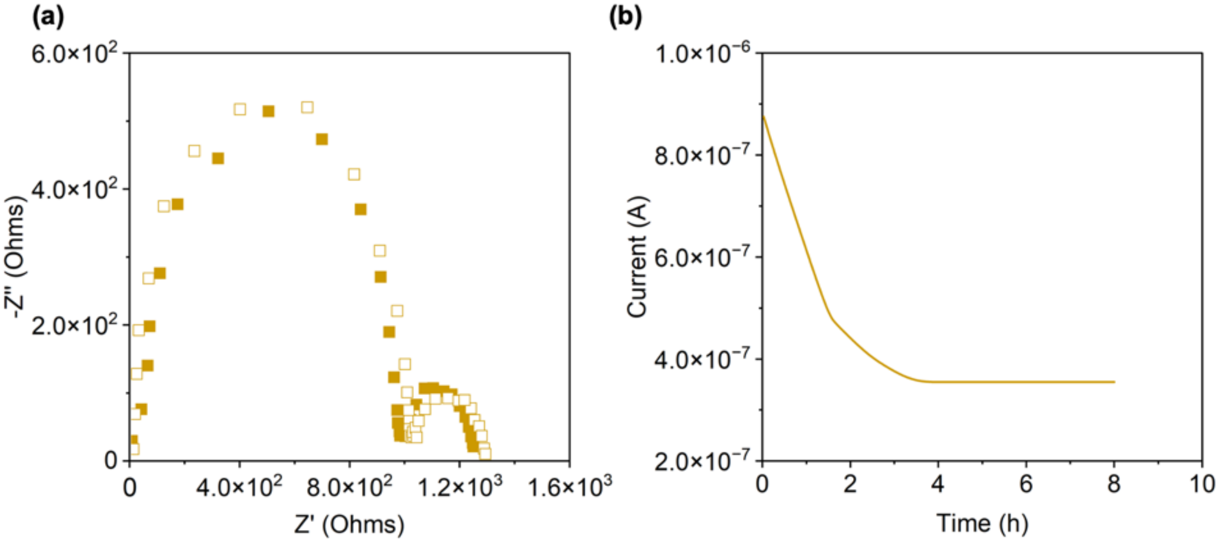

Figure S115. (a) Z″ as a function of Z′ for lithium–lithium symmetric cell A before (filled squares) and after polarization (hollow squares). (b) Current *vs.* time during cell polarization.

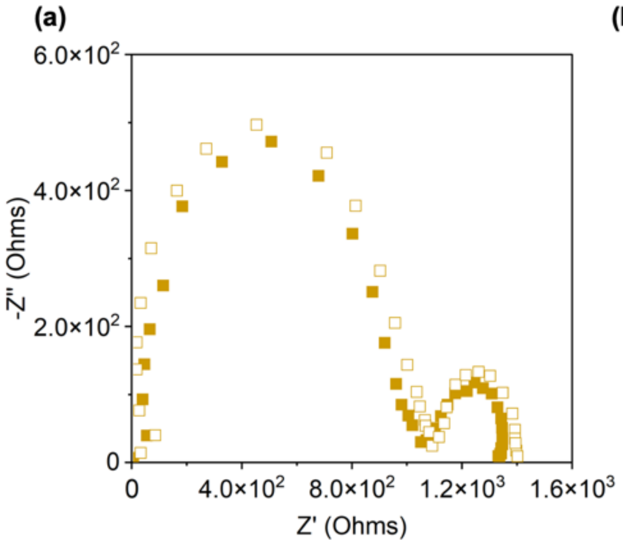

Figure S116. (a) Z'' as a function of Z' for lithium–lithium symmetric cell B before (filled squares) and after polarization (hollow squares). (b) Current *vs.* time during cell polarization.

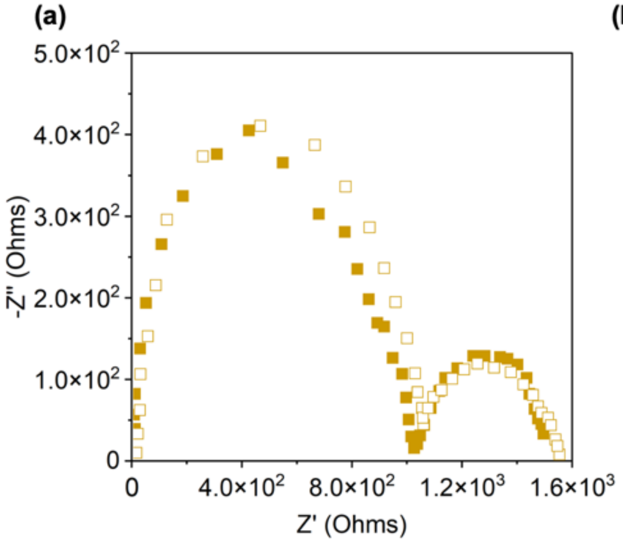

Figure S117. (a) Z'' as a function of Z' for lithium–lithium symmetric cell C before (filled squares) and after polarization (hollow squares). (b) Current *vs.* time during cell polarization.

8.4.3 POEM / PLiMTFSI / LiFSI blend; [EO]:[LiMTFSI]:[LiFSI] = 10:0.25:0.75 (molar ratio)

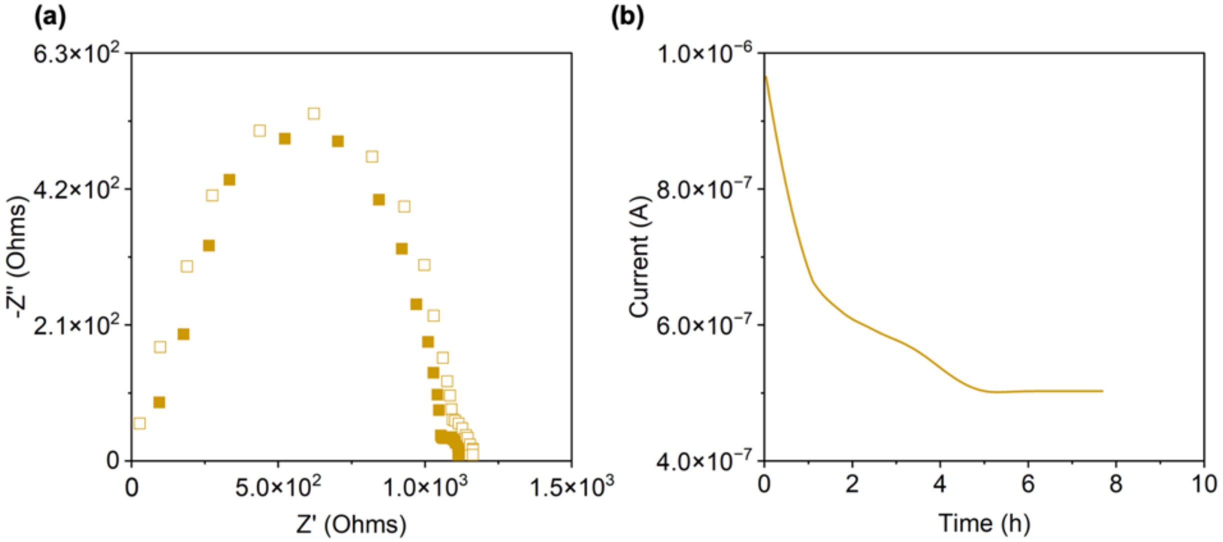

Figure S118. (a) Z″ as a function of Z′ for lithium–lithium symmetric cell A before (filled squares) and after polarization (hollow squares). (b) Current *vs.* time during cell polarization.

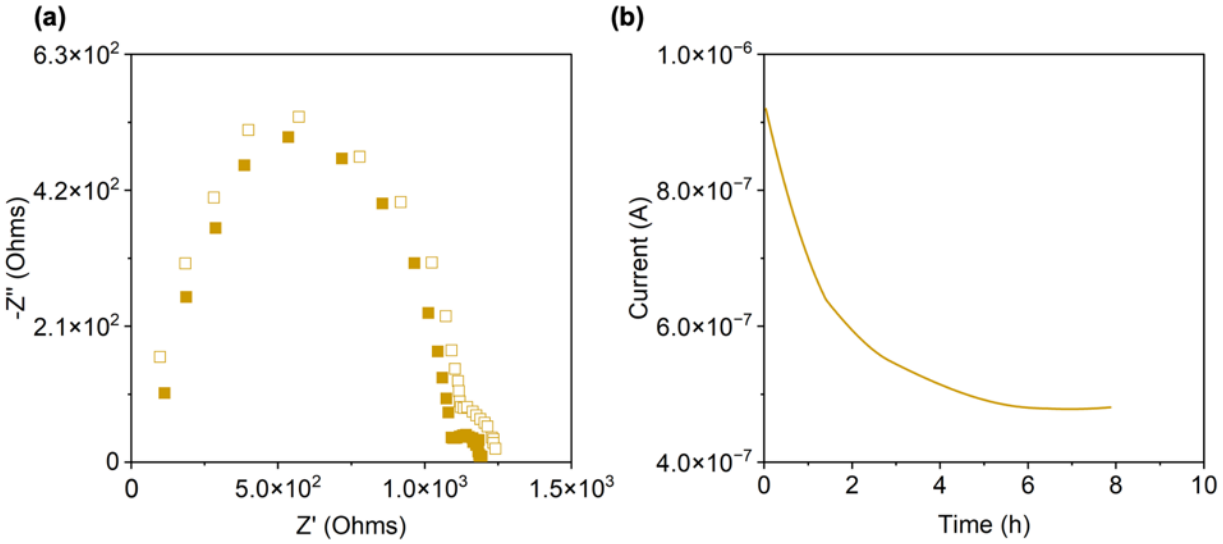

Figure S119. (a) Z'' as a function of Z' for lithium–lithium symmetric cell B before (filled squares) and after polarization (hollow squares). (b) Current *vs.* time during cell polarization.

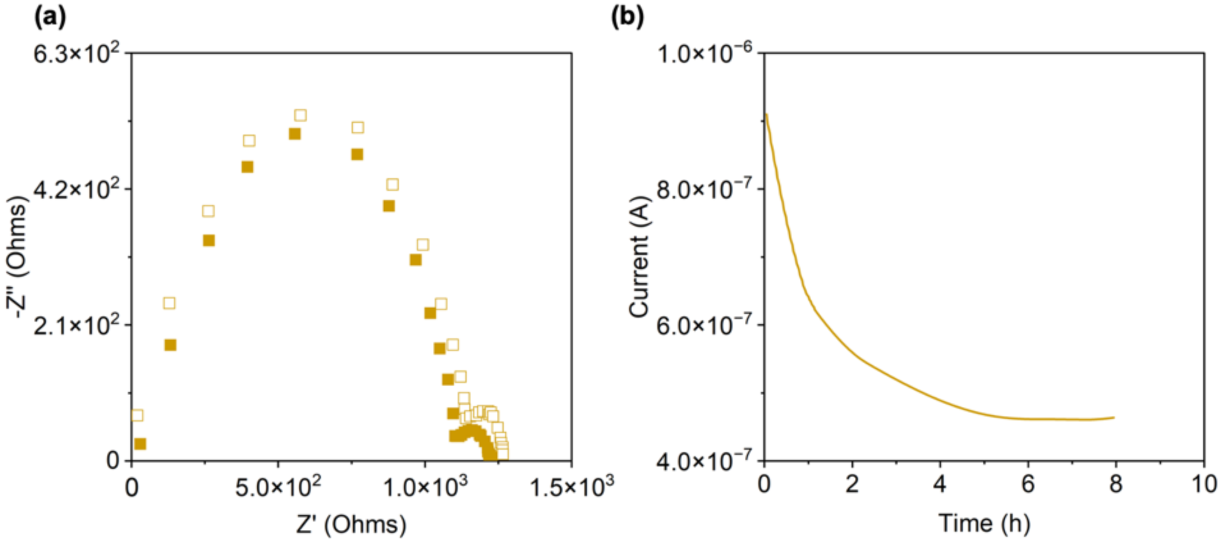

Figure S120. (a) Z'' as a function of Z' for lithium–lithium symmetric cell C before (filled squares) and after polarization (hollow squares). (b) Current *vs.* time during cell polarization.

8.4.4 POEM / PLiMTFSI / LiFSI blend; [EO]:[LiMTFSI]:[LiFSI] = 10:0.40:0.60 (molar ratio)

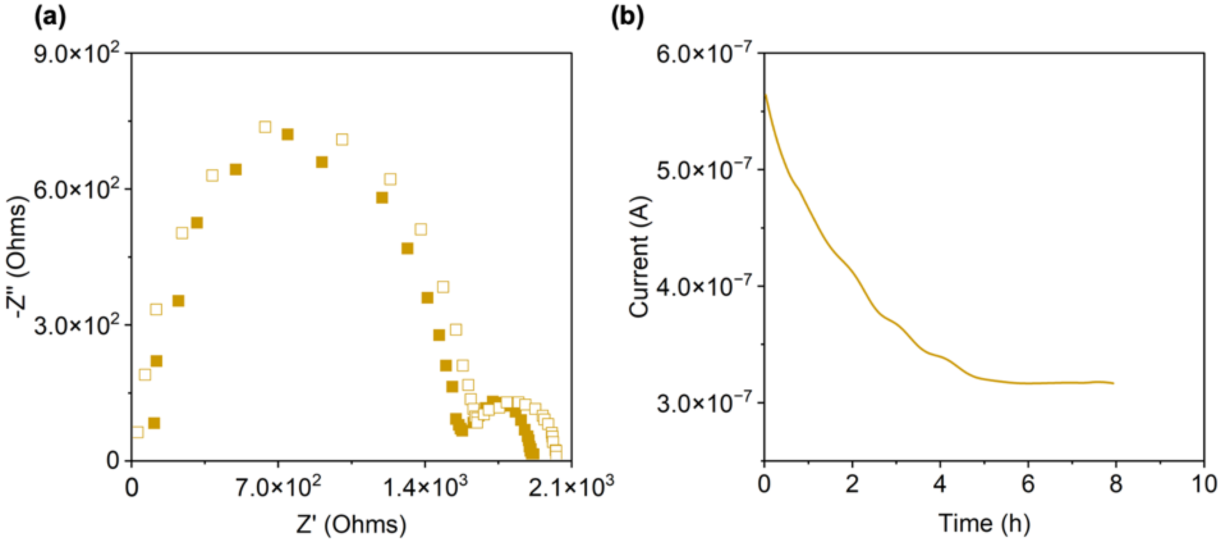

Figure S121. (a) Z″ as a function of Z′ for lithium–lithium symmetric cell A before (filled squares) and after polarization (hollow squares). (b) Current *vs.* time during cell polarization.

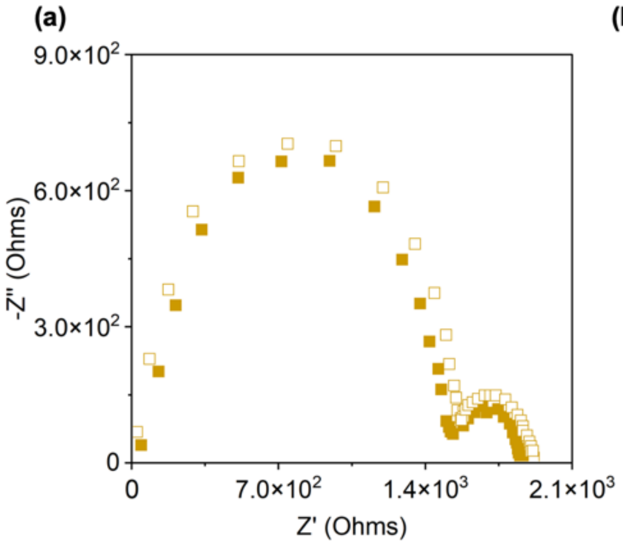

Figure S122. (a) Z'' as a function of Z' for lithium–lithium symmetric cell B before (filled squares) and after polarization (hollow squares). (b) Current *vs.* time during cell polarization.

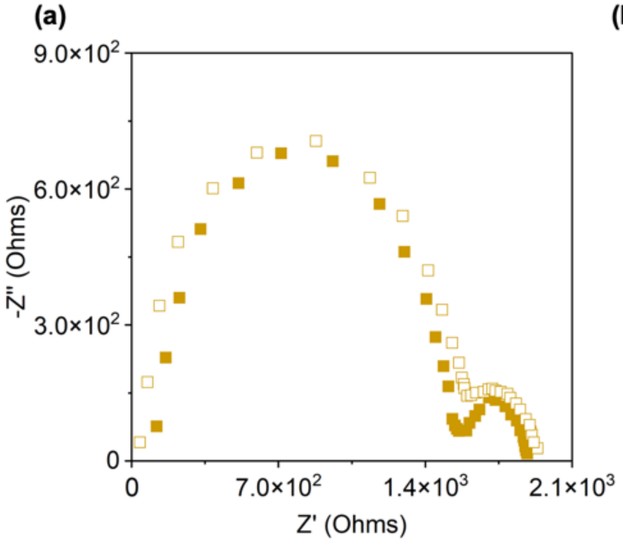

Figure S123. (a) Z'' as a function of Z' for lithium–lithium symmetric cell C before (filled squares) and after polarization (hollow squares). (b) Current *vs.* time during cell polarization.

8.4.5 POEM / PLiMTFSI / LiFSI blend; [EO]:[LiMTFSI]:[LiFSI] = 10:0.50:0.50 (molar ratio)

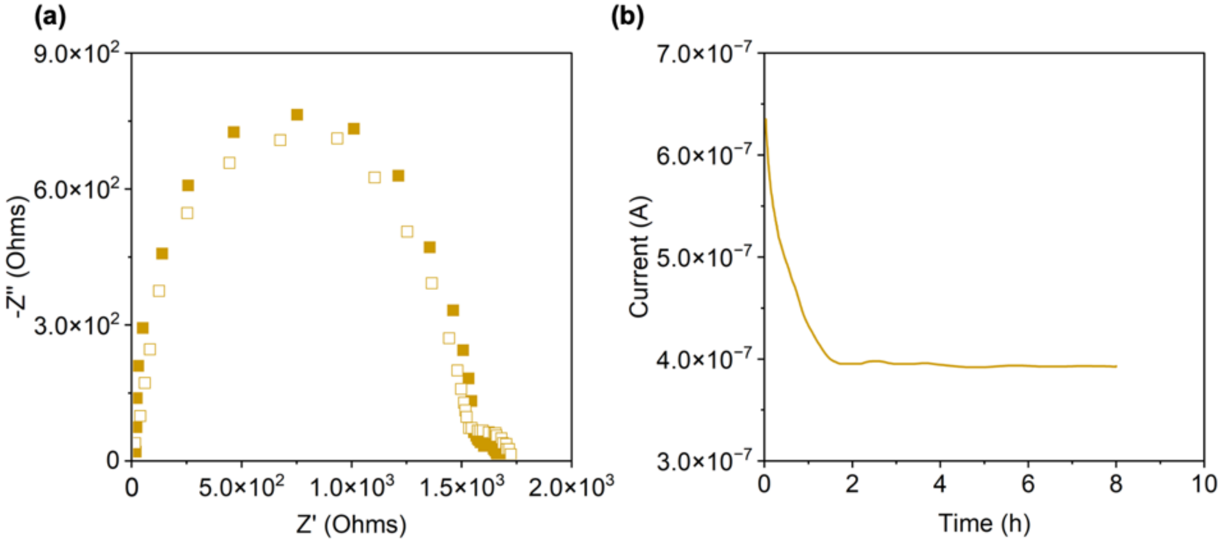

Figure S124. (a) Z″ as a function of Z′ for lithium–lithium symmetric cell A before (filled squares) and after polarization (hollow squares). (b) Current *vs.* time during cell polarization.

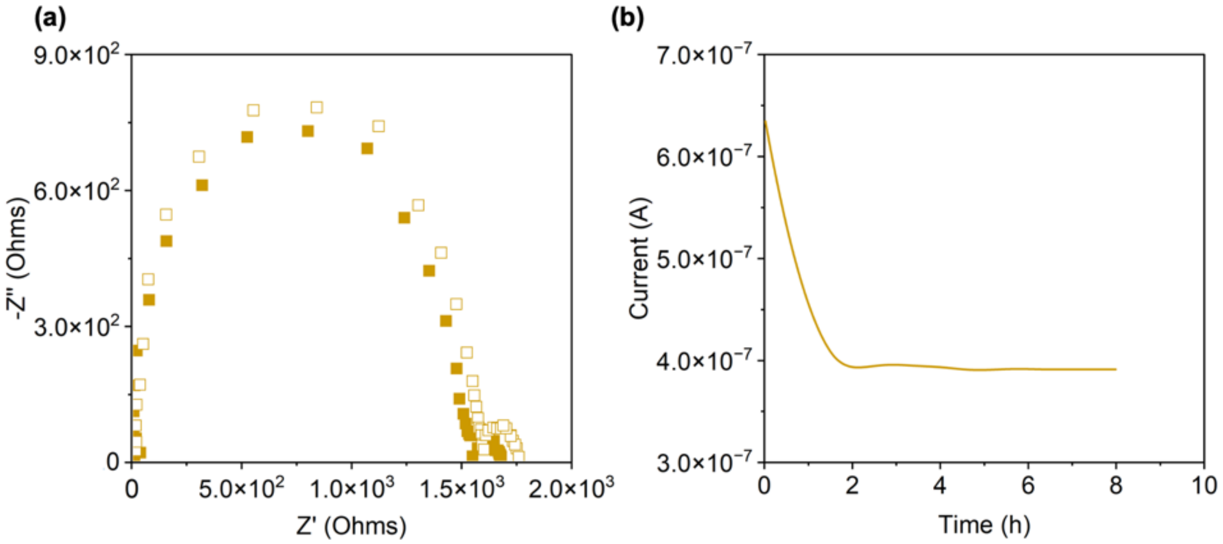

Figure S125. (a) Z'' as a function of Z' for lithium–lithium symmetric cell B before (filled squares) and after polarization (hollow squares). (b) Current *vs.* time during cell polarization.

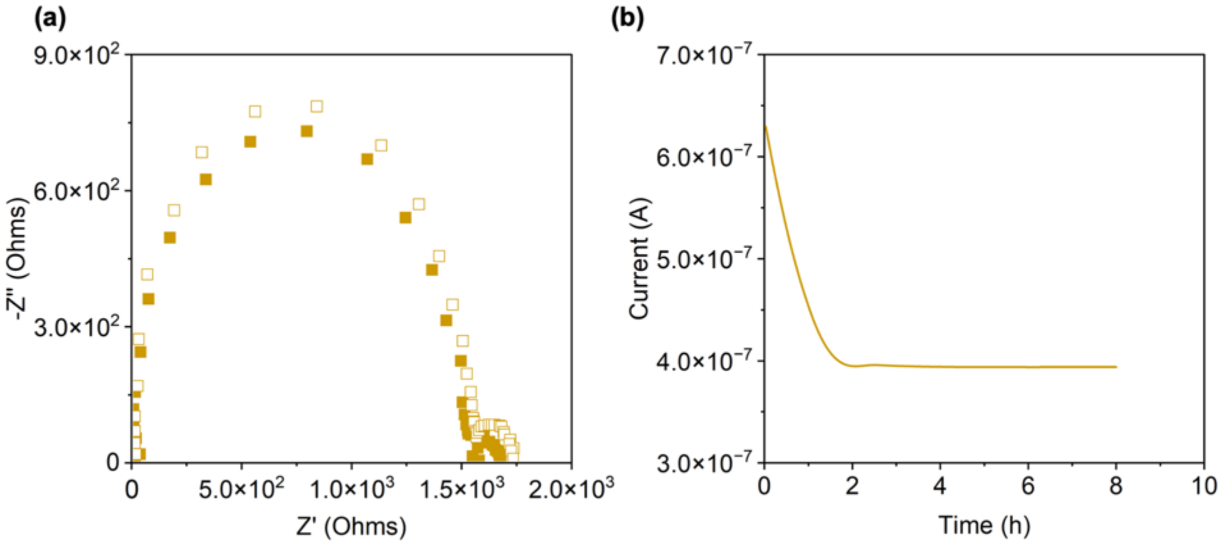

Figure S126. (a) Z'' as a function of Z' for lithium–lithium symmetric cell C before (filled squares) and after polarization (hollow squares). (b) Current *vs.* time during cell polarization.

8.4.6 POEM / PLiMTFSI / LiFSI blend; [EO]:[LiMTFSI]:[LiFSI] = 10:0.60:0.40 (molar ratio)

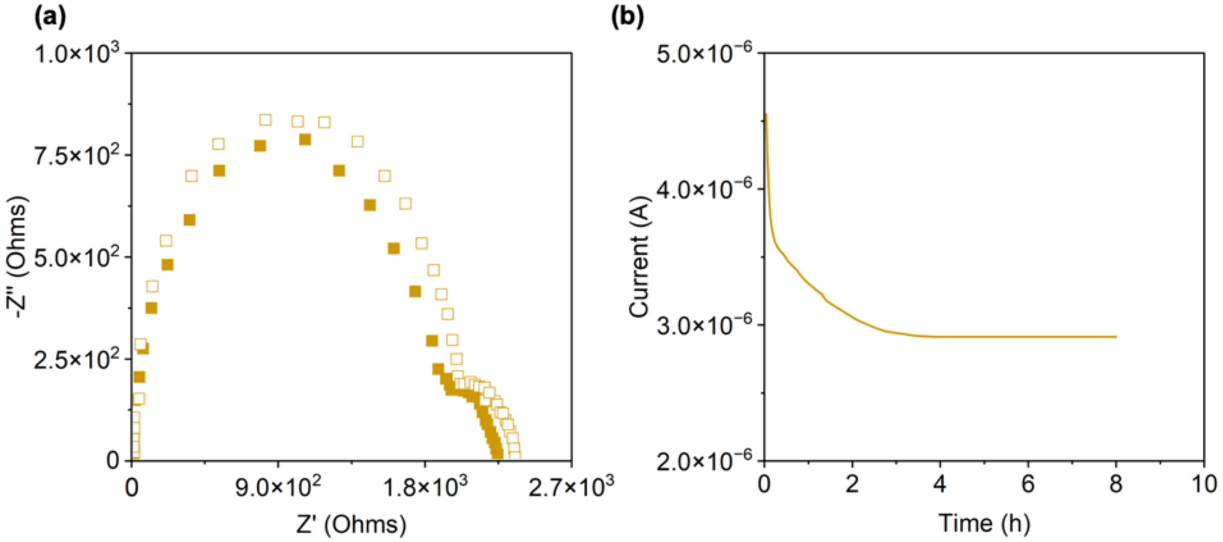

Figure S127. (a) Z'' as a function of Z' for lithium–lithium symmetric cell A before (filled squares) and after polarization (hollow squares). (b) Current *vs.* time during cell polarization.

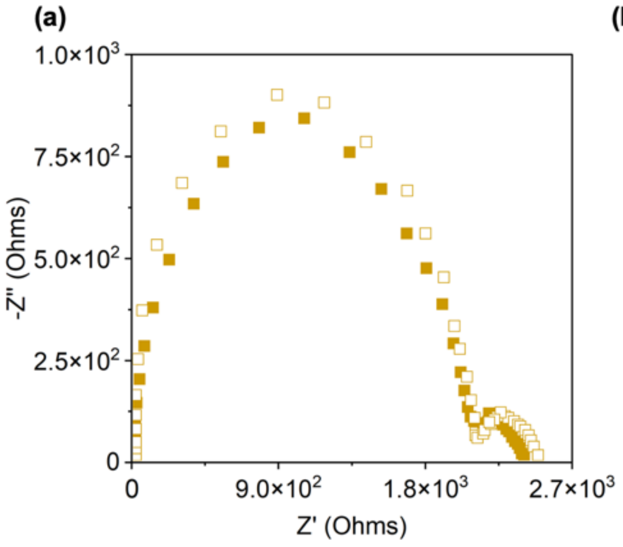

Figure S128. (a) Z'' as a function of Z' for lithium–lithium symmetric cell B before (filled squares) and after polarization (hollow squares). (b) Current *vs.* time during cell polarization.

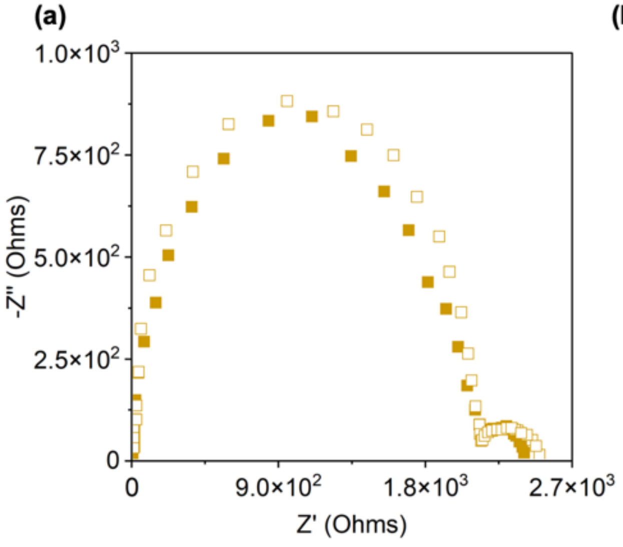

Figure S129. (a) Z'' as a function of Z' for lithium–lithium symmetric cell C before (filled squares) and after polarization (hollow squares). (b) Current *vs.* time during cell polarization.

8.4.7 POEM / PLiMTFSI / LiFSI blend; [EO]:[LiMTFSI]:[LiFSI] = 10:0.80:0.20 (molar ratio)

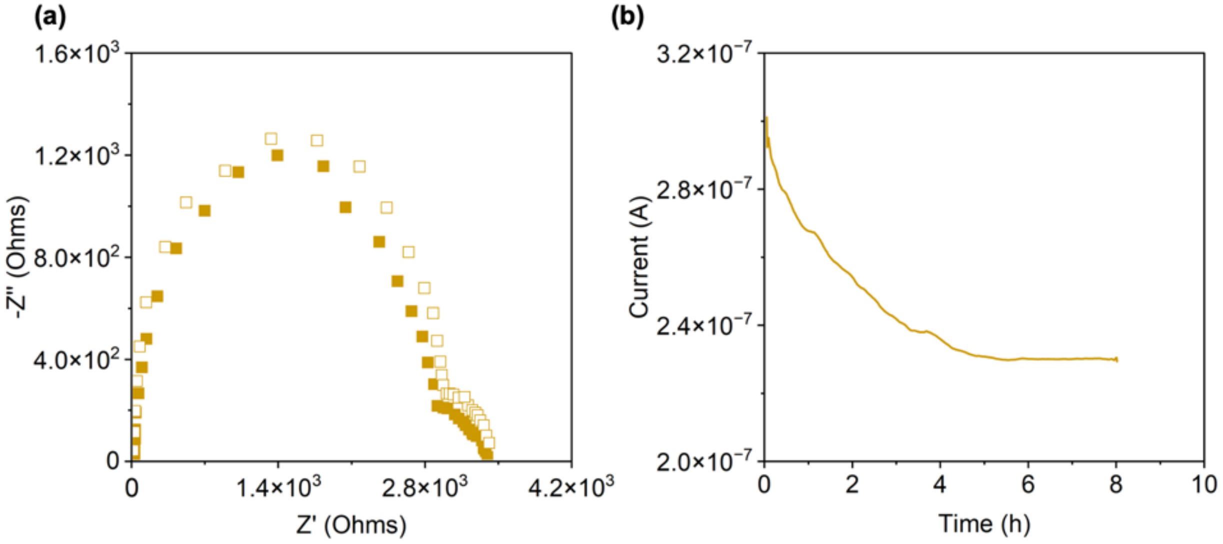

Figure S130. (a) Z″ as a function of Z′ for lithium–lithium symmetric cell A before (filled squares) and after polarization (hollow squares). (b) Current *vs.* time during cell polarization.

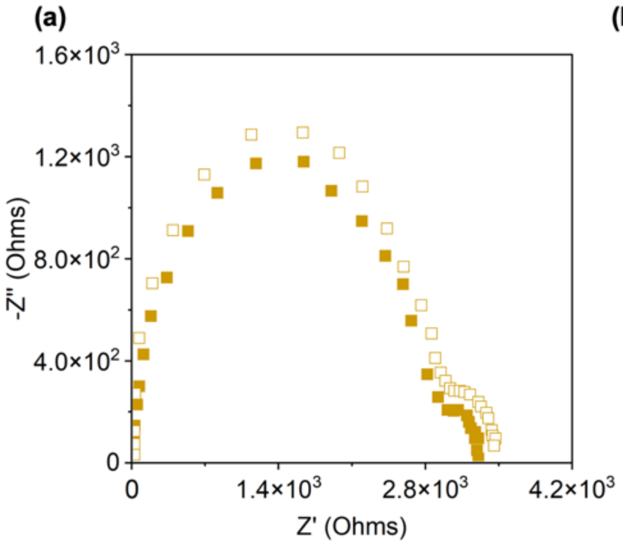

Figure S131. (a) Z'' as a function of Z' for lithium–lithium symmetric cell B before (filled squares) and after polarization (hollow squares). (b) Current *vs.* time during cell polarization.

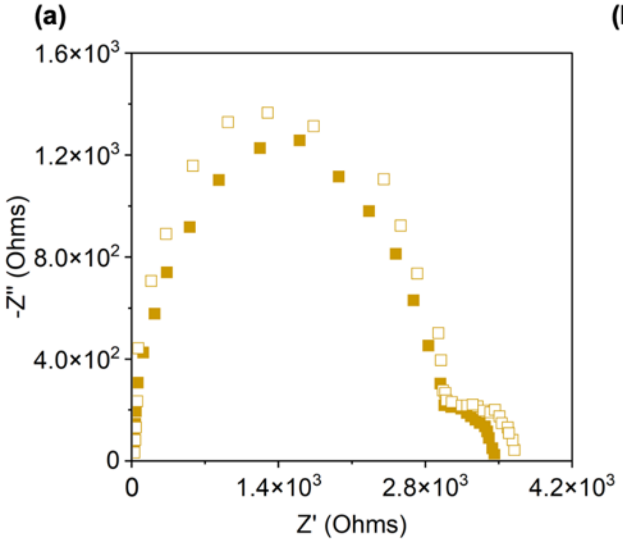

Figure S132. (a) Z'' as a function of Z' for lithium–lithium symmetric cell C before (filled squares) and after polarization (hollow squares). (b) Current *vs.* time during cell polarization.

Summary of the parameters for the Bruce-Vincent method for POEM / PLiMTFSI / LiTf blends.

Table S18 (a). Impedance and current values extracted from AC impedance spectroscopy and potentiostatic polarization measurements of three lithium–lithium symmetric cells for POEM / PLiMTFSI / LiTf blend; [EO]:[LiMTFSI]:[LiTf] = 10:0.05:0.95 (molar ratio) at 60 °C.

	$^{b}R_1$ (Ohms)	$^{b}R_2$ (Ohms)	$^{c}R_1^*$ (Ohms)	$^{c}R_2^*$ (Ohms)	$^{d}R_0$ (Ohms)	$^{e}R_{SS}$ (Ohms)	$^{f}I_0$ (A)	I_{SS} (A)	t_{Li+}
^{a}Cell A	9780	1.44×10^4	1.00×10^4	1.50×10^4	4610	4940	6.95×10^{-7}	1.46×10^{-7}	0.21
^{a}Cell B	9750	1.47×10^4	1.00×10^4	1.49×10^4	4970	4890	6.80×10^{-7}	1.36×10^{-7}	0.20
^{a}Cell C	9830	1.49×10^4	1.02×10^4	1.52×10^4	5030	4980	6.73×10^{-7}	1.35×10^{-7}	0.20

^{a}Cells A, B, and C were made with lithium non-blocking electrodes. $^{b}R_1$ and R_2 are the impedances of the minima at the bounds of the low-frequency semicircle in a Nyquist plot before cell polarization. $^{c}R_1$* and R_2* are the corresponding impedances after polarization. ^{d}Initial interfacial impedance was defined as $R_0 = R_2 - R_1$. ^{e}Steady-state interfacial impedance was defined as $R_{SS} = R_2^* - R_1^*$. ^{f}Initial current, I_0, was calculated by Ohm's law. This footnote applies to all tables in this section.

Table S18 (b). Impedance and current values extracted from AC impedance spectroscopy and potentiostatic polarization measurements of three lithium–lithium symmetric cells for POEM / PLiMTFSI / LiTf blend; [EO]:[LiMTFSI]:[LiTf] = 10:0.05:0.95 (molar ratio) at 100 °C.

	$^{b}R_1$ (Ohms)	$^{b}R_2$ (Ohms)	$^{c}R_1^*$ (Ohms)	$^{c}R_2^*$ (Ohms)	$^{d}R_0$ (Ohms)	$^{e}R_{SS}$ (Ohms)	$^{f}I_0$ (A)	I_{SS} (A)	t_{Li+}
^{a}Cell A	1430	2480	1490	2520	1050	1030	4.02×10^{-6}	9.66×10^{-7}	0.24
^{a}Cell B	1430	2490	1490	2520	1060	1030	4.01×10^{-6}	1.00×10^{-6}	0.25
^{a}Cell C	1440	2490	1460	2550	1050	1090	4.01×10^{-6}	9.63×10^{-7}	0.24

Table S19 (a). Impedance and current values extracted from AC impedance spectroscopy and potentiostatic polarization measurements of three lithium–lithium symmetric cells for POEM / PLiMTFSI / LiTf blend; [EO]:[LiMTFSI]:[LiTf] = 10:0.15:0.85 (molar ratio) at 60 °C.

	$^{b}R_1$ (Ohms)	$^{b}R_2$ (Ohms)	$^{c}R_1^*$ (Ohms)	$^{c}R_2^*$ (Ohms)	$^{d}R_0$ (Ohms)	$^{e}R_{SS}$ (Ohms)	$^{f}I_0$ (A)	I_{SS} (A)	t_{Li+}
^{a}Cell A	1.16×10^{4}	2.00×10^{4}	1.19×10^{4}	2.03×10^{4}	8490	8450	4.99×10^{-7}	1.50×10^{-7}	0.30
^{a}Cell B	1.16×10^{4}	2.00×10^{4}	1.21×10^{4}	1.97×10^{4}	8360	7580	5.00×10^{-7}	1.50×10^{-7}	0.30
^{a}Cell C	1.16×10^{4}	1.98×10^{4}	1.18×10^{4}	2.03×10^{4}	8220	8510	5.04×10^{-7}	1.51×10^{-7}	0.30

Table S19 (b). Impedance and current values extracted from AC impedance spectroscopy and potentiostatic polarization measurements of three lithium–lithium symmetric cells for POEM / PLiMTFSI / LiTf blend; [EO]:[LiMTFSI]:[LiTf] = 10:0.15:0.85 (molar ratio) at 100 °C.

	$^{b}R_1$ (Ohms)	$^{b}R_2$ (Ohms)	$^{c}R_1^*$ (Ohms)	$^{c}R_2^*$ (Ohms)	$^{d}R_0$ (Ohms)	$^{e}R_{SS}$ (Ohms)	$^{f}I_0$ (A)	I_{SS} (A)	t_{Li+}
^{a}Cell A	1660	2550	1710	2590	890	880	3.92×10^{-6}	1.30×10^{-6}	0.33
^{a}Cell B	1670	2590	1720	2630	920	910	3.86×10^{-6}	1.27×10^{-6}	0.33
^{a}Cell C	1650	2530	1700	2610	880	910	3.95×10^{-6}	1.30×10^{-6}	0.33

Table S20 (a). Impedance and current values extracted from AC impedance spectroscopy and potentiostatic polarization measurements of three lithium–lithium symmetric cells for POEM / PLiMTFSI / LiTf blend; [EO]:[LiMTFSI]:[LiTf] = 10:0.25:0.75 (molar ratio) at 60 °C.

	$^{b}R_1$ (Ohms)	$^{b}R_2$ (Ohms)	$^{c}R_1^*$ (Ohms)	$^{c}R_2^*$ (Ohms)	$^{d}R_0$ (Ohms)	$^{e}R_{SS}$ (Ohms)	$^{f}I_0$ (A)	I_{SS} (A)	t_{Li+}
^{a}Cell A	1.56×10^{4}	1.83×10^{4}	1.58×10^{4}	1.86×10^{4}	2710	2750	5.46×10^{-7}	2.24×10^{-7}	0.41
^{a}Cell B	1.56×10^{4}	1.81×10^{4}	1.57×10^{4}	1.84×10^{4}	2480	2680	5.53×10^{-7}	2.27×10^{-7}	0.41
^{a}Cell C	1.56×10^{4}	1.77×10^{4}	1.57×10^{4}	1.80×10^{4}	2150	2290	5.64×10^{-7}	2.37×10^{-7}	0.42

Table S20 (b). Impedance and current values extracted from AC impedance spectroscopy and potentiostatic polarization measurements of three lithium–lithium symmetric cells for POEM / PLiMTFSI / LiTf blend; [EO]:[LiMTFSI]:[LiTf] = 10:0.25:0.75 (molar ratio) at 100 °C.

	$^{b}R_1$ (Ohms)	$^{b}R_2$ (Ohms)	$^{c}R_1^*$ (Ohms)	$^{c}R_2^*$ (Ohms)	$^{d}R_0$ (Ohms)	$^{e}R_{SS}$ (Ohms)	$^{f}I_0$ (A)	I_{SS} (A)	t_{Li+}
^{a}Cell A	2120	2350	2120	2390	230	270	4.26×10^{-6}	1.88×10^{-6}	0.44
^{a}Cell B	2110	2270	2140	2340	160	200	4.41×10^{-6}	1.94×10^{-6}	0.44
^{a}Cell C	2180	2330	2170	2380	150	210	4.29×10^{-6}	1.89×10^{-6}	0.44

Table S21 (a). Impedance and current values extracted from AC impedance spectroscopy and potentiostatic polarization measurements of three lithium–lithium symmetric cells for POEM / PLiMTFSI / LiTf blend; [EO]:[LiMTFSI]:[LiTf] = 10:0.40:0.60 (molar ratio) at 60 °C.

	$^{b}R_1$ (Ohms)	$^{b}R_2$ (Ohms)	$^{c}R_1^*$ (Ohms)	$^{c}R_2^*$ (Ohms)	$^{d}R_0$ (Ohms)	$^{e}R_{SS}$ (Ohms)	$^{f}I_0$ (A)	I_{SS} (A)	t_{Li+}
^{a}Cell A	2.16×10^{4}	2.28×10^{4}	2.15×10^{4}	2.34×10^{4}	1190	1830	4.39×10^{-7}	1.89×10^{-7}	0.43
^{a}Cell B	2.04×10^{4}	2.27×10^{4}	2.06×10^{4}	2.31×10^{4}	2290	2530	4.41×10^{-7}	1.85×10^{-7}	0.42
^{a}Cell C	2.16×10^{4}	2.25×10^{4}	2.13×10^{4}	2.31×10^{4}	980	1820	4.44×10^{-7}	1.91×10^{-7}	0.43

Table S21 (b). Impedance and current values extracted from AC impedance spectroscopy and potentiostatic polarization measurements of three lithium–lithium symmetric cells for POEM / PLiMTFSI / LiTf blend; [EO]:[LiMTFSI]:[LiTf] = 10:0.40:0.60 (molar ratio) at 100 °C.

	$^{b}R_1$ (Ohms)	$^{b}R_2$ (Ohms)	$^{c}R_1^*$ (Ohms)	$^{c}R_2^*$ (Ohms)	$^{d}R_0$ (Ohms)	$^{e}R_{SS}$ (Ohms)	$^{f}I_0$ (A)	I_{SS} (A)	t_{Li+}
^{a}Cell A	2650	2820	2650	2810	170	160	3.54×10^{-6}	1.56×10^{-6}	0.44
^{a}Cell B	2640	2780	2680	2830	140	150	3.59×10^{-6}	1.58×10^{-6}	0.44
^{a}Cell C	2640	2790	2680	2830	150	150	3.58×10^{-6}	1.58×10^{-6}	0.44

Table S22 (a). Impedance and current values extracted from AC impedance spectroscopy and potentiostatic polarization measurements of three lithium–lithium symmetric cells for POEM / PLiMTFSI / LiTf blend; [EO]:[LiMTFSI]:[LiTf] = 10:0.50:0.50 (molar ratio) at 60 °C.

	$^{b}R_1$ (Ohms)	$^{b}R_2$ (Ohms)	$^{c}R_1^*$ (Ohms)	$^{c}R_2^*$ (Ohms)	$^{d}R_0$ (Ohms)	$^{e}R_{SS}$ (Ohms)	$^{f}I_0$ (A)	I_{SS} (A)	t_{Li+}
^{a}Cell A	2.94×10^{4}	3.15×10^{4}	2.96×10^{4}	3.24×10^{4}	2120	2800	3.17×10^{-7}	1.59×10^{-7}	0.50
^{a}Cell B	2.95×10^{4}	3.23×10^{4}	3.05×10^{4}	3.26×10^{4}	2790	2100	3.10×10^{-7}	1.55×10^{-7}	0.50
^{a}Cell C	2.94×10^{4}	3.28×10^{4}	3.04×10^{4}	3.36×10^{4}	3430	3210	3.05×10^{-7}	1.53×10^{-7}	0.50

Table S22 (b). Impedance and current values extracted from AC impedance spectroscopy and potentiostatic polarization measurements of three lithium–lithium symmetric cells for POEM / PLiMTFSI / LiTf blend; [EO]:[LiMTFSI]:[LiTf] = 10:0.50:0.50 (molar ratio) at 100 °C.

	$^{b}R_1$ (Ohms)	$^{b}R_2$ (Ohms)	$^{c}R_1^*$ (Ohms)	$^{c}R_2^*$ (Ohms)	$^{d}R_0$ (Ohms)	$^{e}R_{SS}$ (Ohms)	$^{f}I_0$ (A)	I_{SS} (A)	t_{Li+}
^{a}Cell A	3550	3780	3590	3850	230	260	2.64×10^{-6}	1.35×10^{-6}	0.51
^{a}Cell B	3510	3710	3550	3810	200	260	2.70×10^{-6}	1.35×10^{-6}	0.50
^{a}Cell C	3530	3880	3570	4000	350	430	2.58×10^{-6}	1.31×10^{-6}	0.51

Table S23 (a). Impedance and current values extracted from AC impedance spectroscopy and potentiostatic polarization measurements of three lithium–lithium symmetric cells for POEM / PLiMTFSI / LiTf blend; [EO]:[LiMTFSI]:[LiTf] = 10:0.60:0.40 (molar ratio) at 60 °C.

	$^{b}R_1$ (Ohms)	$^{b}R_2$ (Ohms)	$^{c}R_1^*$ (Ohms)	$^{c}R_2^*$ (Ohms)	$^{d}R_0$ (Ohms)	$^{e}R_{SS}$ (Ohms)	$^{f}I_0$ (A)	I_{SS} (A)	t_{Li+}
^{a}Cell A	4.19×10^{4}	4.63×10^{4}	4.25×10^{4}	4.66×10^{4}	4360	4130	2.16×10^{-7}	1.30×10^{-7}	0.60
^{a}Cell B	4.21×10^{4}	4.60×10^{4}	4.21×10^{4}	4.61×10^{4}	3900	4060	2.18×10^{-7}	1.31×10^{-7}	0.60
^{a}Cell C	4.11×10^{4}	4.51×10^{4}	4.23×10^{4}	4.64×10^{4}	4070	4140	2.22×10^{-7}	1.35×10^{-7}	0.61

Table S23 (b). Impedance and current values extracted from AC impedance spectroscopy and potentiostatic polarization measurements of three lithium–lithium symmetric cells for POEM / PLiMTFSI / LiTf blend; [EO]:[LiMTFSI]:[LiTf] = 10:0.60:0.40 (molar ratio) at 100 °C.

	$^{b}R_1$ (Ohms)	$^{b}R_2$ (Ohms)	$^{c}R_1^*$ (Ohms)	$^{c}R_2^*$ (Ohms)	$^{d}R_0$ (Ohms)	$^{e}R_{SS}$ (Ohms)	$^{f}I_0$ (A)	I_{SS} (A)	t_{Li+}
^{a}Cell A	4470	4860	4550	5110	390	560	2.06×10^{-6}	1.28×10^{-6}	0.62
^{a}Cell B	4530	4900	4520	5000	370	480	2.04×10^{-6}	1.27×10^{-6}	0.62
^{a}Cell C	4500	4840	4520	5010	340	490	2.06×10^{-6}	1.30×10^{-6}	0.63

Table S24 (a). Impedance and current values extracted from AC impedance spectroscopy and potentiostatic polarization measurements of three lithium–lithium symmetric cells for POEM / PLiMTFSI / LiTf blend; [EO]:[LiMTFSI]:[LiTf] = 10:0.80:0.20 (molar ratio) at 60 °C.

	$^{b}R_1$ (Ohms)	$^{b}R_2$ (Ohms)	$^{c}R_1^*$ (Ohms)	$^{c}R_2^*$ (Ohms)	$^{d}R_0$ (Ohms)	$^{e}R_{SS}$ (Ohms)	$^{f}I_0$ (A)	I_{SS} (A)	t_{Li+}
^{a}Cell A	4.40×10^{4}	4.81×10^{4}	4.43×10^{4}	4.90×10^{4}	4120	4690	2.08×10^{-7}	1.41×10^{-7}	0.68
^{a}Cell B	4.41×10^{4}	4.88×10^{4}	4.46×10^{4}	4.92×10^{4}	4730	4560	2.05×10^{-7}	1.39×10^{-7}	0.68
^{a}Cell C	4.39×10^{4}	4.88×10^{4}	4.53×10^{4}	4.98×10^{4}	4920	4480	2.05×10^{-7}	1.39×10^{-7}	0.68

Table S24 (b). Impedance and current values extracted from AC impedance spectroscopy and potentiostatic polarization measurements of three lithium–lithium symmetric cells for POEM / PLiMTFSI / LiTf blend; [EO]:[LiMTFSI]:[LiTf] = 10:0.80:0.20 (molar ratio) at 100 °C.

	$^{b}R_1$ (Ohms)	$^{b}R_2$ (Ohms)	$^{c}R_1^*$ (Ohms)	$^{c}R_2^*$ (Ohms)	$^{d}R_0$ (Ohms)	$^{e}R_{SS}$ (Ohms)	$^{f}I_0$ (A)	I_{SS} (A)	t_{Li+}
^{a}Cell A	5100	5550	5230	5550	450	320	1.80×10^{-6}	1.23×10^{-6}	0.68
^{a}Cell B	5050	5540	5240	5560	490	320	1.81×10^{-6}	1.23×10^{-6}	0.68
^{a}Cell C	5130	5540	5270	5590	410	320	1.81×10^{-6}	1.23×10^{-6}	0.68

8.5 Measurement conducted at 60 °C for POEM / PLiMTFSI / LiTf blends

8.5.1 POEM / PLiMTFSI / LiTf blend; [EO]:[LiMTFSI]:[LiTf] = 10:0.05:0.95 (molar ratio)

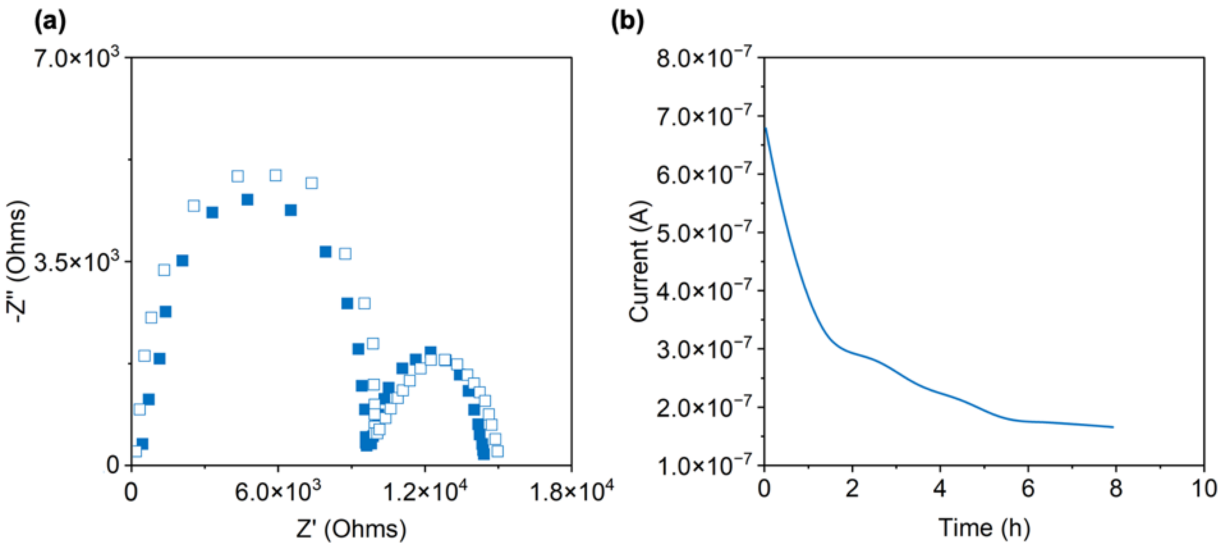

Figure S133. (a) Z'' as a function of Z' for lithium–lithium symmetric cell A before (filled squares) and after polarization (hollow squares). (b) Current *vs.* time during cell polarization.

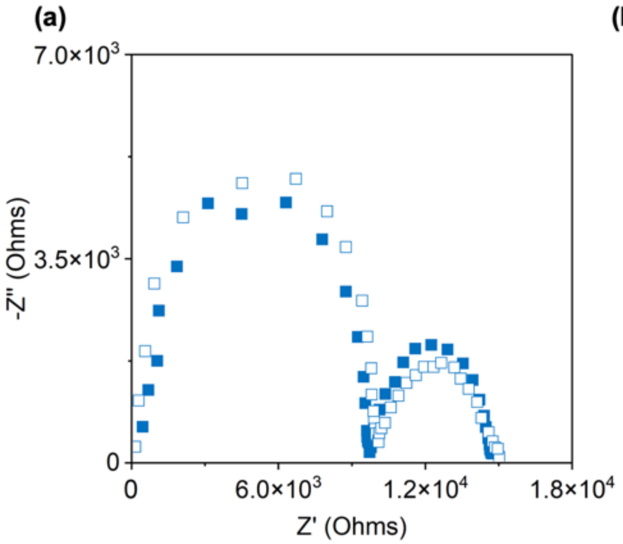

Figure S134. (a) Z'' as a function of Z' for lithium–lithium symmetric cell B before (filled squares) and after polarization (hollow squares). (b) Current *vs.* time during cell polarization.

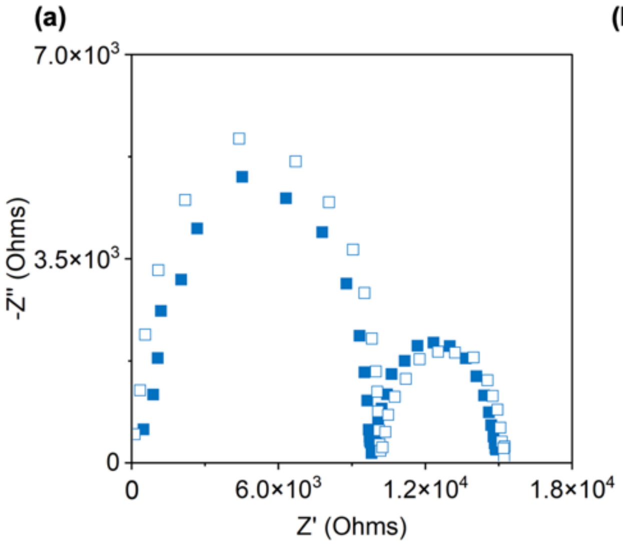

Figure S135. (a) Z'' as a function of Z' for lithium–lithium symmetric cell C before (filled squares) and after polarization (hollow squares). (b) Current *vs.* time during cell polarization.

8.5.2 POEM / PLiMTFSI / LiTf blend; [EO]:[LiMTFSI]:[LiTf] = 10:0.15:0.85 (molar ratio)

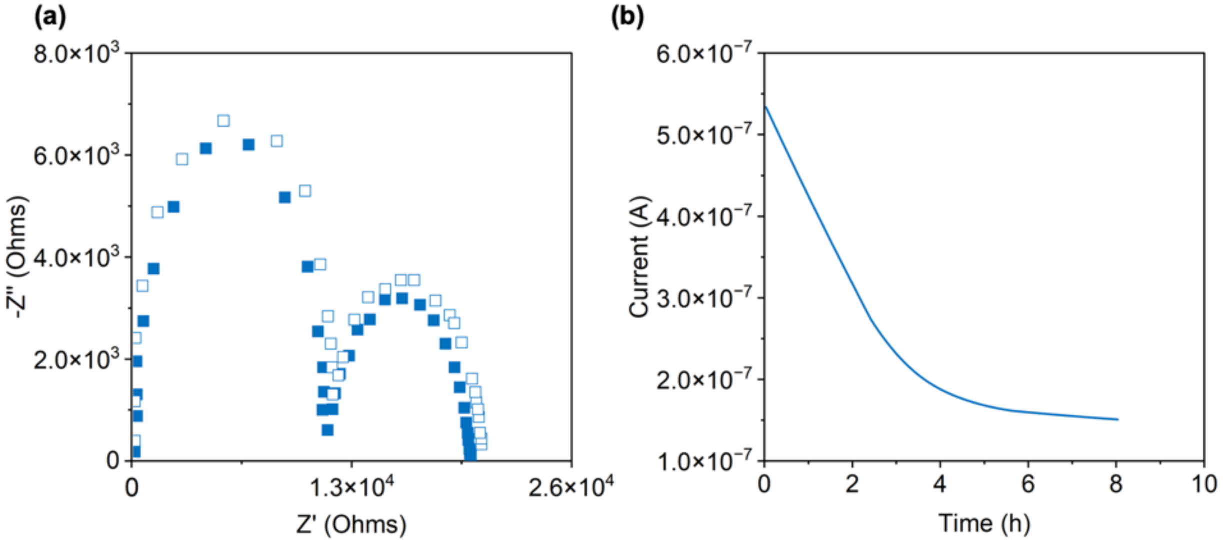

Figure S136. (a) Z″ as a function of Z′ for lithium–lithium symmetric cell A before (filled squares) and after polarization (hollow squares). (b) Current *vs.* time during cell polarization.

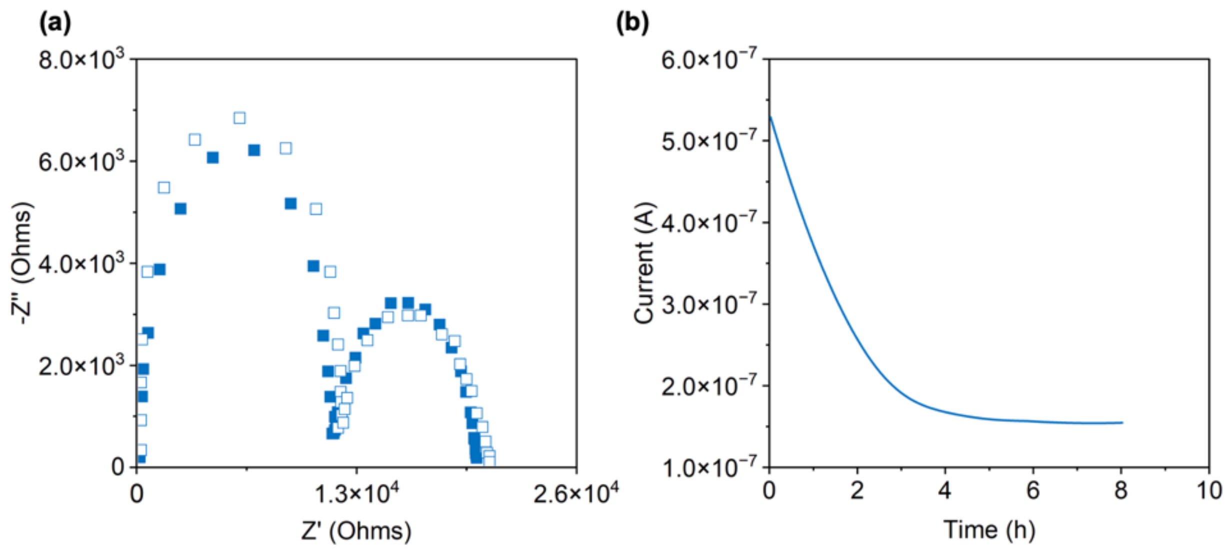

Figure S137. (a) Z'' as a function of Z' for lithium–lithium symmetric cell B before (filled squares) and after polarization (hollow squares). (b) Current *vs.* time during cell polarization.

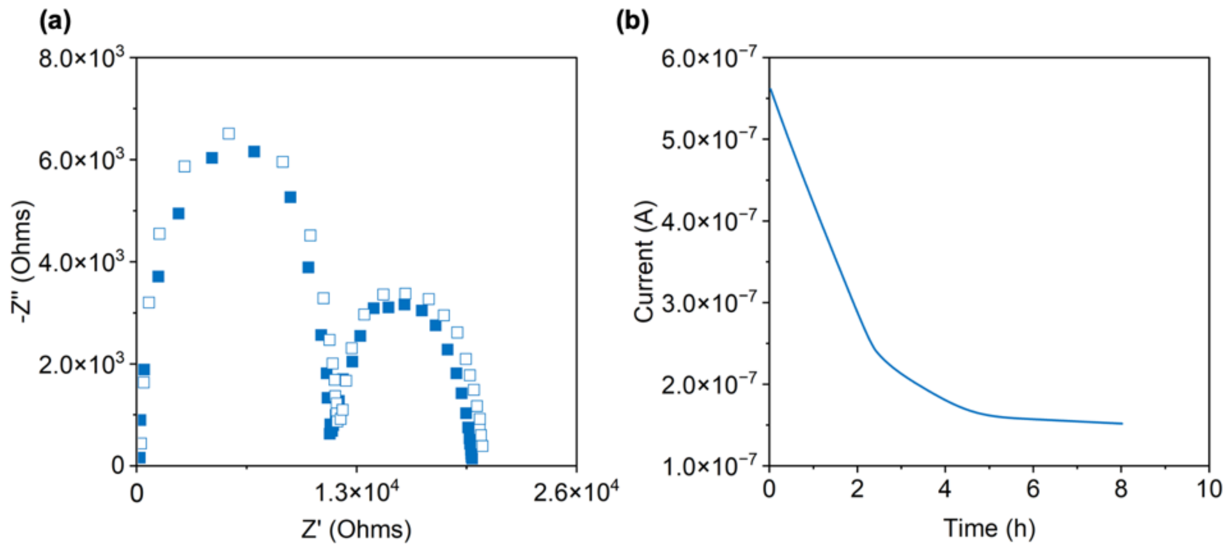

Figure S138. (a) Z'' as a function of Z' for lithium–lithium symmetric cell C before (filled squares) and after polarization (hollow squares). (b) Current *vs.* time during cell polarization.

8.5.3 POEM / PLiMTFSI / LiTf blend; [EO]:[LiMTFSI]:[LiTf] = 10:0.25:0.75 (molar ratio)

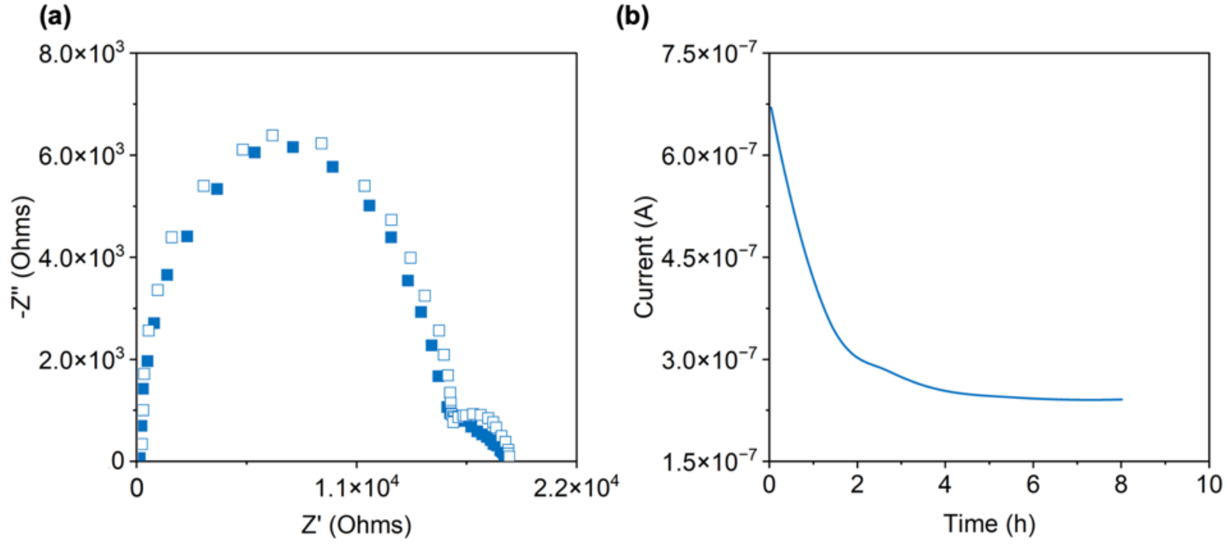

Figure S139. (a) Z″ as a function of Z′ for lithium–lithium symmetric cell A before (filled squares) and after polarization (hollow squares). (b) Current *vs.* time during cell polarization.

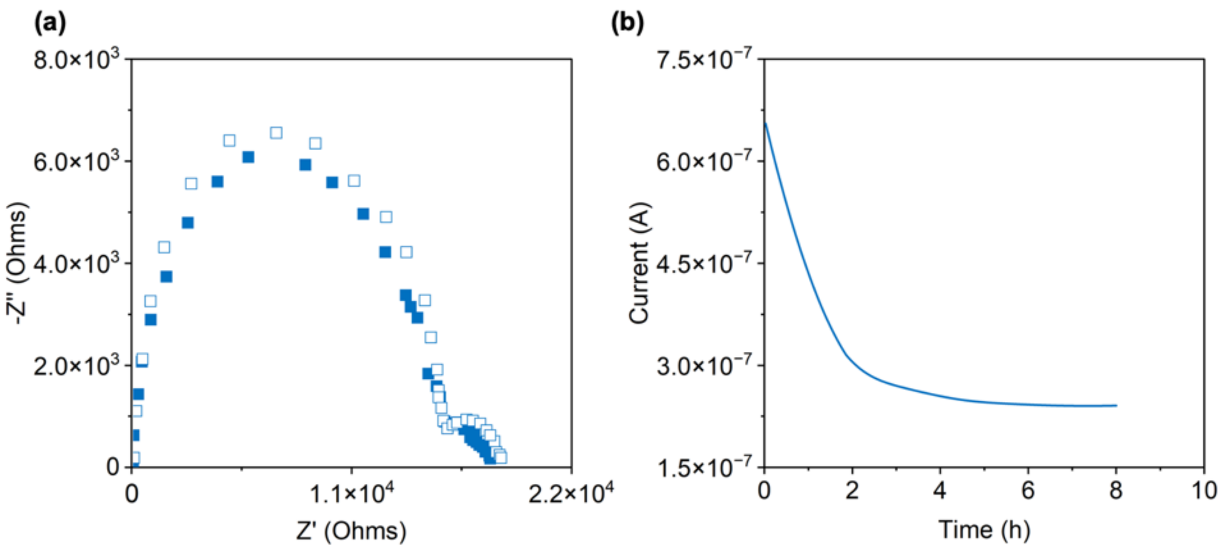

Figure S140. (a) Z'' as a function of Z' for lithium–lithium symmetric cell B before (filled squares) and after polarization (hollow squares). (b) Current *vs.* time during cell polarization.

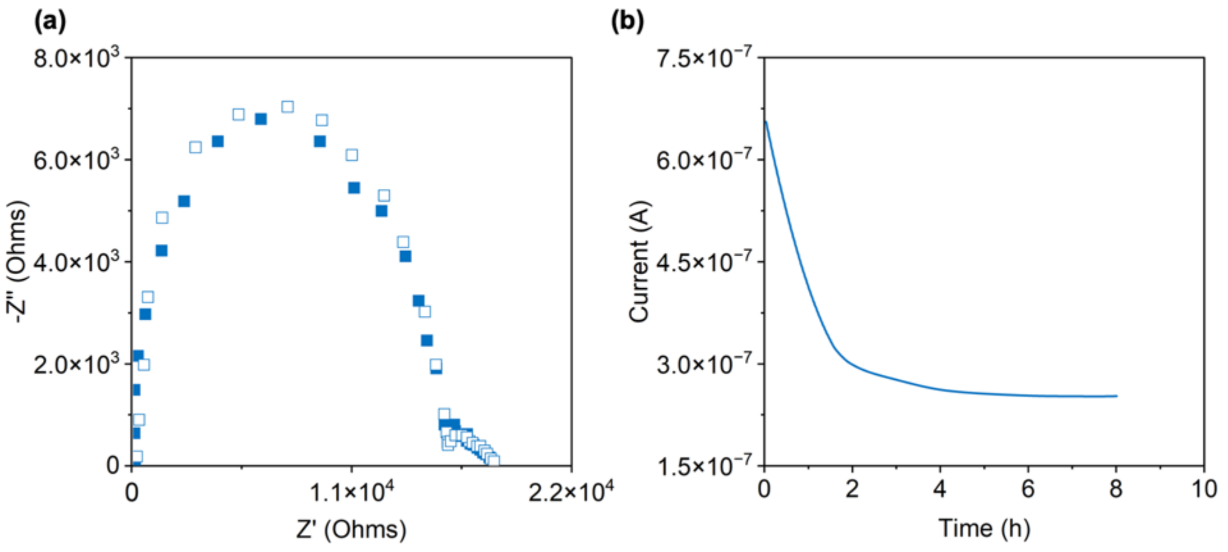

Figure S141. (a) Z'' as a function of Z' for lithium–lithium symmetric cell C before (filled squares) and after polarization (hollow squares). (b) Current *vs.* time during cell polarization.

8.5.4 POEM / PLiMTFSI / LiTf blend; [EO]:[LiMTFSI]:[LiTf] = 10:0.40:0.60 (molar ratio)

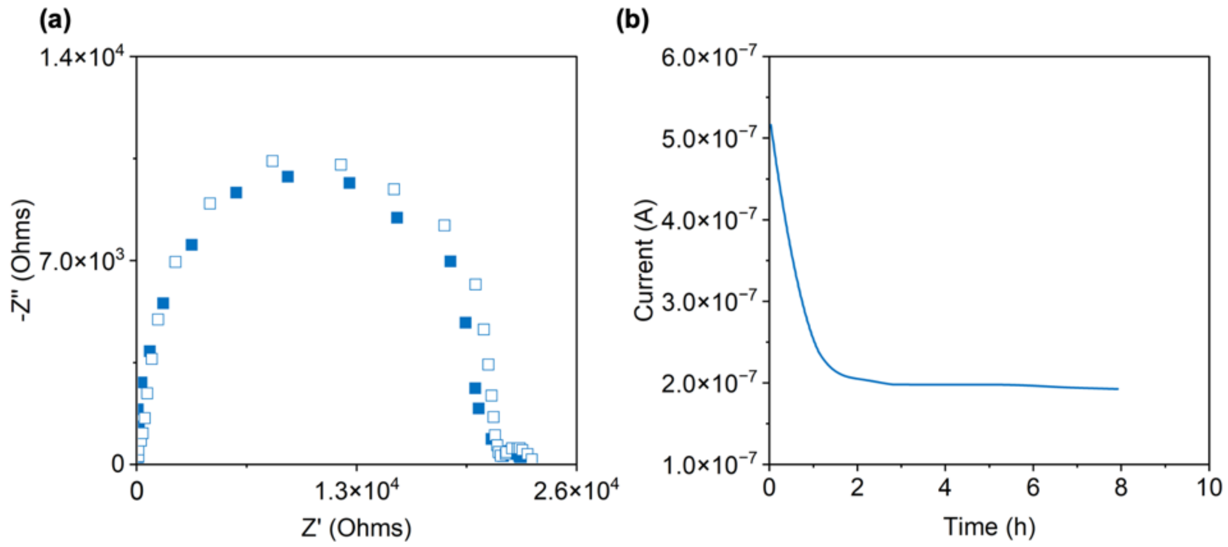

Figure S142. (a) Z'' as a function of Z' for lithium–lithium symmetric cell A before (filled squares) and after polarization (hollow squares). (b) Current *vs.* time during cell polarization.

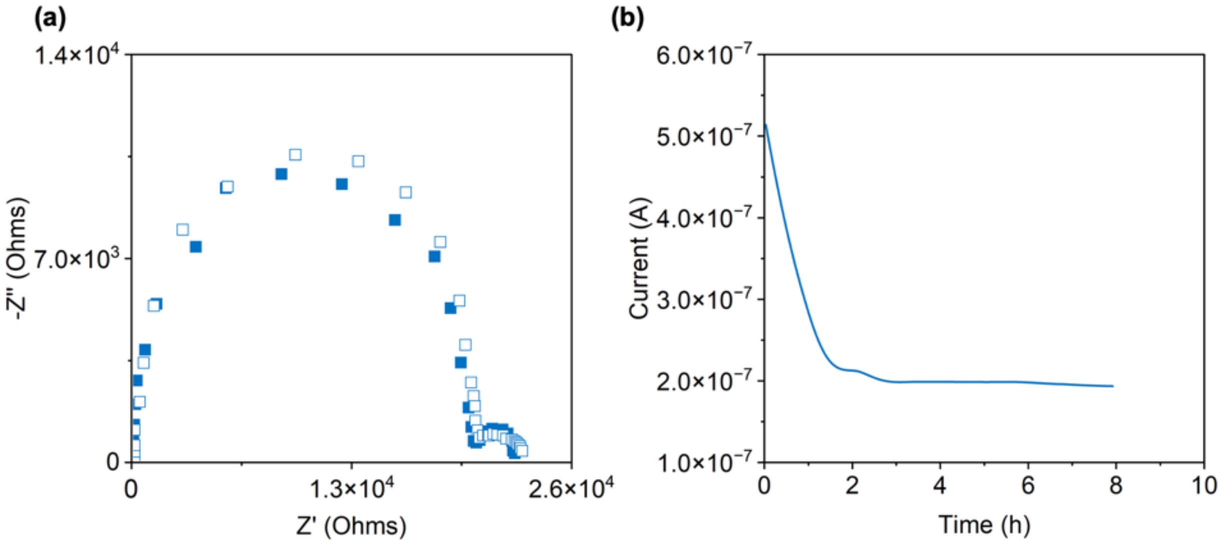

Figure S143. (a) Z'' as a function of Z' for lithium–lithium symmetric cell B before (filled squares) and after polarization (hollow squares). (b) Current *vs.* time during cell polarization.

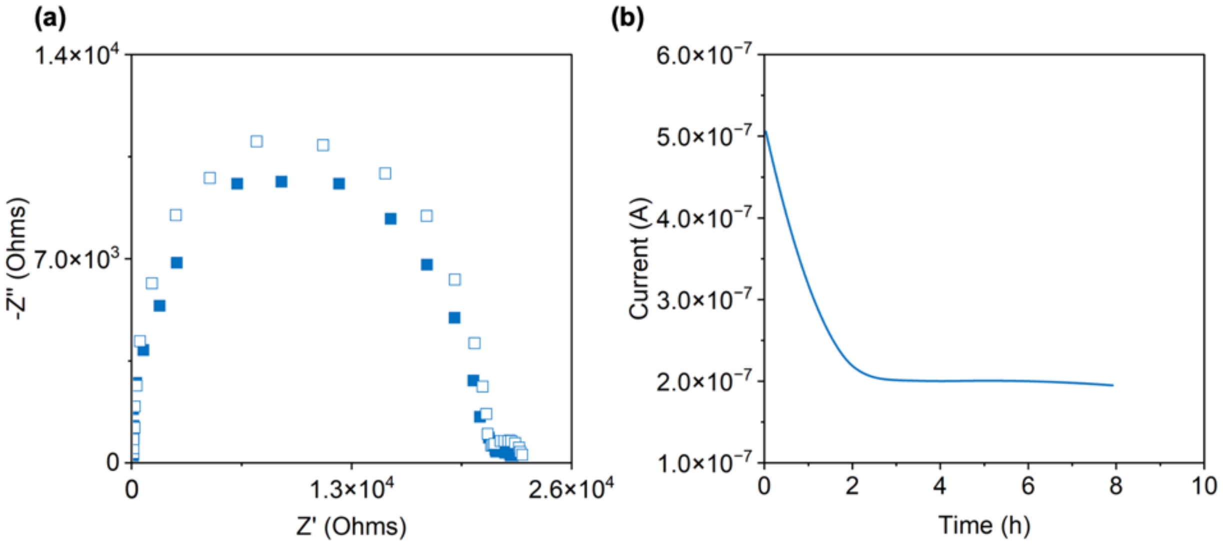

Figure S144. (a) Z'' as a function of Z' for lithium–lithium symmetric cell C before (filled squares) and after polarization (hollow squares). (b) Current *vs.* time during cell polarization.

8.5.5 POEM / PLiMTFSI / LiTf blend; [EO]:[LiMTFSI]:[LiTf] = 10:0.50:0.50 (molar ratio)

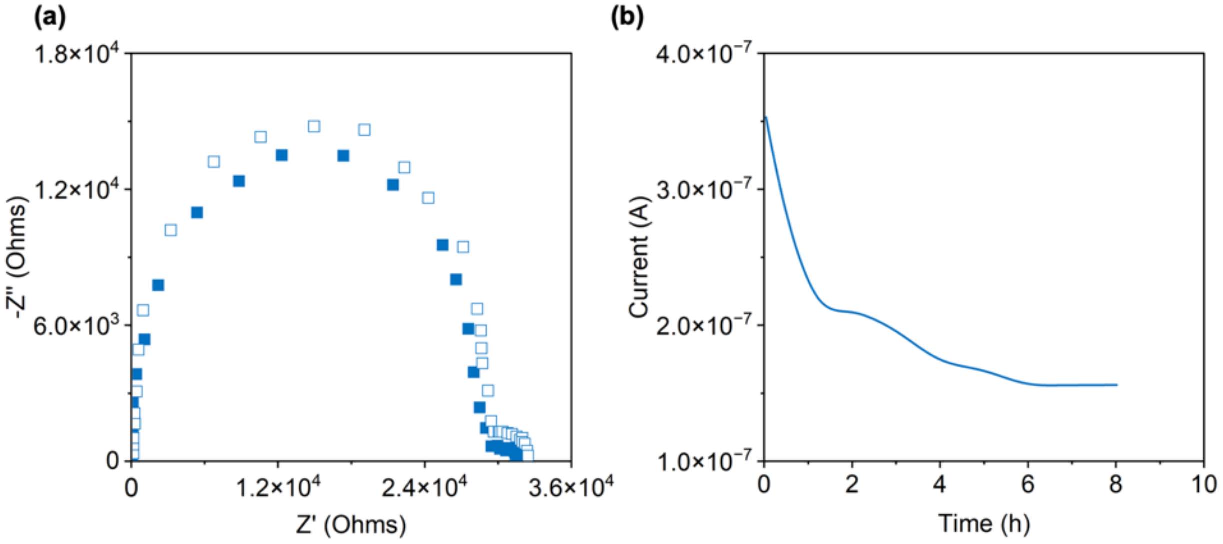

Figure S145. (a) Z'' as a function of Z' for lithium–lithium symmetric cell A before (filled squares) and after polarization (hollow squares). (b) Current *vs.* time during cell polarization.

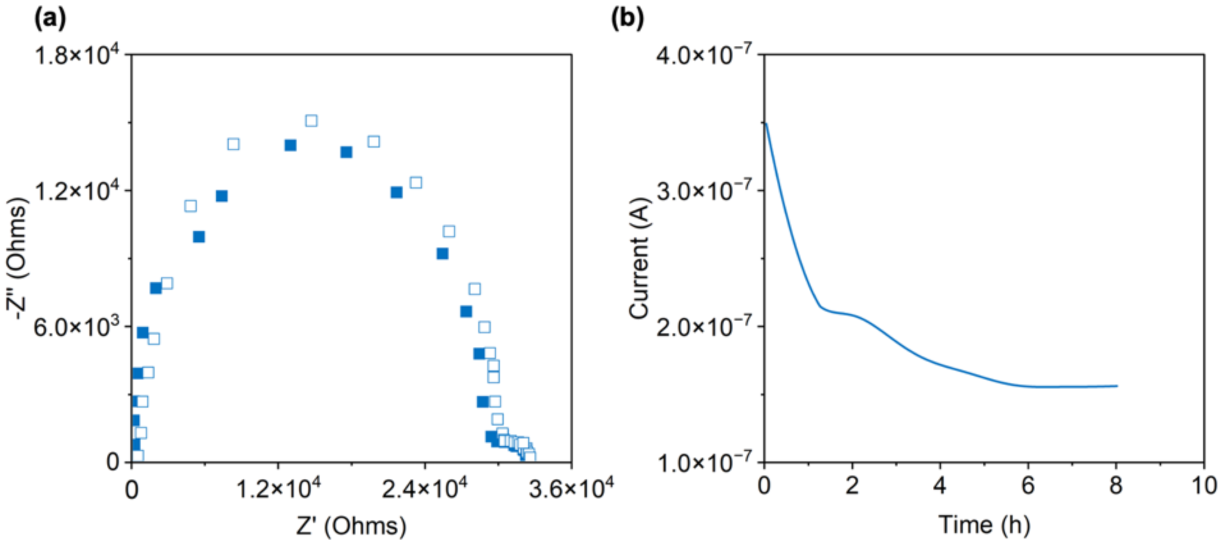

Figure S146. (a) Z'' as a function of Z' for lithium–lithium symmetric cell B before (filled squares) and after polarization (hollow squares). (b) Current *vs.* time during cell polarization.

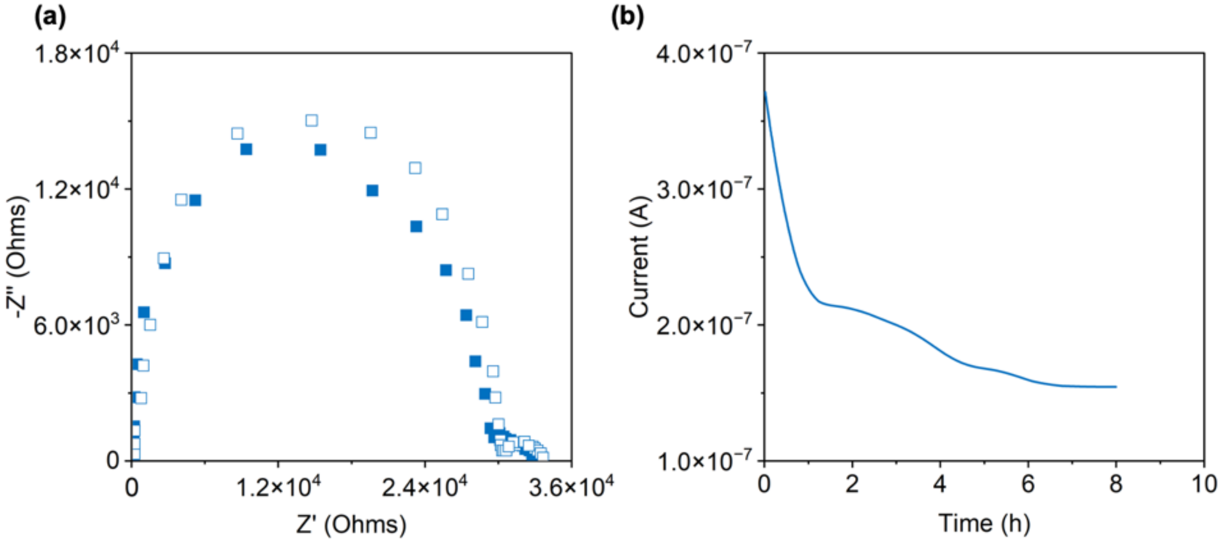

Figure S147. (a) Z'' as a function of Z' for lithium–lithium symmetric cell C before (filled squares) and after polarization (hollow squares). (b) Current *vs.* time during cell polarization.

8.5.6 POEM / PLiMTFSI / LiTf blend; [EO]:[LiMTFSI]:[LiTf] = 10:0.60:0.40 (molar ratio)

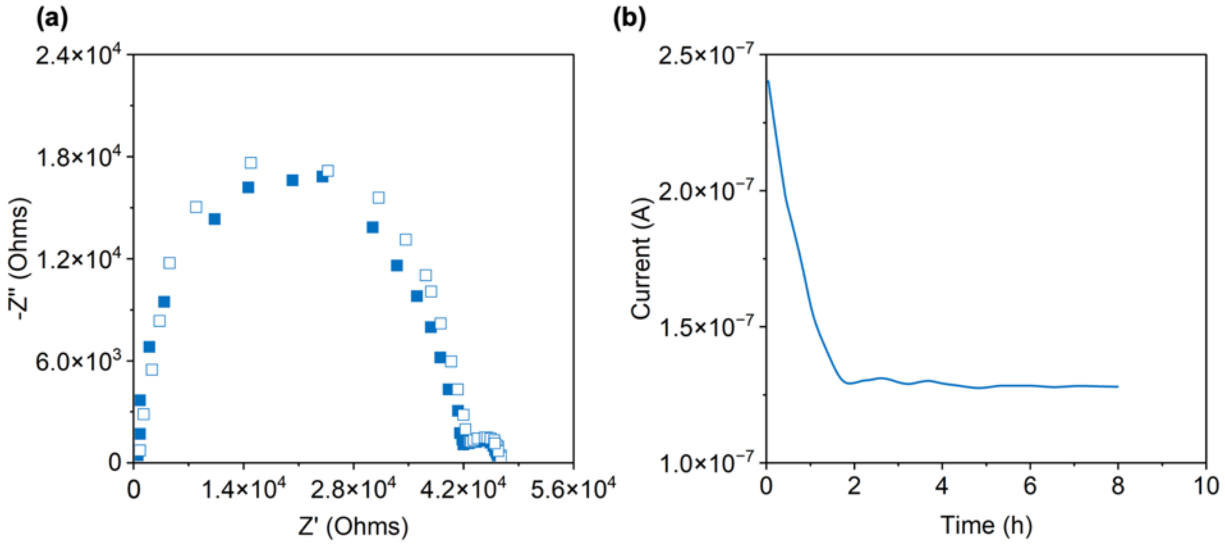

Figure S148. (a) Z″ as a function of Z′ for lithium–lithium symmetric cell A before (filled squares) and after polarization (hollow squares). (b) Current *vs.* time during cell polarization.

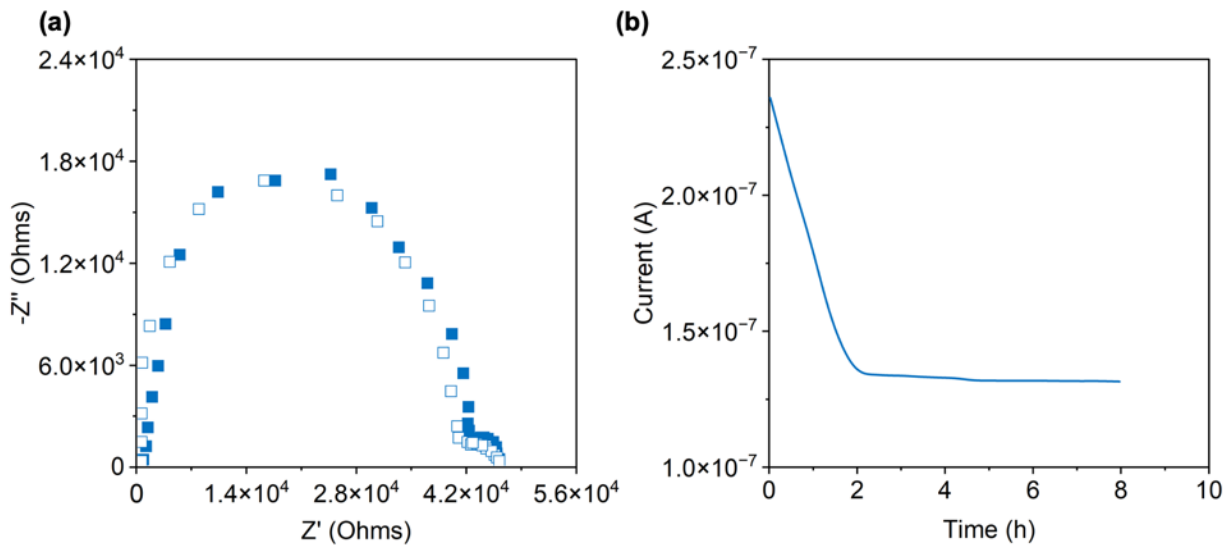

Figure S149. (a) Z'' as a function of Z' for lithium–lithium symmetric cell B before (filled squares) and after polarization (hollow squares). (b) Current *vs.* time during cell polarization.

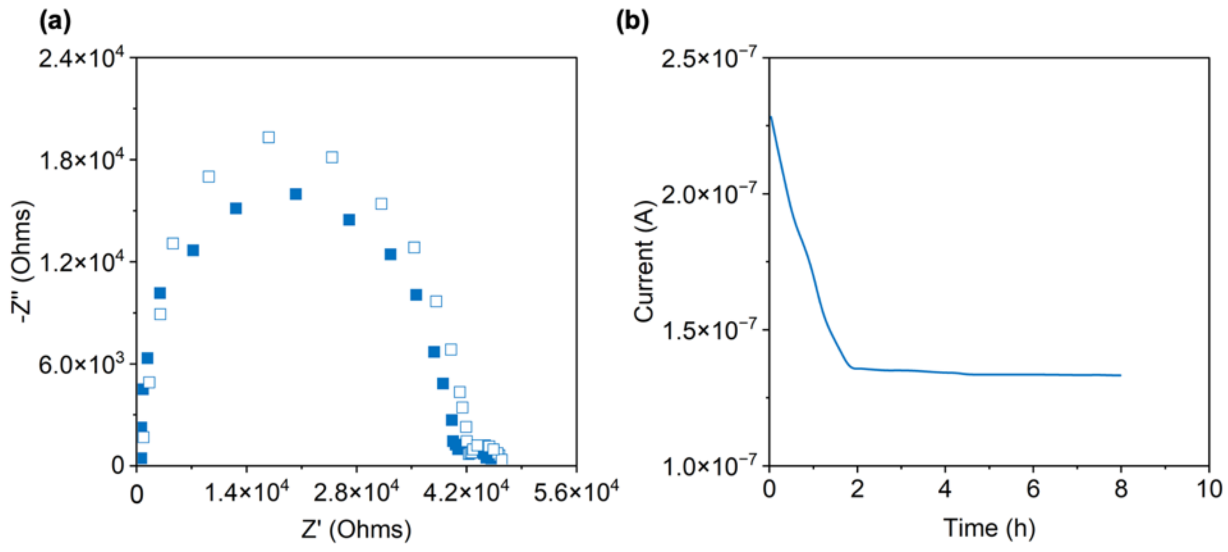

Figure S150. (a) Z'' as a function of Z' for lithium–lithium symmetric cell C before (filled squares) and after polarization (hollow squares). (b) Current *vs.* time during cell polarization.

8.5.7 POEM / PLiMTFSI / LiTf blend; [EO]:[LiMTFSI]:[LiTf] = 10:0.80:0.20 (molar ratio)

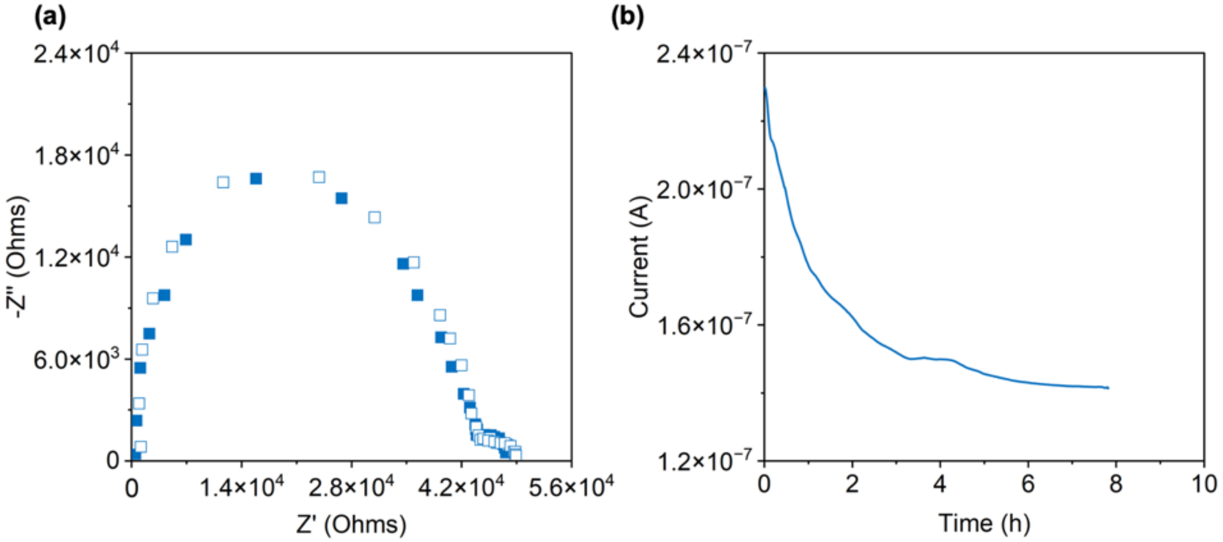

Figure S151. (a) Z″ as a function of Z′ for lithium–lithium symmetric cell A before (filled squares) and after polarization (hollow squares). (b) Current *vs.* time during cell polarization.

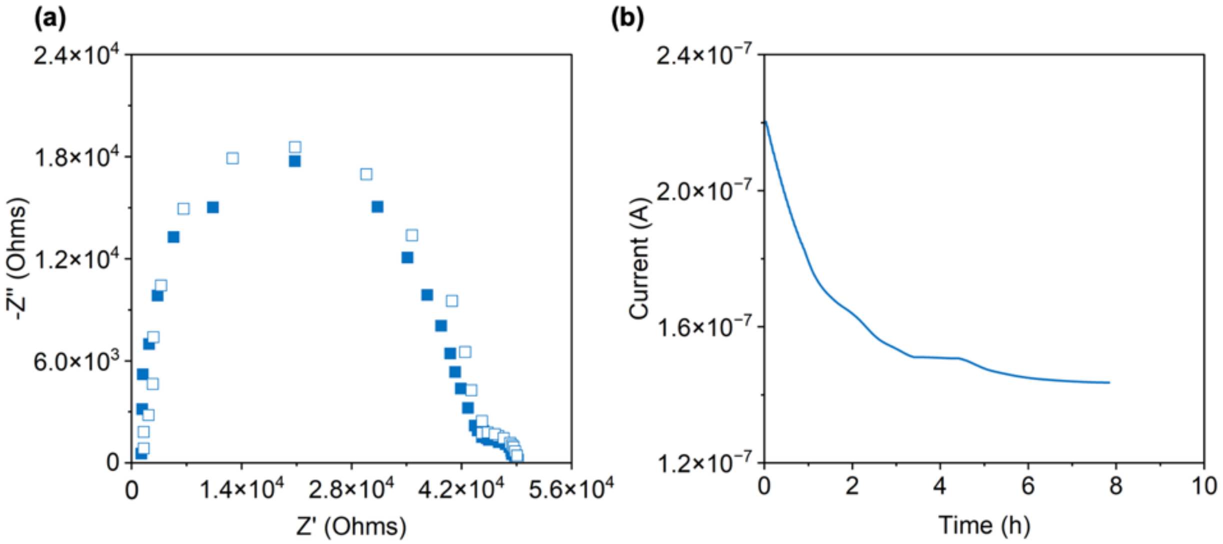

Figure S152. (a) Z'' as a function of Z' for lithium–lithium symmetric cell B before (filled squares) and after polarization (hollow squares). (b) Current *vs.* time during cell polarization.

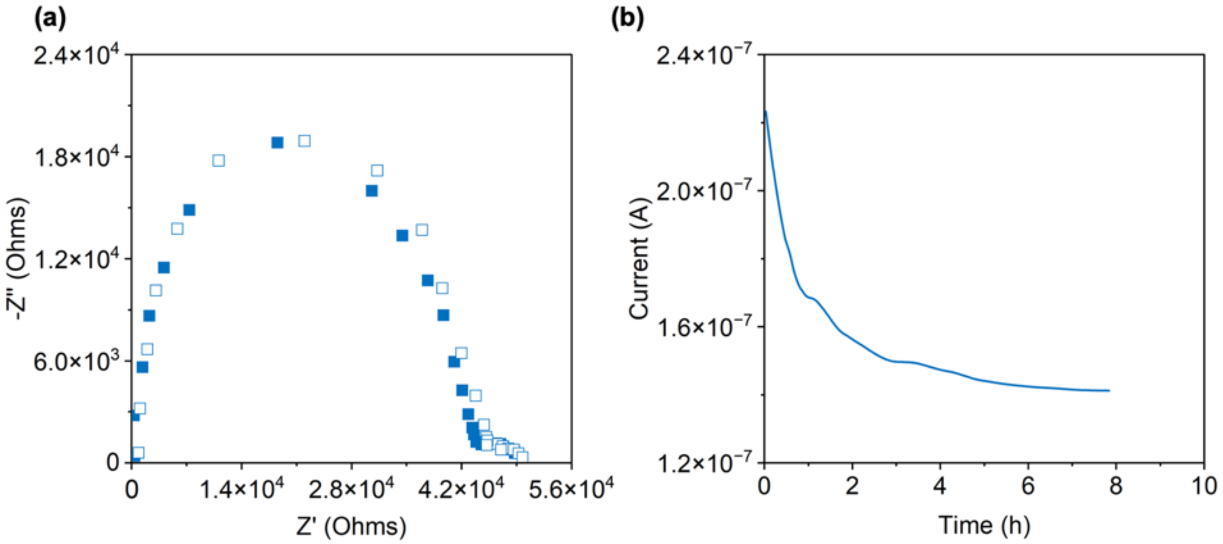

Figure S153. (a) Z'' as a function of Z' for lithium–lithium symmetric cell C before (filled squares) and after polarization (hollow squares). (b) Current *vs.* time during cell polarization.

8.6 Measurement conducted at 100 °C for POEM / PLiMTFSI / LiTf blends

8.6.1 POEM / PLiMTFSI / LiTf blend; [EO]:[LiMTFSI]:[LiTf] = 10:0.05:0.95 (molar ratio)

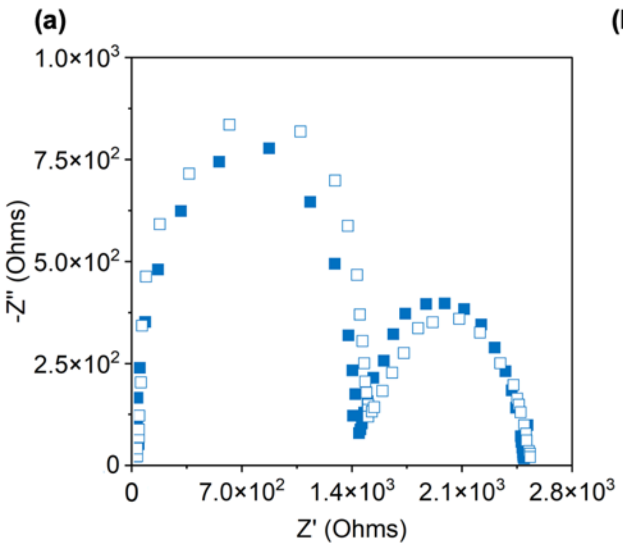

Figure S154. (a) Z'' as a function of Z' for lithium–lithium symmetric cell A before (filled squares) and after polarization (hollow squares). (b) Current *vs.* time during cell polarization.

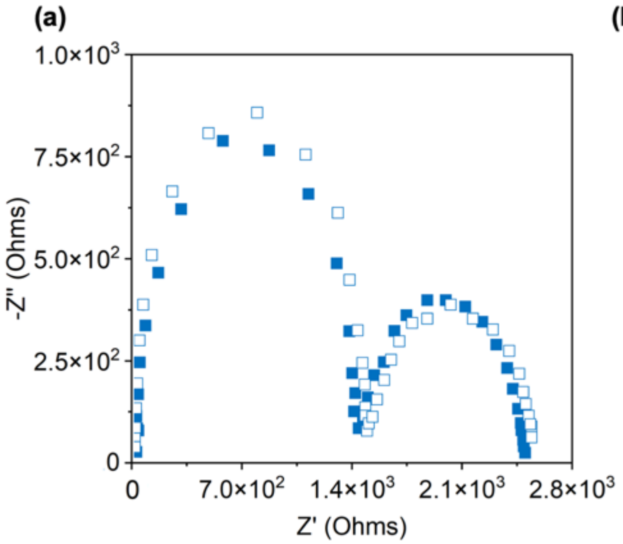

Figure S155. (a) Z'' as a function of Z' for lithium–lithium symmetric cell B before (filled squares) and after polarization (hollow squares). (b) Current *vs.* time during cell polarization.

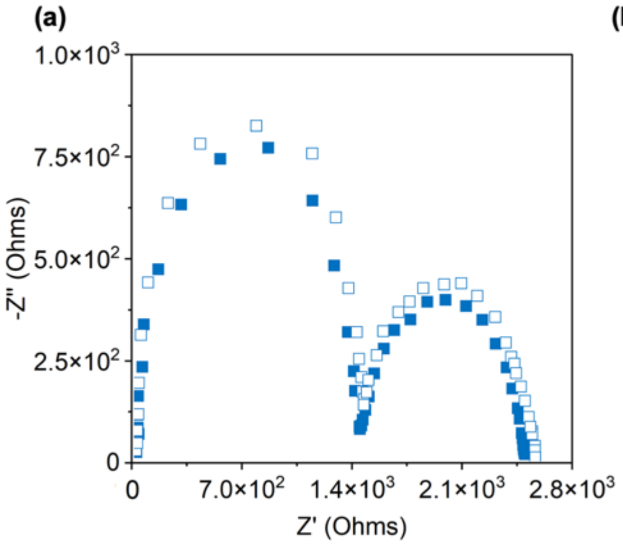

Figure S156. (a) Z'' as a function of Z' for lithium–lithium symmetric cell C before (filled squares) and after polarization (hollow squares). (b) Current *vs.* time during cell polarization.

8.6.2 POEM / PLiMTFSI / LiTf blend; [EO]:[LiMTFSI]:[LiTf] = 10:0.15:0.85 (molar ratio)

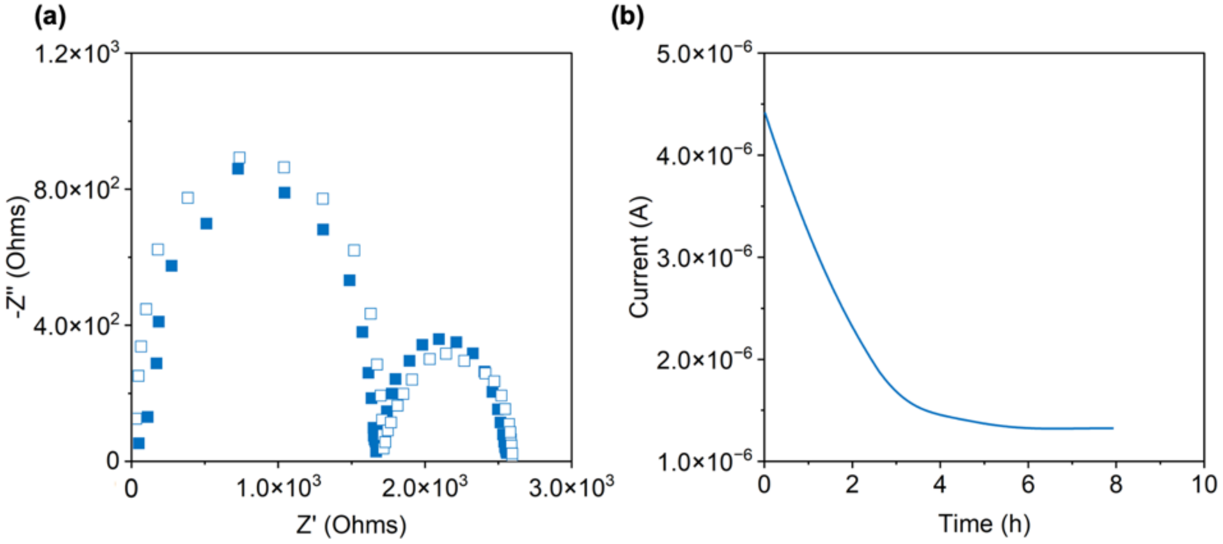

Figure S157. (a) Z″ as a function of Z′ for lithium–lithium symmetric cell A before (filled squares) and after polarization (hollow squares). (b) Current *vs.* time during cell polarization.

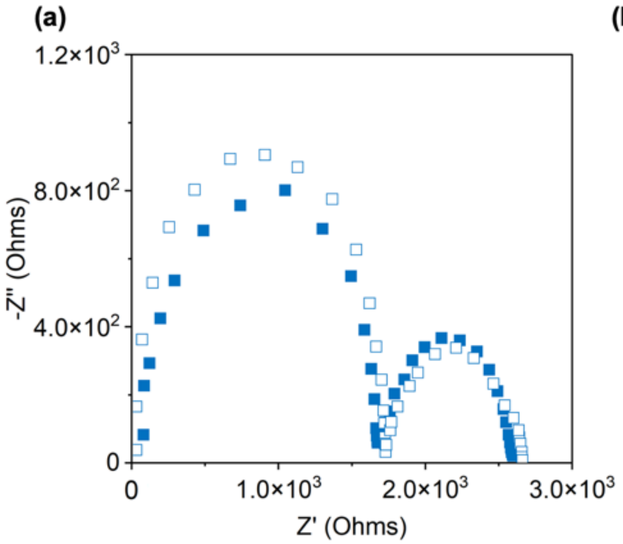

Figure S158. (a) Z'' as a function of Z' for lithium–lithium symmetric cell B before (filled squares) and after polarization (hollow squares). (b) Current *vs.* time during cell polarization.

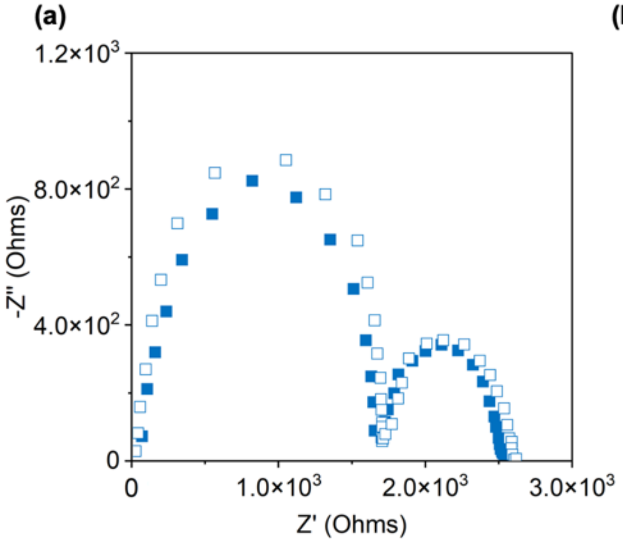

Figure S159. (a) Z'' as a function of Z' for lithium–lithium symmetric cell C before (filled squares) and after polarization (hollow squares). (b) Current *vs.* time during cell polarization.

8.6.3 POEM / PLiMTFSI / LiTf blend; [EO]:[LiMTFSI]:[LiTf] = 10:0.25:0.75 (molar ratio)

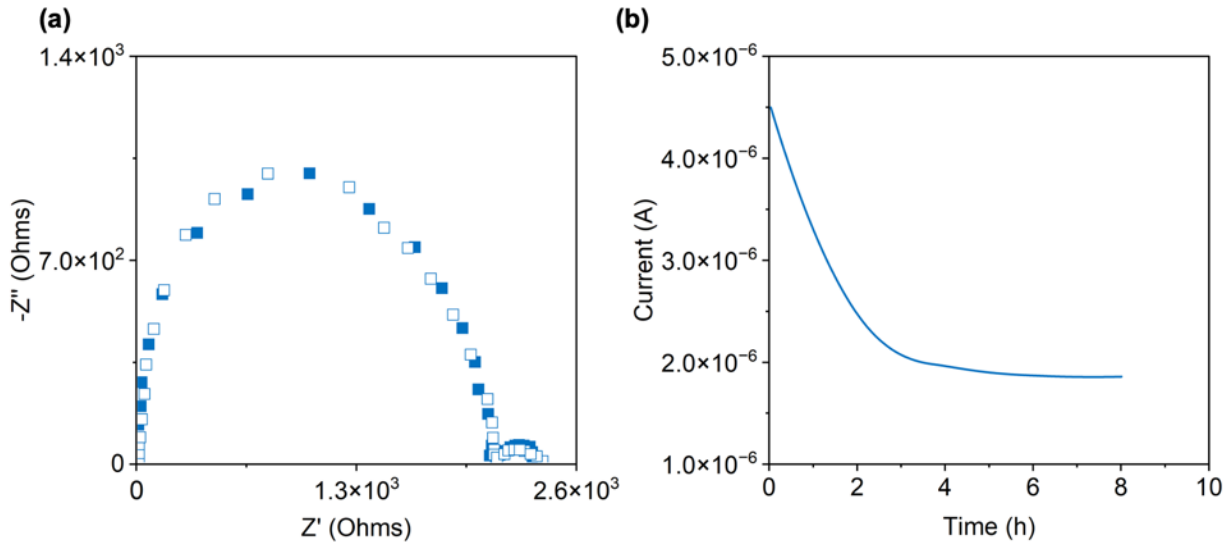

Figure S160. (a) Z″ as a function of Z′ for lithium–lithium symmetric cell A before (filled squares) and after polarization (hollow squares). (b) Current *vs.* time during cell polarization.

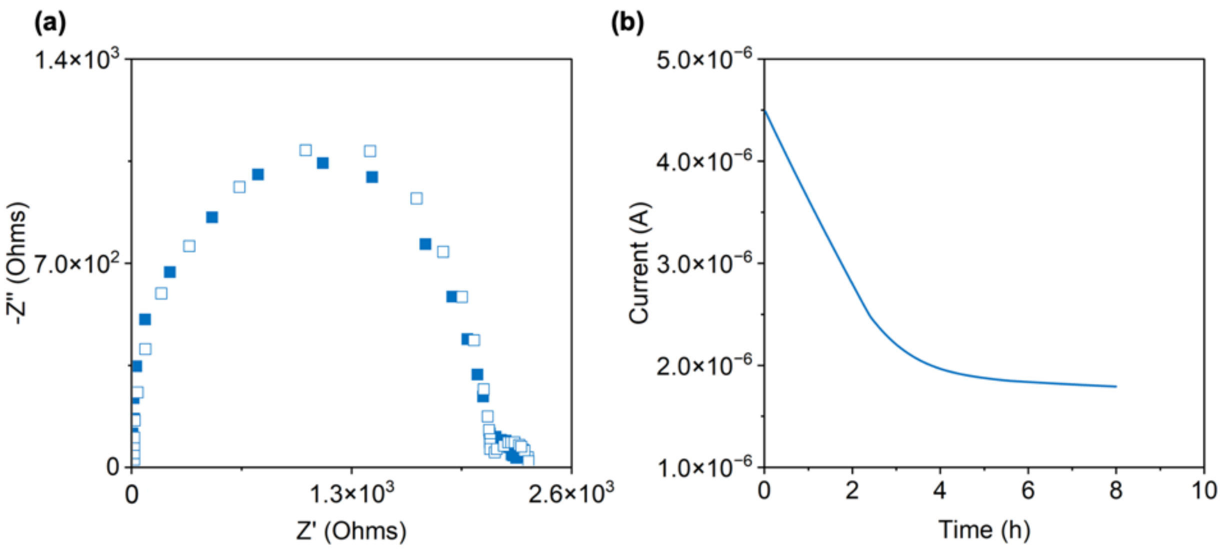

Figure S161. (a) Z'' as a function of Z' for lithium–lithium symmetric cell B before (filled squares) and after polarization (hollow squares). (b) Current *vs.* time during cell polarization.

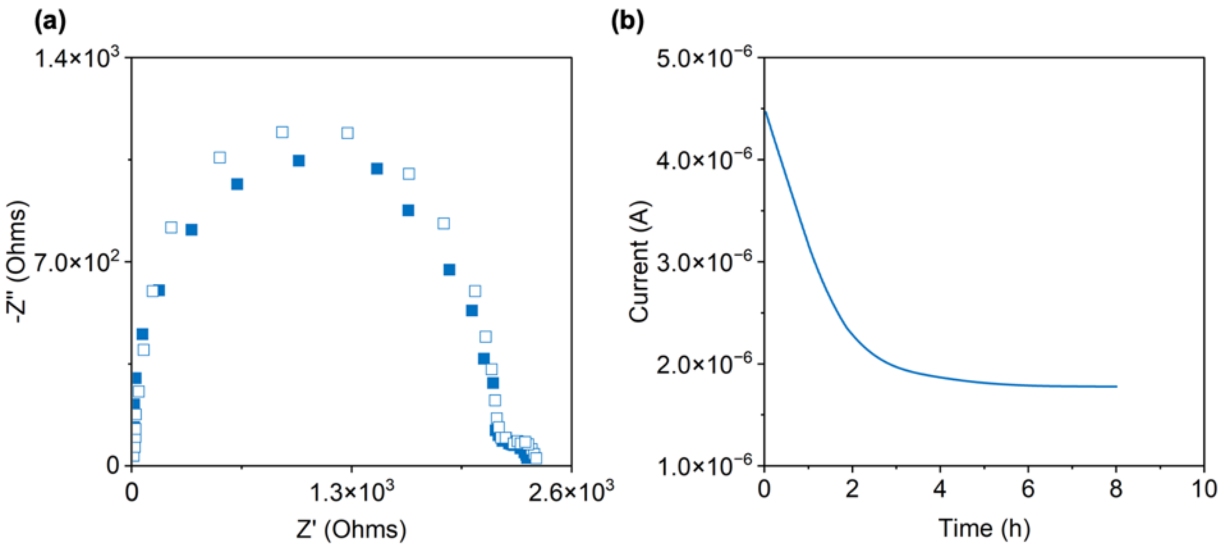

Figure S162. (a) Z'' as a function of Z' for lithium–lithium symmetric cell C before (filled squares) and after polarization (hollow squares). (b) Current *vs.* time during cell polarization.

8.6.4 POEM / PLiMTFSI / LiTf blend; [EO]:[LiMTFSI]:[LiTf] = 10:0.40:0.60 (molar ratio)

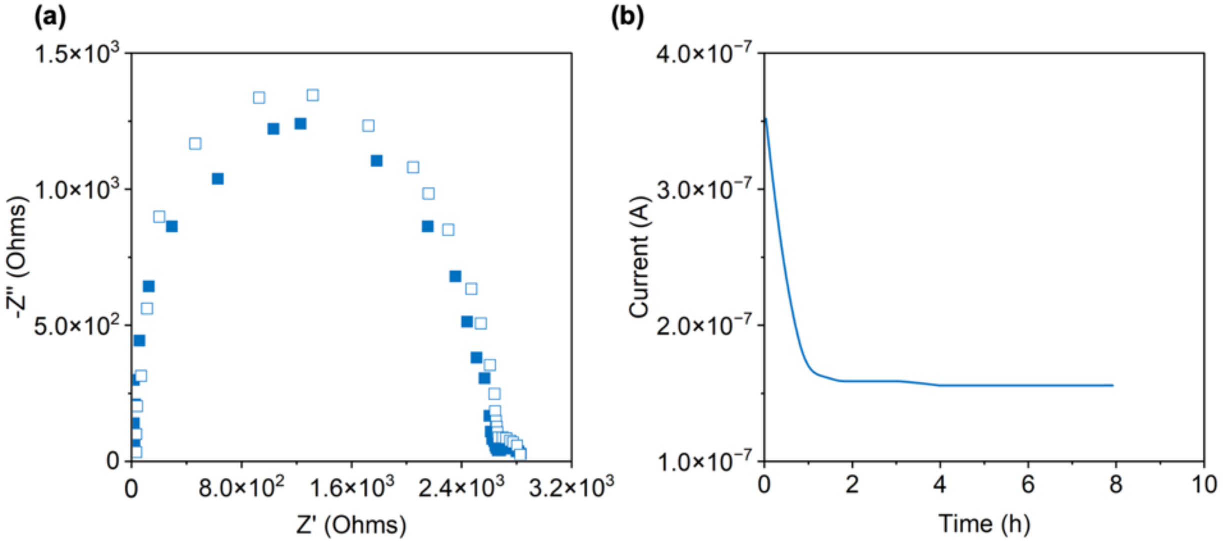

Figure S163. (a) Z'' as a function of Z' for lithium–lithium symmetric cell A before (filled squares) and after polarization (hollow squares). (b) Current *vs.* time during cell polarization.

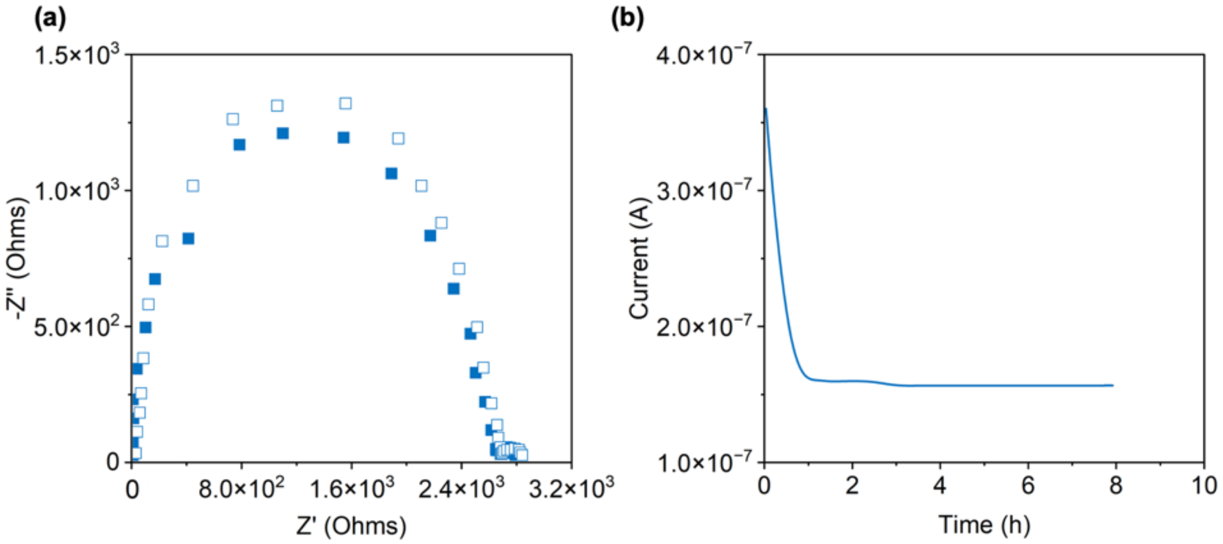

Figure S164. (a) Z'' as a function of Z' for lithium–lithium symmetric cell B before (filled squares) and after polarization (hollow squares). (b) Current *vs.* time during cell polarization.

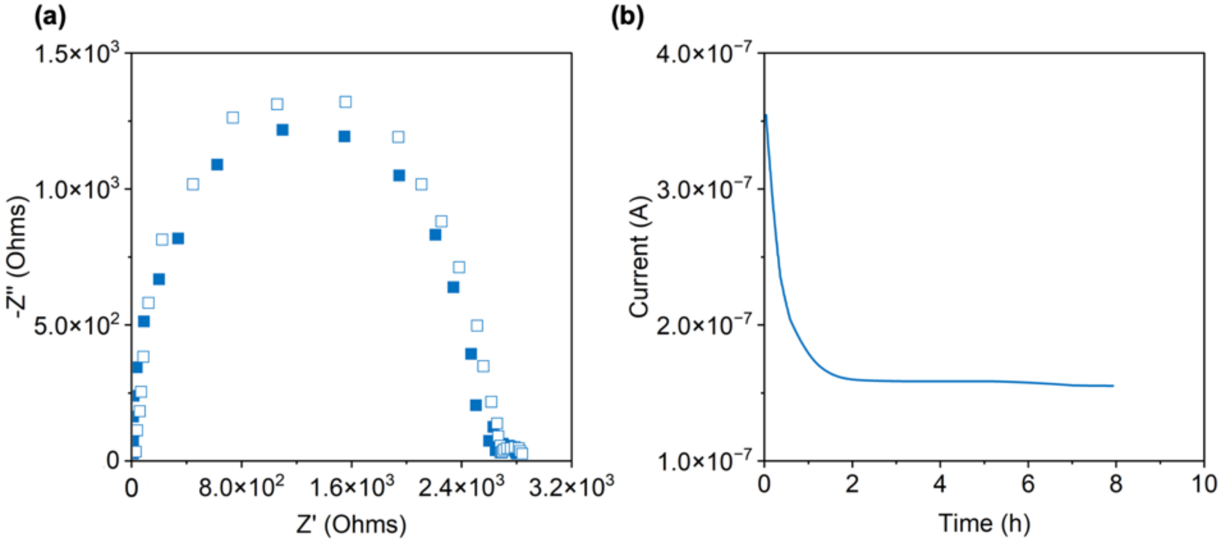

Figure S165. (a) Z'' as a function of Z' for lithium–lithium symmetric cell C before (filled squares) and after polarization (hollow squares). (b) Current *vs.* time during cell polarization.

8.6.5 POEM / PLiMTFSI / LiTf blend; [EO]:[LiMTFSI]:[LiTf] = 10:0.50:0.50 (molar ratio)

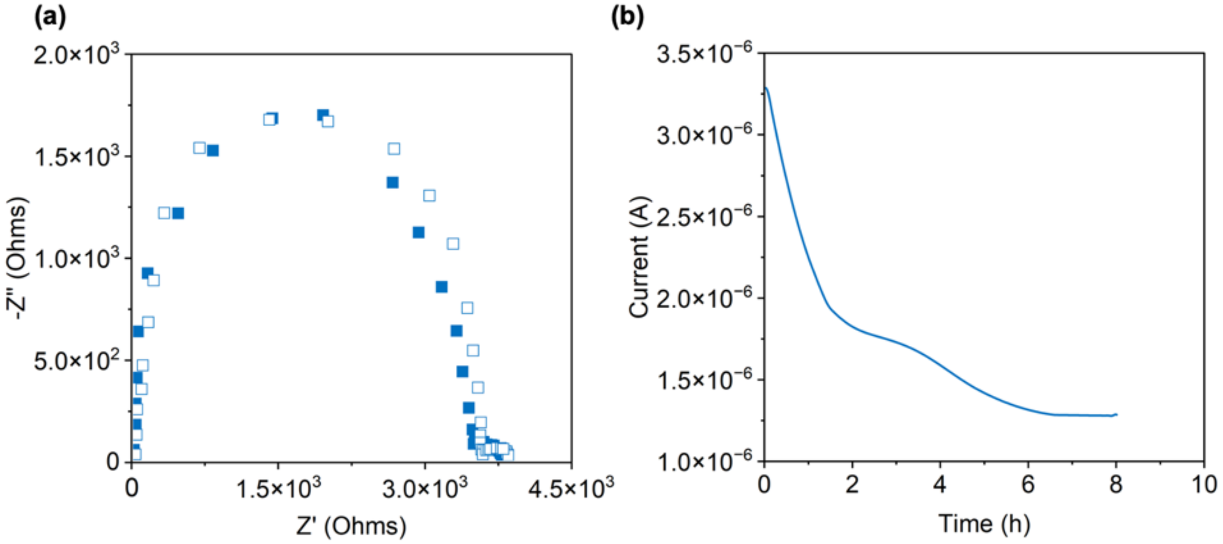

Figure S166. (a) Z″ as a function of Z′ for lithium–lithium symmetric cell A before (filled squares) and after polarization (hollow squares). (b) Current *vs.* time during cell polarization.

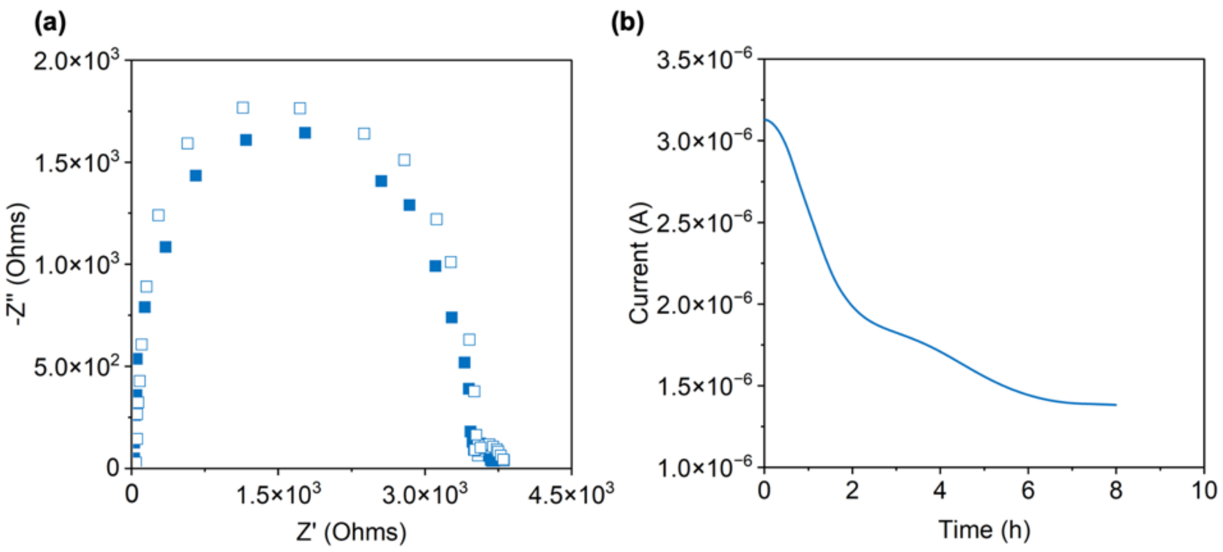

Figure S167. (a) Z'' as a function of Z' for lithium–lithium symmetric cell B before (filled squares) and after polarization (hollow squares). (b) Current *vs.* time during cell polarization.

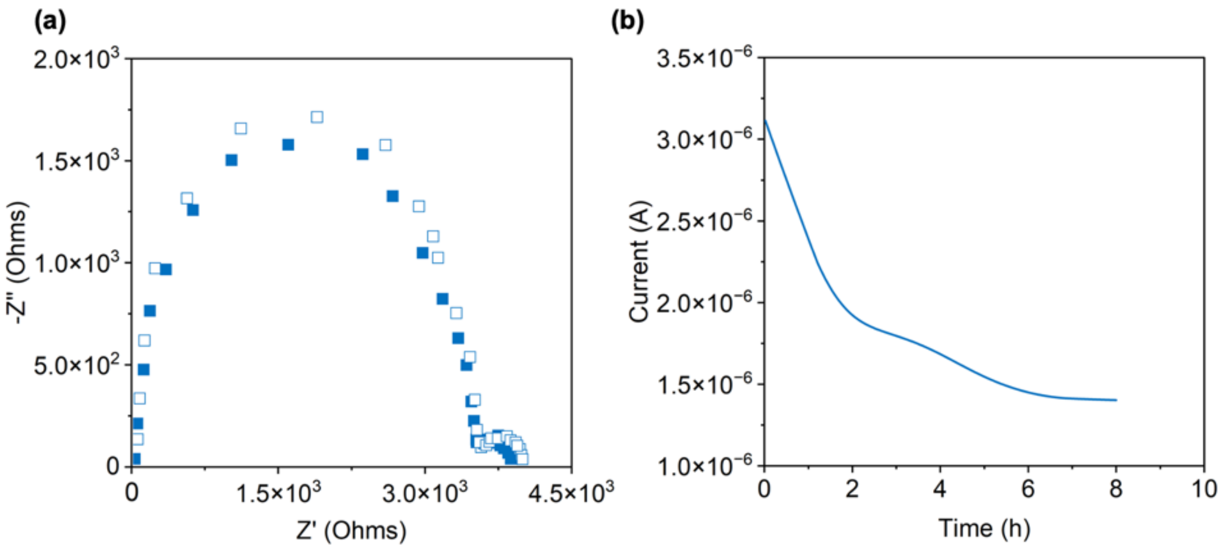

Figure S168. (a) Z'' as a function of Z' for lithium–lithium symmetric cell C before (filled squares) and after polarization (hollow squares). (b) Current *vs.* time during cell polarization.

8.6.6 POEM / PLiMTFSI / LiTf blend; [EO]:[LiMTFSI]:[LiTf] = 10:0.60:0.40 (molar ratio)

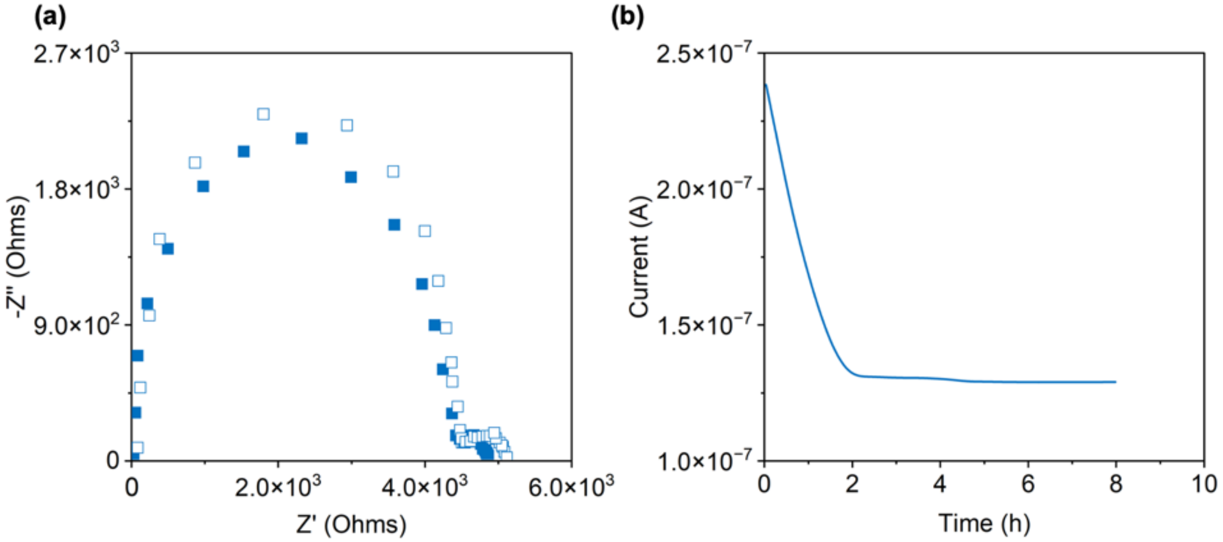

Figure S169. (a) Z'' as a function of Z' for lithium–lithium symmetric cell A before (filled squares) and after polarization (hollow squares). (b) Current *vs.* time during cell polarization.

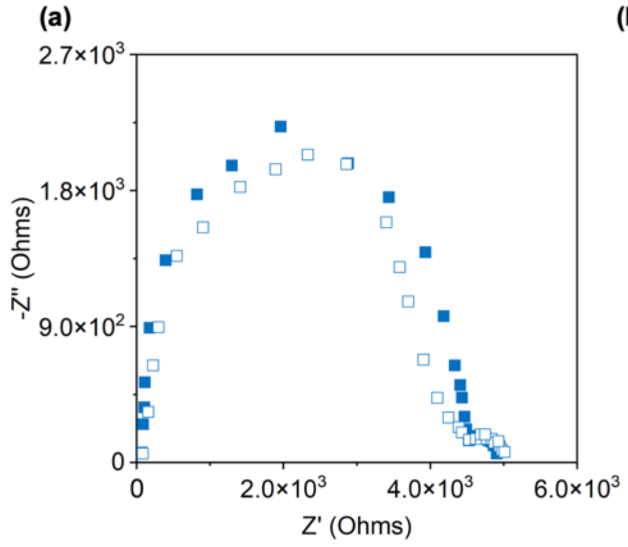

Figure S170. (a) Z'' as a function of Z' for lithium–lithium symmetric cell B before (filled squares) and after polarization (hollow squares). (b) Current *vs.* time during cell polarization.

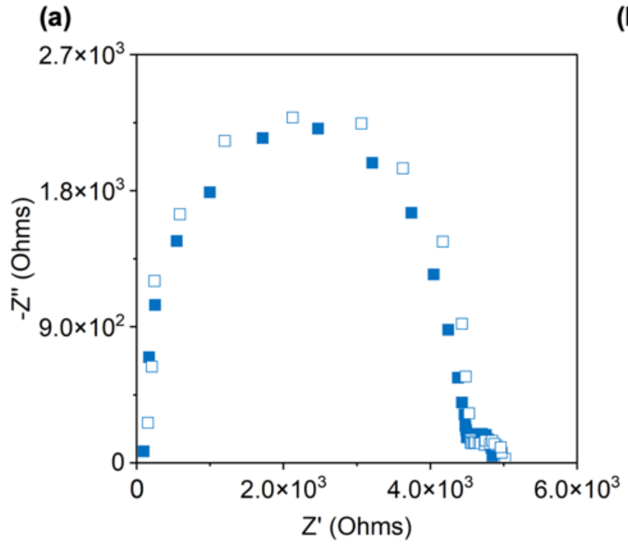

Figure S171. (a) Z'' as a function of Z' for lithium–lithium symmetric cell C before (filled squares) and after polarization (hollow squares). (b) Current *vs.* time during cell polarization.

8.6.7 POEM / PLiMTFSI / LiTf blend; [EO]:[LiMTFSI]:[LiTf] = 10:0.80:0.20 (molar ratio)

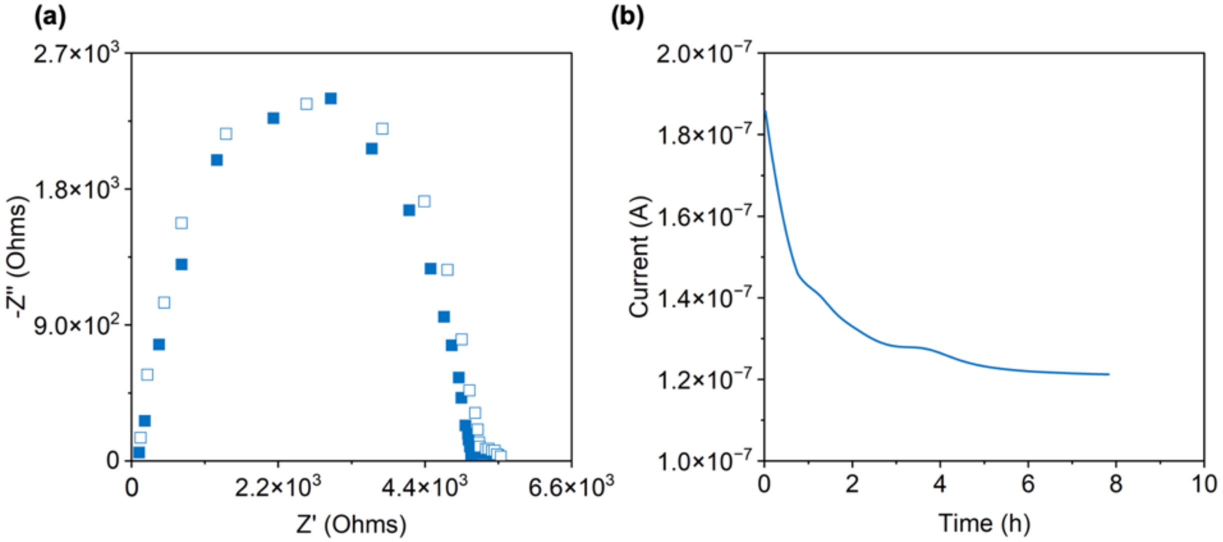

Figure S172. (a) Z'' as a function of Z' for lithium–lithium symmetric cell A before (filled squares) and after polarization (hollow squares). (b) Current *vs.* time during cell polarization.

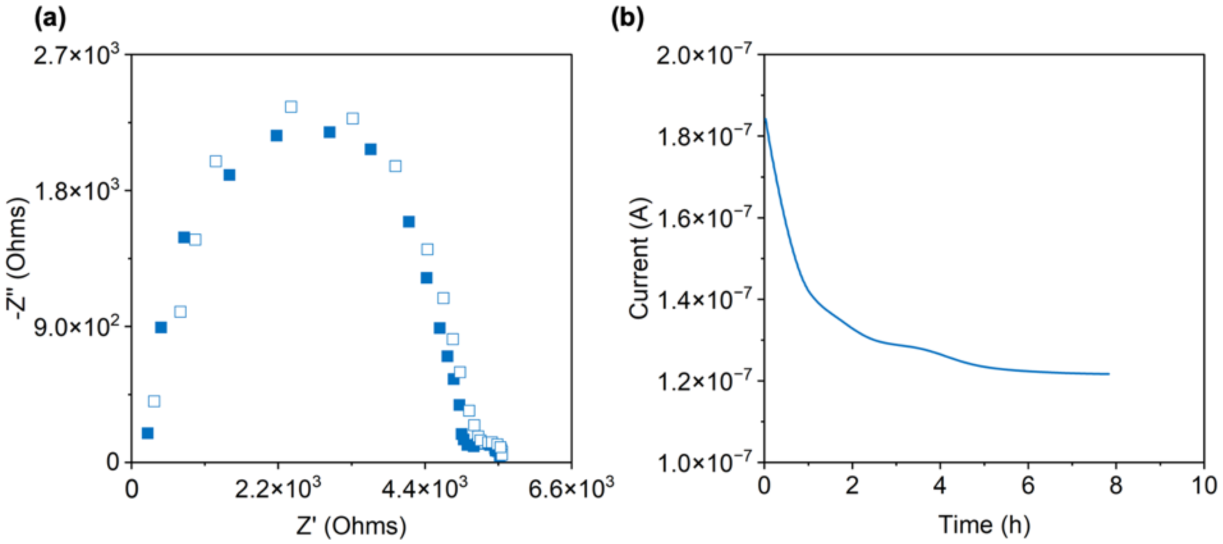

Figure S173. (a) Z'' as a function of Z' for lithium–lithium symmetric cell B before (filled squares) and after polarization (hollow squares). (b) Current *vs.* time during cell polarization.

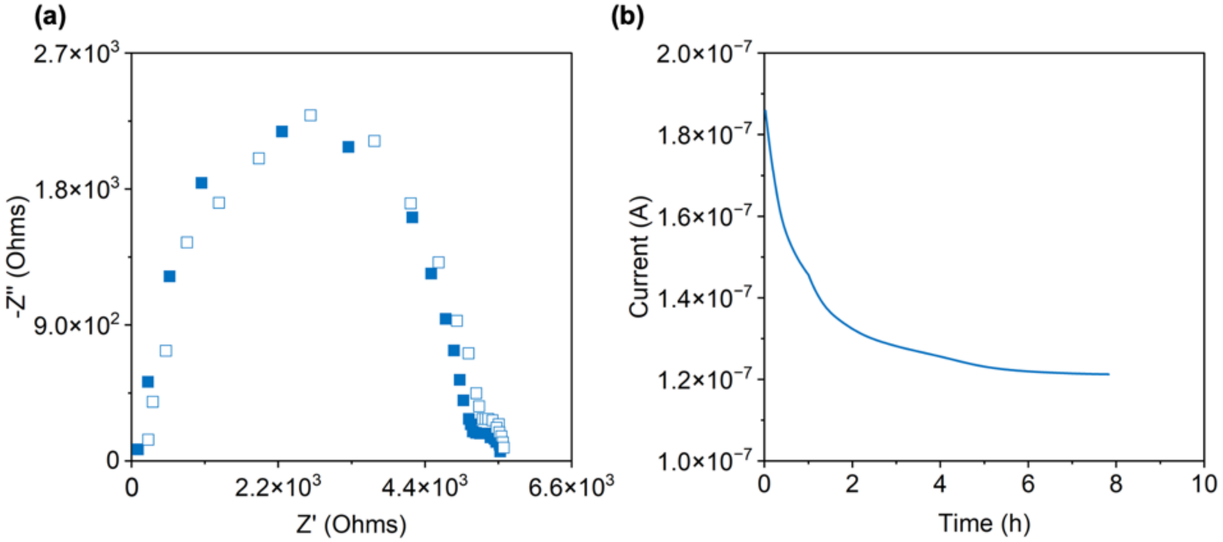

Figure S174. (a) Z'' as a function of Z' for lithium–lithium symmetric cell C before (filled squares) and after polarization (hollow squares). (b) Current *vs.* time during cell polarization.

Summary of the parameters for the Bruce-Vincent method for POEM / PLiMTFSI / $LiClO_4$ blends.

Table S25 (a). Impedance and current values extracted from AC impedance spectroscopy and potentiostatic polarization measurements of three lithium–lithium symmetric cells for POEM / PLiMTFSI / $LiClO_4$ blend; [EO]:[LiMTFSI]:[$LiClO_4$] = 10:0.05:0.95 (molar ratio) at 60 °C.

	$^bR_1$ (Ohms)	$^bR_2$ (Ohms)	$^cR_1^*$ (Ohms)	$^cR_2^*$ (Ohms)	$^dR_0$ (Ohms)	$^eR_{SS}$ (Ohms)	$^fI_0$ (A)	I_{SS} (A)	t_{Li+}
^{a}Cell A	1.58×10^6	1.78×10^6	1.58×10^6	1.80×10^6	2.08×10^5	2.18×10^5	5.60×10^{-9}	1.07×10^{-9}	0.19
^{a}Cell B	1.58×10^6	1.79×10^6	1.60×10^6	1.81×10^6	2.06×10^5	2.08×10^5	5.59×10^{-9}	1.12×10^{-9}	0.20
^{a}Cell C	1.58×10^6	1.79×10^6	1.59×10^6	1.84×10^6	2.15×10^5	2.54×10^5	5.57×10^{-9}	1.06×10^{-9}	0.19

^{a}Cells A, B, and C were made with lithium non-blocking electrodes. $^bR_1$ and R_2 are the impedances of the minima at the bounds of the low-frequency semicircle in a Nyquist plot before cell polarization. $^cR_1$* and R_2* are the corresponding impedances after polarization. ^{d}Initial interfacial impedance was defined as $R_0 = R_2 - R_1$. ^{e}Steady-state interfacial impedance was defined as $R_{SS} = R_2^* - R_1^*$. ^{f}Initial current, I_0, was calculated by Ohm's law. This footnote applies to all tables in this section.

Table S25 (b). Impedance and current values extracted from AC impedance spectroscopy and potentiostatic polarization measurements of three lithium–lithium symmetric cells for POEM / PLiMTFSI / $LiClO_4$ blend; [EO]:[LiMTFSI]:[$LiClO_4$] = 10:0.05:0.95 (molar ratio) at 100 °C.

	$^bR_1$ (Ohms)	$^bR_2$ (Ohms)	$^cR_1^*$ (Ohms)	$^cR_2^*$ (Ohms)	$^dR_0$ (Ohms)	$^eR_{SS}$ (Ohms)	$^fI_0$ (A)	I_{SS} (A)	t_{Li+}
^{a}Cell A	1.82×10^4	1.95×10^4	1.91×10^4	2.03×10^4	1310	1280	5.12×10^{-7}	1.02×10^{-7}	0.20
^{a}Cell B	1.85×10^4	1.97×10^4	1.88×10^4	2.03×10^4	1190	1190	5.07×10^{-7}	1.12×10^{-7}	0.22
^{a}Cell C	1.86×10^4	1.99×10^4	1.91×10^4	2.03×10^4	1210	1210	5.04×10^{-7}	1.11×10^{-7}	0.22

Table S26 (a). Impedance and current values extracted from AC impedance spectroscopy and potentiostatic polarization measurements of three lithium–lithium symmetric cells for POEM / PLiMTFSI / $LiClO_4$ blend; [EO]:[LiMTFSI]:[$LiClO_4$] = 10:0.15:0.85 (molar ratio) at 60 °C.

	$^{b}R_1$ (Ohms)	$^{b}R_2$ (Ohms)	$^{c}R_1^*$ (Ohms)	$^{c}R_2^*$ (Ohms)	$^{d}R_0$ (Ohms)	$^{e}R_{SS}$ (Ohms)	$^{f}I_0$ (A)	I_{SS} (A)	t_{Li+}
^{a}Cell A	5.99×10^{5}	6.69×10^{5}	6.09×10^{5}	6.82×10^{5}	6.96×10^{4}	7.34×10^{4}	1.50×10^{-8}	4.19×10^{-9}	0.28
^{a}Cell B	6.00×10^{5}	6.69×10^{5}	6.10×10^{5}	6.83×10^{5}	6.96×10^{4}	7.34×10^{4}	1.50×10^{-8}	4.18×10^{-9}	0.28
^{a}Cell C	5.97×10^{5}	6.68×10^{5}	6.10×10^{5}	6.86×10^{5}	7.05×10^{4}	7.62×10^{4}	1.50×10^{-8}	4.20×10^{-9}	0.28

Table S26 (b). Impedance and current values extracted from AC impedance spectroscopy and potentiostatic polarization measurements of three lithium–lithium symmetric cells for POEM / PLiMTFSI / $LiClO_4$ blend; [EO]:[LiMTFSI]:[$LiClO_4$] = 10:0.15:0.85 (molar ratio) at 100 °C.

	$^{b}R_1$ (Ohms)	$^{b}R_2$ (Ohms)	$^{c}R_1^*$ (Ohms)	$^{c}R_2^*$ (Ohms)	$^{d}R_0$ (Ohms)	$^{e}R_{SS}$ (Ohms)	$^{f}I_0$ (A)	I_{SS} (A)	t_{Li+}
^{a}Cell A	1.32×10^{4}	1.48×10^{4}	1.36×10^{4}	1.52×10^{4}	1610	1650	6.76×10^{-7}	2.10×10^{-7}	0.31
^{a}Cell B	1.33×10^{4}	1.50×10^{4}	1.36×10^{4}	1.53×10^{4}	1690	1710	6.67×10^{-7}	2.07×10^{-7}	0.31
^{a}Cell C	1.32×10^{4}	1.49×10^{4}	1.36×10^{4}	1.53×10^{4}	1690	1700	6.71×10^{-7}	2.08×10^{-7}	0.31

Table S27 (a). Impedance and current values extracted from AC impedance spectroscopy and potentiostatic polarization measurements of three lithium–lithium symmetric cells for POEM / PLiMTFSI / $LiClO_4$ blend; [EO]:[LiMTFSI]:[$LiClO_4$] = 10:0.25:0.75 (molar ratio) at 60 °C.

	$^{b}R_1$ (Ohms)	$^{b}R_2$ (Ohms)	$^{c}R_1^*$ (Ohms)	$^{c}R_2^*$ (Ohms)	$^{d}R_0$ (Ohms)	$^{e}R_{SS}$ (Ohms)	$^{f}I_0$ (A)	I_{SS} (A)	t_{Li+}
^{a}Cell A	1.45×10^{5}	1.58×10^{5}	1.47×10^{5}	1.61×10^{5}	1.28×10^{4}	1.40×10^{4}	6.34×10^{-8}	2.47×10^{-8}	0.39
^{a}Cell B	1.46×10^{5}	1.58×10^{5}	1.47×10^{5}	1.60×10^{5}	1.21×10^{4}	1.30×10^{4}	6.33×10^{-8}	2.47×10^{-8}	0.39
^{a}Cell C	1.46×10^{5}	1.59×10^{5}	1.47×10^{5}	1.61×10^{5}	1.30×10^{4}	1.40×10^{4}	6.30×10^{-8}	2.52×10^{-8}	0.40

Table S27 (b). Impedance and current values extracted from AC impedance spectroscopy and potentiostatic polarization measurements of three lithium–lithium symmetric cells for POEM / PLiMTFSI / $LiClO_4$ blend; [EO]:[LiMTFSI]:[$LiClO_4$] = 10:0.25:0.75 (molar ratio) at 100 °C.

	$^{b}R_1$ (Ohms)	$^{b}R_2$ (Ohms)	$^{c}R_1^*$ (Ohms)	$^{c}R_2^*$ (Ohms)	$^{d}R_0$ (Ohms)	$^{e}R_{SS}$ (Ohms)	$^{f}I_0$ (A)	I_{SS} (A)	t_{Li+}
^{a}Cell A	5000	5450	5110	5600	450	490	1.84×10^{-6}	7.53×10^{-7}	0.41
^{a}Cell B	5060	5540	5090	5580	480	490	1.81×10^{-6}	7.22×10^{-7}	0.40
^{a}Cell C	5020	5470	5100	5590	450	490	1.83×10^{-6}	7.31×10^{-7}	0.40

Table S28 (a). Impedance and current values extracted from AC impedance spectroscopy and potentiostatic polarization measurements of three lithium–lithium symmetric cells for POEM / PLiMTFSI / $LiClO_4$ blend; [EO]:[LiMTFSI]:[$LiClO_4$] = 10:0.40:0.60 (molar ratio) at 60 °C.

	$^{b}R_1$ (Ohms)	$^{b}R_2$ (Ohms)	$^{c}R_1^*$ (Ohms)	$^{c}R_2^*$ (Ohms)	$^{d}R_0$ (Ohms)	$^{e}R_{SS}$ (Ohms)	$^{f}I_0$ (A)	I_{SS} (A)	t_{Li+}
^{a}Cell A	1.98×10^{5}	2.16×10^{5}	1.97×10^{5}	2.15×10^{5}	1.79×10^{4}	1.85×10^{4}	4.64×10^{-8}	1.86×10^{-8}	0.40
^{a}Cell B	1.97×10^{5}	2.15×10^{5}	1.97×10^{5}	2.16×10^{5}	1.82×10^{4}	1.87×10^{4}	4.65×10^{-8}	1.86×10^{-8}	0.40
^{a}Cell C	1.97×10^{5}	2.16×10^{5}	1.96×10^{5}	2.14×10^{5}	1.82×10^{4}	1.85×10^{4}	4.64×10^{-8}	1.86×10^{-8}	0.40

Table S28 (b). Impedance and current values extracted from AC impedance spectroscopy and potentiostatic polarization measurements of three lithium–lithium symmetric cells for POEM / PLiMTFSI / $LiClO_4$ blend; [EO]:[LiMTFSI]:[$LiClO_4$] = 10:0.40:0.60 (molar ratio) at 100 °C.

	$^{b}R_1$ (Ohms)	$^{b}R_2$ (Ohms)	$^{c}R_1^*$ (Ohms)	$^{c}R_2^*$ (Ohms)	$^{d}R_0$ (Ohms)	$^{e}R_{SS}$ (Ohms)	$^{f}I_0$ (A)	I_{SS} (A)	t_{Li+}
^{a}Cell A	7250	7610	7310	7770	360	460	1.31×10^{-6}	5.39×10^{-7}	0.41
^{a}Cell B	7290	7650	7400	7810	360	410	1.31×10^{-6}	5.36×10^{-7}	0.41
^{a}Cell C	7250	7670	7380	7840	420	460	1.30×10^{-6}	5.48×10^{-7}	0.42

Table S29 (a). Impedance and current values extracted from AC impedance spectroscopy and potentiostatic polarization measurements of three lithium–lithium symmetric cells for POEM / PLiMTFSI / $LiClO_4$ blend; [EO]:[LiMTFSI]:[$LiClO_4$] = 10:0.50:0.50 (molar ratio) at 60 °C.

	$^{b}R_1$ (Ohms)	$^{b}R_2$ (Ohms)	$^{c}R_1^*$ (Ohms)	$^{c}R_2^*$ (Ohms)	$^{d}R_0$ (Ohms)	$^{e}R_{SS}$ (Ohms)	$^{f}I_0$ (A)	I_{SS} (A)	t_{Li+}
^{a}Cell A	9.84×10^{4}	1.06×10^{5}	9.99×10^{4}	1.08×10^{5}	7770	8310	9.41×10^{-8}	4.33×10^{-8}	0.46
^{a}Cell B	9.89×10^{4}	1.07×10^{5}	9.97×10^{4}	1.09×10^{5}	7780	8770	9.37×10^{-8}	4.41×10^{-8}	0.47
^{a}Cell C	9.91×10^{4}	1.07×10^{5}	9.92×10^{4}	1.08×10^{5}	7820	8760	9.36×10^{-8}	4.31×10^{-8}	0.46

Table S29 (b). Impedance and current values extracted from AC impedance spectroscopy and potentiostatic polarization measurements of three lithium–lithium symmetric cells for POEM / PLiMTFSI / $LiClO_4$ blend; [EO]:[LiMTFSI]:[$LiClO_4$] = 10:0.50:0.50 (molar ratio) at 100 °C.

	$^{b}R_1$ (Ohms)	$^{b}R_2$ (Ohms)	$^{c}R_1^*$ (Ohms)	$^{c}R_2^*$ (Ohms)	$^{d}R_0$ (Ohms)	$^{e}R_{SS}$ (Ohms)	$^{f}I_0$ (A)	I_{SS} (A)	t_{Li+}
^{a}Cell A	4410	4760	4510	4850	350	340	2.10×10^{-6}	1.01×10^{-6}	0.48
^{a}Cell B	4480	4800	4540	4920	320	380	2.08×10^{-6}	1.00×10^{-6}	0.48
^{a}Cell C	4420	4790	4520	4960	370	440	2.09×10^{-6}	1.00×10^{-6}	0.48

Table S30 (a). Impedance and current values extracted from AC impedance spectroscopy and potentiostatic polarization measurements of three lithium–lithium symmetric cells for POEM / PLiMTFSI / $LiClO_4$ blend; [EO]:[LiMTFSI]:[$LiClO_4$] = 10:0.60:0.40 (molar ratio) at 60 °C.

	$^{b}R_1$ (Ohms)	$^{b}R_2$ (Ohms)	$^{c}R_1^*$ (Ohms)	$^{c}R_2^*$ (Ohms)	$^{d}R_0$ (Ohms)	$^{e}R_{SS}$ (Ohms)	$^{f}I_0$ (A)	I_{SS} (A)	t_{Li+}
^{a}Cell A	3.59×10^{4}	3.91×10^{4}	3.67×10^{4}	3.98×10^{4}	3210	3110	2.56×10^{-7}	1.30×10^{-7}	0.51
^{a}Cell B	3.60×10^{4}	3.91×10^{4}	3.66×10^{4}	3.99×10^{4}	3040	3320	2.56×10^{-7}	1.31×10^{-7}	0.51
^{a}Cell C	3.60×10^{4}	3.90×10^{4}	3.63×10^{4}	3.95×10^{4}	3020	3160	2.56×10^{-7}	1.31×10^{-7}	0.51

Table S30 (b). Impedance and current values extracted from AC impedance spectroscopy and potentiostatic polarization measurements of three lithium–lithium symmetric cells for POEM / PLiMTFSI / $LiClO_4$ blend; [EO]:[LiMTFSI]:[$LiClO_4$] = 10:0.60:0.40 (molar ratio) at 100 °C.

	$^{b}R_1$ (Ohms)	$^{b}R_2$ (Ohms)	$^{c}R_1^*$ (Ohms)	$^{c}R_2^*$ (Ohms)	$^{d}R_0$ (Ohms)	$^{e}R_{SS}$ (Ohms)	$^{f}I_0$ (A)	I_{SS} (A)	t_{Li+}
^{a}Cell A	2330	2490	2350	2540	160	190	4.01×10^{-6}	2.09×10^{-6}	0.52
^{a}Cell B	2330	2520	2340	2530	190	190	3.97×10^{-6}	2.07×10^{-6}	0.52
^{a}Cell C	2320	2480	2340	2540	160	200	4.04×10^{-6}	2.14×10^{-6}	0.53

Table S31 (a). Impedance and current values extracted from AC impedance spectroscopy and potentiostatic polarization measurements of three lithium–lithium symmetric cells for POEM / PLiMTFSI / $LiClO_4$ blend; [EO]:[LiMTFSI]:[$LiClO_4$] = 10:0.80:0.20 (molar ratio) at 60 °C.

	$^{b}R_1$ (Ohms)	$^{b}R_2$ (Ohms)	$^{c}R_1^*$ (Ohms)	$^{c}R_2^*$ (Ohms)	$^{d}R_0$ (Ohms)	$^{e}R_{SS}$ (Ohms)	$^{f}I_0$ (A)	I_{SS} (A)	t_{Li+}
^{a}Cell A	1.38×10^{5}	1.46×10^{5}	1.40×10^{5}	1.49×10^{5}	7400	8600	6.87×10^{-8}	4.05×10^{-8}	0.59
^{a}Cell B	1.40×10^{5}	1.47×10^{5}	1.40×10^{5}	1.48×10^{5}	7200	7700	6.80×10^{-8}	4.08×10^{-8}	0.60
^{a}Cell C	1.40×10^{5}	1.48×10^{5}	1.41×10^{5}	1.50×10^{5}	7800	8900	6.77×10^{-8}	3.99×10^{-8}	0.59

Table S31 (b). Impedance and current values extracted from AC impedance spectroscopy and potentiostatic polarization measurements of three lithium–lithium symmetric cells for POEM / PLiMTFSI / $LiClO_4$ blend; [EO]:[LiMTFSI]:[$LiClO_4$] = 10:0.80:0.20 (molar ratio) at 100 °C.

	$^{b}R_1$ (Ohms)	$^{b}R_2$ (Ohms)	$^{c}R_1^*$ (Ohms)	$^{c}R_2^*$ (Ohms)	$^{d}R_0$ (Ohms)	$^{e}R_{SS}$ (Ohms)	$^{f}I_0$ (A)	I_{SS} (A)	t_{Li+}
^{a}Cell A	8690	9310	8760	9410	620	650	1.07×10^{-6}	6.34×10^{-7}	0.59
^{a}Cell B	8670	9270	8780	9490	600	710	1.08×10^{-6}	6.37×10^{-7}	0.59
^{a}Cell C	8700	9350	8770	9460	650	690	1.07×10^{-6}	6.31×10^{-7}	0.59

8.7 Measurement conducted at 60 °C for POEM / PLiMTFSI / $LiClO_4$ blends

8.7.1 POEM / PLiMTFSI / $LiClO_4$ blend; [EO]:[LiMTFSI]:[$LiClO_4$] = 10:0.05:0.95 (molar ratio)

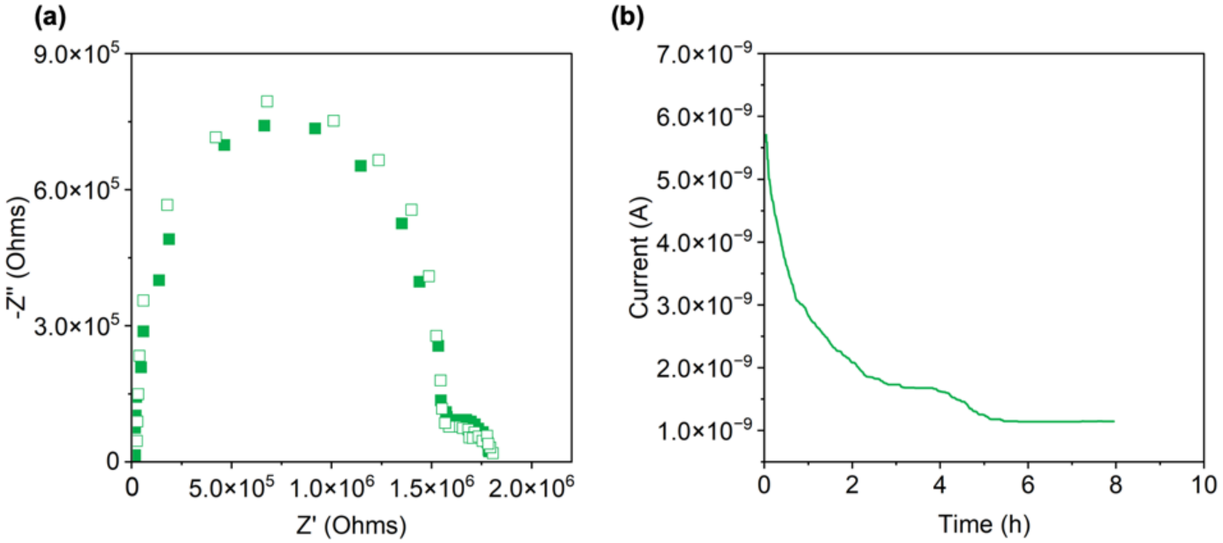

Figure S175. (a) Z″ as a function of Z′ for lithium–lithium symmetric cell A before (filled squares) and after polarization (hollow squares). (b) Current *vs.* time during cell polarization.

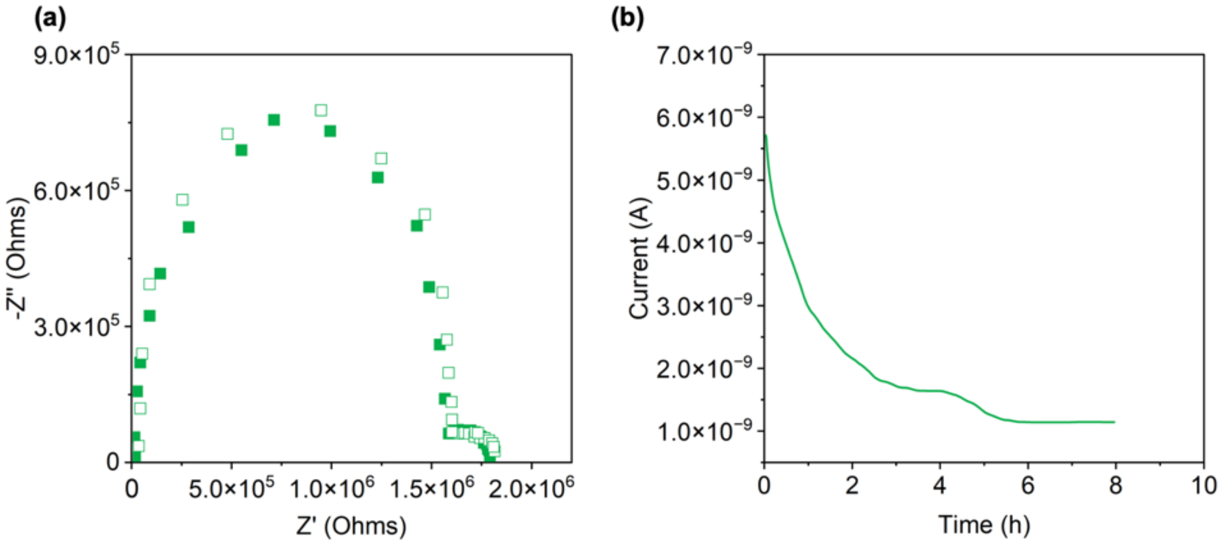

Figure S176. (a) Z'' as a function of Z' for lithium–lithium symmetric cell B before (filled squares) and after polarization (hollow squares). (b) Current *vs.* time during cell polarization.

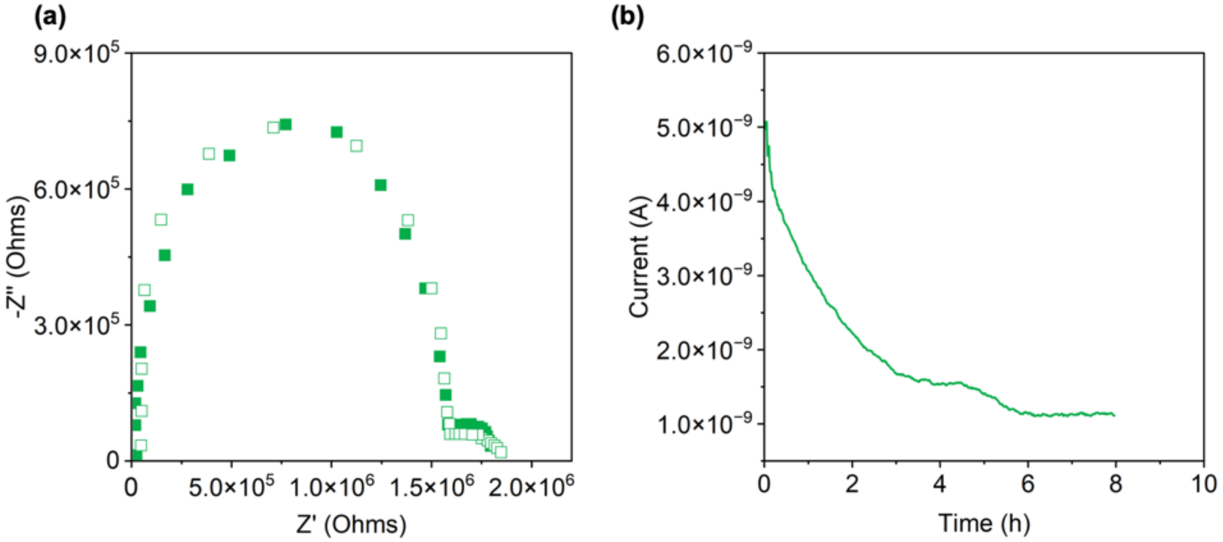

Figure S177. (a) Z'' as a function of Z' for lithium–lithium symmetric cell C before (filled squares) and after polarization (hollow squares). (b) Current *vs.* time during cell polarization.

8.7.2 POEM / PLiMTFSI / $LiClO_4$ blend; [EO]:[LiMTFSI]:[$LiClO_4$] = 10:0.15:0.85 (molar ratio)

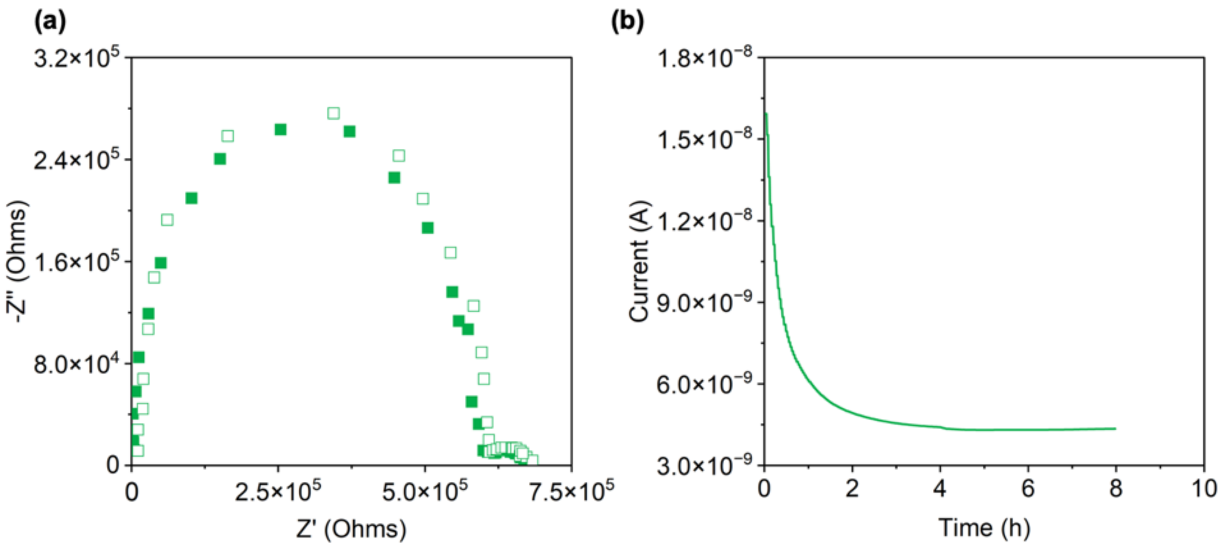

Figure S178. (a) Z'' as a function of Z' for lithium–lithium symmetric cell A before (filled squares) and after polarization (hollow squares). (b) Current *vs.* time during cell polarization.

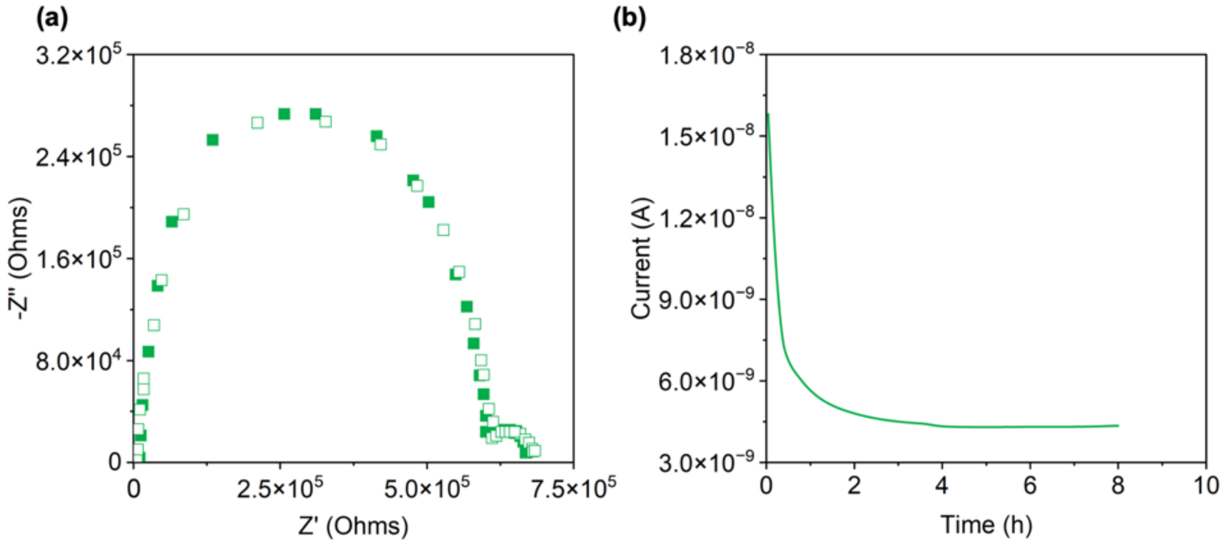

Figure S179. (a) Z'' as a function of Z' for lithium–lithium symmetric cell B before (filled squares) and after polarization (hollow squares). (b) Current *vs.* time during cell polarization.

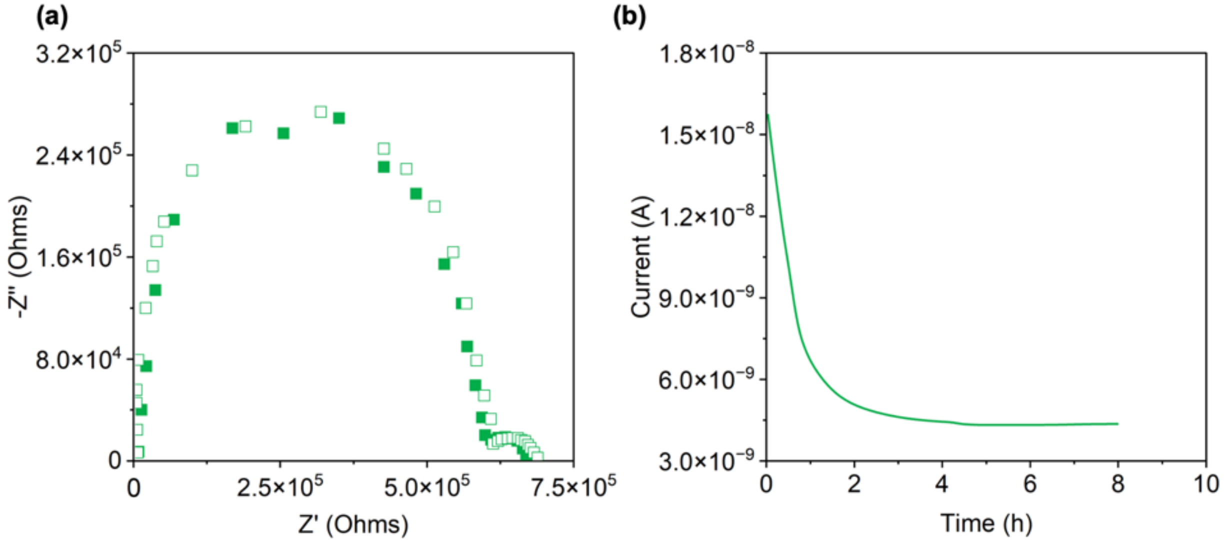

Figure S180. (a) Z'' as a function of Z' for lithium–lithium symmetric cell C before (filled squares) and after polarization (hollow squares). (b) Current *vs.* time during cell polarization.

8.7.3 POEM / PLiMTFSI / $LiClO_4$ blend; [EO]:[LiMTFSI]:[$LiClO_4$] = 10:0.25:0.75 (molar ratio)

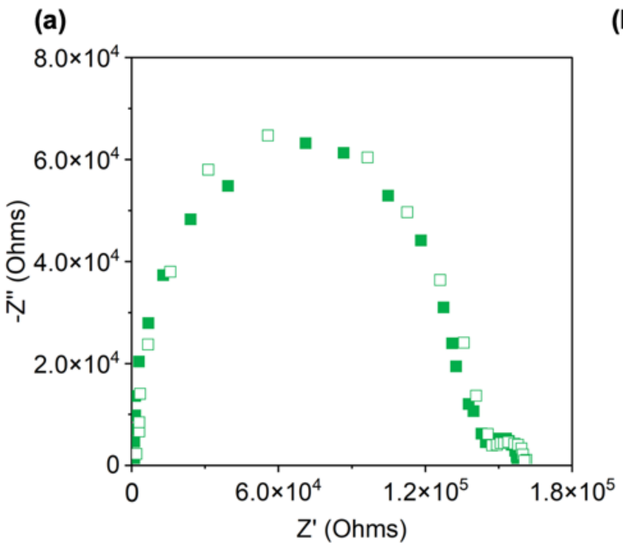

Figure S181. (a) Z'' as a function of Z' for lithium–lithium symmetric cell A before (filled squares) and after polarization (hollow squares). (b) Current *vs.* time during cell polarization.

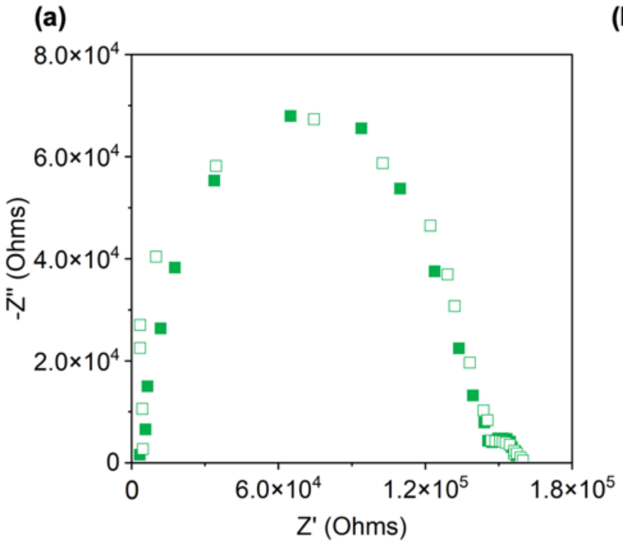

Figure S182. (a) Z'' as a function of Z' for lithium–lithium symmetric cell B before (filled squares) and after polarization (hollow squares). (b) Current *vs.* time during cell polarization.

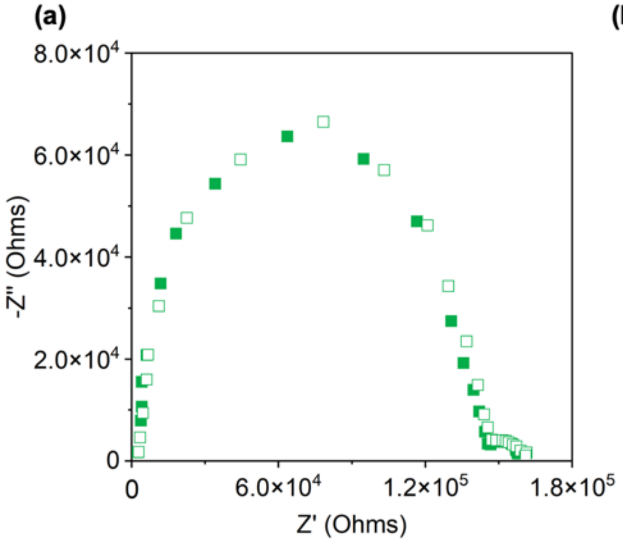

Figure S183. (a) Z'' as a function of Z' for lithium–lithium symmetric cell C before (filled squares) and after polarization (hollow squares). (b) Current *vs.* time during cell polarization.

8.7.4 POEM / PLiMTFSI / $LiClO_4$ blend; [EO]:[LiMTFSI]:[$LiClO_4$] = 10:0.40:0.60 (molar ratio)

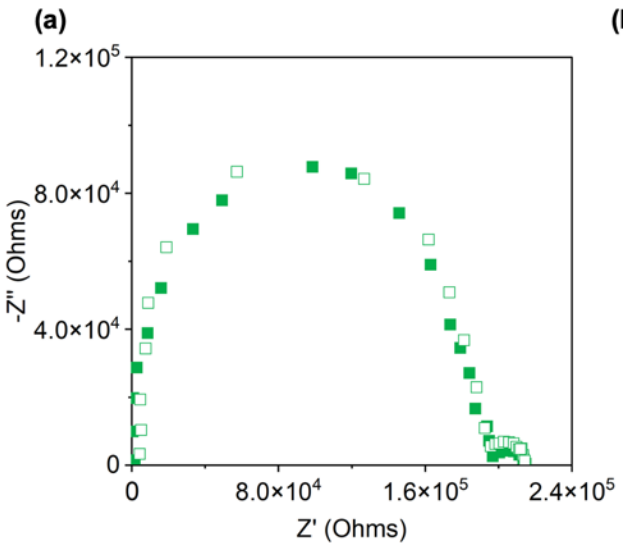

Figure S184. (a) Z'' as a function of Z' for lithium–lithium symmetric cell A before (filled squares) and after polarization (hollow squares). (b) Current *vs.* time during cell polarization.

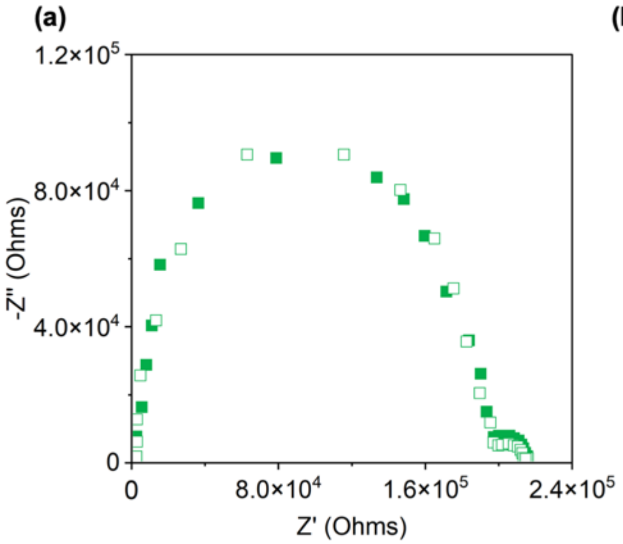

Figure S185. (a) Z'' as a function of Z' for lithium–lithium symmetric cell B before (filled squares) and after polarization (hollow squares). (b) Current *vs.* time during cell polarization.

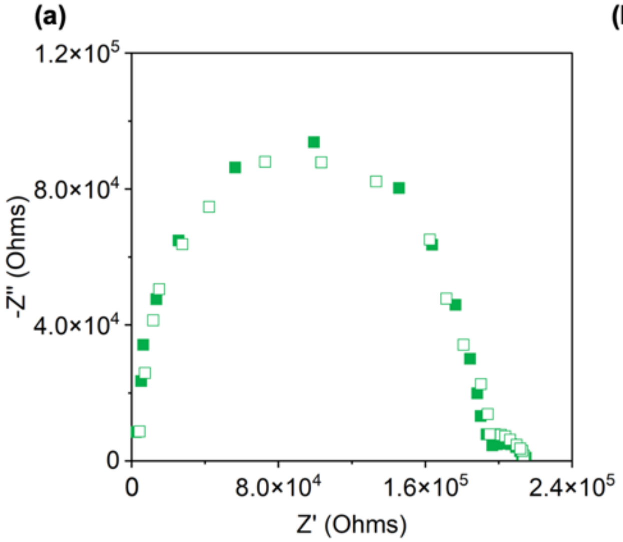

Figure S186. (a) Z'' as a function of Z' for lithium–lithium symmetric cell C before (filled squares) and after polarization (hollow squares). (b) Current *vs.* time during cell polarization.

8.7.5 POEM / PLiMTFSI / $LiClO_4$ blend; [EO]:[LiMTFSI]:[$LiClO_4$] = 10:0.50:0.50 (molar ratio)

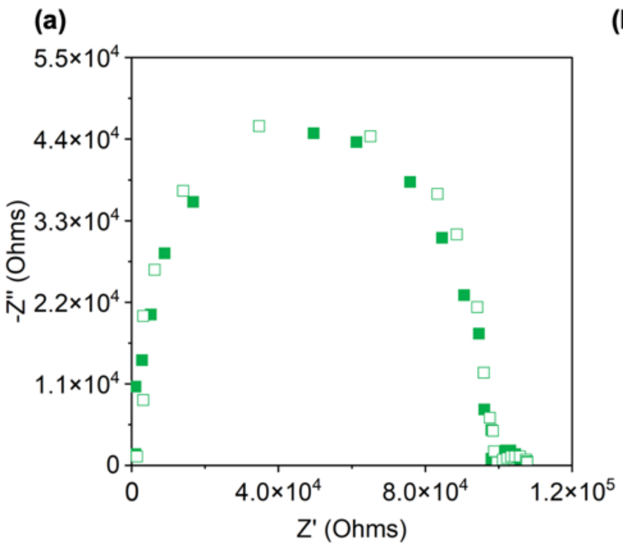

Figure S187. (a) Z'' as a function of Z' for lithium–lithium symmetric cell A before (filled squares) and after polarization (hollow squares). (b) Current *vs.* time during cell polarization.

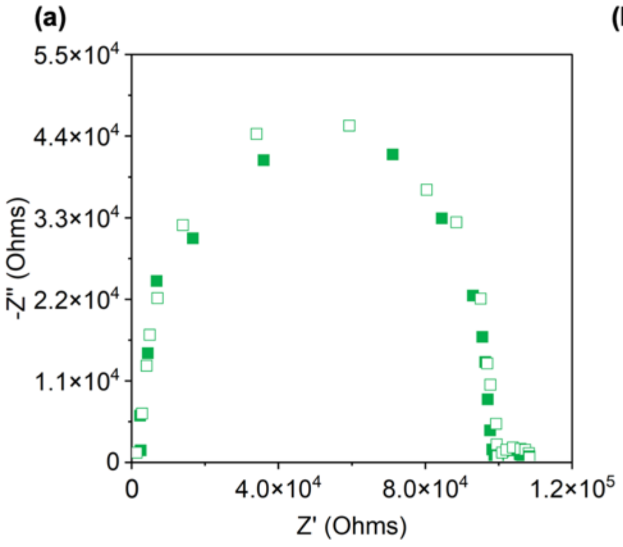

Figure S188. (a) Z'' as a function of Z' for lithium–lithium symmetric cell B before (filled squares) and after polarization (hollow squares). (b) Current *vs.* time during cell polarization.

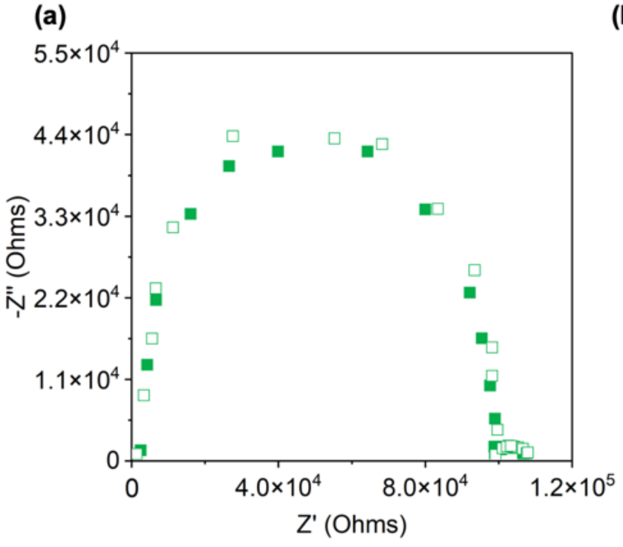

Figure S189. (a) Z'' as a function of Z' for lithium–lithium symmetric cell C before (filled squares) and after polarization (hollow squares). (b) Current *vs.* time during cell polarization.

8.7.6 POEM / PLiMTFSI / $LiClO_4$ blend; [EO]:[LiMTFSI]:[$LiClO_4$] = 10:0.60:0.40 (molar ratio)

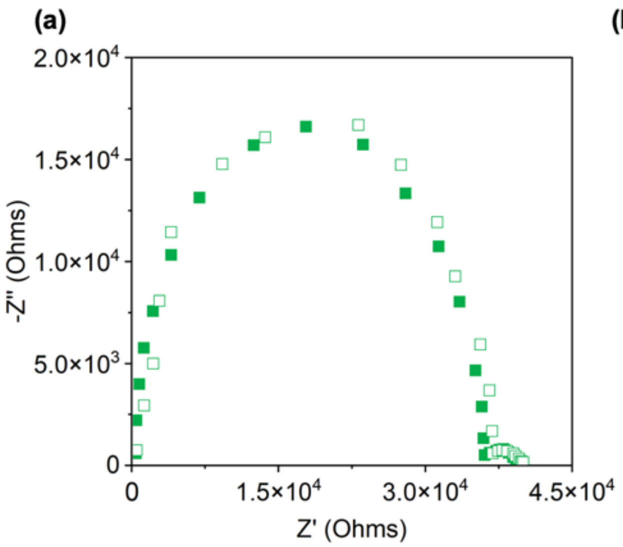

Figure S190. (a) Z'' as a function of Z' for lithium–lithium symmetric cell A before (filled squares) and after polarization (hollow squares). (b) Current *vs.* time during cell polarization.

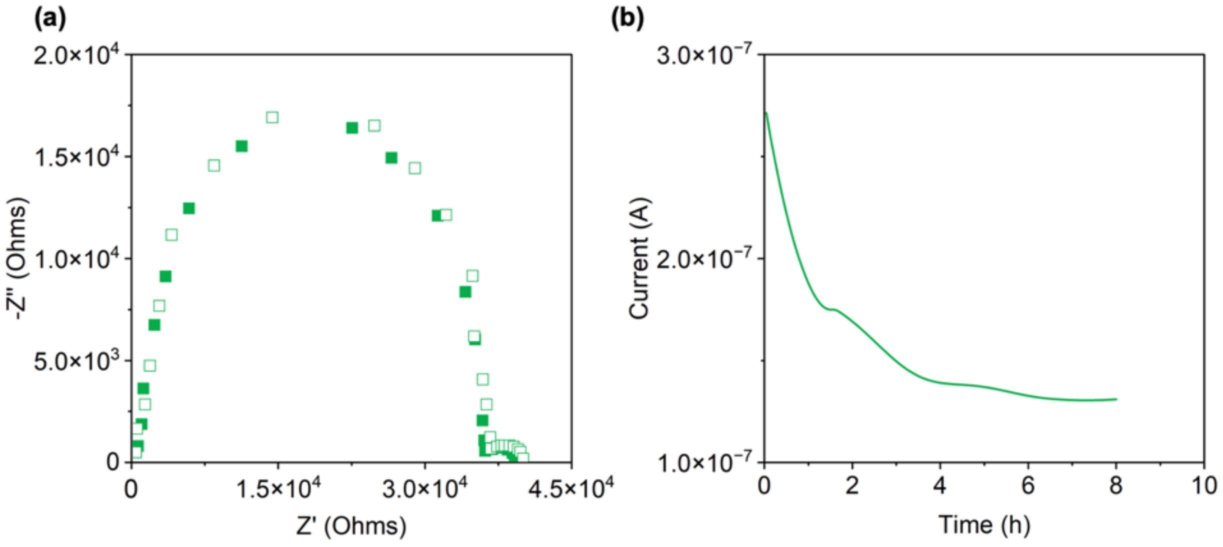

Figure S191. (a) Z'' as a function of Z' for lithium–lithium symmetric cell B before (filled squares) and after polarization (hollow squares). (b) Current *vs.* time during cell polarization.

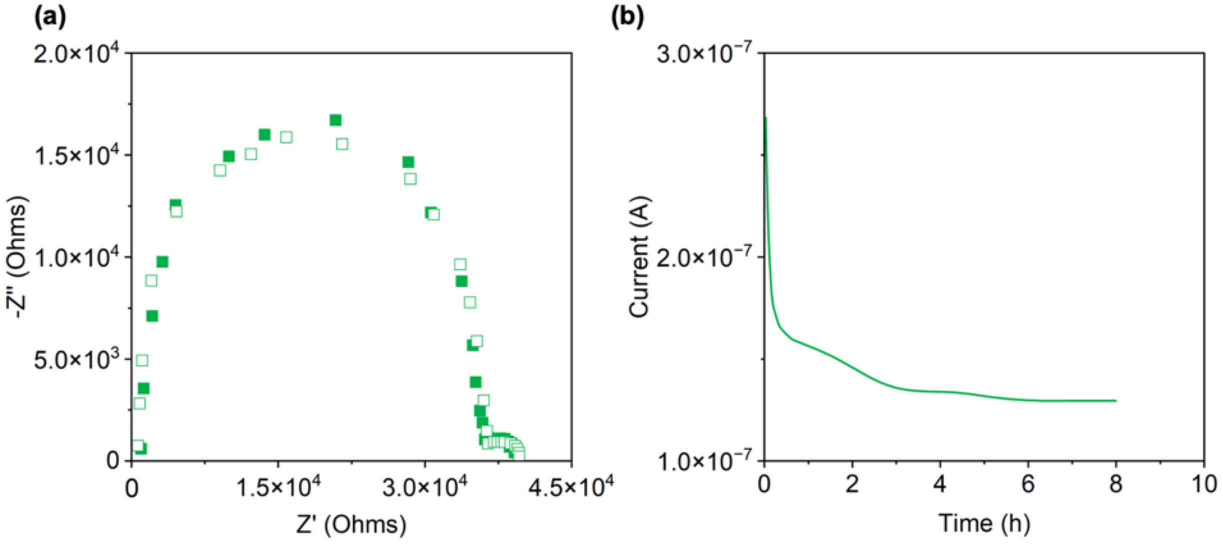

Figure S192. (a) Z'' as a function of Z' for lithium–lithium symmetric cell C before (filled squares) and after polarization (hollow squares). (b) Current *vs.* time during cell polarization.

8.7.7 POEM / PLiMTFSI / $LiClO_4$ blend; [EO]:[LiMTFSI]:[$LiClO_4$] = 10:0.80:0.20 (molar ratio)

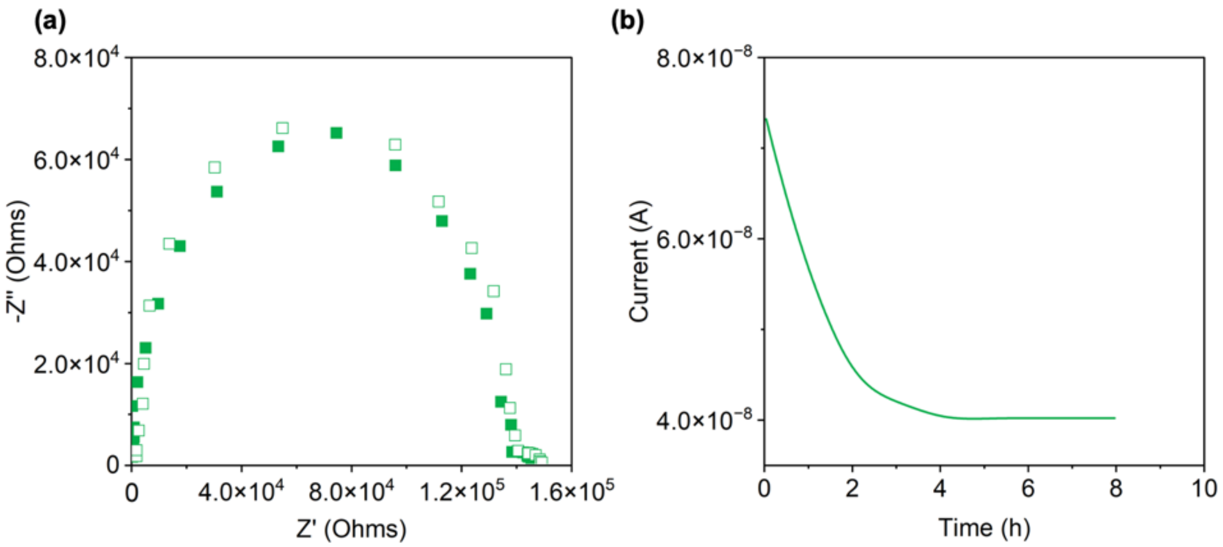

Figure S193. (a) Z'' as a function of Z' for lithium–lithium symmetric cell A before (filled squares) and after polarization (hollow squares). (b) Current *vs.* time during cell polarization.

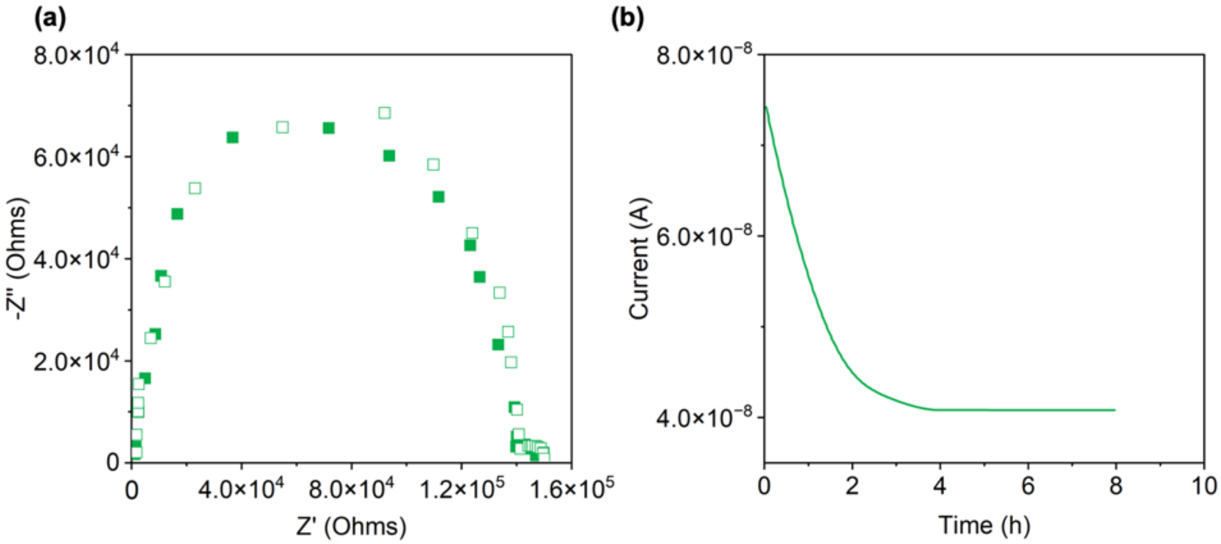

Figure S194. (a) Z'' as a function of Z' for lithium–lithium symmetric cell B before (filled squares) and after polarization (hollow squares). (b) Current *vs.* time during cell polarization.

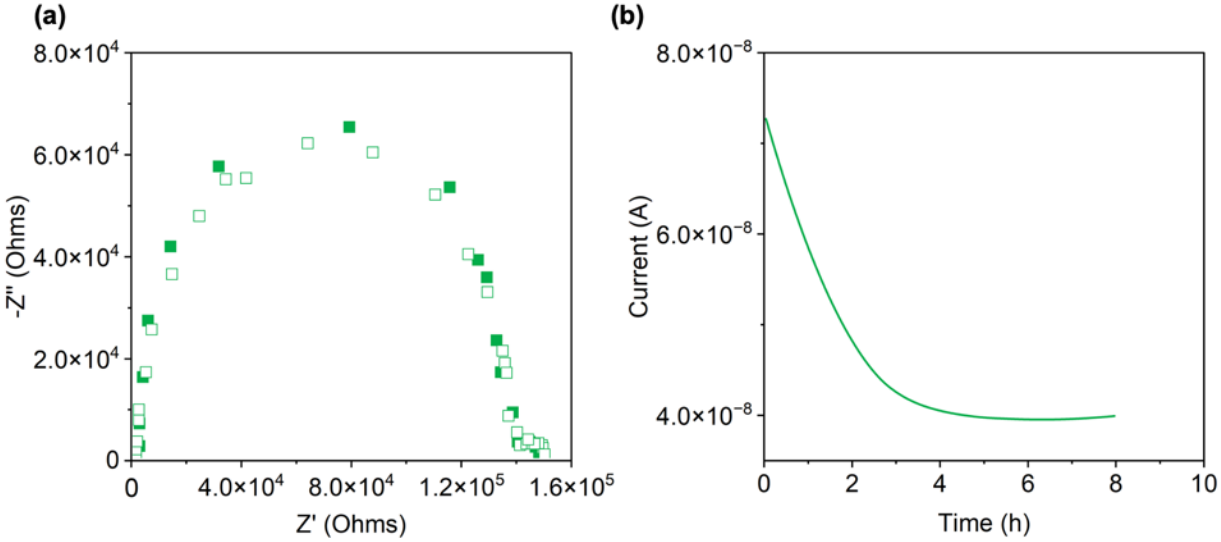

Figure S195. (a) Z'' as a function of Z' for lithium–lithium symmetric cell C before (filled squares) and after polarization (hollow squares). (b) Current *vs.* time during cell polarization.

8.8 Measurement conducted at 100 °C for POEM / PLiMTFSI / $LiClO_4$ blends

8.8.1 POEM / PLiMTFSI / $LiClO_4$ blend; [EO]:[LiMTFSI]:[$LiClO_4$] = 10:0.05:0.95 (molar ratio)

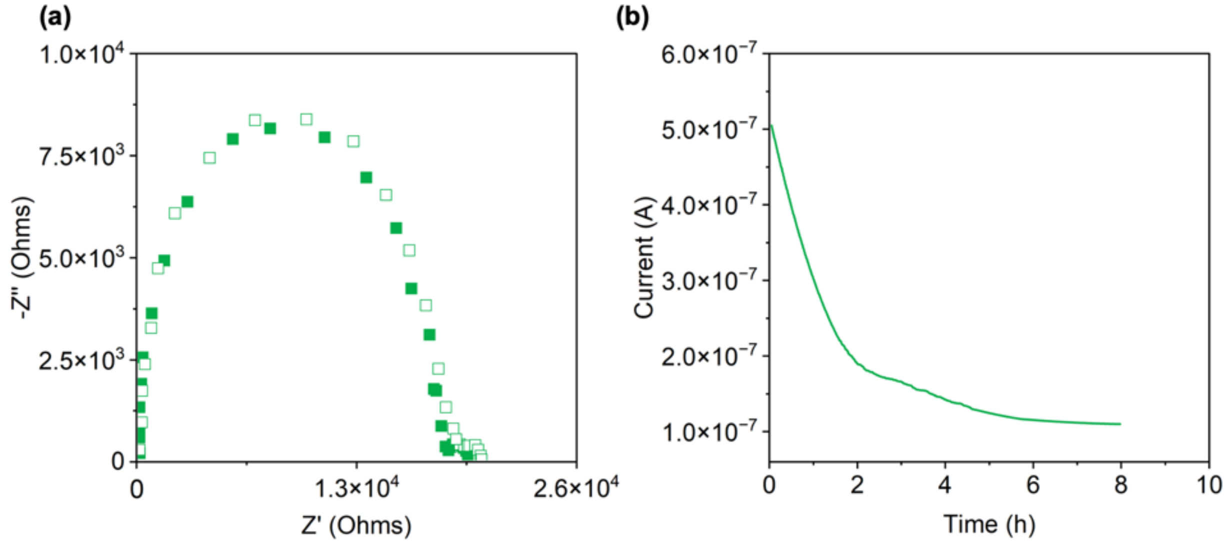

Figure S196. (a) Z″ as a function of Z′ for lithium–lithium symmetric cell A before (filled squares) and after polarization (hollow squares). (b) Current *vs.* time during cell polarization.

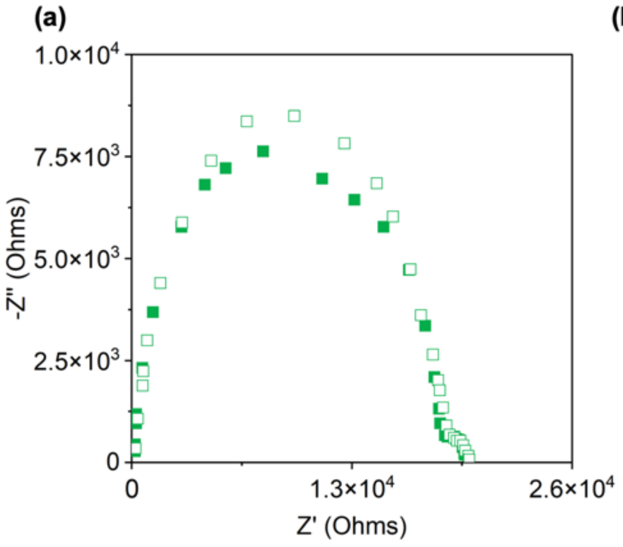

Figure S197. (a) Z'' as a function of Z' for lithium–lithium symmetric cell B before (filled squares) and after polarization (hollow squares). (b) Current *vs.* time during cell polarization.

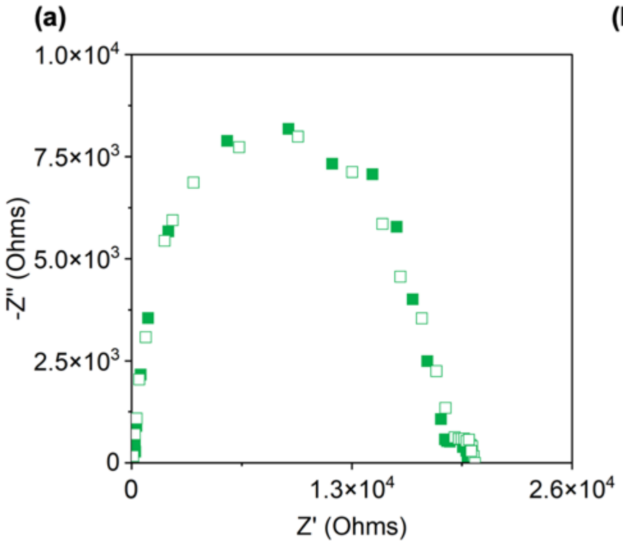

Figure S198. (a) Z'' as a function of Z' for lithium–lithium symmetric cell C before (filled squares) and after polarization (hollow squares). (b) Current *vs.* time during cell polarization.

8.8.2 POEM / PLiMTFSI / $LiClO_4$ blend; [EO]:[LiMTFSI]:[$LiClO_4$] = 10:0.15:0.85 (molar ratio)

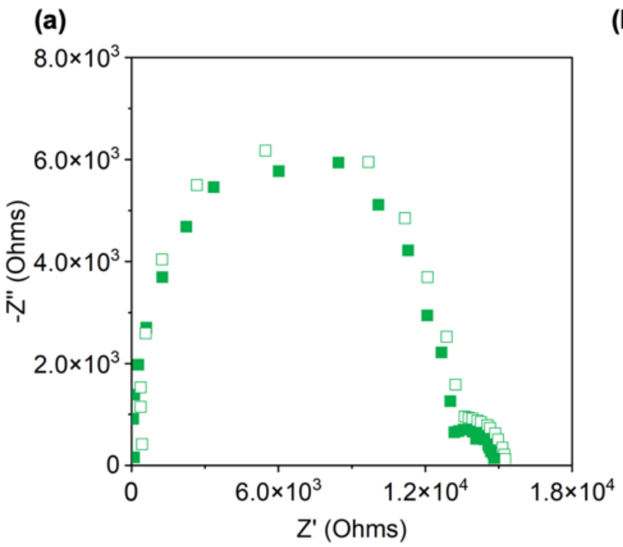

Figure S199. (a) Z'' as a function of Z' for lithium–lithium symmetric cell A before (filled squares) and after polarization (hollow squares). (b) Current *vs.* time during cell polarization.

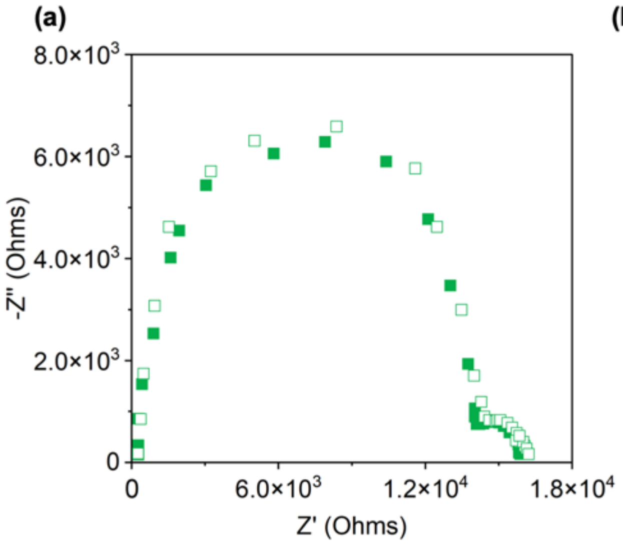

Figure S200. (a) Z'' as a function of Z' for lithium–lithium symmetric cell B before (filled squares) and after polarization (hollow squares). (b) Current *vs.* time during cell polarization.

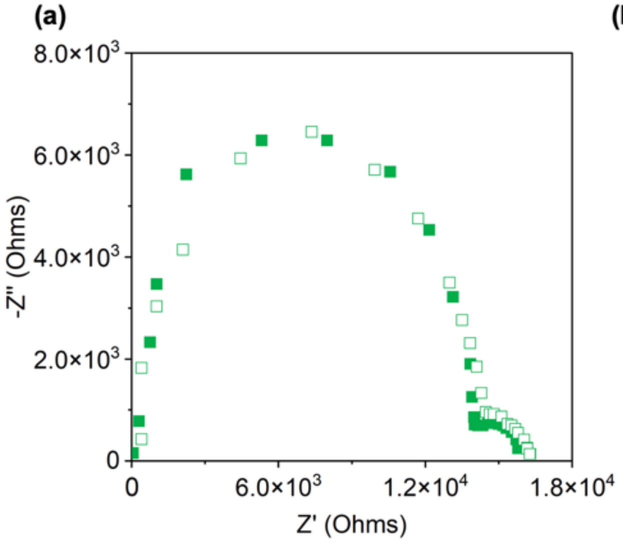

Figure S201. (a) Z'' as a function of Z' for lithium–lithium symmetric cell C before (filled squares) and after polarization (hollow squares). (b) Current *vs.* time during cell polarization.

8.8.3 POEM / PLiMTFSI / $LiClO_4$ blend; [EO]:[LiMTFSI]:[$LiClO_4$] = 10:0.25:0.75 (molar ratio)

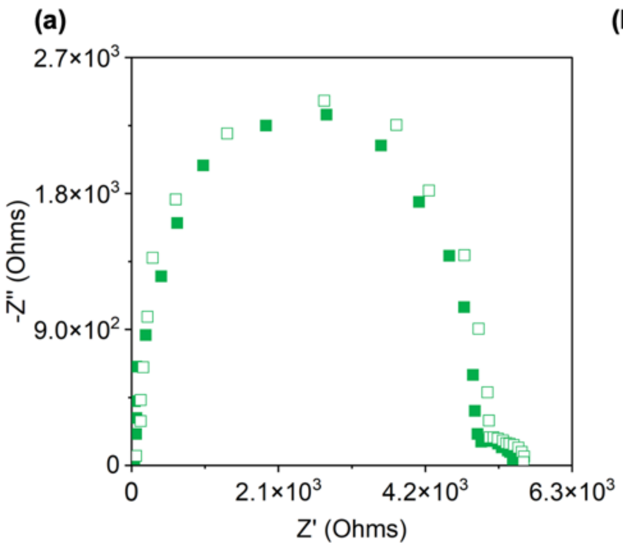

Figure S202. (a) Z'' as a function of Z' for lithium–lithium symmetric cell A before (filled squares) and after polarization (hollow squares). (b) Current *vs.* time during cell polarization.

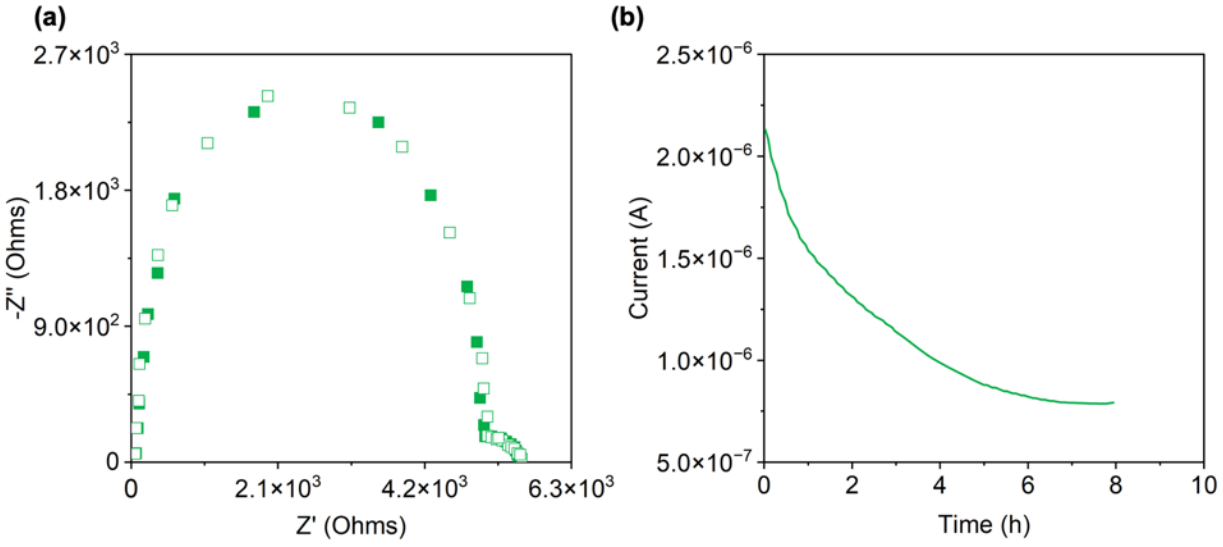

Figure S203. (a) Z'' as a function of Z' for lithium–lithium symmetric cell B before (filled squares) and after polarization (hollow squares). (b) Current *vs.* time during cell polarization.

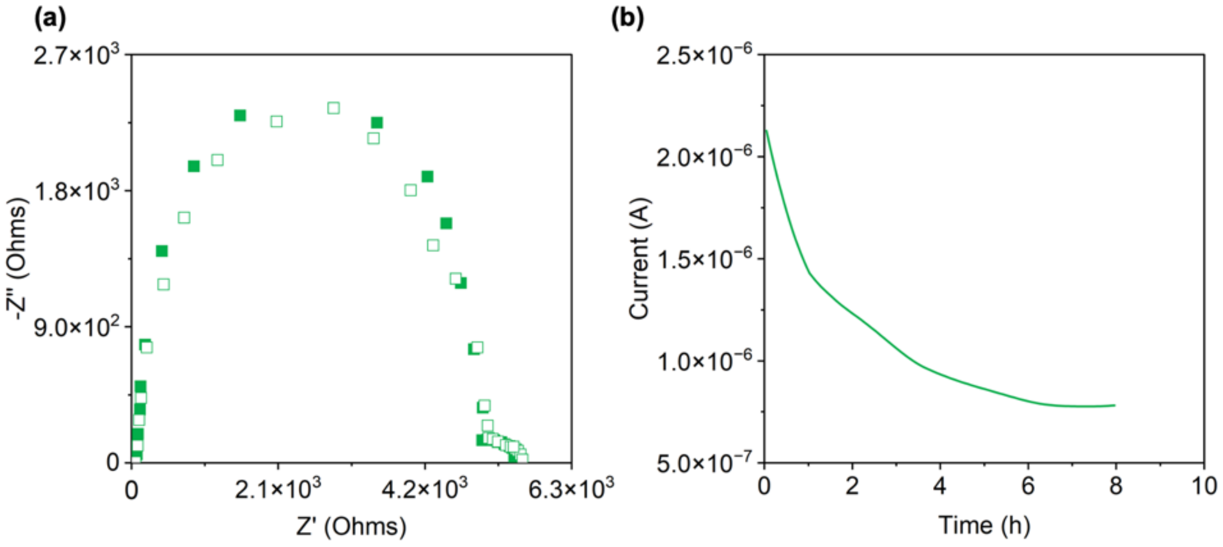

Figure S204. (a) Z'' as a function of Z' for lithium–lithium symmetric cell C before (filled squares) and after polarization (hollow squares). (b) Current *vs.* time during cell polarization.

8.8.4 POEM / PLiMTFSI / $LiClO_4$ blend; [EO]:[LiMTFSI]:[$LiClO_4$] = 10:0.40:0.60 (molar ratio)

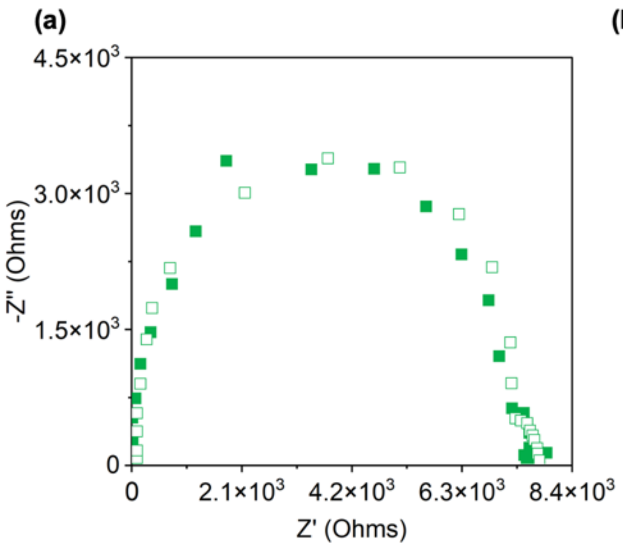

Figure S205. (a) Z'' as a function of Z' for lithium–lithium symmetric cell A before (filled squares) and after polarization (hollow squares). (b) Current *vs.* time during cell polarization.

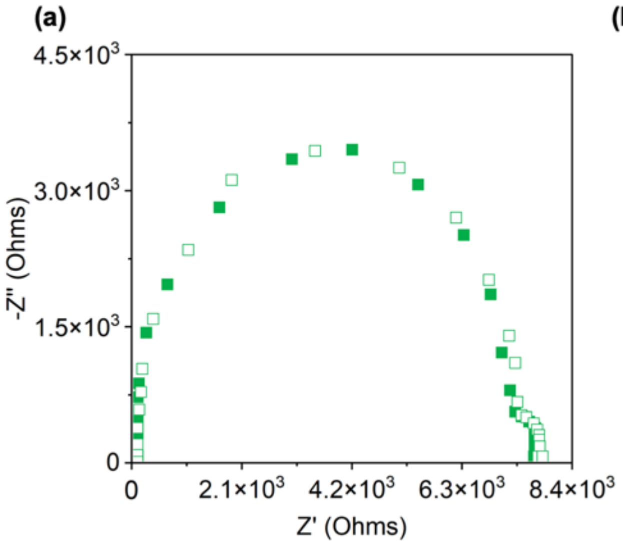

Figure S206. (a) Z'' as a function of Z' for lithium–lithium symmetric cell B before (filled squares) and after polarization (hollow squares). (b) Current *vs.* time during cell polarization.

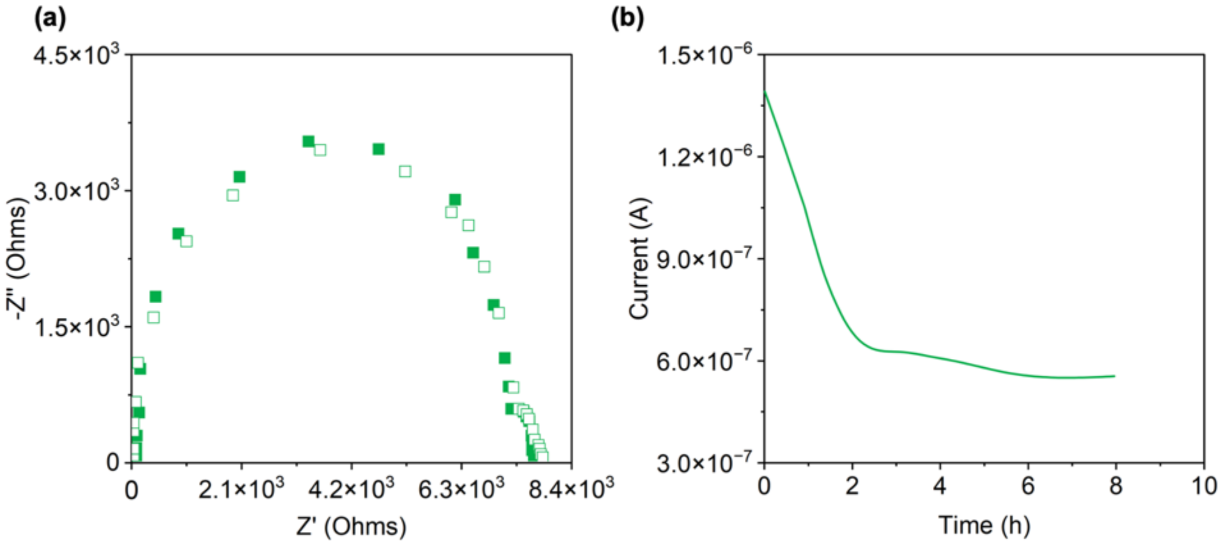

Figure S207. (a) Z'' as a function of Z' for lithium–lithium symmetric cell C before (filled squares) and after polarization (hollow squares). (b) Current *vs.* time during cell polarization.

8.8.5 POEM / PLiMTFSI / $LiClO_4$ blend; [EO]:[LiMTFSI]:[$LiClO_4$] = 10:0.50:0.50 (molar ratio)

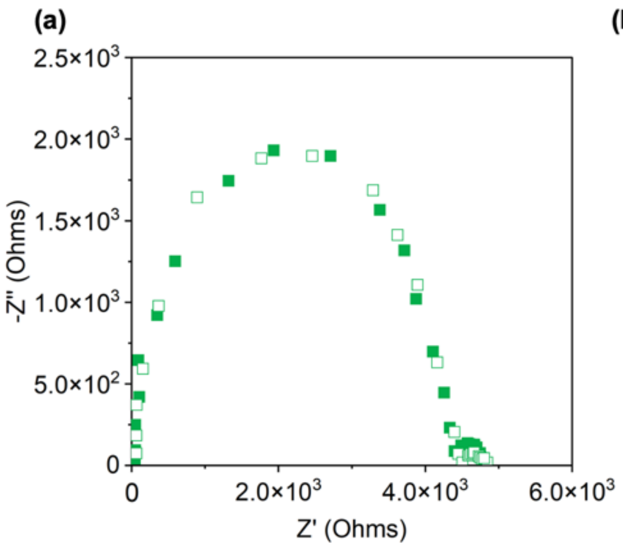

Figure S208. (a) Z'' as a function of Z' for lithium–lithium symmetric cell A before (filled squares) and after polarization (hollow squares). (b) Current *vs.* time during cell polarization.

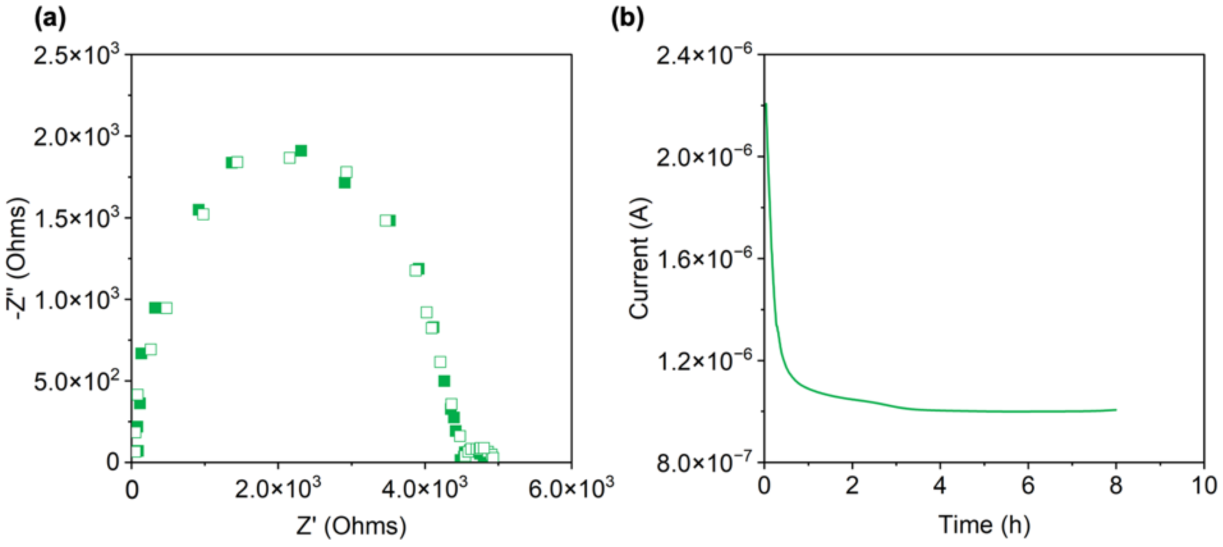

Figure S209. (a) Z'' as a function of Z' for lithium–lithium symmetric cell B before (filled squares) and after polarization (hollow squares). (b) Current *vs.* time during cell polarization.

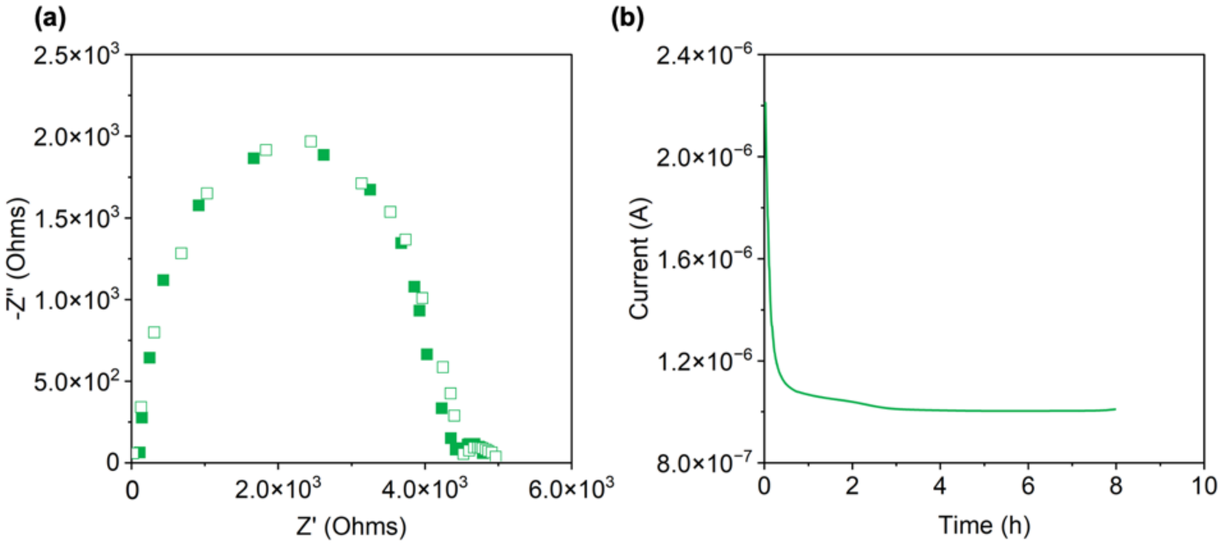

Figure S210. (a) Z'' as a function of Z' for lithium–lithium symmetric cell C before (filled squares) and after polarization (hollow squares). (b) Current *vs.* time during cell polarization.

8.8.6 POEM / PLiMTFSI / $LiClO_4$ blend; [EO]:[LiMTFSI]:[$LiClO_4$] = 10:0.60:0.40 (molar ratio)

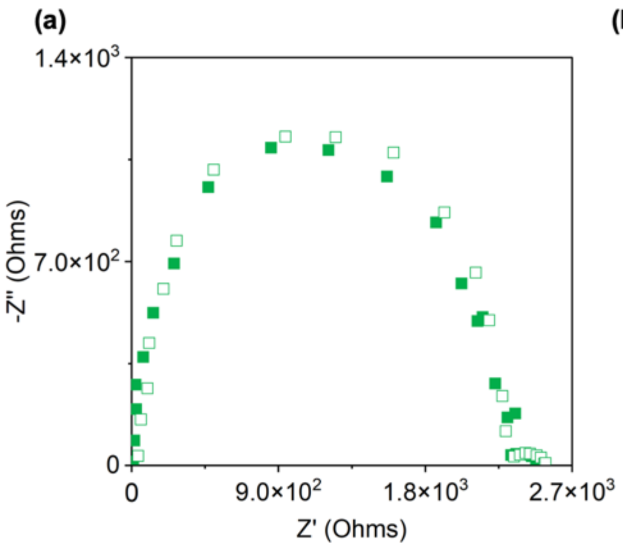

Figure S211. (a) Z'' as a function of Z' for lithium–lithium symmetric cell A before (filled squares) and after polarization (hollow squares). (b) Current *vs.* time during cell polarization.

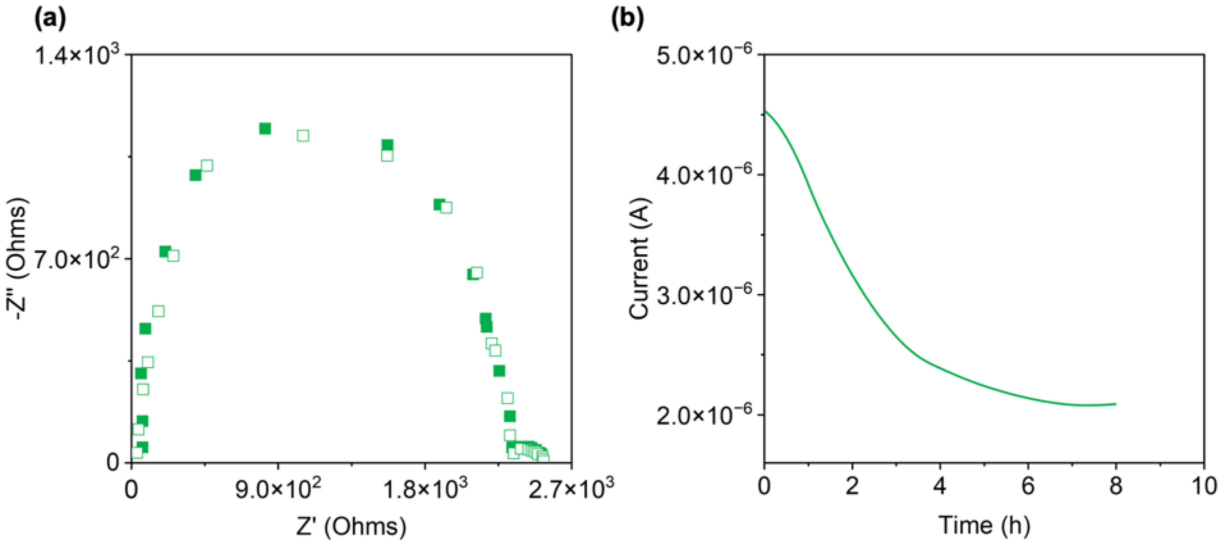

Figure S212. (a) Z'' as a function of Z' for lithium–lithium symmetric cell B before (filled squares) and after polarization (hollow squares). (b) Current *vs.* time during cell polarization.

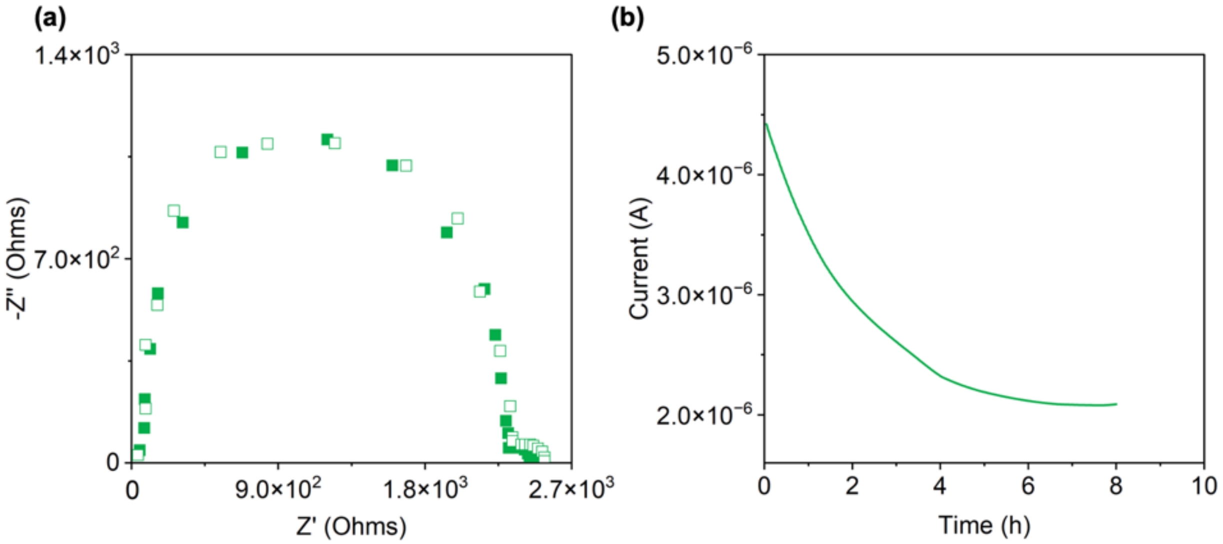

Figure S213. (a) Z'' as a function of Z' for lithium–lithium symmetric cell C before (filled squares) and after polarization (hollow squares). (b) Current *vs.* time during cell polarization.

8.8.7 POEM / PLiMTFSI / $LiClO_4$ blend; [EO]:[LiMTFSI]:[$LiClO_4$] = 10:0.80:0.20 (molar ratio)

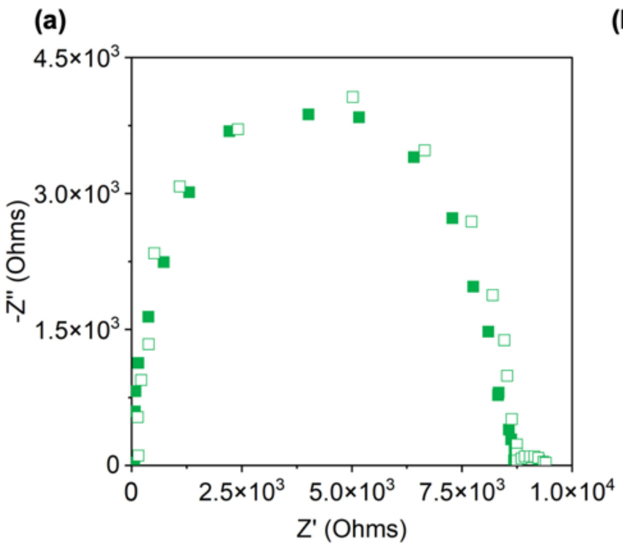

Figure S214. (a) Z'' as a function of Z' for lithium–lithium symmetric cell A before (filled squares) and after polarization (hollow squares). (b) Current *vs.* time during cell polarization.

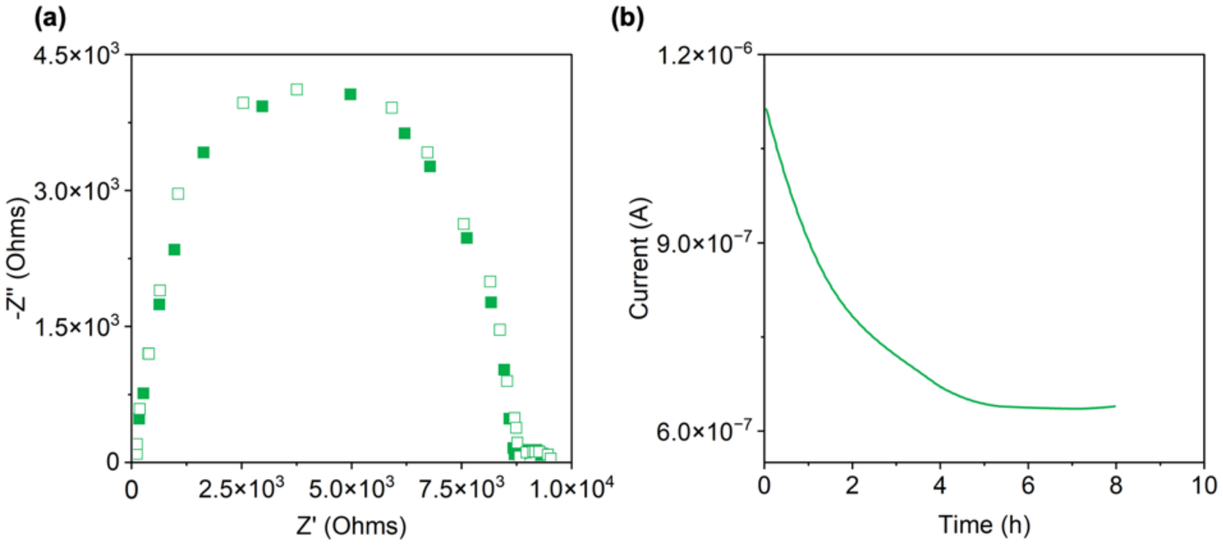

Figure S215. (a) Z'' as a function of Z' for lithium–lithium symmetric cell B before (filled squares) and after polarization (hollow squares). (b) Current *vs.* time during cell polarization.

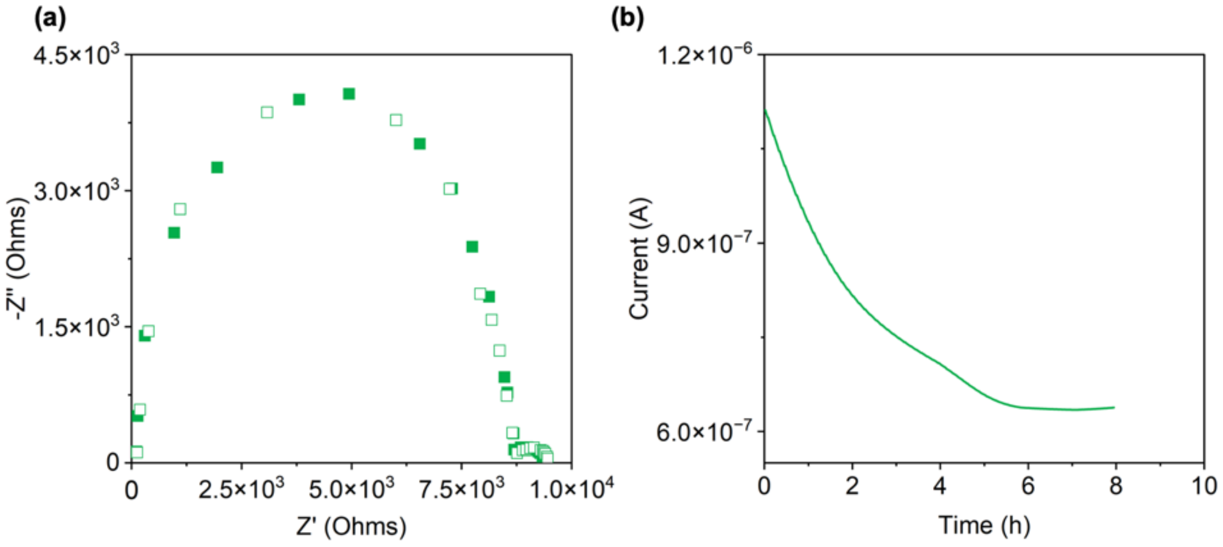

Figure S216. (a) Z'' as a function of Z' for lithium–lithium symmetric cell C before (filled squares) and after polarization (hollow squares). (b) Current *vs.* time during cell polarization.

Summary of the parameters for the Bruce-Vincent method for POEM / PLiMTFSI blend.

Table S32 (a). Impedance and current values extracted from AC impedance spectroscopy and potentiostatic polarization measurements of three lithium–lithium symmetric cells for POEM / PLiMTFSI blend at 60 °C.

	$^{b}R_1$ (Ohms)	$^{b}R_2$ (Ohms)	$^{c}R_1^*$ (Ohms)	$^{c}R_2^*$ (Ohms)	$^{d}R_0$ (Ohms)	$^{e}R_{SS}$ (Ohms)	$^{f}I_0$ (A)	I_{SS} (A)	t_{Li+}
^{a}Cell A	1.90×10^4	2.17×10^4	1.91×10^4	2.18×10^4	2730	2700	4.61×10^{-7}	4.42×10^{-7}	0.96
^{a}Cell B	1.87×10^4	2.16×10^4	1.91×10^4	2.20×10^4	2850	2820	4.63×10^{-7}	4.45×10^{-7}	0.96
^{a}Cell C	1.88×10^4	2.15×10^4	1.90×10^4	2.18×10^4	2730	2760	4.65×10^{-7}	4.46×10^{-7}	0.96

^{a}Cells A, B, and C were made with lithium non-blocking electrodes. $^{b}R_1$ and R_2 are the impedances of the minima at the bounds of the low-frequency semicircle in a Nyquist plot before cell polarization. $^{c}R_1$* and R_2* are the corresponding impedances after polarization. ^{d}Initial interfacial impedance was defined as $R_0 = R_2 - R_1$. ^{e}Steady-state interfacial impedance was defined as $R_{SS} = R_2^* - R_1^*$. ^{f}Initial current, I_0, was calculated by Ohm's law. This footnote applies to all tables in this section.

Table S32 (b). Impedance and current values extracted from AC impedance spectroscopy and potentiostatic polarization measurements of three lithium–lithium symmetric cells for POEM / PLiMTFSI blend at 100 °C.

	$^{b}R_1$ (Ohms)	$^{b}R_2$ (Ohms)	$^{c}R_1^*$ (Ohms)	$^{c}R_2^*$ (Ohms)	$^{d}R_0$ (Ohms)	$^{e}R_{SS}$ (Ohms)	$^{f}I_0$ (A)	I_{SS} (A)	t_{Li+}
^{a}Cell A	2690	2890	2740	2950	200	210	3.46×10^{-6}	3.32×10^{-6}	0.96
^{a}Cell B	2680	2900	2730	2940	220	210	3.45×10^{-6}	3.31×10^{-6}	0.96
^{a}Cell C	2700	2920	2720	2940	220	220	3.43×10^{-6}	3.32×10^{-6}	0.97

8.9 Measurement conducted at 60 °C for POEM / PLiMTFSI blend

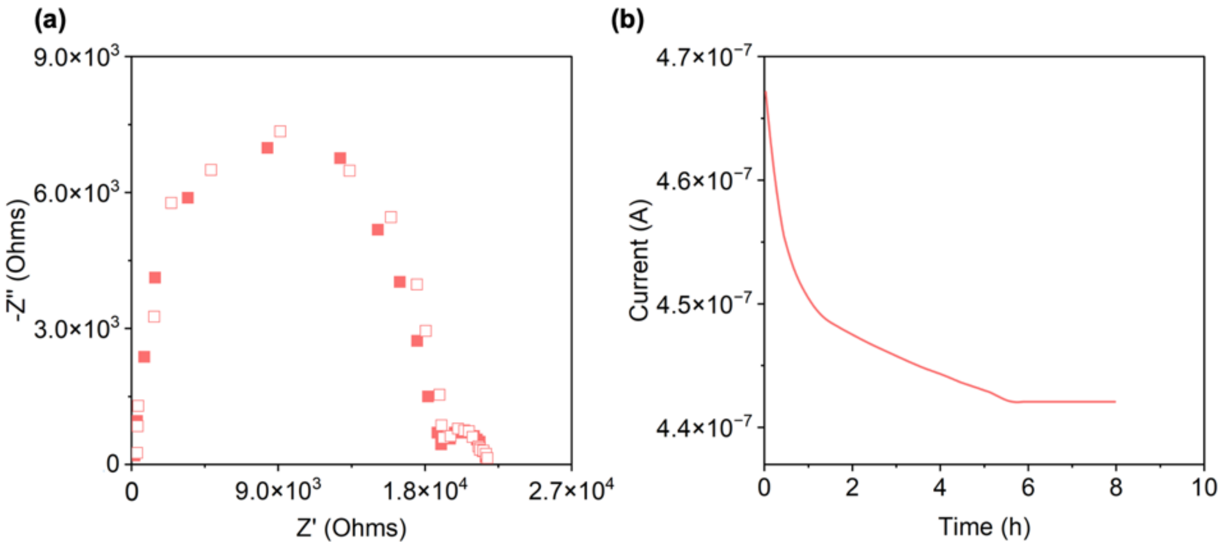

Figure S217. (a) Z″ as a function of Z′ for lithium–lithium symmetric cell A before (filled squares) and after polarization (hollow squares). (b) Current *vs.* time during cell polarization.

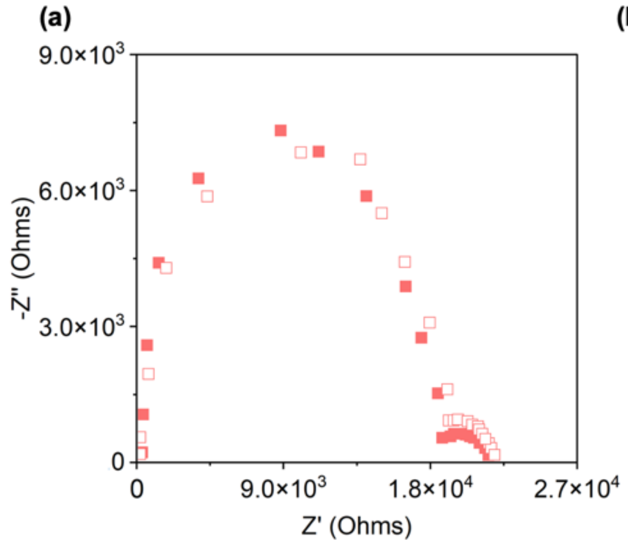

Figure S218. (a) Z'' as a function of Z' for lithium–lithium symmetric cell B before (filled squares) and after polarization (hollow squares). (b) Current *vs.* time during cell polarization.

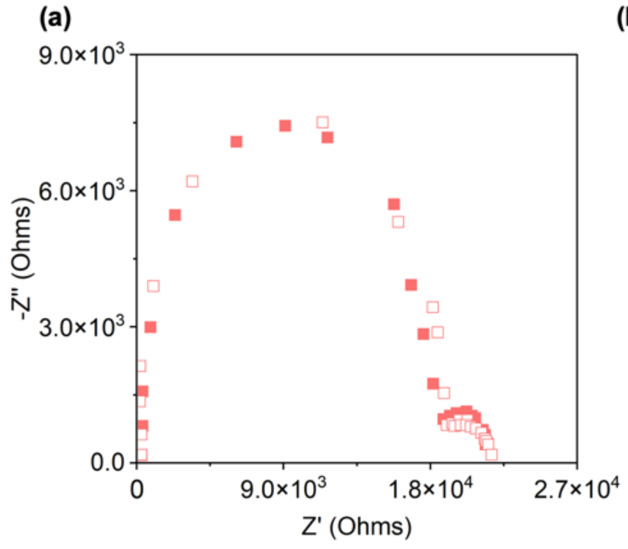

Figure S219. (a) Z'' as a function of Z' for lithium–lithium symmetric cell C before (filled squares) and after polarization (hollow squares). (b) Current *vs.* time during cell polarization.

8.10 Measurement conducted at 100 °C for POEM / PLiMTFSI blend

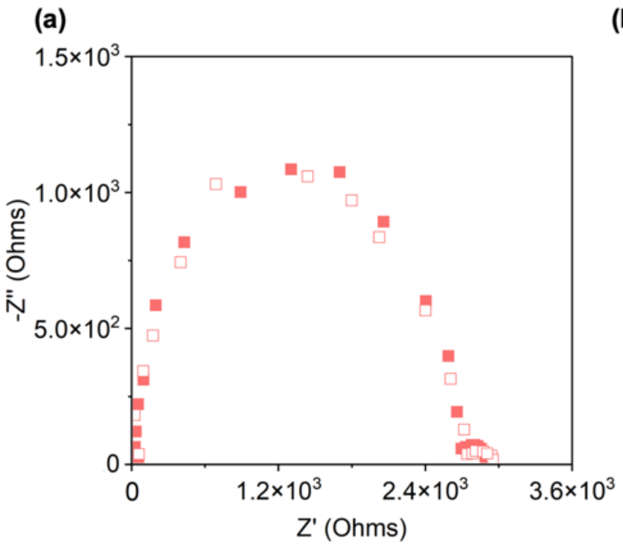

Figure S220. (a) Z″ as a function of Z′ for lithium–lithium symmetric cell A before (filled squares) and after polarization (hollow squares). (b) Current *vs.* time during cell polarization.

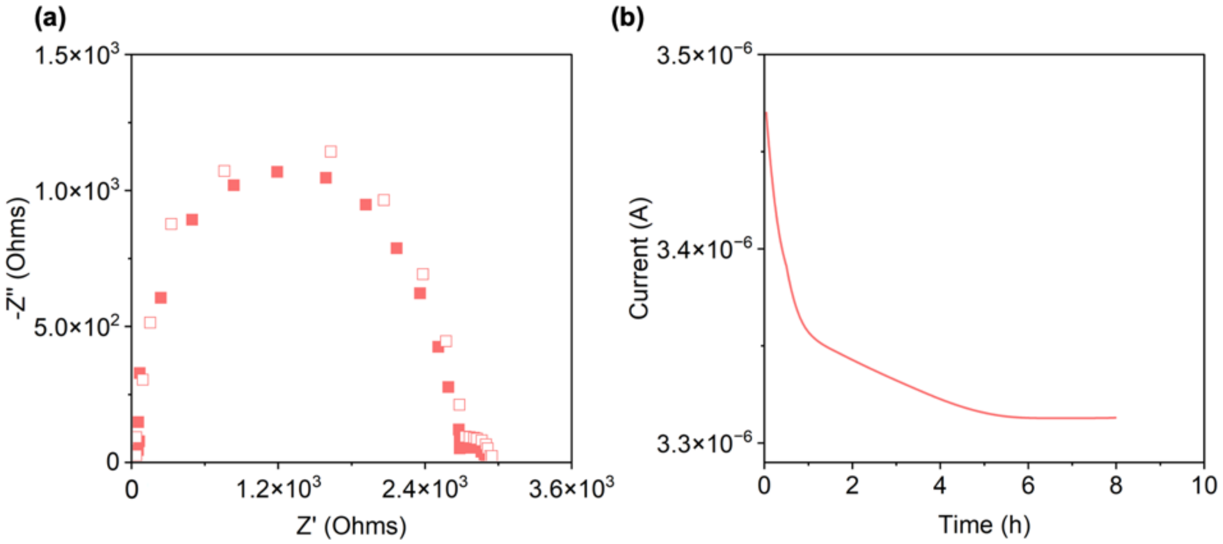

Figure S221. (a) Z'' as a function of Z' for lithium–lithium symmetric cell B before (filled squares) and after polarization (hollow squares). (b) Current *vs.* time during cell polarization.

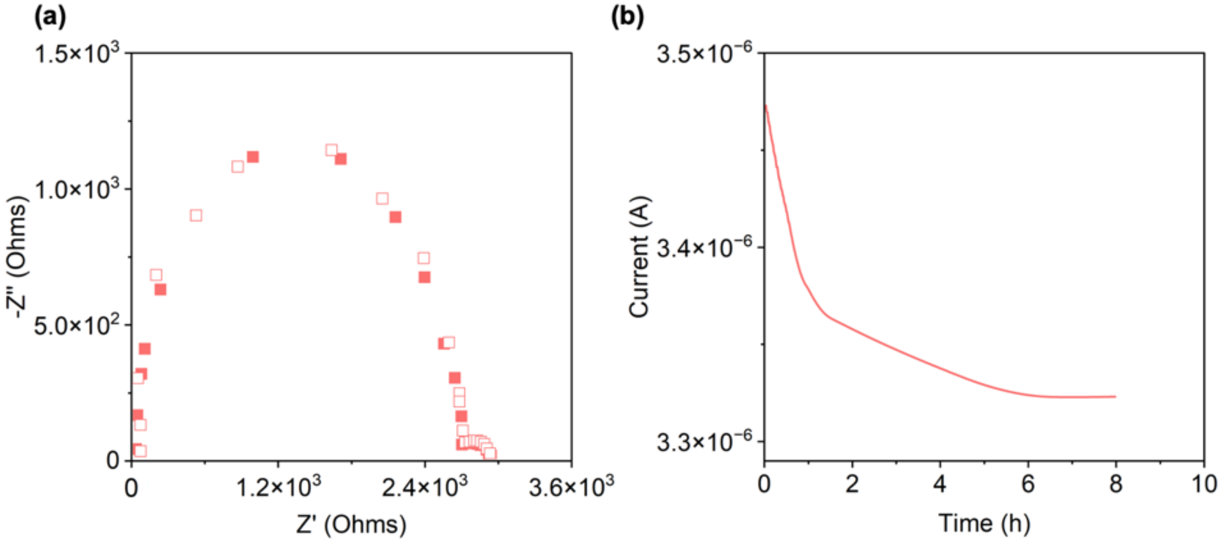

Figure S222. (a) Z'' as a function of Z' for lithium–lithium symmetric cell C before (filled squares) and after polarization (hollow squares). (b) Current *vs.* time during cell polarization.

References

1. Etkin, V. Modified Coulomb Law. *World Scientific News* **2017**, *87*, 163-174.
2. Shen, K.-H.; Hall, L. M. Ion Conductivity and Correlations in Model Salt-Doped Polymers: Effects of Interaction Strength and Concentration. *Macromolecules* **2020**, *53* (10), 3655-3668.
3. He, T.; Lu, Y.; Su, Y.; Bao, L.; Tan, J.; Chen, L.; Zhang, Q.; Li, W.; Chen, S.; Wu, F. Sufficient Utilization of Zirconium Ions to Improve the Structure and Surface Properties of Nickel-Rich Cathode Materials for Lithium-Ion Batteries. *ChemSusChem* **2018**, *11* (10), 1639-1648.
4. Demaison, J.; Wlodarczak, G. The Equilibrium C-H Bond Length. *Structural Chemistry* **1994**, *5* (1), 57-66.
